# Active Mechanical Threading by a Molecular Motor**

**DOI:** 10.1002/anie.202201882

**Published:** 2022-03-04

**Authors:** Nicolai N. Bach, Verena Josef, Harald Maid, Henry Dube

**Affiliations:** ^1^ Friedrich-Alexander-Universität Erlangen-Nürnberg Department of Chemistry and Pharmacy Nikolaus-Fiebiger-Str. 10 91058 Erlangen Germany

**Keywords:** Hemithioindigo, Indigoids, Molecular Machines, Molecular Motors, Photochemistry

## Abstract

Molecular motors transform external energy input into directional motions and offer exquisite precision for nano‐scale manipulations. To make full use of molecular motor capacities, their directional motions need to be transmitted and used for powering downstream molecular events. Here we present a macrocyclic molecular motor structure able to perform repetitive molecular threading of a flexible tetraethylene glycol chain through the macrocycle. This mechanical threading event is actively powered by the motor and leads to a direct translation of the unidirectional motor rotation into unidirectional translation motion (chain versus ring). The mechanism of the active mechanical threading is elucidated and the actual threading step is identified as a combined helix inversion and threading event. The established molecular machine function resembles the crucial step of macroscopic weaving or sewing processes and therefore offers a first entry point to a “molecular knitting” counterpart.

## Graphical abstract

A molecular machine is presented that uses the light‐powered unidirectional rotation trajectory of a molecular motor to actively thread a tetraethylene glycol chain through a macrocyclic ring. This molecular setup thus realizes the miniaturization of macroscopic threading providing a fundamental entry step into mechanical molecular weaving or sewing processes.

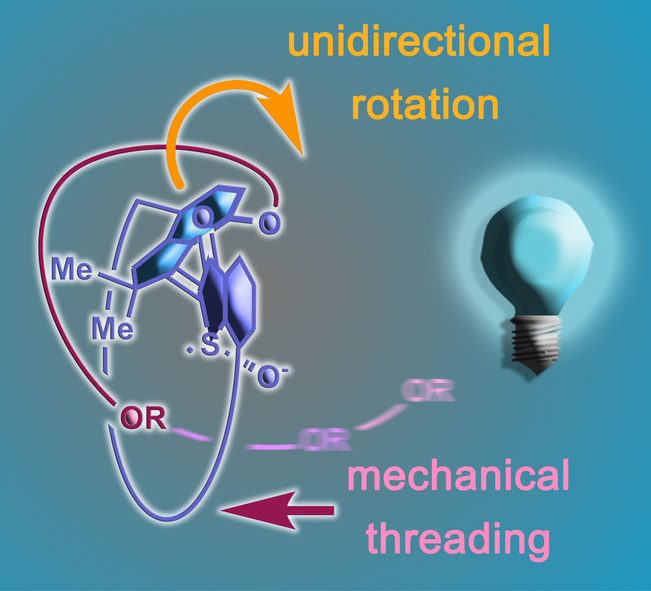

## Introduction

Molecular machines promise unprecedented control over the behavior of matter at the smallest scales.^[1]^ Central to this promise is their capacity to power and steer precise molecular motions in order to carry out their functions. Molecular motors are essential to this endeavor as they provide directional rotations powered by energy input.^[2]^ A number of different molecular motors are available nowadays, covering light‐powered versions as pioneered by Feringa and co‐workers,^[3]^ catenane‐based motors developed first by Leigh and co‐workers,^[4]^ and a number of chemically[^4d^, ^5^] or electrically^[6]^ fueled versions, as well as distinct types of light‐powered^[7]^ or mixed‐input^[8]^ systems. We have contributed to this quickly growing field by devising visible‐light‐driven molecular motors^[9]^ that operate similar to Feringa's sterically overcrowded stilbenes and further by developing two new archetypes of light driven molecular motors that function by very different mechanisms. In 2018 we have devised the first photon‐only driven molecular motor that becomes faster and more efficient instead of stalling at lower temperatures.^[10]^ In 2019 we presented a green light‐powered molecular motor providing a more complex figure‐of‐eight shaped directional motion.^[11]^

At the current frontier of research, motor function needs to be integrated with other molecular processes and their motions need to be transmitted to drive another level of molecular processes. Such integration has been done in liquid crystals,^[12]^ polymers,^[13]^ gels,^[14]^ supramolecular assemblies,^[15]^ 3D‐porous structures,^[16]^ by interfacing motors with biological cells^[17]^ or functionalized surfaces^[18]^ and nano‐particles.^[19]^ However, the precise transmission of the trajectories of directional motions to separate entities is a standing challenge at the molecular level where progress has just started. Some of the rare examples are the active entanglement of motor‐containing gel‐networks,[^14b^, ^14c^] locked synchronizing of molecular motions,^[20]^ the recent active crossing of imine bis‐macrocycles,^[21]^ or the directional transmission and acceleration of biaryl rotations by molecular motors.^[22]^ In the latter, a first macrocyclic version of molecular motors was established in which rotor and stator parts are connected by the ring structure (for a more recent variant using a Feringa motor within a macrocyclic structure see reference [23] and for a mechanically interlocked structure based on templating see reference [24]).

**Figure 1:**
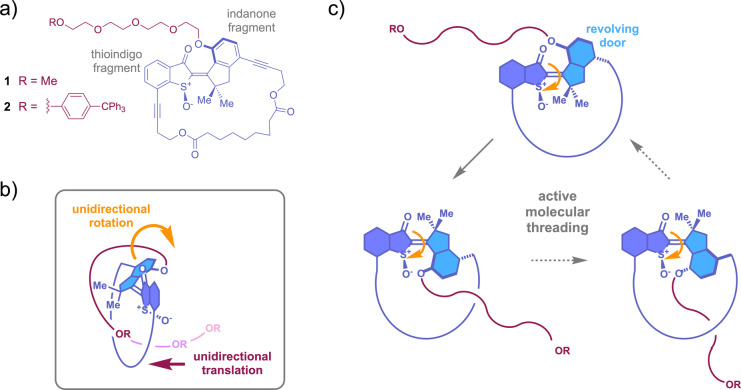
Mechanical and unidirectional threading of a TEG chain through a macrocycle powered by the operation of a molecular motor. a) Molecular structure of macrocyclic motors **1** and **2**. b) Active translation of the unidirectional rotation into a directional linear threading motion. c) Stepwise mechanism of motor‐powered threading. The rotor part (light blue) serves as revolving door dragging the attached TEG chain through the macrocycle during motor operation.

In this work we present a different macrocyclic molecular motor setup allowing for active and mechanical threading of a linear chain through a ring structure (Figure 1). The rotor part of the molecular motor represents a “revolving door” within the macrocycle that undergoes unidirectional rotation upon visible light fueling. Attachment of a tetraethylene‐glycol (TEG) chain to the rotor causes the active threading and pulling of the TEG chain through the macrocycle in a fully directional sense. By this process the unidirectional rotation of the motor is translated into an unidirectional linear motion in a mechanical and energy consuming way. This mechanism therefore provides an entry‐point to the translation of one directional motion type (rotation) into a fundamentally distinct one (translation). Different to established linear motors using intrinsic ratcheting mechanisms here the unidirectional linear motion is actively powered by a rotary molecular motor. The working principle of our molecular machine thus rather resembles a pumping mechanism than a classical linear molecular motor and does not use “passive” templating effects for interlocking.^[25]^ The threading process established here is also distinct from the previously reported back‐and‐forth sliding motion within a pre‐assembled rotaxane structure, which was powered either by a molecular motor^[26]^ or a stilbenoid switch.^[27]^

## Results and Discussion

Motor systems **1** and **2** consist of a hemithioindigo (HTI)^[28]^ molecular motor unit, which is integrated into a macrocyclic structure by connecting the indanone and the thioindigo fragments via an aliphatic diester chain. Attachment points for the aliphatic ring chain were chosen such, that a roughly 90° angle with respect to the photoisomerizable double bond is established at the thioindigo fragment and a roughly colinear arrangement at the indanone fragment. In this way the latter is defined as revolving door that should experience minimal resistance for its unidirectional rotation within the macrocycle. At the indanone fragment a flexible TEG chain is introduced bearing either a small methyl group (structure **1**) or a bulky tetraphenyl methane stopper group (structure **2**) at the end. For the integrated system **1** full motor operation was expected resulting in a continuous and repetitive threading of the TEG chain owed to the small diameter and flexibility of the latter. For system **2** the tetraphenyl methane group at the end was projected to serve as stopper moiety prohibiting full threading through and thus inducing a stalling of the motor operation. In this way the actual threading process could be evidenced and possible slipping modes avoiding entanglement of the TEG chain could be excluded by using derivative **2**. Most importantly, the specific stage in the motor rotation cycle constituting the threading step can be identified by the stopper unit as this would be the step in which stalling is expected to occur.

The syntheses of macrocyclic motors **1** and **2** are described in detail in the Supporting Information. Synthesis starts with condensation of brominated benzothiophenone **3** with indanone **4** and concomitant demethylation of the indanones hydroxy group yielding dibrominated HTI **5**. Introduction of the TEG chain at the indanone fragment yields HTI **6**, which is subjected to a subsequent twofold Sonogashira‐type cross‐coupling reaction to install two butynol alcohol functions. Macrocyclization via twofold esterification yielding the cyclic structure **7** is followed by a final oxidation of the sulfur to the corresponding sulfoxide to obtain cyclic motors **1** or **2**.

**Figure 2:**
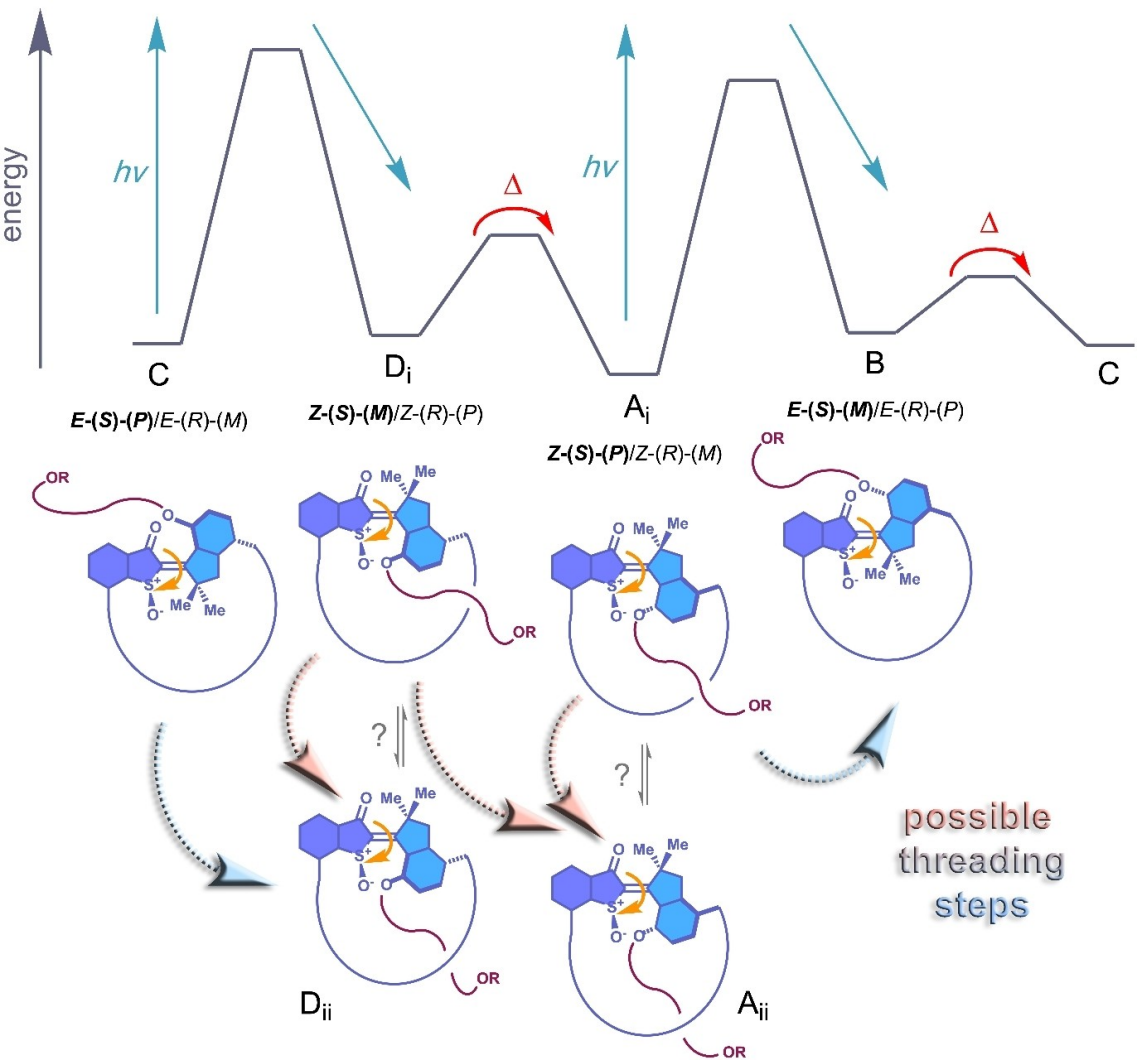
Qualitative energy profile for the operation mechanism of macrocyclic motors **1** or **2**. For isomers of type **A** and **D** two different structures (pre‐threaded **i** or already threaded **ii**) are possible. The actual threading step is possible for different isomer interconversion steps (dashed beveled arrows), either photochemically in a concerted fashion together with double bond isomerization (**C** to **D_ii_** or **A_i_** to **B** transition), as single thermally induced step (**A_i_** to **A_ii_** or **D_i_** to **D_ii_**), or in a concerted, thermally induced fashion together with helix inversion (**D_i_** to **A_ii_**).

After successful synthesis motor operation was scrutinized for both macrocycles **1** and **2**. Owed to their similarity to the first HTI motor system a four‐step rotation mechanism was expected in which two photoisomerization reactions are intersected by two respective thermal helix inversion steps (Figure 2). Two of the four states of such HTI motor are typically stable at ambient temperatures while two states are thermally instable and can only be observed with ultrafast spectroscopy methods or at low temperatures.[^9b^, ^9c^, ^9f^]

In accordance with that expectation it was possible to isolate macrocycle **1** in pure *Z* isomeric and in pure *E* isomeric form after HPLC purification. The two species could be identified as the stable racemic *Z*‐(*S*)‐(*P*)/*Z*‐(*R*)‐(*M*) and *E*‐(*S*)‐(*P*)/*E*‐(*R*)‐(*M*) isomer, respectively (see Supporting Information for details), which we term isomers **A‐1** and **C‐1**, respectively in the following for simplicity. Likewise, for macrocyclic motor **2** the two stable isomers **A‐2** and **C‐2** could be isolated and analyzed in pure form. In Figure 2 schematic representations of all possible isomers **A** to **D** for motors **1** and **2** are shown together with their individual stereochemical assignments. It is important to note that the *Z* configured isomers **A** and **D** can assume two possible conformations termed **i** and **ii** with the flexible TEG chain being positioned at either side of the macrocyclic ring structure (either at the same or the opposite side as the sulfoxide‐oxygen position). For isomers **B** and **C** the *E* isomeric configuration and flexibility of the chain preclude such defined positioning with respect to the two macrocycle sides and only one conformation is expected in each case. Given this structural setup of the macrocyclic motor different possibilities exist for the active threading step to proceed. The crucial process can be elicited either photochemically in a combined fashion (double bond photoisomerization and threading as shown by dashed light blue arrows in Figure 2) by transitioning from isomer **C** to **D_ii_** or **A_i_** to **B** or in a thermally activated process (shown in dashed light red arrows in Figure 2). For the latter two more possibilities exist, either an isolated threading event without motor helicity inversion, i.e. transitioning from **D_i_** to **D_ii_** or from **A_i_** to **A_ii_**, or a coupled event in which motor helicity inversion is coupled to threading by transitioning from **D_i_** to **A_ii_**.

**Figure 3:**
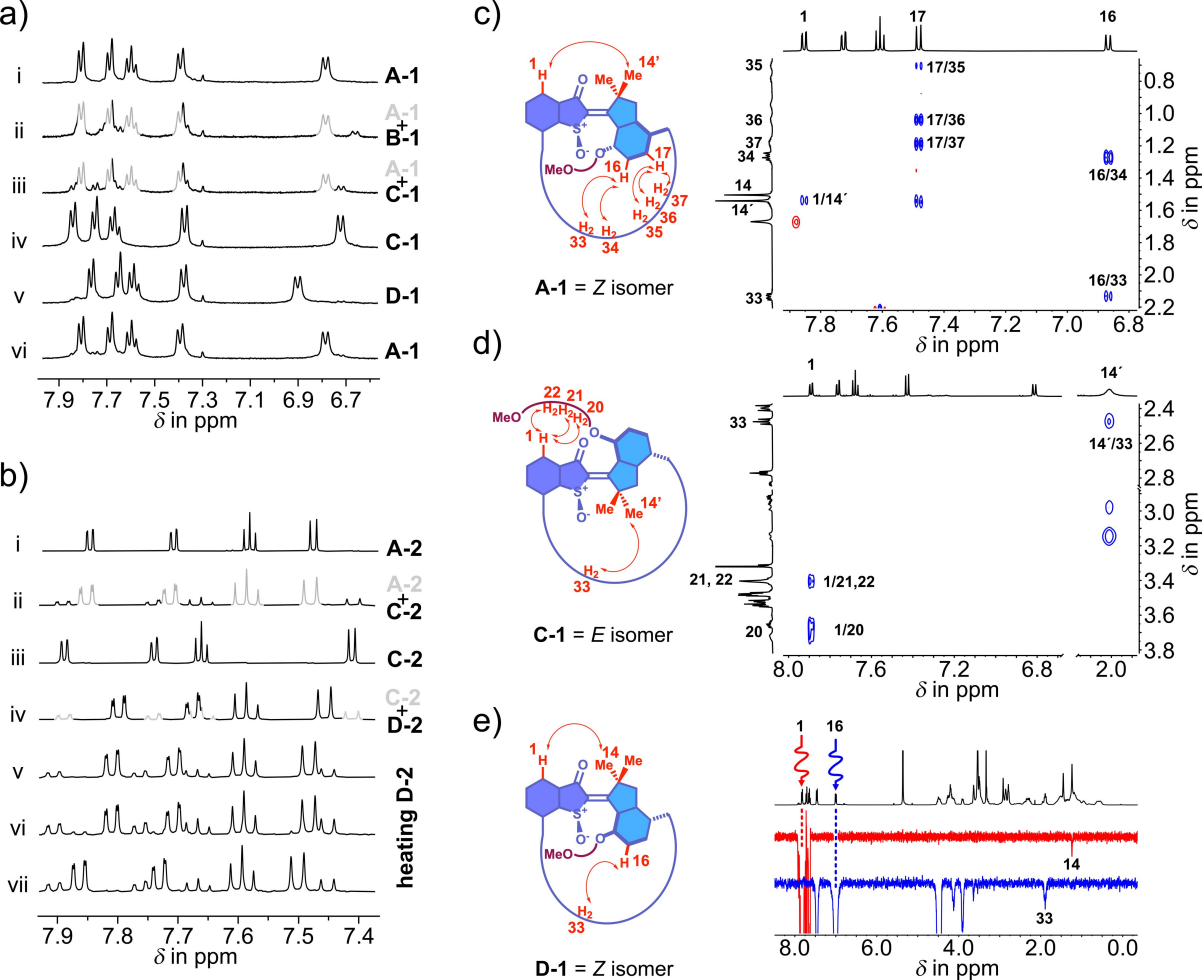
Photochemical and thermal interconversions of macrocyclic motors **1** and **2** and spectral signatures used to identify the nature of the different isomers. a) ^1^H NMR (400 MHz, CD_2_Cl_2_/CS_2_=4/1 solution) spectra recorded during a full cycle of motor **1** rotation. Spectrum i: Starting point of pure **A‐1**. ii: After irradiation of **A‐1** with 405 nm light at −105 °C isomer **B‐1** is obtained in small amounts. iii: After thermal annealing isomer **B‐1** converted exclusively to **C‐1**. iv: Starting point of pure **C‐1**. v: After irradiation of **C‐1** with 450 nm light at −105 °C isomer **D‐1** is obtained almost quantitatively. iv: After thermal annealing isomer **D‐1** converted exclusively to **A‐1**. b) ^1^H NMR (400 MHz, CD_2_Cl_2_ (i to iv) and C_2_D_2_Cl_4_ (v to vii) solution) spectra recorded during operation of macrocycle **2**. Spectrum i: Starting point of pure **A‐2**. ii: After irradiation with 405 nm light at 22 °C isomer **C‐2** is obtained. iii: Starting point of pure **C‐2**. iv: After irradiation of **C‐2** with 450 nm light at 22 °C isomer **D‐2** is accumulated strongly. v to vii: Prolonged heating at 65 °C converts **D‐2** first to **C‐2** and then to **A‐2** (see also Supporting Information). c) Segment of the NOESY spectrum evidencing *Z* configuration of **A‐1**. d) Segment of the NOESY spectrum evidencing *E* configuration of **1**‐**C**. e) NOE spectra evidencing *Z* configuration of **1‐D**.

**Figure 4:**
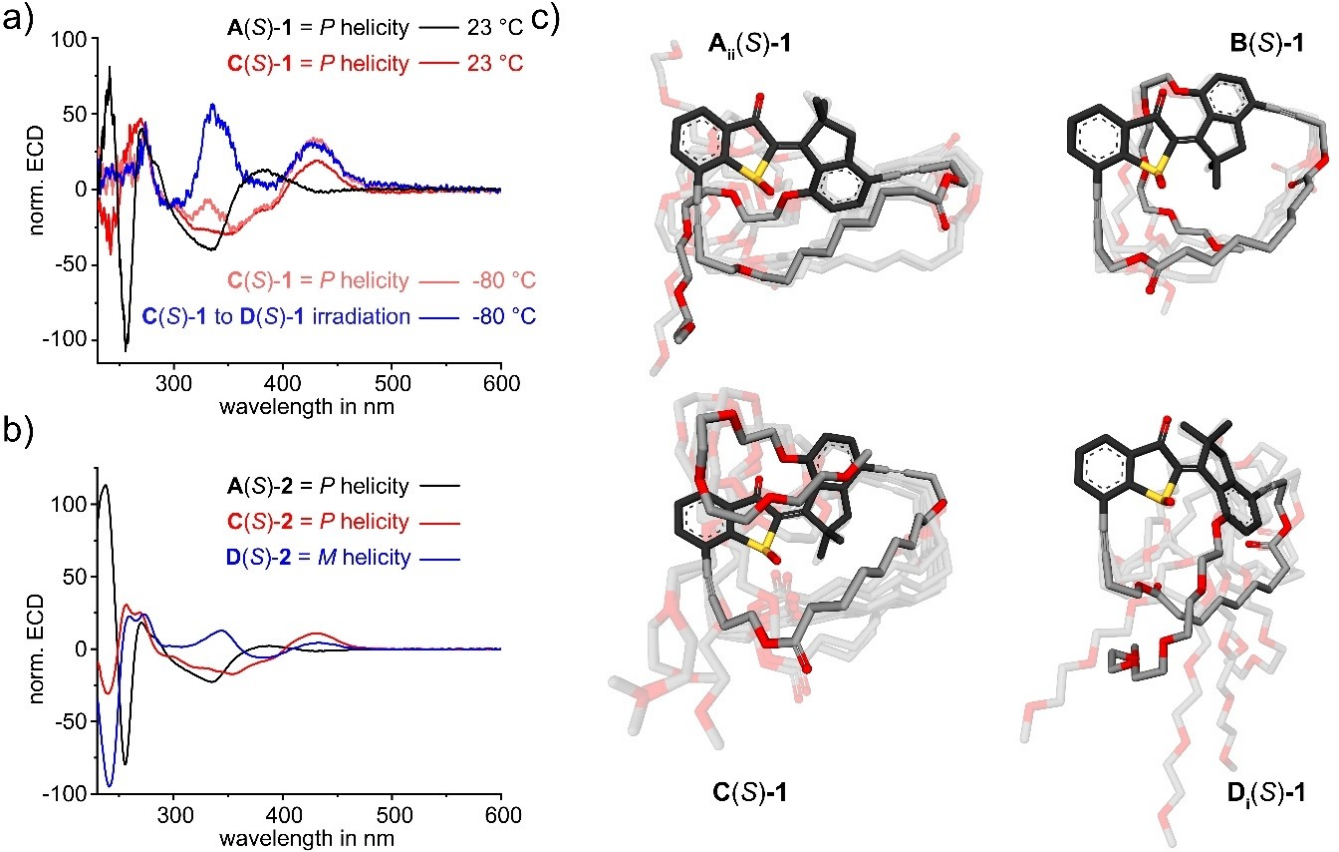
a) Experimental ECD spectra for the two stable isomers **A** and **C** of HTI motor **1** possessing (*S*) configuration at the sulfoxide stereocenter. The helicity assignment is in good agreement with the theoretical description. Spectra were measured in CH_2_Cl_2_ solution at different indicated temperatures. Spectral changes upon irradiation of **C‐2** at low temperatures show the hallmarks of a **D**‐isomeric structure with inverted helicity (pink versus blue spectrum, also compare to the corresponding spectra of **C‐2** and **D‐2** shown in subfigure (b). b) Comparison of experimental ECD spectra for the three stable isomers **A**, **C**, and **D** of HTI motor **2** possessing (*S*) configuration at the sulfoxide stereocenter in CH_2_Cl_2_ solution at 25 °C. Spectra of isomers **C‐2** and **D‐2** were originally measured for the (*R*) configured isomers and are mirrored to allow direct comparison with the spectra of **1** shown in sub‐Figure (a). c) Low energy minimum structures of isomers **A** to **D** of HTI motor **1** obtained from the theoretical analysis on the B3LYP‐D3BJ/6‐311G(d,p) IEFPCM(CH_2_Cl_2_) level of theory. From the minimum structures the Boltzmann‐averaged ECD spectra were calculated on the TD‐DFT B3LYP‐D3BJ/6‐311+G(d,p,) IEFPCM(CH_2_Cl_2_) level of theory. The global minimum structures are emphasized and the HTI motor component shown in darker colors.

The motions of macrocyclic motors **1** and **2** could be scrutinized in detail mainly by UV/Vis and NMR spectroscopy. In Figure 3a the corresponding ^1^H NMR spectra recorded during a full rotation cycle of **1** are shown and in Figure 3b the corresponding ^1^H NMR spectra recorded during operation of macrocyclic motor **2** are given. In Figure 3c, d segments of NOESY and NOE spectra are shown evidencing individual configurations of the double bond in isomers **A‐1**, **C‐1**, and **D‐1**. In Figure 4a, b ECD spectra of individual isomers with (*S*) configuration of the sulfoxide stereocenter are shown, which evidence the particular helicity of the motor moieties. For further details on structure assignments of different isomers we refer the reader to the Supporting Information.

At ambient temperatures photoirradiation of **A‐1** leads to interconversion with a second isomer, that was identified as *E* configured isomer **C‐1**. Irradiation of pure **A‐1** at −105 °C however, led to the population of a different isomer in up to 20 %, which was assigned to the second *E* configured isomer **B‐1** (Figure 3a i, ii). Upon warming up the solution to −80 °C isomer **B‐1** decayed completely and solely into isomer **C‐1** (Figure 3a ii, iii) with first order kinetics. The corresponding Gibbs energy of activation was found to be Δ*G* ^≠^=14.1 kcal mol^−1^, which is somewhat higher than the 13.1 kcal mol^−1^ measured for the corresponding barrier for the parent first generation HTI motor entity without the macrocycle and bearing a methoxy group instead of a TEG chain.^[9a]^ However, given the considerable structural differences between the original HTI motor and macrocycle **1** the rather small differences in the energy barrier for this thermal helix inversion are noteworthy.

Irradiation of isomer **C‐1** at ambient temperatures produces only one distinct isomer **A‐1** and no second isomer is seen under these conditions. However, at −105 °C irradiation of pure **C‐1** leads again to population of a different, fourth isomer in up to 90 % (Figure 3a iv, v). This isomer also possesses *Z* configuration of the double bond (Figure 3e) and therefore could be either a second **A‐1** isomer (**A_i_‐1** or **A_ii_‐1**) or one of the two possible **D** isomers (**D_i_‐1** or **D_ii_‐1**). It was clearly identified as a **D** isomer by a combined ECD, 1D, and 2D NMR analysis as well as comparison with the behavior of stoppered macrocyclic motor **2** (see Figure 4a, b for ECD analysis and the Supporting Information for further details). To exemplify, a solution of enantiomerically pure **C‐1** was irradiated at −80 °C and the ECD spectral changes were recorded at the low temperature. The ECD spectrum obtained upon photoconversion of **C**‐**1** showed a consistent sign‐reversal when compared to the corresponding **A**‐**1** ECD spectrum, which corroborates helicity inversion between **A**‐**1** and the photoproduct of **C**‐**1** irradiation, i.e. **D**‐**1** (Figure 4a). A final thermal step fully converts the **D‐1** isomer to the previously already observed **A‐1** isomer at −50 °C (Figure 3a v, vi). Taking the results of this analysis together a four‐step motor mechanism is elucidated for macrocycle **1**, which resembles the established mechanism of HTI molecular motors of the first generation. Similar to the first reported HTI motor, isomer **A** with *Z* configuration of the double bond is the thermodynamically most stable isomer. However, in case of macrocyclic motor **1** all four isomers populated during the rotation cycle in fixed sequence are directly observable with variable temperature NMR spectroscopy. In the first HTI motor isomer **D** was not visible even at −105 °C because of a very low activation barrier for its thermal helix inversion leading to isomer **A**.^[9a]^ Isomer **D** is thus significantly stabilized in the macrocycle of **1** (with a corresponding Gibbs energy of activation Δ*G* ^≠^=16.6 kcal mol^−1^ for **D** to **A** conversion in **1** as compared to 5.6 kcal mol^−1^ in the original HTI motor for the corresponding helix inversion process; see Supporting Information for details of the kinetic analysis). This behavior illustrates a very strong effect of the macrocyclic structure on motor rotation in **1**. Likewise, the Gibbs energy of activation Δ*G* ^≠^ for the thermal helix inversion from **B** to **C** is increased but to a significantly lesser degree (Δ*G* ^≠^=14.1 kcal mol^−1^ for **B** to **C** conversion in **1** as compared to 13.1 kcal mol^−1^ in the original HTI motor; see Supporting Information for details of the kinetic analysis). This macrocyclic effect is also significantly larger as compared to an earlier macrocyclic HTI motor system, where the thioindigo fragment serves as revolving door instead of the indanone rotor fragment.^[22]^ Despite the apparent hindrance of motor rotation in **1**, this motion still proceeds in a fully directional manner and after four steps the initial starting isomer is recovered. This behavior therefore directly evidences that the flexible TEG chain is actively threaded through the macrocycle to allow motor rotation to proceed repetitively.

However, at this stage it is not unraveled at what step the active threading actually happens. As outlined above this is conceivable at various stages of the motor cycle. Since only four different states were observed (corresponding to the motor configurations **A‐1** to **D‐1**) even at low temperatures, the presence of threading equilibria in any of those states are highly unlikely. Consistent with our observations is a full conversion from a state prior threading and a next state corresponding to the completely threaded through structure. Therefore, we attempted to elucidate the particular step in the motor rotation cycle at which the threading actually occurs and analyzed the rotation behavior of molecular motor **2** bearing a stopper moiety at the end of the flexible TEG chain.

In case of motor **2** a distinctly different behavior was observed. Again, a number of analytical methods most prominently NMR (see e.g. Figure 3b) and ECD (see Figure 4b) analysis allowed us to identify the nature of different isomers of **2** (see also the Supporting Information for further details). Isomers **A‐2** and **C‐2** could be isolated after HPLC purification and scrutinized in pure form. Irradiation of **A‐2** at ambient temperatures leads to population of **C‐2** (Figure 3b i, ii), which allowed us to assign the starting *Z* configured isomer as **A_ii_‐2** since only this configuration allows motor rotation to proceed photochemically and at ambient temperatures. The intermediate isomer **B** could not be evidenced directly at low temperatures as no productive photochemistry was observed at temperatures below −60 °C. If a similar Gibbs energy of activation for the conversion of **B‐2** to **C‐2** is assumed as in the case of **B‐1** to **C‐1** conversion, then no population of **B‐1** should be visible at temperatures above −60 °C hampering a direct observation. Irradiation of isomer **C‐2** at ambient temperatures leads exclusively to population of **D_i_‐2** (see Figure 3b iii, iv as well as Figure 4b for the corresponding ECD spectrum evidencing the inverted helicity as compared to **A‐2**). **D_i_‐2** is the only possible product of directional motor rotation as full threading through the macrocycle to reach isomer **D_ii_‐2** is inhibited by the bulky stopper group. Isomer **D_i_‐2** can be accumulated in up to 80 %. However, we did not observe any thermal decay of **D_i_‐2** to a helix‐inverted isomer **A‐2** even at elevated temperatures. In fact, the typically highly labile and transient **D** isomer is now kinetically inert in macrocycle **2**. When temperature elevation to 65 °C finally allows for thermal double bond isomerization a transition from **D_i_‐2** to **C‐2** is observed (see Figure 3b v, vi) and at a later time isomer **A_ii_‐2** is formed as the thermodynamically most stable state (see Figure 3b vi, vii). This isomerization process is thus reversing the inherent motor direction to reach the starting state. Taking these results together it becomes apparent that molecular motor **2** is stalled at isomer **D_i_‐2** and further conversion from **D_i_‐2** to either a threaded through **D_ii_‐2** or an isomer of type **A** (to maintain the directional motor rotation) is inhibited by the bulky stopper group under ambient conditions. A significant stabilization of the usually fleeting **D** state is observed beyond the previously described macrocyclic effect in **1**. Now **D_i_‐2** is stable even at ambient temperatures signifying a severe inhibition of its thermal helix inversion to reach the next isomeric state within the motor cycle. For macrocyclic motor **2** the observable isomers and their interconversions could thus be fully elucidated and a directional rotation sequence starting from **A_ii_‐2** and moving to **C‐2** (via fleeting isomer **B‐2**) to finally **D_i_‐2**, where rotation stops, could be established. Heating to elevated temperatures leads to a thermal reversal of the rotation direction and a repopulation of **A_ii_‐2**.

When comparing the spectral hallmarks of isomers **A_ii_‐2** and **D_i_‐2** with the corresponding isomers **A‐1** and **D‐1**, it becomes apparent that they possess very similar signatures (for direct comparisons of ECD spectra see Figure 4a, b, for UV/Vis and ^1^H NMR spectra see the Supporting Information). Both **A** isomers exhibit essentially the same signal positions in their ^1^H NMR spectra, which allows specifying that the configuration in macrocyle **1** is in fact **A_ii_‐1**. Similarly, the second *Z* configured isomer of **1** could be assigned to **D_i_‐1** owed again to the very similar spectral signatures as compared to **D_i_‐2** in the ^1^H NMR spectra. In addition, ECD spectroscopy proved to be highly effective for reporting the helicity of the motor structure (see Figure 4a, b), which helped to directly distinguish between isomers of type **A** and type **D** as they possess inverted helicities. Since the second *Z* configured isomer of macrocyclic motor **2** showed inverted helicity in its ECD spectrum as compared to **A_ii_‐2** it could confidently be assigned to a **D** type isomer (Figure 4b).

Further evidence for individual isomer assignments of macrocyclic system **1** were obtained from its theoretical description (Figure 4c). Optimization of the different isomer structures was conducted in a step‐by‐step procedure. First a conformational Monte‐Carlo search on the MMFF level of theory was used, which delivered starting structures for full DFT optimizations on the B3LYP‐D3BJ/6‐311G(d,p) IEFPCM(CH_2_Cl_2_) level of theory. The lowest energy structures thus obtained for each isomer are shown in Figure 4. Although up to 11 different structures with similar energies had to be taken into account to describe the corresponding spectra, the structural differences are mainly located at the flexible TEG chain conformation, while the central HTI chromophore remained essentially the same. Therefore, especially the electronic excitation spectra are dominated by the HTI structure in the spectral range >300 nm and can be used to elucidate e.g. molecular helicities with good confidence (see the Supporting Information for superpositions of experimentally obtained and calculated ECD spectra).

Two conclusions can thus be drawn from the behavior of macrocyclic motors **1** and **2**: first isomers **B**, **C**, and **D_i_**, represent structures in which the TEG chain is not threaded through but resides outside of the macrocycle at the same half‐space in which the rotor fragment is residing—i.e. in a “non‐threaded” state. Second the thermal **D_i_** to **A_ii_** transition represents the threading step in which the TEG chain is translocated, a process only possible in motor system **1** but not in the stoppered system **2**. This interpretation is strengthened by the fact that the thermal helix inversion step in HTI motors represents a much smaller motion as opposed to a photochemical double bond isomerization step. Despite the correspondingly expected smaller structural and energetic changes of helix inversion within the macrocycle of **2**, an effective stalling is observed in exactly this step, which hints at a severe destabilization of a frozen “threaded” structure **A_i_‐2**. In the macrocycle **1** the **A_i_** isomer can stabilize itself by allowing the threading to complete reaching state **A_ii_** ‐ and thus the motor cycle can be repeated and is not inhibited. Correspondingly, the transition from the type **D** to the type **A** isomer in **1** encounters an unusually high Gibbs energy of activation of 16.6 kcal mol^−1^. From the data gathered it is thus evident that only four states are populated during the rotation cycle of **1** and the threading step is coupled to the thermal helix inversion of the *Z* configured isomers.

## Conclusion

In summary, we show in this work how the unidirectional rotation of a molecular motor can be used to actively thread a linear chain fragment through a macrocyclic ring in a fully directional and repetitive manner. This process transforms a directional rotary motion into a directional linear motion between two molecular fragments (ring and chain thread) and is powered by light energy. Using a derivative bearing a bulky stopper group—an analogue of macroscopic reels—the actual threading step could be identified as the thermal helix inversion between the two *Z* isomeric motor states **D_i_** to **A_ii_**. These results have provided the first entry point into coupling the directional molecular motor rotation to a mechanical threading event akin to macroscopic weaving or sewing processes. We expect that such concept opens up an entirely new prospect for applying molecular motors as central powering units controlling mechanical manipulation of molecular structures and thus for gaining the highest precision in tailoring matter from the bottom up.

## Conflict of interest

The authors declare no conflict of interest.

1

## Supporting information

As a service to our authors and readers, this journal provides supporting information supplied by the authors. Such materials are peer reviewed and may be re‐organized for online delivery, but are not copy‐edited or typeset. Technical support issues arising from supporting information (other than missing files) should be addressed to the authors.

Supporting Information

Supporting Information

Supporting Information

## Data Availability

The data that support the findings of this study are available from the corresponding author upon reasonable request.

## References

[1a] E. R. Kay , D. A. Leigh , F. Zerbetto , Angew. Chem. Int. Ed. 2007, 46, 72–191; 10.1002/anie.200504313 17133632

[1b] S. Erbas-Cakmak , D. A. Leigh , C. T. McTernan , A. L. Nussbaumer , Chem. Rev. 2015, 115, 10081–10206; 26346838 10.1021/acs.chemrev.5b00146 PMC4585175

[1c] J. F. Stoddart , Angew. Chem. Int. Ed. 2017, 56, 11094–11125; 10.1002/anie.201703216 28815900

[1d] A. Coskun , M. Banaszak , R. D. Astumian , J. F. Stoddart , B. A. Grzybowski , Chem. Soc. Rev. 2012, 41, 19–30; 22116531 10.1039/c1cs15262a

[1e] C. Pezzato , C. Cheng , J. F. Stoddart , R. D. Astumian , Chem. Soc. Rev. 2017, 46, 5491–5507; 28338143 10.1039/c7cs00068e

[1f] B. L. Feringa , Angew. Chem. Int. Ed. 2017, 56, 11060–11078; 10.1002/anie.201702979 28851050

[1g] J. P. Sauvage , Angew. Chem. Int. Ed. 2017, 56, 11080–11093; 10.1002/anie.201702992 28632333

[2a] D. R. S. Pooler , A. S. Lubbe , S. Crespi , B. L. Feringa , Chem. Sci. 2021, 12, 14964–14986; 34909140 10.1039/d1sc04781g PMC8612399

[2b] S. Kassem , T. van Leeuwen , A. S. Lubbe , M. R. Wilson , B. L. Feringa , D. A. Leigh , Chem. Soc. Rev. 2017, 46, 2592–2621; 28426052 10.1039/c7cs00245a

[2c] D. Roke , S. J. Wezenberg , B. L. Feringa , Proc. Natl. Acad. Sci. USA 2018, 115, 9423–9431. 29712825 10.1073/pnas.1712784115 PMC6156651

[3a] N. Koumura , R. W. J. Zijlstra , R. A. van Delden , B. L. Feringa , Nature 1999, 401, 152–155; 10490022 10.1038/43646

[3b] M. M. Pollard , M. Klok , D. Pijper , B. L. Feringa , Adv. Funct. Mater. 2007, 17, 718–729;

[3c] H. A. Kistemaker , P. Stacko , J. Visser , B. L. Feringa , Nat. Chem. 2015, 7, 890–896; 26492009 10.1038/nchem.2362

[3d] T. van Leeuwen , A. S. Lubbe , P. Štacko , S. J. Wezenberg , B. L. Feringa , Nat. Chem. Rev. 2017, 1, 0096.

[4a] J. V. Hernández , E. R. Kay , D. A. Leigh , Science 2004, 306, 1532–1537; 15567858 10.1126/science.1103949

[4b] M. J. Barrell , A. G. Campana , M. von Delius , E. M. Geertsema , D. A. Leigh , Angew. Chem. Int. Ed. 2011, 50, 285–290; 10.1002/anie.201004779 20954231

[4c] M. R. Wilson , J. Sola , A. Carlone , S. M. Goldup , N. Lebrasseur , D. A. Leigh , Nature 2016, 534, 235–240; 27279219 10.1038/nature18013

[4d] S. Erbas-Cakmak , S. D. P. Fielden , U. Karaca , D. A. Leigh , C. T. McTernan , D. J. Tetlow , M. R. Wilson , Science 2017, 358, 340–343. 29051374 10.1126/science.aao1377

[5a] S. P. Fletcher , F. Dumur , M. M. Pollard , B. L. Feringa , Science 2005, 310, 80–82; 16210531 10.1126/science.1117090

[5b] B. S. L. Collins , J. C. M. Kistemaker , E. Otten , B. L. Feringa , Nat. Chem. 2016, 8, 860–866.

[6a] C. Cheng , P. R. McGonigal , S. T. Schneebeli , H. Li , N. A. Vermeulen , C. Ke , J. F. Stoddart , Nat. Nanotechnol. 2015, 10, 547–553; 25984834 10.1038/nnano.2015.96

[6b] Y. Qiu , L. Zhang , C. Pezzato , Y. Feng , W. Li , M. T. Nguyen , C. Cheng , D. Shen , Q. H. Guo , Y. Shi , K. Cai , F. M. Alsubaie , R. D. Astumian , J. F. Stoddart , J. Am. Chem. Soc. 2019, 141, 17472–17476. 31622089 10.1021/jacs.9b08927

[7a] L. Greb , J. M. Lehn , J. Am. Chem. Soc. 2014, 136, 13114–13117; 25211628 10.1021/ja506034n

[7b] L. Greb , A. Eichhofer , J. M. Lehn , Angew. Chem. Int. Ed. 2015, 54, 14345–14348; 10.1002/anie.201506691 26449964

[7c] I. Schapiro , M. Gueye , M. Paolino , S. Fusi , G. Marchand , S. Haacke , M. E. Martin , M. Huntress , V. P. Vysotskiy , V. Veryazov , J. Leonard , M. Olivucci , Photochem. Photobiol. Sci. 2019, 18, 2259–2269; 31347633 10.1039/c9pp00223e

[7d] M. Paolino , T. Giovannini , M. Manathunga , L. Latterini , G. Zampini , R. Pierron , J. Leonard , S. Fusi , G. Giorgi , G. Giuliani , A. Cappelli , C. Cappelli , M. Olivucci , J. Phys. Chem. Lett. 2021, 12, 3875–3884. 33856801 10.1021/acs.jpclett.1c00526

[8] G. Haberhauer , Angew. Chem. Int. Ed. 2011, 50, 6415–6418; 10.1002/anie.201101501 21630400

[9a] M. Guentner , M. Schildhauer , S. Thumser , P. Mayer , D. Stephenson , P. J. Mayer , H. Dube , Nat. Commun. 2015, 6, 8406; 26411883 10.1038/ncomms9406 PMC4598625

[9b] L. A. Huber , K. Hoffmann , S. Thumser , N. Böcher , P. Mayer , H. Dube , Angew. Chem. Int. Ed. 2017, 56, 14536–14539; 10.1002/anie.201708178 28892243

[9c] R. Wilcken , M. Schildhauer , F. Rott , L. A. Huber , M. Guentner , S. Thumser , K. Hoffmann , S. Oesterling , R. de Vivie-Riedle , E. Riedle , H. Dube , J. Am. Chem. Soc. 2018, 140, 5311–5318; 29578704 10.1021/jacs.8b02349

[9d] K. Hoffmann , P. Mayer , H. Dube , Org. Biomol. Chem. 2019, 17, 1979–1983; 30377683 10.1039/c8ob02424c

[9e] M. Schildhauer , F. Rott , S. Thumser , P. Mayer , R. de Vivie-Riedle , H. Dube , ChemPhotoChem 2019, 3, 365–371;

[9f] R. Wilcken , L. Huber , K. Grill , M. Guentner , M. Schildhauer , S. Thumser , E. Riedle , H. Dube , Chem. Eur. J. 2020, 26, 13507–13512; 32692896 10.1002/chem.202003096 PMC7702134

[9g] L. A. Huber , S. Thumser , K. Grill , D. Vossiek , N. N. Bach , P. Mayer , H. Dube , Chem. Eur. J. 2021, 27, 10758–10765. 33945652 10.1002/chem.202100950 PMC8361725

[10] A. Gerwien , P. Mayer , H. Dube , J. Am. Chem. Soc. 2018, 140, 16442–16445. 30449088 10.1021/jacs.8b10660

[11] A. Gerwien , P. Mayer , H. Dube , Nat. Commun. 2019, 10, 4449. 31575868 10.1038/s41467-019-12463-4 PMC6773862

[12a] R. Eelkema , M. M. Pollard , J. Vicario , N. Katsonis , B. S. Ramon , C. W. Bastiaansen , D. J. Broer , B. L. Feringa , Nature 2006, 440, 163; 16525460 10.1038/440163a

[12b] T. Orlova , F. Lancia , C. Loussert , S. Iamsaard , N. Katsonis , E. Brasselet , Nat. Nanotechnol. 2018, 13, 304–308. 29434262 10.1038/s41565-017-0059-x

[13] T. van Leeuwen , G. H. Heideman , D. Zhao , S. J. Wezenberg , B. L. Feringa , Chem. Commun. 2017, 53, 6393–6396. 10.1039/c7cc03188b 28524188

[14a] J. Chen , F. K. Leung , M. C. A. Stuart , T. Kajitani , T. Fukushima , E. van der Giessen , B. L. Feringa , Nat. Chem. 2018, 10, 132–138; 29359757 10.1038/nchem.2887

[14b] Q. Li , G. Fuks , E. Moulin , M. Maaloum , M. Rawiso , I. Kulic , J. T. Foy , N. Giuseppone , Nat. Nanotechnol. 2015, 10, 161–165; 25599191 10.1038/nnano.2014.315

[14c] J. T. Foy , Q. Li , A. Goujon , J.-R. Colard-Itte , G. Fuks , E. Moulin , O. Schiffmann , D. Dattler , D. P. Funeriu , N. Giuseppone , Nat. Nanotechnol. 2017, 12, 540–545. 28319615 10.1038/nnano.2017.28

[15a] S. J. Wezenberg , B. L. Feringa , Nat. Commun. 2018, 9, 1984; 29777101 10.1038/s41467-018-04249-x PMC5959844

[15b] K. Grill , H. Dube , J. Am. Chem. Soc. 2020, 142, 19300–19307. 33112151 10.1021/jacs.0c09519

[16] W. Danowski , T. van Leeuwen , S. Abdolahzadeh , D. Roke , W. R. Browne , S. J. Wezenberg , B. L. Feringa , Nat. Nanotechnol. 2019, 14, 488–494. 30886378 10.1038/s41565-019-0401-6

[17a] V. García-López , F. Chen , L. G. Nilewski , G. Duret , A. Aliyan , A. B. Kolomeisky , J. T. Robinson , G. Wang , R. Pal , J. M. Tour , Nature 2017, 548, 567–572; 28858304 10.1038/nature23657

[17b] R. S. Gunasekera , T. Galbadage , C. Ayala-Orozco , D. Liu , V. Garcia-Lopez , B. E. Troutman , J. J. Tour , R. Pal , S. Krishnan , J. D. Cirillo , J. M. Tour , ACS Appl. Mater. Interfaces 2020, 12, 13657–13670. 32091877 10.1021/acsami.9b22595 PMC8189693

[18a] J. Kaleta , J. Chen , G. Bastien , M. Dracinsky , M. Masat , C. T. Rogers , B. L. Feringa , J. Michl , J. Am. Chem. Soc. 2017, 139, 10486–10498; 28654753 10.1021/jacs.7b05404

[18b] J. Vachon , G. T. Carroll , M. M. Pollard , E. M. Mes , A. M. Brouwer , B. L. Feringa , Photochem. Photobiol. Sci. 2014, 13, 241–246; 24096390 10.1039/c3pp50208b

[18c] G. London , G. T. Carroll , T. Fernández Landaluce , M. M. Pollard , P. Rudolf , B. L. Feringa , Chem. Commun. 2009, 1712–1714; 10.1039/b821755f 19294271

[18d] B. Krajnik , J. Chen , M. A. Watson , S. L. Cockroft , B. L. Feringa , J. Hofkens , J. Am. Chem. Soc. 2017, 139, 7156–7159. 28497688 10.1021/jacs.7b02758

[19] M. M. Pollard , M. K. J. ter Wiel , R. A. van Delden , J. Vicario , N. Koumura , C. R. van den Brom , A. Meetsma , B. L. Feringa , Chem. Eur. J. 2008, 14, 11610–11622. 19009571 10.1002/chem.200800814

[20] P. Štacko , J. C. M. Kistemaker , T. van Leeuwen , M.-C. Chang , E. Otten , B. L. Feringa , Science 2017, 356, 964–968. 28572394 10.1126/science.aam8808

[21] M. Kathan , S. Crespi , N. O. Thiel , D. L. Stares , D. Morsa , J. de Boer , G. Pacella , T. van den Enk , P. Kobauri , G. Portale , C. A. Schalley , B. L. Feringa , Nat. Nanotechnol. 2022, 17, 159–165. 34916655 10.1038/s41565-021-01021-z PMC8956507

[22a] E. Uhl , S. Thumser , P. Mayer , H. Dube , Angew. Chem. Int. Ed. 2018, 57, 11064–11068; 10.1002/anie.201804716 29932486

[22b] E. Uhl , P. Mayer , H. Dube , Angew. Chem. Int. Ed. 2020, 59, 5730–5737; 10.1002/anie.201913798 PMC7154650 31943681

[23] Y. Liu , Q. Zhang , S. Crespi , S. Chen , X. K. Zhang , T. Y. Xu , C. S. Ma , S. W. Zhou , Z. T. Shi , H. Tian , B. L. Feringa , D. H. Qu , Angew. Chem. Int. Ed. 2021, 60, 16129–16138; 10.1002/anie.202104285 PMC8361693 33955650

[24] D. H. Qu , B. L. Feringa , Angew. Chem. Int. Ed. 2010, 49, 1107–1110; 10.1002/anie.200906064 20039249

[25a] C. O. Dietrich-Buchecker , J. P. Sauvage , J. P. Kintzinger , Tetrahedron Lett. 1983, 24, 5095–5098;

[25b] C. O. Dietrich-Buchecker , J. P. Sauvage , Chem. Rev. 1987, 87, 795–810.

[26] J. J. Yu , L. Y. Zhao , Z. T. Shi , Q. Zhang , G. London , W. J. Liang , C. Gao , M. M. Li , X. M. Cao , H. Tian , B. L. Feringa , D. H. Qu , J. Org. Chem. 2019, 84, 5790–5802. 30971085 10.1021/acs.joc.9b00783

[27] Y. Wang , Y. Tian , Y. Z. Chen , L. Y. Niu , L. Z. Wu , C. H. Tung , Q. Z. Yang , R. Boulatov , Chem. Commun. 2018, 54, 7991–7994. 10.1039/c8cc04542a 29966026

[28] S. Wiedbrauk , H. Dube , Tetrahedron Lett. 2015, 56, 4266–4274.

