# Supplementary material for: Active Mechanical Threading by a Molecular Motor
Source: Angew Chem Int Ed Engl. 2022 Mar 4;61(19):e202201882. doi: 10.1002/anie.202201882 (PMC9314141; doi:10.1002/anie.202201882)
Supplement: Supplementary file 1 — Supporting Information [file ANIE-61-0-s001.pdf]

Angewandte Chemie
Eine Zeitschrift der Gesellschaft Deutscher Chemiker

Supporting Information

Active Mechanical Threading by a Molecular Motor

*N. N. Bach, V. Josef, H. Maid, H. Dube**

Table of Contents

Materials and General Methods 3
Synthesis of Compounds 5
Synthesis Route 5
Building Block Syntheses 6
Structural and Conformational Analysis 29
Structures in Solution 29
Isomer **C-1** 29
Isomer **A-1** 33
Metastable Isomer **D-1** 38
Isomer **C-2** 43
Isomer **A-2** 46
Isomer **D-2** 50
Comparison of ^{1}H-NMR spectra of stoppered system 1 and unstoppered system 2 53
Elevated Temperature Behavior of Macrocyclic Motors 1 and 2 54
Motor Function Elucidation of HTI 1 62
Comparison Between Ambient and Low Temperature ^{1}H NMR Spectra 62
Irradiation of Isomer A-1 63
Irradiation of Isomer C-1 64
Thermal Conversion of B-1 at Low Temperatures 64
Thermal Conversion of D-1 at Low Temperatures 67
Switching Behaviour of HTI 2 72
Photoswitching 72
Thermal Conversion of D-2 at Elevated Temperature 73
Separation of Enantiomers 76
Photophysical Properties 78
UV-Vis Absorption Spectra and Photoisomerization 78
ECD Spectra 82
Experimentally determined *g*-Factors 84
ECD Photoisomerization Experiments 86
Theoretical Description of Macrocyclic Motor 1 91
Ground State Geometries 91
Theoretically Obtained ECD Spectra 93
Comparison of calculated and experimental ECD spectra 94
Pre-threaded Structure A_i-1 97
XYZ-Data 98
Crystal Structure Data of *(E)*-5 99
NMR Spectra of Synthetic Intermediates 100
References 118

Materials and General Methods

Reagents and solvents were obtained from *abcr, Acros, Merck, Sigma-Aldrich* or *TCI* in the qualities puriss., p.a., or purum and used as received. Technical solvents were distilled on a rotary evaporator (*Heidolph Hei-VAP*) before use for column chromatography and extraction. Anhydrous solvents purchased from *Merck*, *Sigma-Aldrich* and *Acros* were used without further purification. Monitoring of reaction progress was done by thin-layer chromatography (TLC) using aluminum plates coated with SiO_2 (*Merck 60*, *F-254*). Detection was done by irradiation with UV light (254 nm or 366 nm) in order to determine retardation factors (R_f).

Flash column chromatography was performed with silica gel 60 (*Merck*, particle size 0.063-0.200 mm; or *Macherey-Nagel*, particle size 0.04-0.063 mm). Automated flash column chromatography was performed on *Biotage Isolera One* or *Biotage Selekt* machines with pre-packed silica columns from *Biotage* or *Macherey-Nagel*.

High Performance Liquid Chromatography (HPLC) was performed on a *Shimadzu* HPLC system consisting of a *LC-20AP* solvent delivery module, a *CTO-20A* column oven, a *SPD-M20A* photodiode array UV/Vis detector, and a *CBM-20A* system controller using a semi-preparative CHIRALPAK® IC or ID column (particle size 5 µm) from *Daicel* and HPLC grade solvents from *Sigma-Aldrich, Honeywell, VWR*, and *ROTH*.

^{1}H-NMR and ^{13}C-NMR spectra were measured on a *Jeol ECX-400* (400 MHz), *Varian 400 MHz*, *Bruker Avance III HD 400* (400 MHz), *Bruker Avance Neo HD 400 MHz*, *Bruker Avance Neo HDX 500 MHz*, *Bruker Avance Neo HDX 600 MHz* with *cryo probe DCH-Z^{13}C/^{1}H*, *Varian 600 NMR*, or *Bruker Avance III HD 800* (800 MHz) spectrometer. Chemical shifts (δ) are reported relative to residual solvent signals in the ^{1}H- and ^{13}C-NMR spectra, which were used as internal reference. Deuterated solvents were obtained from *Cambridge Isotope Laboratories* or *Eurisotop*, *Deutero GmbH*, and *Sigma-Aldrich*. For ^{1}H-NMR: $CDCl_3$ = 7.26 ppm, CD_2Cl_2 = 5.32 ppm, $(CDCl_2)_2$ = 6.00 ppm, $(CD_3)_2SO$ = 2.50 ppm. For ^{13}C-NMR: $CDCl_3$ = 77.16 ppm, CD_2Cl_2 = 54.00 ppm, $(CD_3)_2SO$ = 39.52 ppm. Resonance multiplicity is indicated as *s* (singlet), *d* (doublet), *t* (triplet), *q* (quartet) and *m* (multiplet). Chemical shifts are given in parts per million (ppm). Coupling constant values (*J*) are given in *Hertz* (*Hz*).

Electron Impact (EI) mass spectra were measured on a *Thermo Q Exactive GC Orbitrap* or *Finnigan MAT 95* mass spectrometer. **Electrospray ionisation (ESI) mass spectra** were measured on a *Thermo Finnigan LTQ FT Ultra Fourier Transform Ion Cyclotron Resonance* mass spectrometer, a *Bruker Daltonics maXis 4G* or a *micrOTOF II* spectrometer. Atmospheric pressure photoionization (APPI) mass spectra were recorded on a *Bruker Daltonics maXis 4G* or a *micrOTOF II* spectrometer.

Infrared spectra (ATR) were recorded on a *Perkin Elmer Spectrum BX* spectrometer equipped with a *Smiths Detection DuraSamplIR II Diamond-ATR* unit or on a *Varian 660-IR* spectrometer with a spectral resolution of 4 cm^{-1}. Transmittance values are qualitatively described by wavenumber (cm^{-1}) as strong (s), medium (m), weak (w), and very weak (vw).

UV/Vis spectra were measured on a Varian Cary 5000 spectrophotometer. The spectra were recorded in a quartz cuvette (1 cm path length). CH_2Cl_2 for spectroscopy was obtained from VWR and filtered over basic alumina (activated, Brockmann I) prior to use.

Electronic circular dichroism (ECD) spectra were measured on a *Jasco J-810* or *Jasco J-815 CD Spectrometer*. For low temperature measurements, the samples were measured in fluorescence cuvettes and were placed inside an *Oxford Optistat DN 1704* cryostat with *Oxford ITC-4* temperature controller. Liquid nitrogen was used as cryogen. A steady flow of nitrogen gas was attached in order to minimize condensation of water inside the sample chamber. The detector was removed from the spectrometer housing, so that the cryostat could be placed in the beam path. Cryostat, cuvette, and detector were aligned with the beam path and the components were affixed on an optical breadboard.

Photoisomerization experiments: Continuous irradiations of the solutions were conducted either in NMR tubes in different deuterated solvents (CD_2Cl_2, CD_2Cl_2/CS_2 (4/1, v/v), $(CDCl_2)_2$; CD_2Cl_2 and $(CDCl_2)_2$ were filtered over basic alumina (activated, Brockmann I) prior to use) or in quartz cuvettes (1 cm) in protonated solvents. Irradiations were conducted using a Prizmatix or Mightex UHP LED (405 nm, 450 nm) coupled to a 1500 µm quartz fiber for *in situ* irradiation NMR-experiments or using LEDs (Roithner/Thorlabs) for external irradiation experiments.

Melting points (mp) were measured on a *Stuart SMP10* or *Büchi M-560* melting point apparatus in open capillaries and are not corrected.

Synthesis of Compounds

Synthesis Route

Macrocyclic motors **1** and **2** can be synthesized from simple building blocks in a modular fashion as shown in **Scheme 1**.

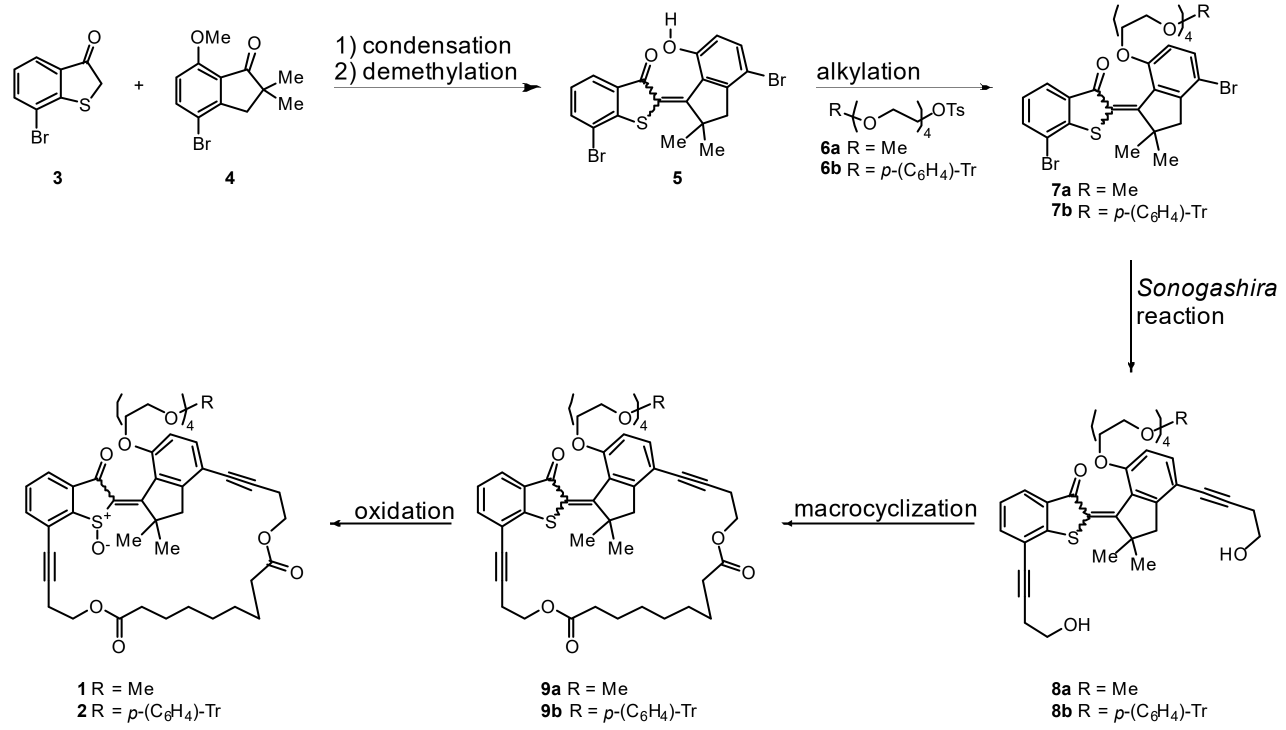

Scheme 1 General scheme for the synthesis of macrocyclic motors **1** and **2**.

Building blocks **3**, **6a** and **6b** were synthesized following adapted literature procedures (see **Scheme 2**).

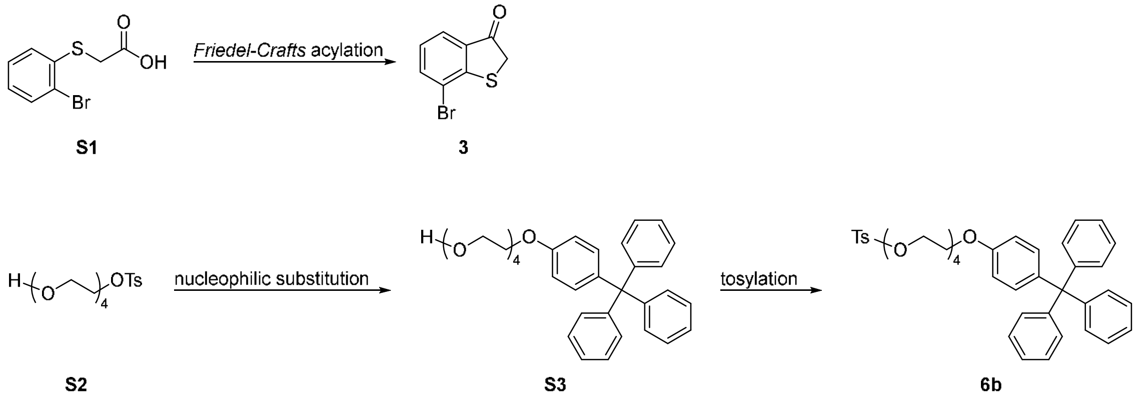

Scheme 2 Synthesis of **3** and **6b** starting from precursors **S1** and **S2**.

Building Block Syntheses

2-((2-bromophenyl)thio)acetic acid (S1)[1]

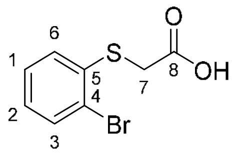

To a solution of 2-bromo acetic acid (2.5 g, 18.0 mmol, 1.1 equiv.) in acetone (170 mL), potassium carbonate (7.0 g, 50.6 mmol, 3.0 equiv.) and 2-bromo thiophenol (2.0 mL, 16.6 mmol, 1.0 equiv.) were added at 0 °C. The suspension was allowed to warm to 25 C overnight and was stirred for a total of 116 h (after 27 h additional 100 mL of acetone were added). The reaction was stopped by the addition of aq. HCl (2 M, 200 mL). Acetone was removed *in vacuo* and the resulting precipitate was separated by filtration, washed with H_2O and dried under high vacuum. Compound **S1** (3.6 g, 14.6 mmol, 88%) was obtained as colorless microcrystalline solid, which was used without further purification.

^{1}H NMR (400 MHz, $(CD_3)_2SO$): δ / ppm = 12.93 (s, 1H, CO_2H), 7.60 (dd, J = 8.0, 1.3 Hz, 1H, H-C(3)), 7.37 (ddd, J = 8.5, 7.3, 1.4 Hz, 1H, H-C(1)), 7.30 (dd, J = 8.0, 1.6 Hz, 1H, H-C(6)), 7.10 (ddd, J = 7.9, 7.2, 1.6 Hz, 1H, H-C(2)), 3.90 (s, 2H, H_2-C(7)).

^{13}C NMR (101 MHz, $(CD_3)_2SO$): δ / ppm = 170.1 (C(8)), 137.2 (C(5)), 132.6 (C(3)), 128.3 (C(1)), 126.7 (C(2/6)), 126.6 (C(2/6)), 120.8 (C(4)), 34.2 (C(7)).

HRMS (EI) calcd. for $[C_8H_7BrO_2S]^+$: 245.9350; found: 245.9344.

Tetraethylene glycol monotosylate (S2)[2]

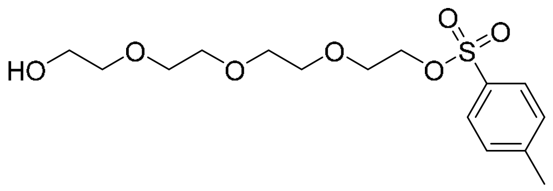

Tetraethylene glycol (37.1 g, 191.0 mmol, 10.4 equiv.) was dissolved in THF (7 mL). Aq. NaOH (1.4 g NaOH in 7 mL of water, 35.0 mmol, 1.9 equiv.) was added at 0 °C, followed by the slow addition of *p*-TsCl (3.5 g in 20 mL THF, 18.4 mmol, 1.0 equiv.) over the course of 30 min. The reaction mixture was stirred at 0 °C for 6.5 h, then it was poured into ice water (250 mL). The mixture was extracted with CH_2Cl_2 (2 × 200 mL) and the combined organic phases were washed with H_2O (4 × 200 mL), dried over sodium sulphate, filtered, and concentrated *in vacuo* to give compound **S2** (5.1 g, 14.6 mmol, 79%) as a faintly yellow oil, which was used without further purification.

^{1}H NMR (400 MHz, $CDCl_3$): δ / ppm = 7.79–7.74 (m, 2H), 7.34–7.29 (m, 2H), 4.15–4.11 (m, 2H), 3.69–3.64 (m, 4H), 3.61 (dt, J = 5.3, 2.4 Hz, 4H), 3.57 (d, J = 4.1 Hz, 6H), 2.42 (s, 3H).

^{13}C NMR (101 MHz, $CDCl_3$): δ / ppm = 144.9, 133.0, 129.9, 128.0, 72.5, 70.8, 70.7, 70.5, 70.4, 69.3, 68.7, 61.7, 21.7.

HRMS (ESI$^+$) calcd. for $[C_{15}H_{24}O_7S+H]^+$ 349.1316; found: 349.1318.

IR: $\tilde{\upsilon}$ / cm^{-1} = 2870 (w), 1597 (w), 1452 (w), 1350 (s), 1306 (w), 1292 (w), 1248 (w), 1188 (m), 1174 (vs), 1120 (s), 1095 (s), 1066 (m), 1011 (m), 916 (s), 816 (s), 773 (s), 706 (w), 690 (w), 661 (s), 634 (w), 582 (m), 552 (vs), 501 (m), 467 (m), 434 (m), 418 (w), 409 (w).

Compound S3[3]

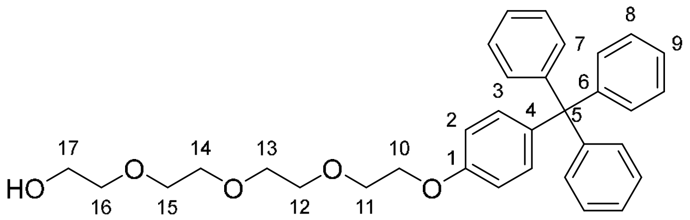

To a solution of compound **S2** (4.1 g, 11.8 mmol, 2.0 equiv.) in acetonitrile (100 mL), 4-tritylphenol (2.0 g, 5.9 mmol, 1.0 equiv.) and potassium carbonate (1.0 g, 7.1 mmol, 1.2 equiv.) were added and the mixture was refluxed for 6 h, then stirring was continued at 60 °C for an additional 18 h. The solvent was removed *in vacuo* and the residue was distributed between H_2O (100 mL) and chloroform (100 mL). The organic phase was separated, washed with brine (100 mL), dried over sodium sulphate, filtered, and concentrated *in vacuo*. The crude product was purified by flash column chromatography (SiO_2, CH_2Cl_2/EtOAc, 50/50) to give compound **S3** (2.1 g, 4.1 mmol, 69%) as a white solid.

R_f (CH_2Cl_2/EtOAC, 50/50) = 0.16.

^{1}H NMR (400 MHz, CD_2Cl_2): δ / ppm = 7.29–7.17 (m, 15H, H-C(7+8+9)), 7.16–7.11 (m, 2H, H-C(3)), 6.84–6.79 (m, 2H, H-C(2)), 4.12–4.08 (m, 2H, H-C(10)), 3.83–3.79 (m, 2H, H-C(11)), 3.70–3.61* (m, 10H), 3.58–3.55* (m, 2H).

*TEG-chain H_2-C(12+13+14+15+16+17).

^{13}C NMR (101 MHz, CD_2Cl_2): δ / ppm = 157.2 (C(1)), 147.6 (C(6)), 139.6 (C(4)), 132.5 (C(3)), 131.4 (C(7/8)), 127.9 (C(7/8)), 126.2 (C(9)), 113.7 (C(2)), 72.8*, 71.1*, 71.0*, 70.9*, 70.7*, 70.1 (C(11)), 67.8 (C(10)), 64.7 (C(5)), 62.0*.

*TEG-chain C(12/13/14/15/16/17).

HRMS (ESI$^+$) calcd. for $[C_{33}H_{36}O_5+Na]^+$: 535.2455; found: 535.2456.

mp: 89.7-90.3 °C.

7-bromobenzo[*b*]thiophen-3(2*H*)-one (3)[1]

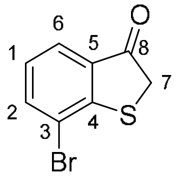

Compound **S1** (3.6 g, 14.6 mmol, 1.0 equiv.) was refluxed in thionyl chloride (9.0 mL, 123.4 mmol, 8.5 equiv.) for 2.5 h. Excess thionyl chloride was removed *in vacuo* and the residue was dissolved in $(CH_2Cl)_2$ (60 mL). At 0 °C, $AlCl_3$ (3.7 g, 27.7 mmol, 1.9 equiv.) was added and the mixture was stirred under nitrogen atmosphere at 0 °C for 2 h. The reaction mixture was poured into ice water (300 mL) and the mixture was extracted with CH_2Cl_2 (2 × 200 mL). The combined organic phases were dried over sodium sulphate, filtered, and concentrated *in vacuo* to give compound **3** (3.2 g, 14.0 mmol,96%) as a pink solid, which was used without further purification.

^{1}H NMR (400 MHz, $CDCl_3$): δ / ppm = 7.74 (dd, J = 6.1, 1.2 Hz, 1H, H-C(6)), 7.72 (dd, J = 6.1, 1.0 Hz, 1H, H-C(2)), 7.13 (t, J = 7.7 Hz, 1H, H-C(1)), 3.84 (s, 2H, H_2-C(7)).

^{13}C NMR (101 MHz, $CDCl_3$): δ / ppm = 199.6 (C(8)), 155.5 (C(4)), 138.2 (C(2)), 133.1 (C(5)), 126.3 (C(1)), 125.4 (C(6)), 118.9 (C(3)), 40.3 (C(7)).

HRMS (EI) calcd. for $[C_8H_5BrOS]^+$: 227.9245; found: 227.9238.

4-bromo-7-methoxy-2,2-dimethyl-2,3-dihydro-1*H*-inden-1-one (4)

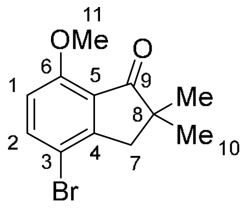

In a flame-dried Schlenk flask, NaH (60% in mineral oil, 1.0 g, 24.9 mmol, 3.0 equiv.) was dispersed in dry THF (60 mL) under nitrogen atmosphere. At 0 °C, 4-bromo-7-methoxy-1-indanone (2.0 g, 8.3 mmol, 1.0 equiv.) was added and the reaction mixture was stirred at 0 °C for 30 min. Iodomethane (1.3 mL, 20.8 mmol, 2.5 equiv.) was added slowly and stirring was continued at 0 °C for 2.5 h, then the mixture was allowed to warm to 23 °C and subsequently stirred at that temperature for 18.5 h. The reaction was stopped by the addition of H_2O (100 mL) and the mixture was extracted with EtOAc (3 × 100 mL). The combined organic phases were dried over sodium sulphate, filtered, and concentrated *in vacuo*. The crude product was purified by flash column chromatography (SiO_2, EtOAc/*i*-Hex, 30/70) to give compound **4** (1.5 g, 5.6 mmol, 67%) as a white solid.

R_f (EtOAc/*i*-Hex, 20/80) = 0.37.

^{1}H NMR (599 MHz, $CDCl_3$): δ / ppm = 7.64 (d, J = 8.7 Hz, 1H, H-C(2)), 6.72 (d, J = 8.7 Hz, 1H, H-C(1)), 3.92 (s, 3H, H-C(11)), 2.86 (s, 2H, H_2-C(7)), 1.22 (s, 6H, H_3-C(10)).

^{13}C NMR (151 MHz, $CDCl_3$): δ / ppm = 208.6 (C(9)), 157.9 (C(6)), 153.9 (C(4)), 138.8 (C(2)), 125.2 (C(5)), 112.0 (C(3)), 111.4 (C(1)), 56.2 (C(11)), 45.7 (C(8)), 43.8 (C(7)), 25.5 (C(10)).

HRMS (EI) calcd. for $[C_{12}H_{13}BrO_2]^+$: 268.0099; found: 268.0092.

mp: 112.3-114.5 °C.

HTI 5

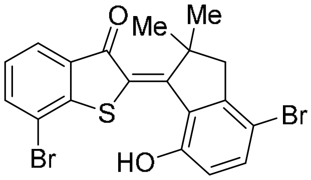

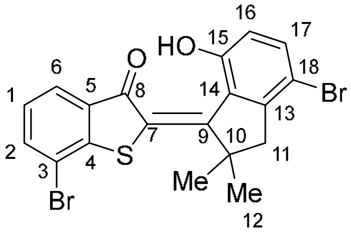

In a flame-dried Schlenk-flask a solution of compound **3** (276 mg, 1.2 mmol, 1.1 equiv.) in dry CH_2Cl_2 (3 mL) was cooled to −78 °C under nitrogen atmosphere. Subsequently, BCl_3 (1 M in CH_2Cl_2, 1.2 mL, 1.2 mmol, 1.1 equiv.) was added. The mixture was immediately taken up by syringe (an additional 2 mL of dry CH_2Cl_2 were added to rinse the flask) and added to a second Schlenk-flask containing a solution of compound **4** (301 mg, 1.1 mmol, 1.0 equiv.) in dry CH_2Cl_2 (1.5 mL) at 0 °C. The reaction mixture was stirred at 0 °C for 45 min. BBr_3 (1 M in CH_2Cl_2, 1.1 mL, 1.1 mmol, 1.0 equiv.) was added and stirring was continued at 0 °C for 45 min. The reaction was stopped by the addition of H_2O (5 mL). The mixture was diluted with H_2O (50 mL) and extracted with EtOAc (3 × 50 mL). The combined organic phases were dried over sodium sulphate, filtered, and concentrated *in vacuo*. The crude product was purified by flash column chromatography (SiO_2, EtOAc/*i*-Hex, 10/90) to give compound **5** (mixture of (*E*)- and (*Z*)-isomers, 411 mg, 0.9 mmol, 82%) as a red foam.

Single crystals of the (*E*)-isomer for structure determination by X-ray diffraction could be obtained by recrystallization from *n*-heptane.

R_f (EtOAc/*i*-Hex, 10/90) = 0.63 and 0.69 (heat is necessary to colorize the second spot).

HRMS (EI) calcd. for $[C_{19}H_{14}{}^{79}Br_2O_2S]^+$: 463.9081; found: 463.9079.

(*E*)-5

^{1}H NMR (400 MHz, CD_2Cl_2): δ / ppm = 8.01 (dd, *J* = 7.8, 1.1 Hz, 1H, H-C(6)), 7.86 (s, 1H, OH), 7.83 (dd, *J* = 7.8, 1.1 Hz, 1H, H-C(2)), 7.62 (d, *J* = 8.8 Hz, 1H, H-C(17)), 7.29 (t, *J* = 7.7 Hz, 1H, H-C(1)), 6.96 (dt, *J* = 8.8, 0.8 Hz, 1H, H-C(16)), 3.11 (s, 2H, H_2-C(11)), 1.64 (s, 6H, H_3-C(12)).

^{13}C NMR (101 MHz, CD_2Cl_2): δ / ppm = 191.1 (C-8), 167.1 (C-9), 160.2 (C-15), 150.5 (C-13), 146.5 (C-4), 138.1 (C-2), 137.6 (C-17), 134.1 (C-5), 131.0 (C-14), 127.0 (C-7), 126.9 (C-1), 126.4 (C-6), 122.2 (C-16), 117.1 (C-3), 110.9 (C-18), 52.3 (C-10), 51.5 (C-11), 27.7 (C-12).

IR: $\tilde{\nu}$ / cm^{-1} = 3059 (w), 2956 (w), 2924 (m), 1577 (m), 1560 (m), 1498 (s), 1493 (s), 1464 (s), 1454 (s), 1431 (m), 1423 (m), 1408 (s), 1387 (m), 1369 (m), 1361 (m), 1263 (s), 1215 (m), 1176 (m), 1159 (s), 1132 (m), 1103 (s), 1057 (s), 1039 (s), 1009 (m), 883 (m), 872 (m), 816 (m), 800 (vs), 775 (s), 758 (s),

741 (vs), 735 (vs), 702 (s), 683 (m), 660 (s), 648 (m), 561 (s), 540 (m), 521 (m), 496 (m), 474 (m), 461 (m), 449 (s).

mp: 148.0-150.0 °C.

Tosylate 6a[4]

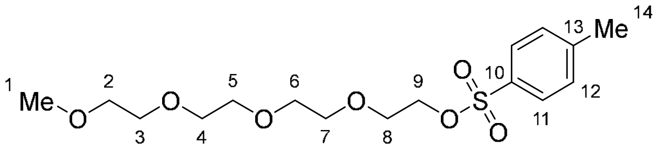

To a solution of tetraethyleneglycol monomethyl ether (3.0 g, 14.4 mmol, 1.0 equiv.) in THF (30 mL) a solution of NaOH (0.85 g in 5 mL of H_2O, 21.6 mmol, 1.5 equiv.) was added at 0 °C. Then, a solution of *p*-TsCl (3.0 g in 10 mL THF, 15.8 mmol, 1.1 equiv.) was added dropwise at 0 °C over the course of 30 min. After addition, stirring was continued at 0 °C for 2 h. The reaction mixture was poured into ice-water (200 mL) and extracted with CH_2Cl_2 (3 × 100 mL). The combined organic phases were washed with H_2O (2 × 200 mL) and brine (100 mL), dried over sodium sulphate, filtered, and concentrated *in vacuo* to give compound **S4** (3.68 g, 10.2 mmol, 71%) as a faint yellow oil.

^{1}H NMR (400 MHz, $CDCl_3$): δ / ppm = 7.80–7.75 (m, 2H, H-C(11)), 7.32 (d, J = 7.9 Hz, 2H, H-C(12)), 4.16–4.11 (m, 2H, H_2-C(9)), 3.68–3.65 (m, 2H, H_2-C(8)), 3.63–3.60 (m, 6H, H_2-C(3) and either H_2-C(4+5) or H_2-C(6+7)), 3.56 (s, 4H, either H_2-C(4+5) or H_2-C(6+7)), 3.52 (dd, J = 6.0, 3.0 Hz, 2H, H_2-C(2)), 3.35 (s, 3H, H_3-C(1)), 2.43 (s, 3H, H_3-C(14)).

^{13}C NMR (101 MHz, $CDCl_3$): δ / ppm = 144.9 (C(13)), 133.1 (C(10)), 129.9 (C(12)), 128.0 (C(11)), 72.0 (C(2)), 70.8*, 70.7*, 70.7*, 70.6*, 70.6*, 69.3 (C(9)), 68.7 (C(8)), 59.1 (C(1)), 21.7 (C(14)).

*(C(3/4/5/6/7)).

HRMS (ESI$^+$) calcd. for $[C_{16}H_{26}O_7S+K]^+$: 401.1031; found: 401.1036.

IR: $\tilde{\nu}$ / cm^{-1} = 2871 (w), 1597 (w), 1495 (vw), 1450 (w), 1398 (w), 1352 (s), 1306 (w), 1292 (w), 1248 (w), 1188 (m), 1174 (vs), 1095 (s), 1016 (m), 916 (s), 849 (m), 816 (s), 773 (s), 706 (w), 690 (w), 661 (s), 634 (w), 575 (m), 553 (vs), 530 (m), 501 (m), 467 (w), 428 (w), 422 (w), 413 (w), 405 (w).

Compound 6b[3]

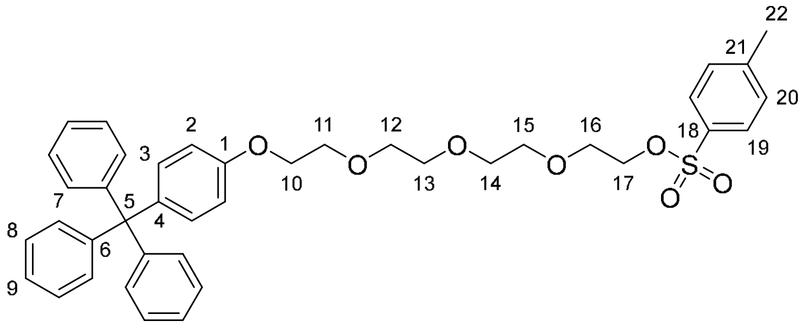

Compound **S3** (978 mg, 1.9 mmol, 1.0 equiv.), NEt_3 (1.3 mL, 9.5 mmol, 5.0 equiv.) and 4-dimethylaminopyridine (12 mg, 5 mol-%) were dissolved in CH_2Cl_2 (20 mL) and cooled to 0 °C. A solution of *p*-TsCl (915 mg in 7.0 mL CH_2Cl_2, 4.8 mmol, 2.5 equiv.) was slowly added at 0 °C. The reaction mixture was stirred for 14 h while slowly warming to 23 °C. The reaction was stopped by the addition of aq. HCl (2 M, 70 mL). The aqueous layer was separated and extracted with CH_2Cl_2 (100 mL). The combined organic phases were washed with H_2O (100 mL) and brine (100 mL), dried over sodium sulphate, filtered, and concentrated *in vacuo*. The residue was purified by flash column chromatography (SiO_2, EtOAc/CH_2Cl_2, 10/90) to yield compound **6b** (1.1 g, 1.6 mmol, 84%) as a colorless viscous oil.

R_f (EtOAc/CH_2Cl_2, 10/90) = 0.76.

^{1}H NMR (400 MHz, CD_2Cl_2): δ / ppm = 7.78 (d, J = 8.3 Hz, 2H, H-C(19)), 7.36 (d, J = 8.1 Hz, 2H, H-C(20)), 7.30–7.16 (m, 15H, H-C(7+8+9)), 7.13 (d, J = 8.8 Hz, 2H, H-C(3)), 6.80 (d, J = 8.9 Hz, 2H, H-C(2)), 4.14–4.10 (m, 2H, H_2-C(17)), 4.10–4.06 (m, 2H, H_2-C(10)), 3.82–3.76 (m, 2H, H_2-C(11)), 3.68–3.62 (m, 4H, H_2-C(16+12)), 3.62–3.57 (m, 2H, H_2-C(13)), 3.55 (s, 4H, H_2-C(14+15)), 2.43 (s, 3H, H_3-C(22)).

^{13}C NMR (101 MHz, CD_2Cl_2): δ / ppm = 157.4 (C(1)), 147.7 (C(6)), 145.6 (C(21)), 139.7 (C(4)), 133.5 (C(18)), 132.6 (C(3)), 131.5 (C(7/8)), 130.4 (C(20)), 128.4 (C(19)), 128.0 (C(7/8)), 126.4 (C(9)), 113.9 (C(2)), 71.3*, 71.2*, 71.1*, 71.0*, 70.2*, 70.1*, 69.1*, 68.0 (C(10)), 64.9 (C(5)), 21.9 (C(22)).

*TEG chain C(11/12/13/14/15/16/17)

HRMS (EI) calcd. for $[C_{40}H_{42}O_7S]^+$: 666.2651; found: 666.2638.

HTI (*E*/*Z*)-7a

Compound **5** (mixture of (*E*)- and (*Z*)-isomers, 220 mg, 0.47 mmol, 1.0 equiv.), sodium iodide (80 mg, 0.53 mmol, 1.1 equiv.) and potassium carbonate (196 mg, 1.41 mmol, 3.0 equiv.) were added to a two-neck round bottom flask equipped with a magnetic stirring bar, a septum and a reflux condenser. The flask was evacuated and refilled with dry nitrogen once. Dry butanone (5 mL) was added, followed by the addition of compound **6a** (206 mg, 0.57 mmol, 1.2 eq). The reaction mixture was refluxed under nitrogen for a total of 4 h (after a period of 2.75 h additional 3 mL of butanone were added). The reaction mixture was poured into H_2O (100 mL) and extracted with EtOAc (2 × 100 mL). The combined organic phases were washed with H_2O (200 mL, a small amount of 10% aq. LiCl was added for better phase separation) and brine (2 × 200 mL), dried over sodium sulphate, filtered, and concentrated *in vacuo*. The residue was purified by flash column chromatography (SiO_2, EtOAc/*i*-Hex, 60/40). Isomer (*Z*)-**7a** (140 mg of an inseparable mixture with 37% TEG-iodide as judged by ^{1}H NMR spectroscopy, 0.17 mmol, 36%) and isomer (*E*)-**7a** (84 mg of an inseparable mixture with 34% tosylate **6a** as judged by ^{1}H NMR spectroscopy, 0.10 mmol, 21%) were obtained orange oils. The obtained compounds were used in the next synthetic step without further purification.

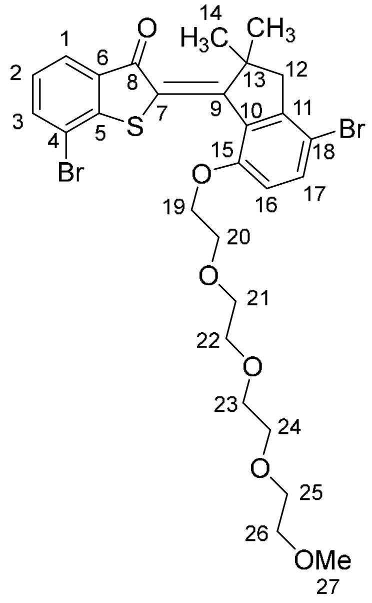

(*Z*)-**7a**

R_f (EtOAc/*i*-Hex, 50/50) = 0.22.

^{1}H NMR (400 MHz, CD_2Cl_2): δ / ppm = 7.73 (dd, *J* = 7.6, 1.1 Hz, 1H, H-C(1)), 7.68 (dd, *J* = 7.7, 1.1 Hz, 1H, H-C(3)), 7.54 (d, *J* = 8.8 Hz, 1H, H-C(17)), 7.15 (t, *J* = 7.7 Hz, 1H, H-C(2)), 6.84 (d, *J* = 8.8 Hz, 1H, H-C(16)), 4.36–4.31 (m, 2H, H-C(19)), 4.02–3.97 (m, 2H, H-C(20)), 3.73 (t, *J* = 6.7 Hz, 1H, H-C(26)), 3.65–3.56 (m,

4H, H-C(21, 24)), 3.55–3.44 (m, 4H, H-C(22, 23)), 3.31 (s, 3H, H-C(27)), 2.97 (s, 2H, H-C(12)), 1.54 (s, 6H, H-C(14)).

^{13}C NMR (101 MHz, CD_2Cl_2): δ / ppm = 188.7 (C(8)), 163.2 (C(9)), 155.5 (C(15)), 150.0 (C(11)), 147.3 (C(5)), 137.1 (C(3)), 136.1 (C(17)), 134.4 (C(6)), 129.8 (C(10)), 129.5 (C(7)), 126.3 (C(2)), 124.8 (C(1)), 117.0 (C(4)), 113.3 (C(16)), 111.1 (C(18)), 72.3 (C(25)), 72.3 (C(26)), 71.0 (C(22)), 70.9 (C(23)), 70.8 (C(21)), 70.6 (C(24)), 69.5 (C(20)), 68.8 (C(19)), 59.0 (C(27)), 52.7 (C(12)), 51.2 (C(13)), 26.5 (C(14)).

HRMS (EI) calcd. for $[C_{28}H_{32}{}^{79}Br_2O_6S]^+$: 654.0286; found 654.0282.

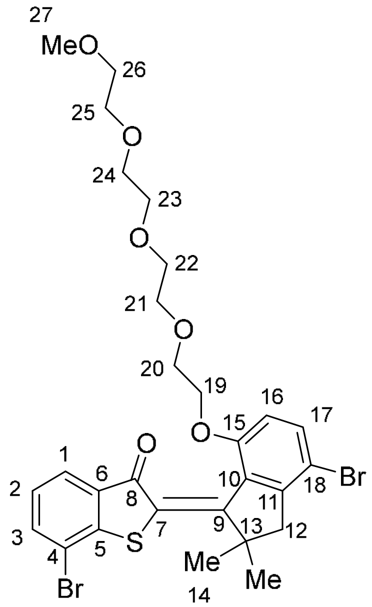

(*E*)-**7a**

R_f (EtOAc/*i*-Hex, 50/50) = 0.07.

^{1}H NMR (400 MHz, CD_2Cl_2): δ / ppm = 7.81 (dd, J = 7.6, 1.1 Hz, 1H, H-C(1)), 7.73 (dd, J = 7.8, 1.1 Hz, 1H, H-C(3)), 7.47 (d, J = 8.8 Hz, 1H, H-C(17)), 7.22 (t, J = 7.7 Hz, 1H, H-C(2)), 6.74 (d, J = 8.9 Hz, 1H, H-C(16)), 4.22–4.10 (m, 4H, H-C(19, 20)), 3.71–3.63 (m, 2H, H-C(21)), 3.62–3.42 (m, 10H, H-C(22, 23, 24, 25, 26)), 3.31 (s, 3H, H-C(27)), 3.13 (d, J = 16.0 Hz, 1H, H-C(12)), 2.86 (d, J = 16.0 Hz, 1H, H-C(12)), 1.74 (s, 3H, H-C(14)), 1.28 (s, 3H, H-C(14)).

^{13}C NMR (101 MHz, CD_2Cl_2): δ / ppm = 186.6 (C(8)), 171.2 (C(9)), 157.7 (C(15)), 148.8 (C(11)), 145.5 (C(5)), 136.8 (C(3)), 134.7 (C(17)), 134.4 (C(6)), 130.3(C(7)), 130.0 (C(10)), 126.7 (C(2)), 125.0 (C(1)), 117.8 (C(4)), 112.5 (C(16)), 110.3 (C(18)), 72.3 (C(26)), 72.3(C(25)), 70.8 (C(22)), 70.6 (C(23)), 69.9 (C(21)), 69.8 (C(24)), 69.0 (C(20)), 68.2 (C(19)), 59.0 (C(27)), 50.8 (C(12)), 26.6 (C(14)), 26.5 (C(14)), 26.3 (C(13)).

HRMS (EI): calcd. for $[C_{28}H_{32}{}^{79}Br_2O_6S]^+$: 654.0286; found 654.0272.

HTI (*E*/*Z*)-7b

Compound **6b** (716 mg, 1.1 mmol, 0.9 equiv.) was dissolved in DMF (13 mL). Compound **5** (91-4)(559 mg, 1.2 mmol, 1.0 equiv.), K_2CO_3 (739 mg, 5.3 mmol, 4.4 equiv.) and NaI (44 mg, 0.3 mmol, 25 mol-%) were added and the reaction mixture was stirred at 90 °C for 21 h. The reaction mixture was poured into H_2O (150 mL) and extracted with EtOAc (3 × 100 mL, small amounts of brine were added for better phase separation). The combined organic phases were washed with H_2O (1 × 200 mL, 1 × 300 mL) and brine (4 × 200 mL), dried over sodium sulphate, filtered, and concentrated *in vacuo*. The crude product was purified by flash column chromatography (SiO_2, EtOAc/*i*-Hex, 40/60) to give isomer (*Z*)-**7b** (156 mg, 0.2 mmol, 18%) as an orange oil and isomer (*E*)-**7b** (403 mg, 0.4 mmol, 36%) as an orange foam. Note: 189 mg of starting material **5** were recovered (total yield brsm: 75%).

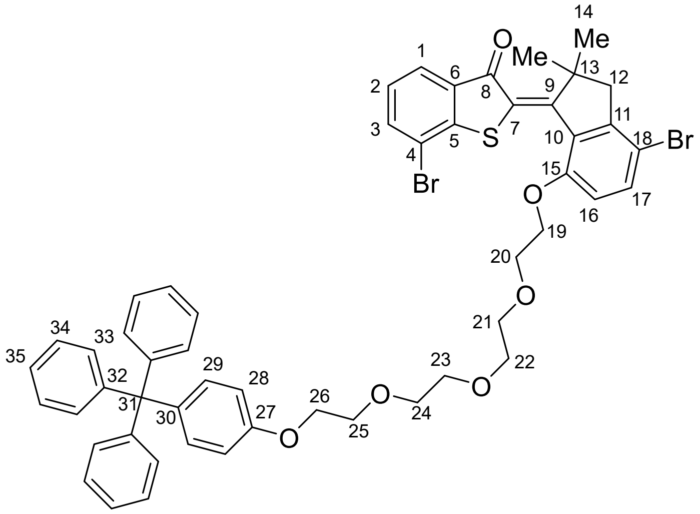

(*Z*)-**7b**

R_f (EtOAc/*i*-Hex, 50/50) = 0.65.

^{1}H NMR (400 MHz, CD_2Cl_2): δ / ppm = 7.72 (dd, *J* = 7.6, 1.1 Hz, 1H, H-C(1)), 7.66 (dd, *J* = 7.7, 1.1 Hz, 1H, H-C(3)), 7.52 (d, *J* = 8.8 Hz, 1H, H-C(17)), 7.27–7.17 (m, 16H, H-C(2+33+34+35)), 7.12–7.09 (m, 2H, H-C(29)), 6.81 (d, *J* = 8.8 Hz, 1H, H-C(16)), 6.78 (d, *J* = 8.9 Hz, 2H, H-C(28)), 4.32 (t, *J* = 5.1 Hz, 2H)*, 4.07–4.02 (m, 2H)*, 3.98 (t, *J* = 5.1 Hz, 2H)*, 3.76–3.72 (m, 2H)*, 3.62–3.55 (m, 4H)*, 3.52 (m, 4H)*, 2.97 (s, 2H, H_2-C(12)), 1.53 (s, 6H, H_3-C(14)).

*TEG-chain H_2-C(19/20/21/22/23/24/25/26).

HRMS (EI): calcd. for $[C_{52}H_{48}{}^{79}Br_2O_6S]^+$: 958.1538; found: 958.1545.

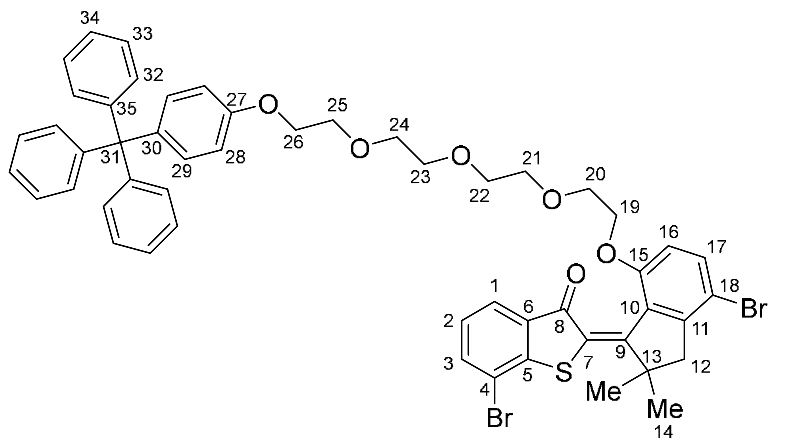

(E)-**7b**

R_f (EtOAc/*i*-Hex, 50/50) = 0.29.

^{1}H NMR (601 MHz, CD_2Cl_2): δ / ppm = 7.80 (dd, *J* = 7.6, 1.1 Hz, 1H, H-C(1)), 7.70 (dd, *J* = 7.7, 1.1 Hz, 1H, H-C(3)), 7.45 (d, *J* = 8.8 Hz, 1H, H-C(17)), 7.27–7.16 (m, 16H, H-C(2+32+33+34)), 7.11 (d, *J* = 9.1 Hz, 2H, H-C(29)), 6.78 (d, *J* = 9.1 Hz, 2H, H-C(28)), 6.72 (d, *J* = 8.8 Hz, 1H, H-C(16)), 4.17 (d, *J* = 4.2 Hz, 1H, H-C(19)), 4.16–4.10 (m, 1H, H-C(19)), 4.07–4.02 (m, 2H, H-C(26)), 3.77–3.72 (m, 2H, H-C(25)), 3.69–3.64 (m, 1H, H-C(24)), 3.58 (d, *J* = 9.4 Hz, 3H, H-C(20+24)), 3.47 (d, *J* = 4.4 Hz, 2H, H-C(23)), 3.37–3.30 (m, 3H, H-C(21+22)), 3.27 (dd, *J* = 7.3, 3.9 Hz, 1H, H-C(21)), 3.12 (d, *J* = 16.0 Hz, 1H, H_2-C(12)), 2.86 (d, *J* = 16.0 Hz, 1H, H_2-C(12)), 1.73 (s, 3H, H_3-C(14)), 1.26 (s, 3H, H_3-C(14)).

^{13}C NMR (151 MHz, CD_2Cl_2): δ / ppm = 186.6 (C(8)), 157.7 (C(9)), 157.4 (C(15)), 157.2 (C(27)), 145.6 (C(35)), 139.5 (C(5)), 136.8 (C(3)), 134.7 (C(17)), 134.4 (C(6)), 132.4 (C(29)), 131.3 (C(32)), 129.9 (C(10)), 127.8 (C(33)), 127.2 (C(7)), 126.7 (C(2)), 126.2 (C(34)), 125.0 (C(1)), 117.7 (C(4)), 113.7 (C(28)), 112.4 (C(16)), 110.3 (C(18)), 71.0 (C(24)), 71.0 (C(21)), 70.8 (C(23)), 70.6 (C(22)), 70.0 (C(25)), 69.7 (C(20)), 68.2 (C(19)), 67.7 (C(26)), 64.7 (C(31)), 50.6 (C(12)), 50.4 (C(13)), 26.6 (C(14)), 26.2 (C(14)).

HRMS (EI): calcd. for $[C_{52}H_{48}{}^{79}Br_2O_6S]^+$: 958.1538; found: 958.1576.

HTI (*E*/*Z*)-8a

Compound **7a** (289 mg of a mixture with TEG-tosylate and TEG-iodide, total content of **7a** as judged by ^{1}H NMR spectroscopy: 0.33 mmol, 1.0 equiv.), CuI (9 mg, 0.05 mmol, 15 mol-%) and XPhos Pd G2 (31 mg, 0.04 mmol, 12 mol-%) were added to a Schlenk-flask equipped with a septum and a magnetic stirring bar. The flask was evacuated and refilled with nitrogen three times. Dry 1,4-dioxane (2.0 mL) and dry diisopropylamine (2.0 mL) were added and the flask was evacuated and refilled with nitrogen three times under vigorous stirring. 3-Butyn-1-ol (78 µL, 1.0 mmol, 3.0 eq) was added and the reaction mixture was stirred at 65 °C under nitrogen for 4.5 h. The reaction was stopped by the addition of sat. aq. NH_4Cl (10 mL). H_2O (50 mL) was added and the mixture was extracted with CH_2Cl_2 (2 × 50 mL). The combined organic phases were dried over sodium sulphate, filtered, and concentrated *in vacuo*. The crude product was purified by flash column chromatography (SiO_2, MeOH/CH_2Cl_2, 5/95) to give **8a** (mixture of (*E*)- and (*Z*)-isomers, 172 mg, 0.27 mmol, 82%) as a red oil.

R_f (MeOH/CH_2Cl_2, 5/95) = 0.32.

Note: NMR analysis was performed with a (*E*)/(*Z*) = 2/1 mixture.

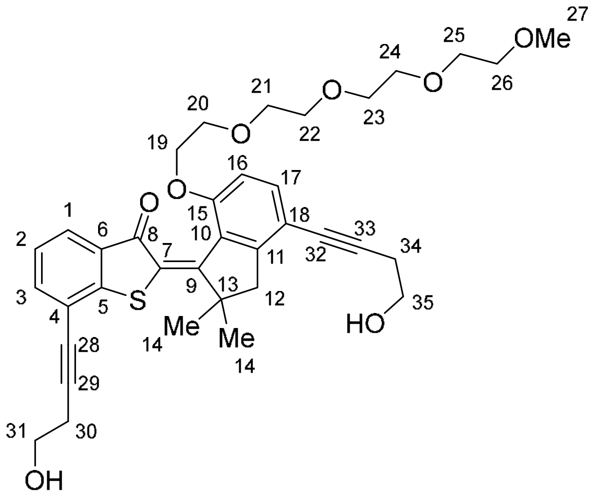

(*E*)-**8a**

^{1}H NMR (800 MHz, CD_2Cl_2): δ / ppm = 7.76 (dd, *J* = 7.6, 1.3 Hz, 1H, H-C(1)), 7.59 (dd, *J* = 7.4, 1.3 Hz, 1H, H-C(3)), 7.39 (d, *J* = 8.5 Hz, 1H, H-C(17)), 7.25 (t, *J* = 7.6 Hz, 1H, H-C(2)), 6.76 (d, *J* = 8.5 Hz, 1H, H-C(16)), 4.20 (dt, *J* = 10.0, 4.1 Hz, 1H, H_2-C(19)), 4.18–4.14 (m, 1H, H_2-C(19)), 3.85 (t, *J* = 6.3 Hz, 2H, H_2-C(31)), 3.78 (t, *J* = 5.4 Hz, 2H, H_2-C(35)), 3.68 (dt, *J* = 10.9, 4.0 Hz, 1H, H_2-C(20)), 3.60–3.57 (m, 1H, H_2-C(20)), 3.55–3.51 (m)*, 3.51–3.49 (m)*, 3.49–3.46 (m)*, 3.43–3.41 (m)*, 3.32 (s, 3H, H_3-C(27)), 3.30–3.24 (m, 2H, H_2-C(21)), 3.15 (d, *J* = 16.0 Hz, 1H, H_2-C(12)), 2.93 (d, *J* = 15.9 Hz, 1H, H_2-C(12)), 2.79 (t, *J* = 6.3 Hz, 2H, H_2-C(30)), 2.69 (t, *J* = 6.0 Hz, 2H), 2.12 (s, 1H), 1.93 (s, 1H), 1.73 (s, 3H, H_3-C(14)), 1.25 (s, 3H, H_3-C(14)).

^{13}C NMR (201 MHz, DCM-d_2): δ / ppm = 187.0 (C(8)), 157.8 (C(9)), 157.4 (C(15)), 152.0 (C(18)), 147.1 (C(5)), 136.6 (C(3)), 135.2 (C(17)), 132.5 (C(4)), 128.5 (C(10)), 126.4 (C(7)), 125.4 (C(1)), 125.2 (C(2)), 119.2 (C(6)), 112.6 (C(11)), 110.6 (C(16)), 94.6 (C(29)), 89.0 (C(33)), 79.8 (C(32)), 78.3 (C(28)), 72.3*, 72.2*, 71.0*, 70.8*, 70.7*, 70.5*, 69.8 (C(20)), 68.0 (C(19)), 61.6 (C(35)), 61.4 (C(31)), 59.0 (C(27)), 50.7 (C(13)), 49.3 (C(12)), 26.7 (C(14)), 26.3 (C(14)), 24.4 (C(30)), 24.3 (C(34)).

*Due to signal overlap, full assignment of the TEG chain signals was not possible.

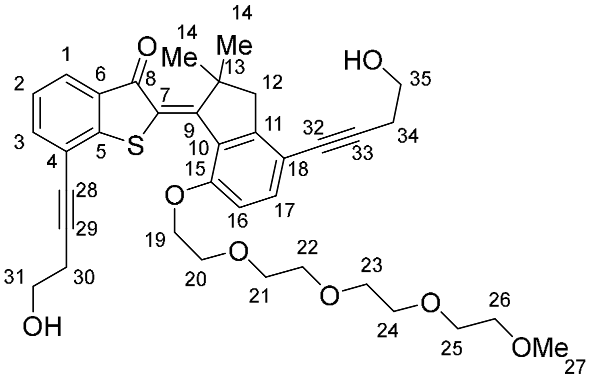

(*Z*)-**8a**

^{1}H NMR (800 MHz, CD_2Cl_2): δ / ppm = 7.68 (dd, J = 7.7, 1.2 Hz, 1H, H-C(1)), 7.53 (dd, J = 7.5, 1.2 Hz, 1H, H-C(3)), 7.45 (d, J = 8.5 Hz, 1H, H-C(17)), 7.39 (d, J = 8.5 Hz, 2H), 7.17 (t, J = 7.6 Hz, 1H), 6.85 (d, J = 8.6 Hz, 1H, H-C(16)), 4.34 (t, J = 5.5 Hz, 2H, H_2-C(19)), 4.01 (t, J = 5.5 Hz, 2H, H_2-C(20)), 3.78 (t, J = 5.4 Hz, 4H, H_2-C(31 and 35)), 3.64–3.61 (m, 2H, H_2-C(21)), 3.55–3.51 (m)*, 3.51–3.49 (m)*, 3.49–3.46 (m)*, 3.43–3.41 (m)*, 3.32 (s, 3H, H_3-C(27)), 3.02 (s, 2H, H_2-C(12)), 2.71 (t, J = 6.3 Hz, 2H, H_2-C(30)), 2.69 (t, J = 5.9 Hz, 2H, H_2-C(34)), 1.53 (s, 5H, H_3-C(14)).

^{13}C NMR (201 MHz, CD_2Cl_2): δ / ppm = 189.0 (C(8)), 162.8 (C(9)), 155.6 (C(15)), 153.2 (C(18)), 149.2 (C(5)), 136.6 (C(3)), 136.4 (C(17)), 132.5 (C(4)), 129.1 (C(7)), 128.0 (C(10)), 125.2 (C(1)), 124.7 (C(2)), 118.6 (C(6)), 113.2 (C(11)), 111.2 (C(16)), 94.8 (C(29)), 89.7 (C(33)), 79.5 (C(32)), 78.1 (C(28)), 72.3*, 72.2*, 71.2 (C(21)), 71.0*, 70.8*, 70.7*, 70.5*, 69.5 (C(20)), 68.2 (C(19)), 61.6 (C(31 or 35)), 61.3 (C(31 or 35)), 59.0 (C(27)), 51.3 (C(13)), 51.2 (C(12)), 26.6 (C(14)), 24.4, 24.32 (C(30 or 34)), 24.26 (C(30 or 34)).

*Due to signal overlap, full assignment of the TEG chain signals was not possible.

HRMS (ESI$^+$) calcd. for $[C_{36}H_{42}O_8S+H]^+$: 635.2673; found: 635.2688.

HTI (*E*/*Z*)-8b

Compound **7b** (mixture of (*E*)- and (*Z*)-isomers) (380 mg, 0.4 mmol, 1.0 equiv.), CuI (6 mg, 0.03 mmol, 8 mol-%) and XPhos Pd G2 (29 mg, 0.04 mmol, 9 mol-%) were added to a Schlenk-flask equipped with a magnetic stirring bar and a septum. The flask was evacuated and refilled with nitrogen three times. Dry 1,4-dioxan (2.0 mL) and dry *N*,*N*,-diisopropylamine (2.0 mL) were added and the flask was evacuated and refilled with nitrogen five times while stirring vigorously. 3-Butyn-1-ol (75 µl, 1.0 mmol, 2.5 equiv.) was added and the reaction mixture was stirred at 65 °C for 5 h. The reaction was stopped by the addition of sat. aq. NH_4Cl (15 mL). The mixture was diluted with H_2O (75 mL) and extracted with CH_2Cl_2 (2 × 100 mL). The combined organic phases were dried over sodium sulphate, filtered, and concentrated *in vacuo*. The residue was purified by flash column chromatography (SiO_2, MeOH/ CH_2Cl_2, 5/95) to give compound **8b** (mixture of (*E*)- and (*Z*)-isomers, 277 mg, 0.3 mmol, 75%) as an orange foam.

R_f (MeOH/CH_2Cl_2, 5/95) = 0.44.

HRMS (ESI$^+$) calcd. for $[C_{60}H_{58}O_8S+H]^+$: 939.3925; found: 939.3944.

NMR analysis was performed on a 2:1 (*E*):(*Z*) mixture.

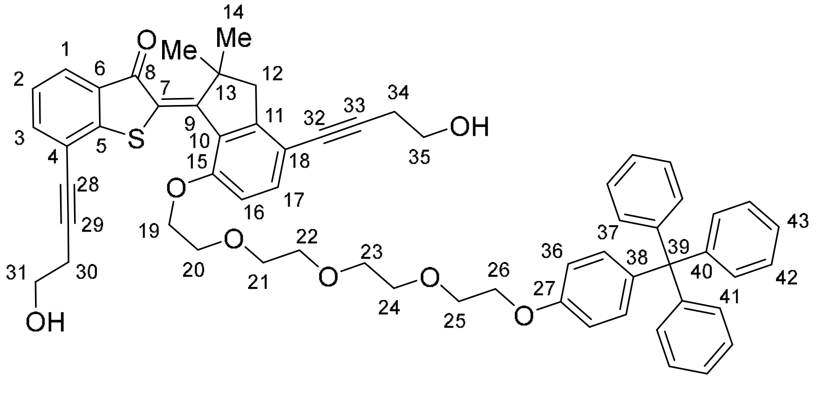

(*Z*)-**8b**

^{1}H NMR (400 MHz, CD_2Cl_2): δ / ppm = 7.67 (dd, *J* = 7.7, 1.3 Hz, 1H, H-C(1), 7.51 (dd, *J* = 7.5, 1.3 Hz, 1H, H-C(3)), 7.43 (d, *J* = 8.6 Hz, 1H, H-C(17)), 7.29–7.17 (m, 15H, H-C(41+42+43), 7.15 (s, 1H, H-C(2)), 7.14–7.09 (m, 2H, H-C(37)), 6.83 (d, *J* = 8.6 Hz, 1H, H-C(16)), 6.81–6.77 (m, 2H, H-C(36)), 4.32 (t, *J* = 5.5 Hz, 2H, H_2-C(19)), 4.00 (t, *J* = 5.4 Hz, 2H, H_2-C(20)), 3.81–3.77 (m, 4H, H_2C-(31+35)), 3.70–3.64 (m)*, 3.63–3.60 (m)*, 3.60–3.56 (m)*, 3.56–3.51 (m)*, 3.45 (dd, *J* = 5.7, 4.0 Hz)*, 3.36–3.24 (m)*, 3.02 (s, 2H, H_2C-(12)), 2.69 (tt, *J* = 6.3, 2.2 Hz, 4H, H_2C-(30+34)), 1.53 (s, 6H, H_3C-(14)).

*Due to signal overlap of (*E*)- and (*Z*)-isomer, the TEG-chain signals could not be fully assigned.

^{13}C NMR (101 MHz, CD_2Cl_2): δ / ppm = 189.0 (C(8)), 162.9 (C(9)), 157.2 (C(27)), 155.6*, 153.2*, 149.2 (C(5)), 147.5 (C(40)), 136.6 (C(3)), 136.4 (C(17)), 132.6*, 132.54*, 131.4 (C(41/42)), 128.5*, 127.9 (C(41/42)), 126.4*, 126.2 (C(43)), 125.2 (C(1)), 124.7 (C(2)), 118.6 (C(4)), 113.7 (C(36)), 111.3 (C(16)), 94.8 (C(29/33)), 89.7 (C(29/33)), 79.5 (C(28/32)), 78.1 (C(28/32)), 71.2‡, 71.0‡, 71.0‡, 71.0‡, 70.9‡, 70.8‡, 70.7‡, 70.5‡, 69.5 (C(20)), 68.2 (C(19)), 67.7*, 64.7 (C(39)), 61.6 (C(31/35)), 61.3 (C(31/35)), 51.3 (C(12/13)), 51.2 (C(12/13)), 26.6 (C(14)), 24.34 (C(30/34)), 24.28 (C(30/34)).

*Could not be assigned unambiguously.

‡Due to signal overlap of the (*E*)- and the (*Z*)-isomer the TEG-chain signals could not be fully assigned.

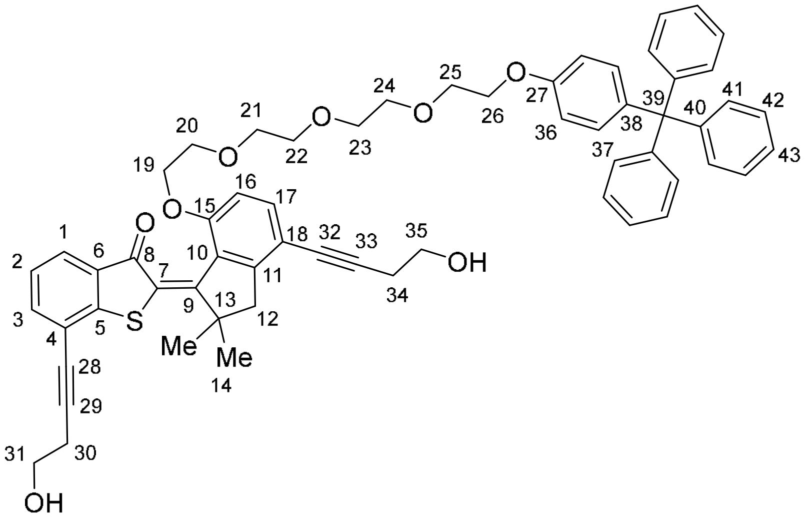

(*E*)-**8b**

^{1}H NMR (400 MHz, CD_2Cl_2): δ / ppm = 7.76 (dd, J = 7.7, 1.3 Hz, 1H, H-C(1)), 7.57 (dd, J = 7.5, 1.3 Hz, 1H, H-C(3)), 7.38 (d, J = 8.5 Hz, 1H, H-C(17)), 7.29–7.17 (m, 16H, H-C(2+41+42+43)), 7.14–7.09 (m, 2H, H-C(37)), 6.81–6.77 (m, 2H, H-C(36)), 6.74 (d, J = 8.6 Hz, 1H, H-C(16)), 4.22–4.14 (m, 2H, H_2-C(19)), 4.07–4.04 (m, 2H, H_2-C(26)), 3.84 (s, 2H, H_2-C(31)), 3.81–3.77 (m, 2H, H_2-C(35)), 3.78–3.72 (m, 2H, H2-C(25)), 3.70–3.64 (m, 1H, H_2-C(20)), 3.63–3.56 (m, 3H, H_2-C(20+24)), 3.56–3.51 (m)*, 3.45 (dd, J = 5.7, 4.0 Hz)*, 3.36–3.24 (m)*, 3.15 (d, J = 16.9 Hz, 1H, H_2-C(12)), 2.93 (d, J = 16.0 Hz, 1H, H_2-C(12)), 2.77 (t, J = 6.3 Hz, 2H, H_2-C(30)), 2.69 (tt, J = 6.3, 2.2 Hz, 2H, H_2-C(34)), 1.73 (s, 3H, H_3-C(14)), 1.25 (s, 3H, H_3-C(14)).

*Due to signal overlap of the (*E*)- and the (*Z*)-isomer the TEG-chain signals could not be fully assigned.

^{13}C NMR (101 MHz, CD_2Cl_2): δ / ppm = 187.0 (C(8)), 157.8 (C(10/15)), 157.4 (C(10/15)), 157.18 (C(27)), 157.16*, 155.6*, 153.2*, 152.0 (C(11)), 147.5 (C(40)), 147.1 (C(5)), 139.6 (C(38)), 136.6 (C(3)), 135.2 (C(17)), 132.6*,132.57*, 132.54 (C(37)), 131.4 (C(41/42)), 128.5*, 127.9 (C(41/42)), 126.4*, 126.2 (C(43)), 125.5 (C(1)), 125.2 (C(2)), 119.2 (C(4)), 113.7 (C(36)), 112.6 (C(18)), 110.6 (C(16)), 94.6 (C(29)), 89.0 (C(33)), 79.8 (C(32)), 78.3 (C(28)), 71.2‡, 71.0‡, 71.0‡, 71.0‡, 70.9‡, 70.8‡, 70.7‡, 70.5‡, 70.0 (C(25)),

69.8 (C(20)), 68.0 (C(19)), 67.8 (C(26)), 67.7*, 64.7 (C(39)), 61.61 (C(35)), 61.4 (C(31)), 50.7 (C(13)), 49.4 (C(12)), 26.8 (C(14)), 26.3 (C(14)), 24.4 (C(30)), 24.31 (C(34)).

*Could not be assigned unambiguously.

‡Due to signal overlap of the (*E*)- and the (*Z*)-isomer the TEG-chain signals could not be fully assigned.

Macrocycle (*E/Z*)-9a

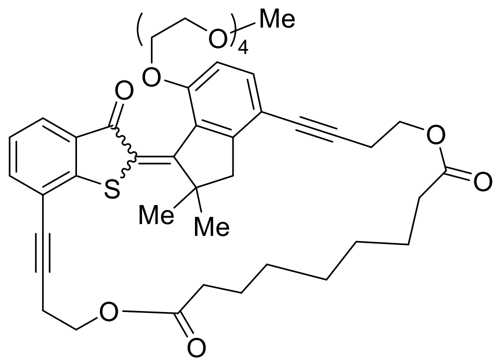

Azelaoyl chloride preparation

Azelaic acid (191 mg, 1.0 mmol, 1.0 equiv.) was refluxed in thionyl chloride (1.3 mL, 17.8 mmol, 17.8 eq) for 3.75 h. Excess thionyl chloride was removed *in vacuo* and the residue was dissolved in dry CH_2Cl_2 (2 mL) to give a 0.5 M solution of azelaoyl chloride, which was used without further purification.

Macrocyclization

In a flame-dried Schlenk flask under nitrogen atmosphere azelaoyl chloride (0.5 M in CH_2Cl_2, 0.28 mL, 0.14 mmol, 1.0 eq) was added to a solution of 4-dimethylaminopyridine (33 mg, 0.27 mmol, 1.9 eq) in dry CH_2Cl_2 (120 mL). The reaction mixture was stirred at room temperature (23 °C) for 5 min, then compound **8a** (117-1) (90 mg in 3.4 mL dry CH_2Cl_2, 0.14 mmol, 1.0 eq) was added and stirring was continued for 7 d. The solvent was removed *in vacuo* and the residue was purified by flash column chromatography (SiO_2, MeOH/DCM, 2/98). Compound **9a** (mixture of (*E*) and (*Z*)-isomers, 40 mg, 0.05 mmol, 36%) was obtained as an orange oil. The compound was used in the next synthetic step without full characterization.

HRMS (ESI^+) calcd for $[C_{45}H_{54}O_{10}S+NH_4]^+$: 804.3776; found: 804.3771.

Macrocycle (*E*/*Z*)-9b

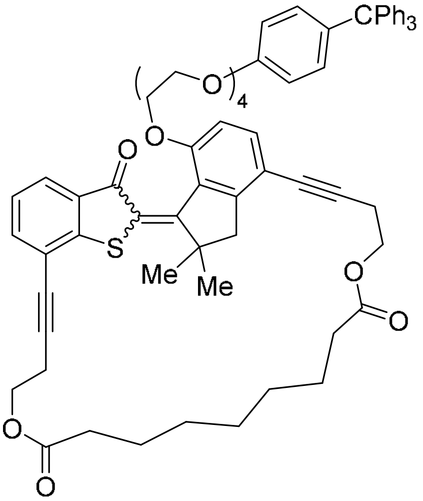

Azelaoyl chloride preparation

Azelaic acid (95 mg, 0.5 mmol, 1.0 eq) was refluxed in thionyl chloride (1.0 mL, 13.7 mmol, 27.4 equiv.) for 1.5 h. Excess thionyl chloride was removed *in vacuo* and the residue was dissolved in dry CH_2Cl_2 (1 mL) to give a 0.5 M solution of azelaoyl chloride, which was used without further purification.

Macrocyclization

In a flame-dried Schlenk flask under nitrogen atmosphere azelaoyl chloride (0.5 M in CH_2Cl_2, 0.1 mL, 0.05 mmol, 1.0 equiv.) was added to a solution of 4-dimethylaminopyridine (14 mg, 0.11 mmol, 2.2 equiv.) in dry CH_2Cl_2 (50 mL). The reaction mixture was stirred at 23 °C for 5 min, then compound **8b** (mixture of (*E*)- and (*Z*)-isomers, 44 mg in 5.0 mL dry CH_2Cl_2, 0.05 mmol, 1.0 equiv.) was added and stirring was continued for 6 d. The solvent was removed *in vacuo* and the residue was purified by flash column chromatography (SiO_2, MeOH/CH_2Cl_2, 1/99). Compound **9b** (mixture of (E)- and (Z)-isomers, 25 mg, 0.02 mmol, 40%) was obtained as an orange oil. The compound was used for the next synthetic step without full characterization.

HRMS (ESI$^+$) calcd for $[C_{69}H_{70}O_{10}S+NH_4]^+$: 1108.5028; found: 1108.5050.

Macrocyclic Motor 1

Compound **9a** (35 mg, 44 µmol, 1.0 equiv.) was dissolved in acetic acid (1.5 mL). Sodium perborate tetrahydrate (26 mg, 169 µmol, 3.8 equiv.) was added and the reaction mixture was stirred at 23 °C in the dark for 2 h. The reaction was stopped by the slow addition of sat. aq. $NaHCO_3$ (5 mL). H_2O (25 mL) and an additional 5 mL of sat. aq. $NaHCO_3$ were added and the mixture was extracted with EtOAc (2 × 25 mL). The combined organic phases were dried over sodium sulphate, filtered, and concentrated *in vacuo*. The residue was purified by flash column chromatography (SiO_2, MeOH/CH_2Cl_2, 2/98). Isomer **A-1** (24 mg, 30 µmol, 68%) and **C-1** (8 mg, 10 µmol, 23%) were obtained separately as yellow oils (combined yield: 91%).

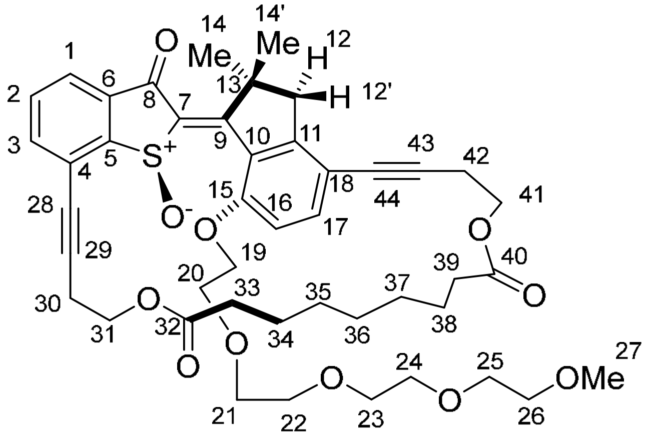

A-1

R_f (MeOH/CH_2Cl_2, 2/98): 0.13.

^{1}H NMR (601 MHz, CD_2Cl_2): δ / ppm = 7.85 (dd, J = 7.7, 1.1 Hz, 1H, H-C(1)), 7.73 (dd, J = 7.6, 1.2 Hz, 1H, H-C(3)), 7.61 (t, J = 7.6 Hz, 1H, H-C(2)), 7.48 (d, J = 8.6 Hz, 1H, H-C(17)), 6.87 (d, J = 8.6 Hz, 1H, H-C(16)), 4.47 (dt, J = 10.4, 4.2 Hz, 1H, H-C(19)), 4.40 (ddd, J = 10.4, 7.8, 4.0 Hz, 1H, H-C(19)), 4.32–4.27 (m, 1H, H-C(31)), 4.27–4.22 (m, 2H, H-C(41)), 4.11–4.08 (m, 1H, H-C(20)), 4.08–4.04 (m, 1H, H-C(31)), 3.91 (dt, J = 10.9, 4.1 Hz, 1H, H-C(20)), 3.62–3.55 (m, 2H, H-C(21)), 3.52–3.47 (m, 6H, H-C(22, 23, 24)), 3.47–3.44 (m, 4H, H-C(25, 26)), 3.30 (s, 3H, H-C(27)), 3.14 (d, J = 16.6 Hz, 1H, H-C(12')), 2.97 (d, J = 16.7 Hz, 1H, H-C(12)), 2.89–2.81 (m, 2H, H-C(30)), 2.79–2.75 (m, 2H, H-C(42)), 2.35–2.23 (m, 2H, H-C(33)), 2.19–2.08 (m, 2H, H-C(36)), 1.59–1.55 (m, 2H, H-C(34)), 1.54 (s, 3H, H-C(14)), 1.50 (s, 3H, H-C(14')), 1.31–1.24 (m, 2H, H-C(34)), 1.23–1.15 (m, 2H, H-C(37)), 1.08–1.00 (m, 1H, H-C(36)), 0.95 (dd, J = 18.8, 9.1 Hz, 1H, H-C(36)), 0.83–0.75 (m, 1H, H-C(35)), 0.75–0.65 (m, 1H, H-C(35)).

^{13}C NMR (151 MHz, CD_2Cl_2): δ / ppm = 184.7 (C(8)), 174.8 (C(32)), 173.8, (C(40)), 170.9 (C(9)), 156.8 (C(15)), 153.7 (C(11)), 151.2 (C(5)), 143.3 (C(7)), 137.9 (C(3)), 137.9 (C(19)), 136.8 (C(6)), 132.1 (C(2)), 125.7 (C(10)), 124.0 (C(1)), 123.6 (C(4)), 113.1 (C(18)), 110.4 (C(16)), 96.8 (C(29)), 89.3 (C(43)), 78.9

(C(44)), 76.5 (C(28)), 72.1 (C(26)), 70.9 (C(21)), 70.7 (C(22)), 70.6 (C(24)), 70.6 (C(23)), 70.6 (C(25)), 69.3 (C(20)), 68.5 (C(19)), 62.2 (C(31)), 62.0 (C(41)), 58.9 (C(27)), 51.8 (C(13)), 50.4 (C(12)), 35.4 (C(39)), 33.8 (C(33)), 30.2 (C(36)), 29.9 (C(37)), 29.5 (C(35)), 28.2 (C(14')), 26.1 (C(38)), 25.5 (C(14)), 25.3 (C(34)), 20.5 (C(42)), 20.4 (C(30)).

HRMS (ESI$^+$) calcd for $[C_{45}H_{54}O_{11}S+H]^+$: 803.3460; found: 803.3465.

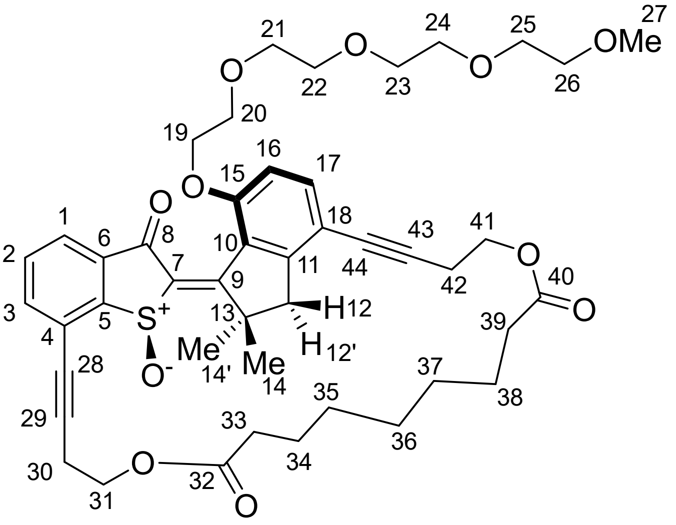

C-1

R_f (MeOH/CH_2Cl_2, 2/98): 0.06.

^{1}H NMR (601 MHz, CD_2Cl_2): δ / ppm = 7.89 (dd, J = 7.6, 1.1 Hz, 1H, H-C(1)), 7.76 (dd, J = 7.6, 1.2 Hz, 1H, H-C(3)), 7.68 (t, J = 7.6 Hz, 1H, H-C(2)), 7.43 (d, J = 8.5 Hz, 1H, H-C(17)), 6.81 (d, J = 8.6 Hz, 1H, H-C(16)), 4.36–4.31 (m, 2H, H-C(31)), 4.31–4.26 (m, 2H, H-C(41)), 4.21 (ddd, J = 16.9, 10.0, 4.6 Hz, 2H, H-C(19)), 3.75–3.70 (m, 1H, H-C(20)), 3.65 (ddd, J = 10.9, 6.7, 4.7 Hz, 1H, H-C(20)), 3.56–3.51 (m, 4H, H-C(23, 24, 25)), 3.49–3.46 (m, 4H, H-C(23, 24, 26)), 3.40 (d, J = 1.8 Hz, 4H, H-C(21, 22)), 3.32 (s, 3H, H-C(27)), 3.14 (d, J = 13.9 Hz, 1H, H-C(12)), 2.98 (d, J = 16.7 Hz, 1H, H-C(12')), 2.96–2.90 (m, 1H, H-C(30)), 2.88–2.82 (m, 1H, H-C(30)), 2.77 (t, J = 5.5 Hz, 2H, H-C(42)), 2.50–2.46 (m, 2H, H-C(33)), 2.42–2.38 (m, 2H, H-C(39)), 2.01 (s, 3H, H-C(14')), 1.74–1.63 (m, 4H, H-C(34, 38)), 1.43 (s, 3H, H-C(14)), 1.39 (s, 6H, H-C(35, 36, 37)).

^{13}C NMR (DEPTq)(151 MHz, CD_2Cl_2): δ / ppm = 184.8 (C(8)), 174.1 (C(32)), 173.7(C(40)), (C(11)), 158.7 (C(15)), 154.0 (C(11)), 150.2 (C(5)), 137.4 (C(3)), 135.6 (C(17)), 133.0 (C(2)), 132.1, 129.8, 129.3, 127.7 (C(10)), 124.7 (C(1)),f 124.1 (C(4, 6)), 113.1 (C(18)), 110.9 (C(16)), 97.4 (C(29)), 90.4 (C(43)), 78.9 (C(44)), 76.5 (C(28)), 72.3 (C(26)), 71.1 (C(21, 22)), 70.8 (C(23)), 70.7 (C(24)), 70.6 (C(25)), 69.7 (C(21, 22)), 68.1 (C(20)), 63.0 (C(41)), 62.5 (C(31)), 59.0 (C(27)), 49.8 (C(12)), 34.8 (C(39)), 34.3 (C(33)), 30.2 (C(37)), 29.9 (C(35)), 29.4 (C(36)), 28.8 (C(21)), 28.3 (C(22)), 25.2 (C(38)), 25.0 (C(34)), 20.7 (C(30)), 20.5 (C(42)).

IR: $\tilde{\upsilon}$ / cm^{-1} = 3055 (m), 3020 (m), 2931 (s), 2868 (m), 1734 (vs), 1682 (s), 1593 (s), 1554 (s), 1489 (m), 1466 (m), 1385 (m), 1363 (m), 1338 (m), 1284 (s), 1261 (s), 1171 (s), 1134 (s), 1099 (s), 1076 (s), 1065 (s), 1053 (m), 1036 (m), 818 (m), 810 (m), 762 (m), 750 (m), 727 (m), 634 (m), 573 (m), 555 (m), 505 (m), 457 (m).

HRMS (ESI$^+$) calcd for $[C_{45}H_{54}O_{11}S+H]^+$: 803.3460; found: 803.3470.

Macrocyclic Motor 2

Compound **9b** (mixture of (E)- and (Z)-isomers, 45 mg, 41 µmol, 1.0 equiv.) was dissolved in acetic acid (1.2 mL). Sodium perborate tetrahydrate (25 mg, 164 µmol, 4.0 equiv.) was added and the reaction mixture was stirred at 23 °C in the dark for 2.5 h. The reaction was stopped by the slow addition of sat. aq. $NaHCO_3$ (5 mL). H_2O (25 mL) was added and the mixture was extracted with EtOAc (2 × 25 mL). The combined organic phases were dried over sodium sulphate, filtered, and concentrated *in vacuo*. The residue was purified by flash column chromatography (SiO_2, EtOAc/*i*-Hex, 70/30). Fractions were concentrated *in vacuo*, subsequently dissolved in a H_2O/MeCN mixture and then lyophilized. Isomer **A-2** (19 mg, 17 µmol, 41%) and **C-2** (10 mg, 9 µmol, 22%) could be isolated in pure form as fine powdered yellow solids (combined yield: 63%).

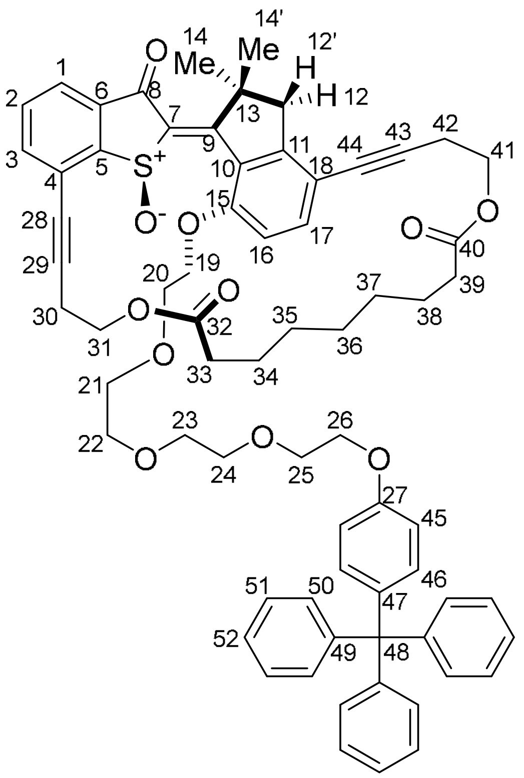

Isomer **A-2**

R_f (EtOAc/*i*-Hex, 70/30): 0.53.

^{1}H NMR (800 MHz, CD_2Cl_2): δ / ppm = 7.85 (dd, J = 7.7, 1.1 Hz, 1H, H-C(1)), 7.71 (dd, J = 7.6, 1.1 Hz, 1H, H-C(3)), 7.58 (t, J = 7.6 Hz, 1H, H-C(2)), 7.47 (d, J = 8.5 Hz, 1H, H-C(17)), 7.26–7.23 (m, 6H, H-C(51)), 7.22 (dd, J = 8.5, 1.6 Hz, 6H, H-C(50)), 7.20–7.17 (m, 3H, H-C(52)), 7.12–7.10 (m, 2H, H-C(46)), 6.86 (d, J = 8.3 Hz, 1H, H-C(16)), 6.79–6.76 (m, 2H, H-C(45)), 4.47 (dt, J = 10.5, 4.3 Hz, 1H, H-C(19)), 4.39 (ddd, J = 10.4, 7.7, 4.0 Hz, 1H, H-C(19)), 4.31–4.28 (m, 1H, H-C(31)), 4.28–4.24 (m, 2H, H-C(41)), 4.09–4.07 (m, 1H, H-C(20)), 4.07–4.05 (m, 1H, H-C(31)), 4.05–4.03 (m, 2H, H-C(26)), 3.91 (dt, J = 11.0, 4.3 Hz, 1H, H-C(20)), 3.74–3.72 (m, 2H, H-C(50)), 3.61–3.57 (m, 2H, H-C(21)), 3.57–3.55 (m, 2H, H-C(24)), 3.52–3.48 (m, 4H, H-C(22, 23)), 3.14 (d, J = 16.6 Hz, 1H, H-C(12')), 2.97 (d, J = 16.6 Hz, 1H, H-C(12)), 2.84 (ddd, J = 17.6, 9.5, 3.6 Hz, 1H, H-C(30)), 2.81–2.78 (m, 1H, H-C(30)), 2.77 (t, J = 5.5 Hz, 2H, H-C(42), 2.33–2.29 (m, 1H, H-C(39)), 2.27 (ddd, J = 14.2, 7.8, 5.9 Hz, 1H, H-C(39)), 2.16–2.12 (m, 2H, H-C(33)), 1.55 (s, 3H, H-C(14')), 1.50 (s, 3H, H-C(14)), 1.32–1.25 (m, 2H, H-C(34)), 1.24–1.18 (m, 2H, H-C(35)), 1.07–0.91 (m, 2H, H-C(36)), 0.83–0.70 (m, 2H, H-C(37)).

^{13}C NMR (201 MHz, CD_2Cl_2): δ / ppm = 184.8 (C(8)), 174.8 (C(32)), 173.8 (C(40)), 171.0 (C(9)), 157.2 (C(27)), 156.9 (C(15)), 153.8 (C(11)), 151.5 (C(5)), 147.5 (C(49)), 143.7 (C(7)), 139.5 (C(47)), 138.0 (C(3)), 137.9 (C(17)), 137.0 (C(6)), 132.4 (C(46)), 132.1 (C(2)), 131.4 (C(50)), 127.9 (C(51)), 126.2 (C(52)), 126.0 (C(10)), 124.1 (C(1)), 123.8 (C(4)), 113.7 (C(45)), 113.3 (C(18)), 110.6 (C(16)), 96.9 (C(29)), 89.4 (C(43)), 79.1 (C(44)), 76.6 (C(28)), 71.0 (C(21)), 71.0 (C(22)), 70.8 (C(23)), 70.8 (C(24)), 70.0 (C(25)), 69.5 (C(20)), 68.6 (C(19)), 67.7 (C(26)), 64.7 (C(48)), 62.2 (C(31)), 62.1 (C(41)), 51.9 (C(13)), 50.6 (C(12)), 35.5 (C(39)), 34.0 (C(33)), 30.2 (C(36)), 29.9 (C(37)), 29.6 (C(35)), 28.3 (C(14)), 26.2 (C(38)), 25.6 (C(14')), 25.4 (C(34)), 20.6 (C(30)), 20.5 (C(42)).

IR: $\tilde{\upsilon}$ / cm^{-1} = 3020 (s), 2987 (s), 2929 (s), 2922 (s), 1732 (vs), 1670 (s), 1547 (s), 1489 (s), 1417 (s), 1369 (s), 1282 (s), 1257 (vs), 1174 (s), 1119 (s), 1095 (s), 1086 (s), 1068 (s), 856 (s), 823 (s), 810 (s), 785 (s), 762 (s), 750 (s), 702 (s), 642 (s), 604 (s), 509 (s), 463 (s).

HRMS (ESI^+) calcd. for $[C_{69}H_{70}O_{11}S+H]^+$: 1107.4712; found: 1107.4736.

Enantiomers were separated on a Daicel Chiralpak IC semi-preparative column eluting with EtOAC/*n*-heptane 25/75 at 30 °C.

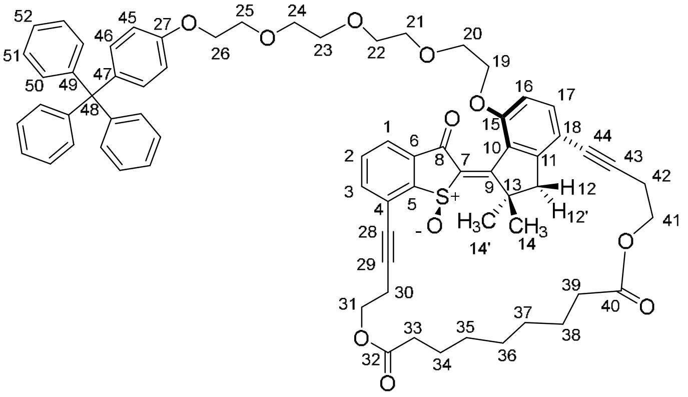

Isomer **C-2**

R_f (EtOAc/*i*-Hex, 70/30): 0.14.

^{1}H NMR (800 MHz, CD_2Cl_2): δ / ppm = 7.88 (d, *J* = 6.3 Hz, 1H, H-C(1)), 7.73 (d, *J* = 7.6 Hz, 1H, H-C(3)), 7.66 (t, *J* = 7.5 Hz, 1H, H-C(2)), 7.41 (d, *J* = 8.5 Hz, 1H, H-C(17)), 7.26–7.23 (m, 6H, H-C(51)), 7.23–7.21 (m, 6H, H-C(50)), 7.20–7.17 (m, 3H, H-C(52)), 7.13–7.10 (m, 2H, H-C(46)), 6.79 (dd, *J* = 8.7, 3.4 Hz, 3H, H-C(16, 45)), 4.34–4.30 (m, 2H, H-C(31)), 4.30–4.23 (m, 2H, H-C(41)), 4.23–4.17 (m, 2H, H-C(19)), 4.07–4.05 (m, 2H, H-C(26)), 3.78–3.74 (m, 2H, H-C(25)), 3.74–3.69 (m, 1H, H-C(20)), 3.66–3.62 (m, 1H, H-C(20)), 3.62–3.58 (m, 2H, H-C(24)), 3.53–3.48 (m, 2H, H-C(23)), 3.41 (d, *J* = 12.3 Hz, 4H, H-C(21, 22)), 3.15 (d, *J* = 16.0 Hz, 1H, H-C(12)), 2.97 (d, *J* = 21.7 Hz, 1H, H-C(12)), 2.92 (ddd, *J* = 17.7, 7.5, 3.6 Hz, 1H, H-C(30)), 2.83 (ddd, *J* = 17.7, 7.0, 3.5 Hz, 1H, H-C(30)), 2.76 (t, *J* = 5.5 Hz, 2H, H-C(42)), 2.47 (t, *J* = 8.3 Hz, 2H, H-C(33)), 2.41–2.38 (m, 2H, H-C(39)), 2.01 (s, 3H, H-C(14 o. 14‘), 1.74–1.63 (m, 4H, H-C(34, 38)), 1.42 (s, 3H, H-C(14 o. 14‘)), 1.39 (s, 6H, H-C(35, 36, 37)).

^{13}C NMR (201 MHz, CD_2Cl_2): δ / ppm = 182.6 (C(9)), 174.1 (C(32)), 173.7 (C(40)), 158.6 (C(15)), 157.2 (C(27)), 154.0 (C(11)), 150.1 (C(5)), 147.5 (C(49)), 139.5 (C(47)), 137.4 (C(3)), 136.4 (C(6)), 135.6 (C(17)), 133.0 (C(2)), 132.5 (C(46)), 131.4 (C(50)), 127.9 (C(51)), 126.2 (C(52)), 124.7 (C(1)), 124.4 (C(4)), 113.7 (C(45)), 113.0 (C(18)), 110.9 (C(16)), 97.4 (C(29)), 90.4 (C(43)), 78.9 (C(44)), 76.4 (C(28)), 70.0 (C(25)), 69.7 (C(20)), 68.1 (C(19)), 67.8 (C(26)), 64.7 (C(48)), 63.0 (C(41)), 62.5 (C(31)), 52.4 (C(13)), 49.8 (C(12)), 34.8 (C(39)), 34.3 (C(33)), 30.2 (C(35 o. 36 o. 37)), 29.9 (C(35 o. 36 o. 37)), 29.4 (C(35 o. 36 o. 37)), 28.7 (C(14 o. 14‘), 27.5 (C(14 o. 14‘)), 25.2 (C(34 o. 38)), 25.0 (C(34 o. 38)), 20.7(C(30)), 20.5(C(42)).

IR: $\tilde{\upsilon}$ / cm^{-1} = 3020 (m), 3008 (m), 2956 (s), 2929 (s), 2862 (s), 1734 (vs), 1682 (s), 1593 (s), 1550 (s), 1508 (s), 1489 (s), 1466 (s), 1446 (s), 1338 (m), 1284 (s), 1255 (s), 1182 (s), 1173 (s), 1130 (s), 1119 (s), 1095 (s), 1080 (s), 1066 (s), 1036 (s), 818 (m), 764 (s), 750 (s), 702 (s), 463 (s), 457 (m), 444 (s), 432 (m).

HRMS (ESI$^+$) calcd. for $[C_{69}H_{70}O_{11}S+H]^+$: 1107.4712; found: 1107.4747.

Enantiomers were separated on a Daicel Chiralpak IC semi-preparative column eluting with EtOAC/*n*-heptane 50/50 at 30 °C.

Structural and Conformational Analysis

Structures in Solution

Isomer **C-1**

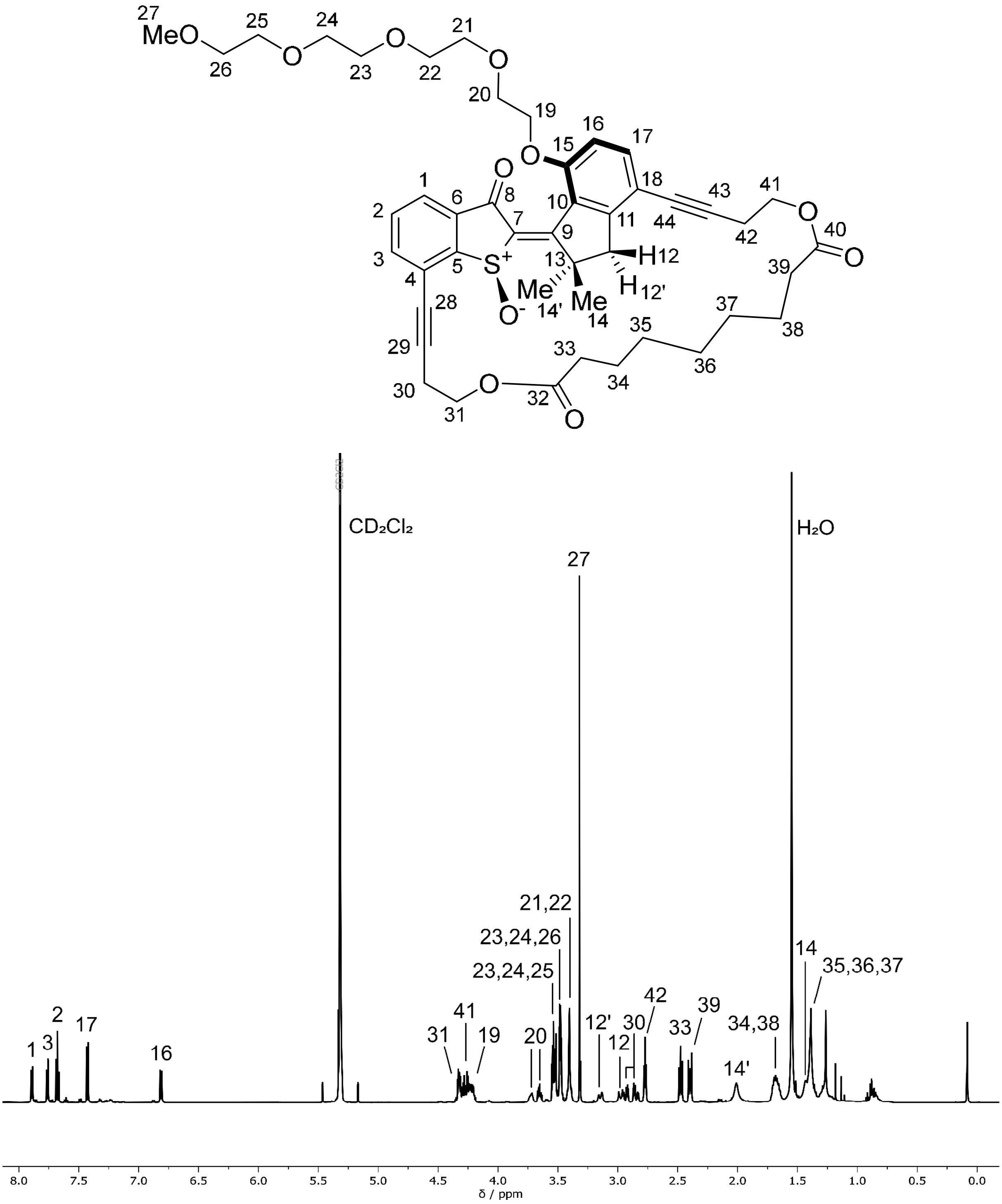

Figure 1 ^{1}H NMR (600 MHz, CD_2Cl_2, 25 °C) of racemic **C-1** and assignment of proton signals to the molecular structure. Residual small signals belong to small amounts of isomer **A-1**.

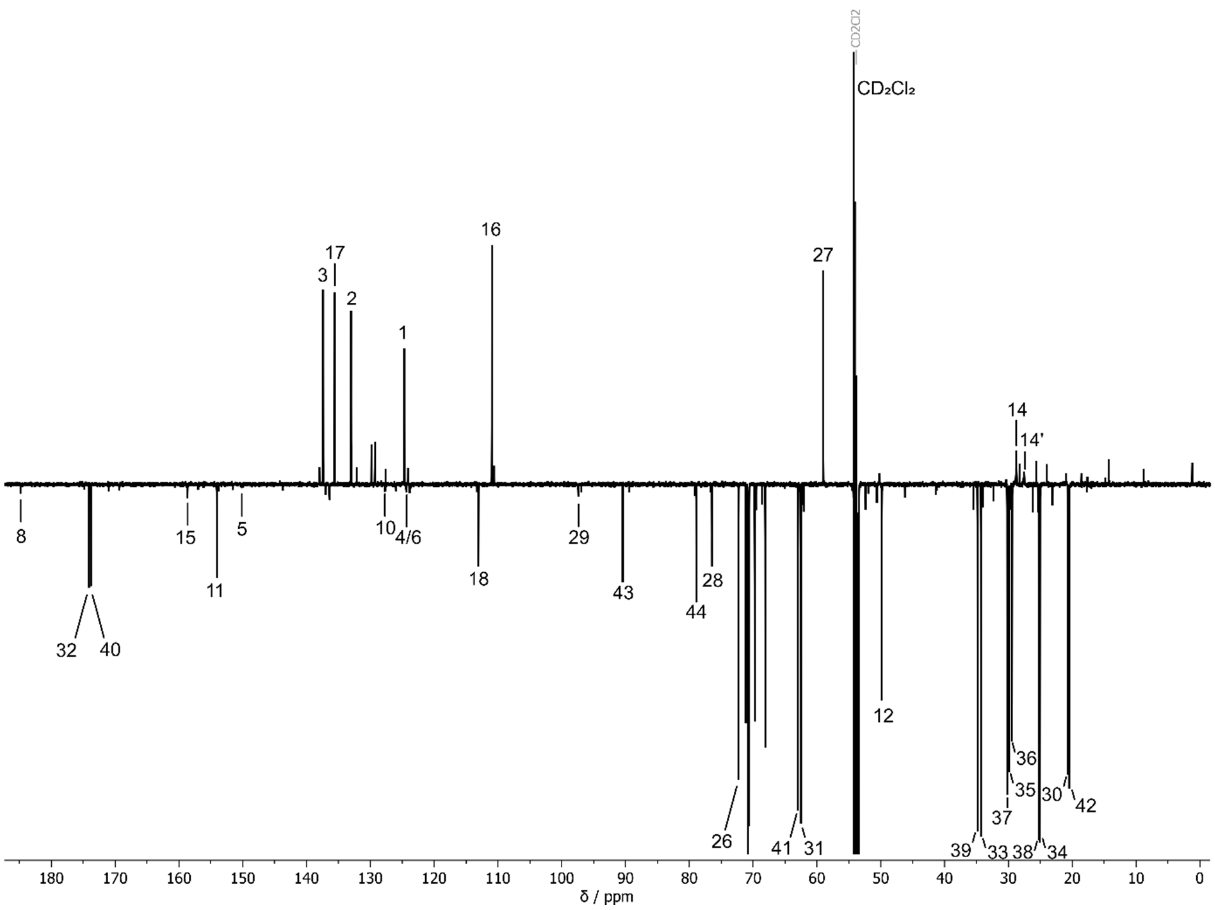

Figure 2 DEPTq NMR (600 MHz, CD_2Cl_2, 25 °C) of racemic **C-1** and assignment of the signals to the molecular structure. Residual small signals belong to small amounts of isomer **A-1**.

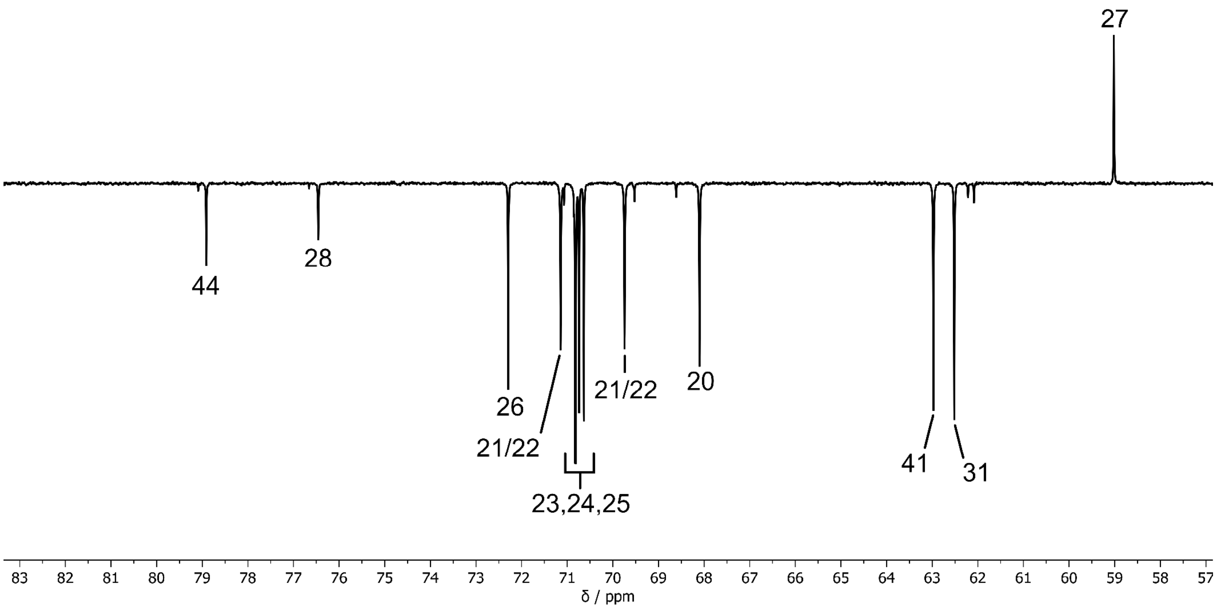

Figure 3 Magnified excerpt from the DEPTq NMR (600 MHz, CD_2Cl_2, 25 °C) spectrum of racemic **C-1**. Residual small signals belong to small amounts of isomer **A**-**1**.

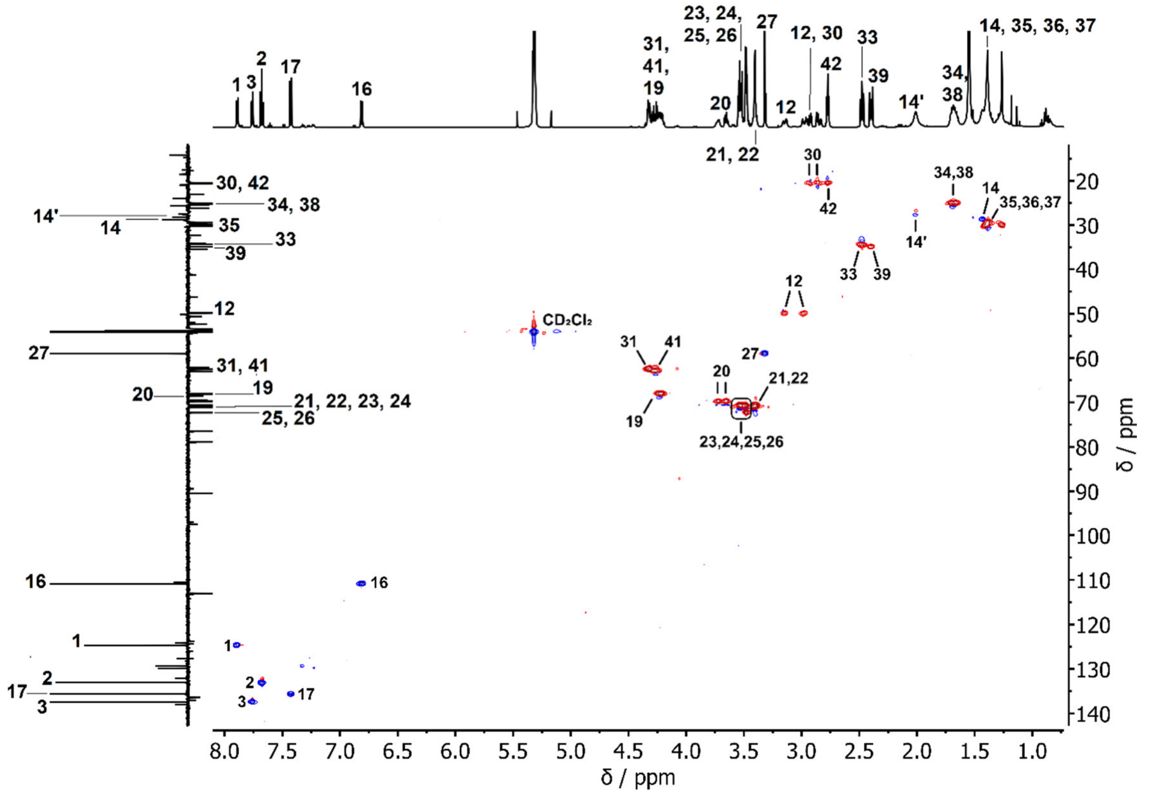

Figure 4 HSQC NMR spectrum (600 MHz, CD_2Cl_2, 25 °C) of racemic **C-1** and assignment of the signals to the molecular structure.

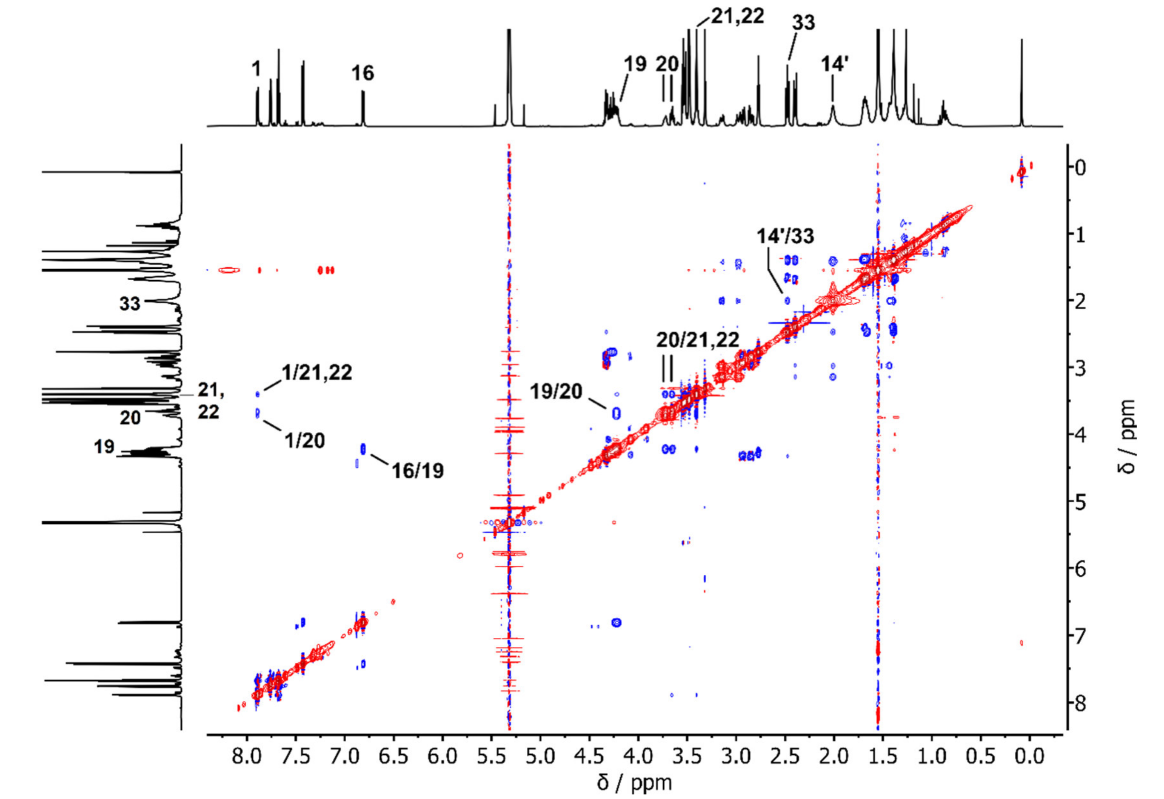

Figure 5 NOESY (600 MHz, CD_2Cl_2, 25 °C) spectrum of **C-1**. Cross signals between protons 1 and 20,21,22 (thioindigo fragment to TEG chain) as well as between protons 14' and 33 (methyl group to aliphatic macrocycle ring) confirm *(E)*-configuration.

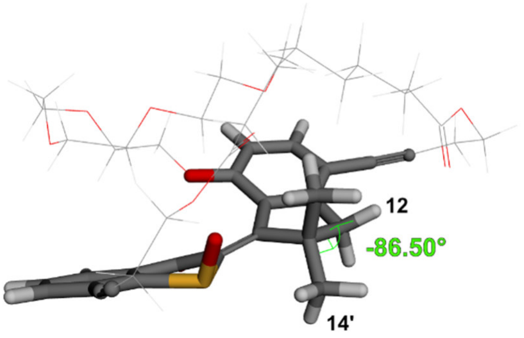

Figure 6 Lowest energy geometry obtained for **C-1** at the B3LYP-D3BJ/6-311G(d,p) IEFPCM (CH_2Cl_2) level of theory. The torsional angle between protons 12 and 14' is close to 90°. The HTI core structure is highlighted for clarity.

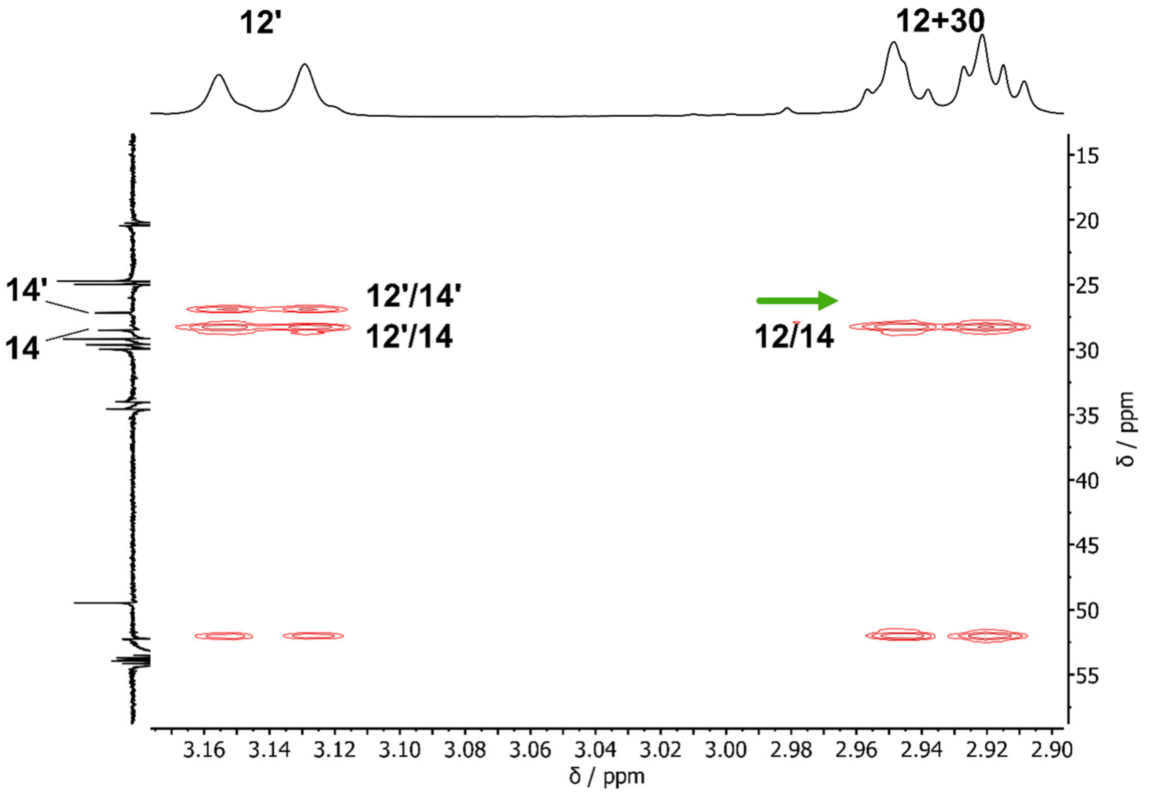

Figure 7 Excerpt from HMBC (600 MHz, CD_2Cl_2, 0 °C) spectrum of **C-1**. There is no correlation between the signals of protons 12 and 14' due to the torsional angle being close to 90°. The spectrum was recorded at 0 °C, as these correlations could not be observed at ambient temperature.

Isomer **A-1**

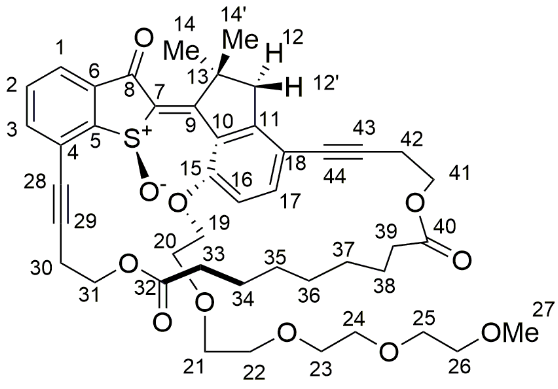

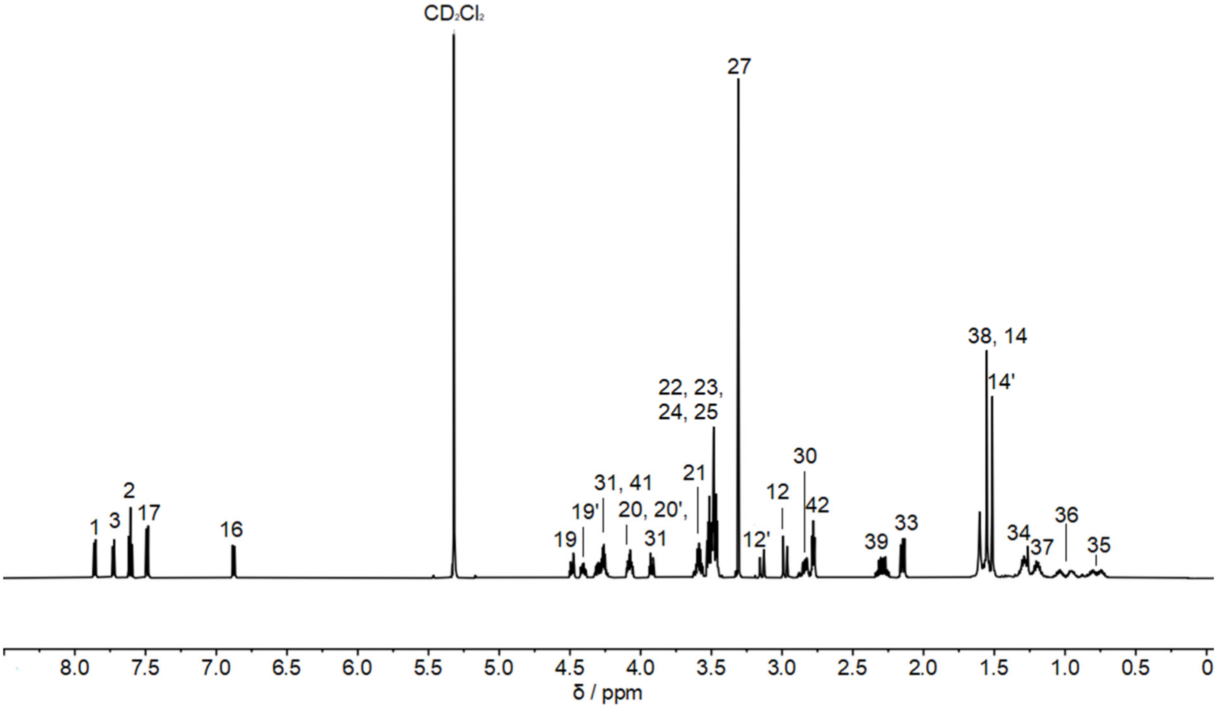

Figure 8 ^{1}H NMR (601 MHz, CD_2Cl_2, 10 °C) spectrum of racemic **A-1** and assignments of the signals to the molecular structure.

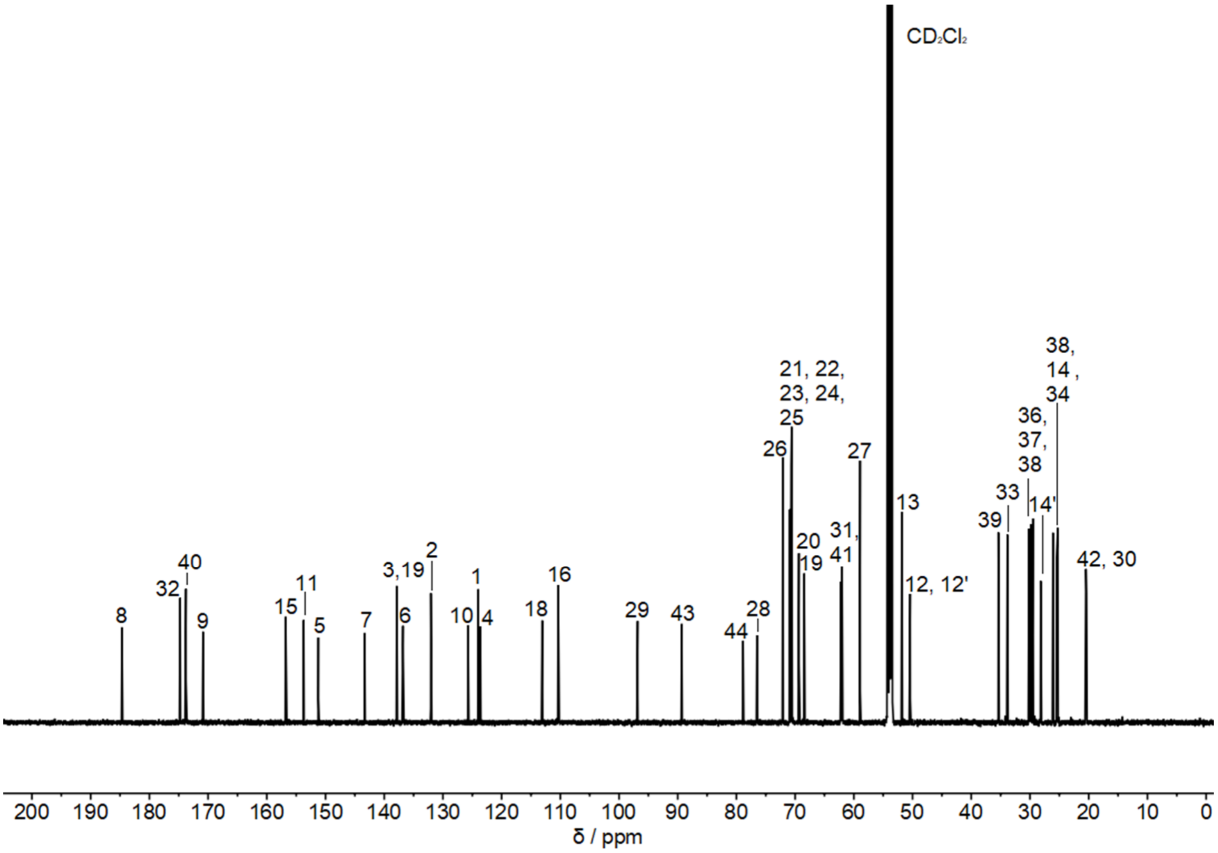

Figure 9 ^{13}C NMR (151 MHz, CD_2Cl_2, 10 °C) spectrum of racemic **A-1** and assignments of the signals to the molecular structure.

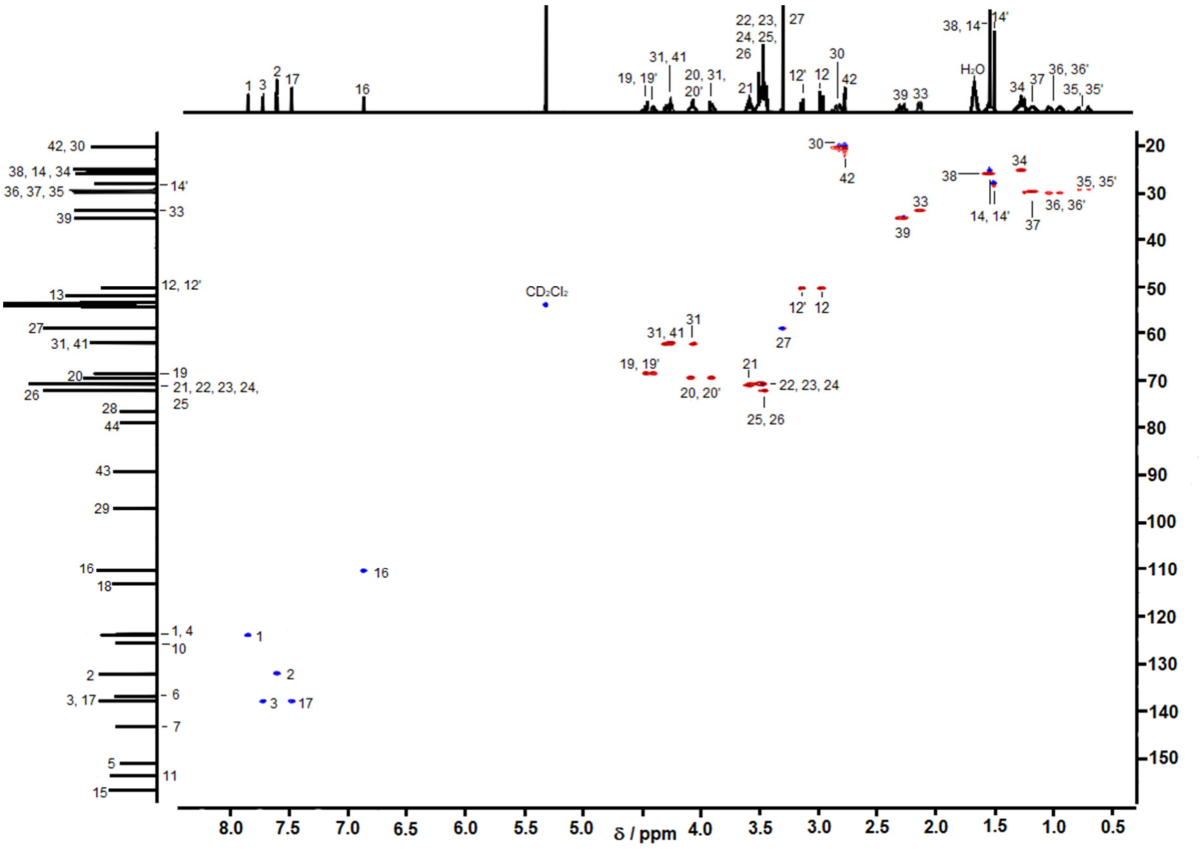

Figure 10 HSQC NMR (601 MHz, CD_2Cl_2, 10 °C) spectrum of racemic **A-1** and assignments of the signals of the molecular structure.

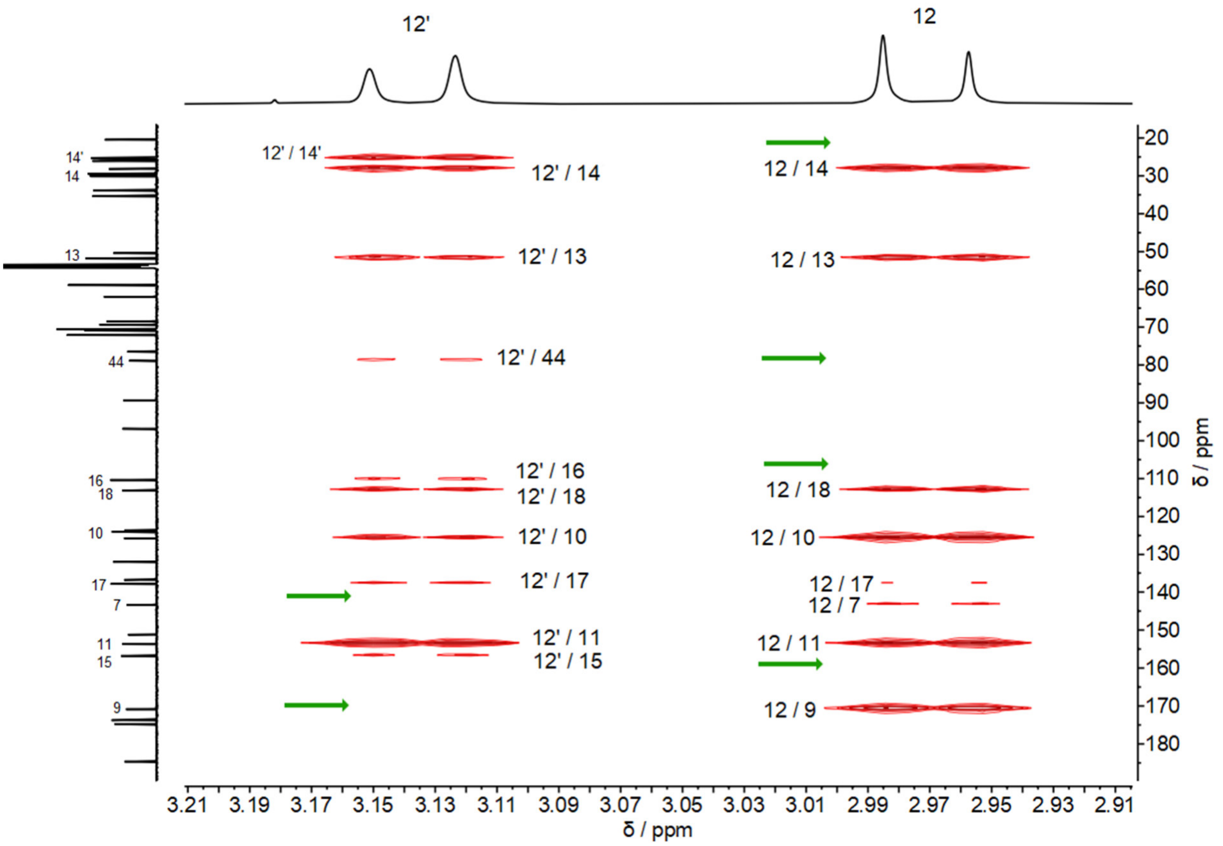

Figure 11 HMBC NMR (601 MHz, CD_2Cl_2, 10 °C) of racemic A-1. There is no correlation observed between proton 12 and carbon atom 14’ due to the torsional angle being close to 90°.

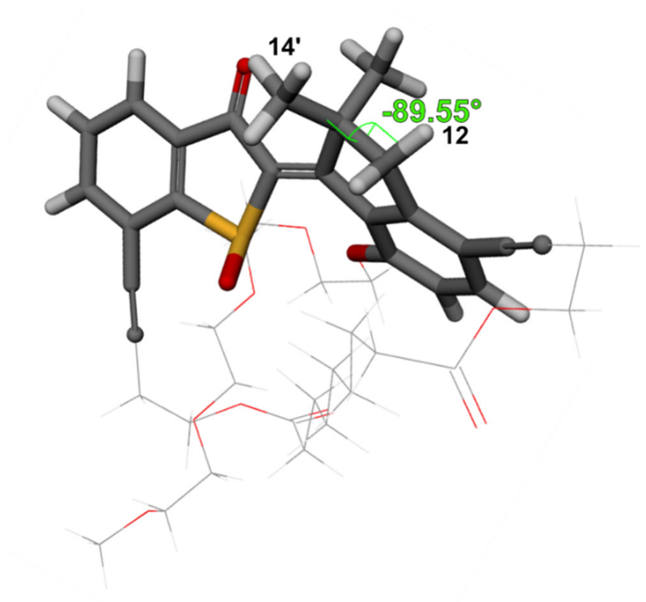

Figure 12 Minimum geometry obtained for **A-1** at the B3LYP-D3BJ/6-311G(d,p) IEFPCM (CH_2Cl_2) level of theory. The HTI core structure is highlighted for clarity. The torsional angle between proton 12 and carbon atom 14' is very close to 90°, which is in agreement with the observed HMBC pattern.

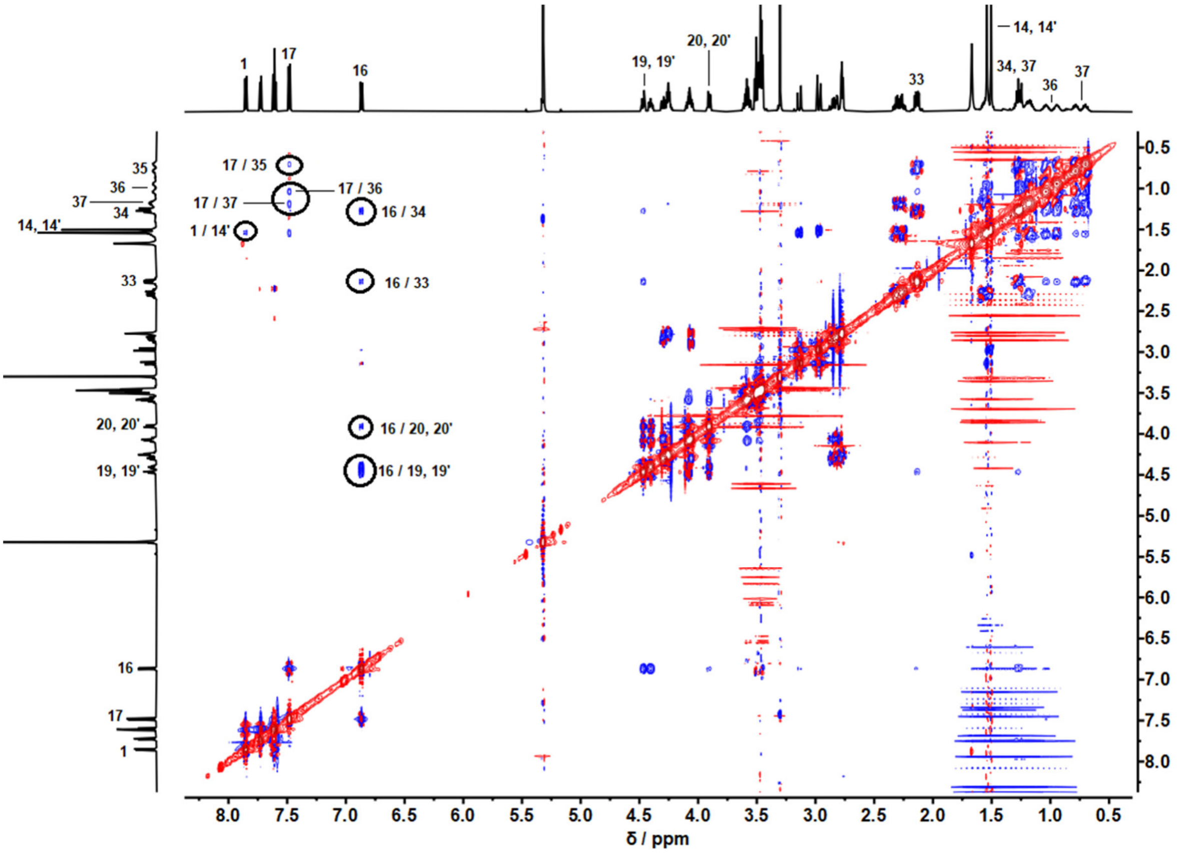

Figure 13 NOESY NMR (601 MHz, CD_2Cl_2, 10 °C) spectrum of racemic **A-1**. The *(Z)* configuration is evidenced by the cross signal of indanone fragment protons 16 and 17 with the aliphatic ring protons (32, 33, 34, 35), as well as cross signal of thioindigo fragment proton 1 with protons 14'.

Metastable Isomer **D-1**

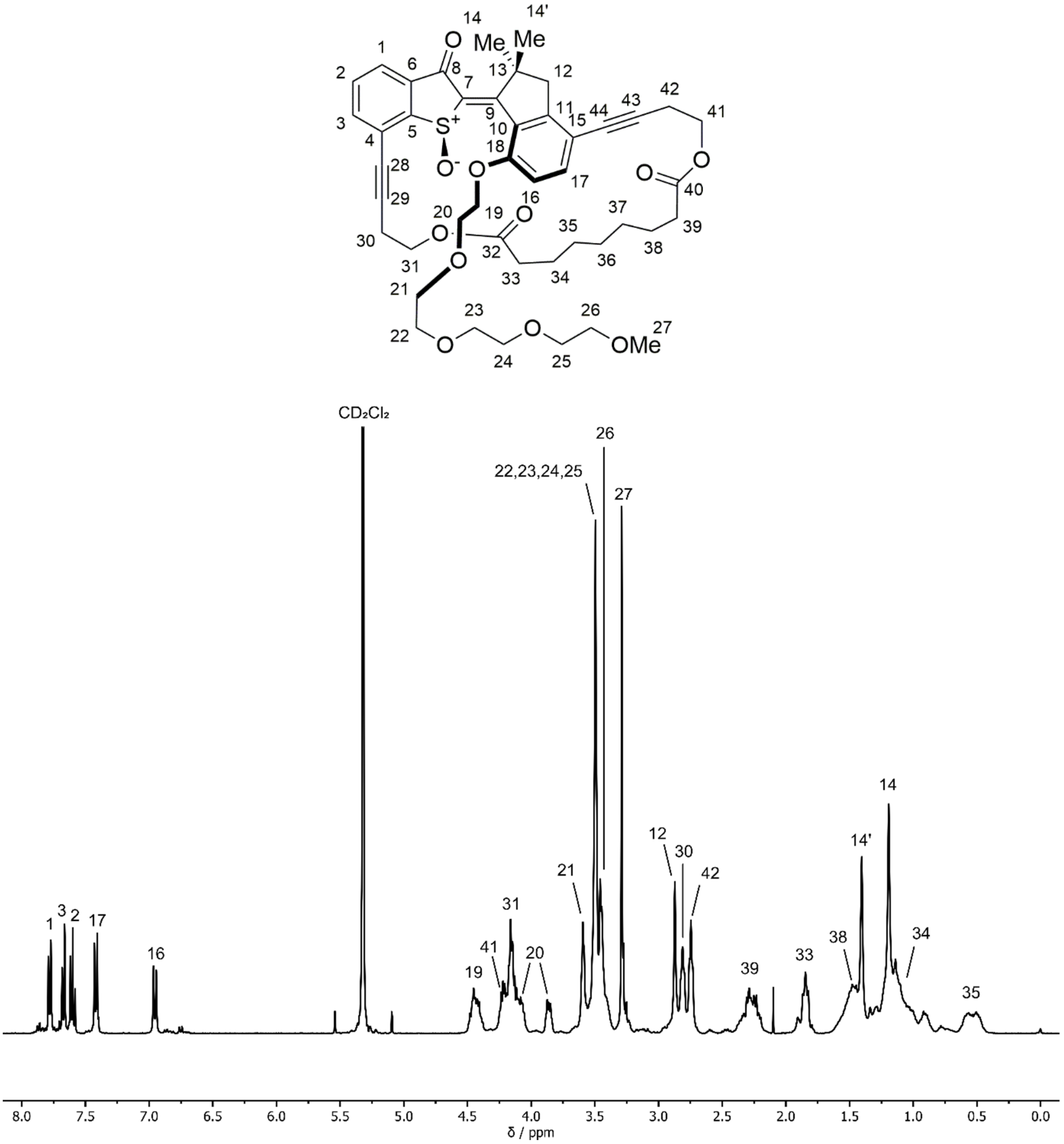

Figure 14 ^{1}H NMR (400 MHz, CD_2Cl_2, −80 °C) of a solution enriched in racemic **D-1** and assignments of the signals to the molecular structure. For preparation, a sample of pure racemic **C-1** in CD_2Cl_2 was irradiated *in situ* with 450 nm light at −80 °C until **C-1** was almost completely converted to **D-1**.

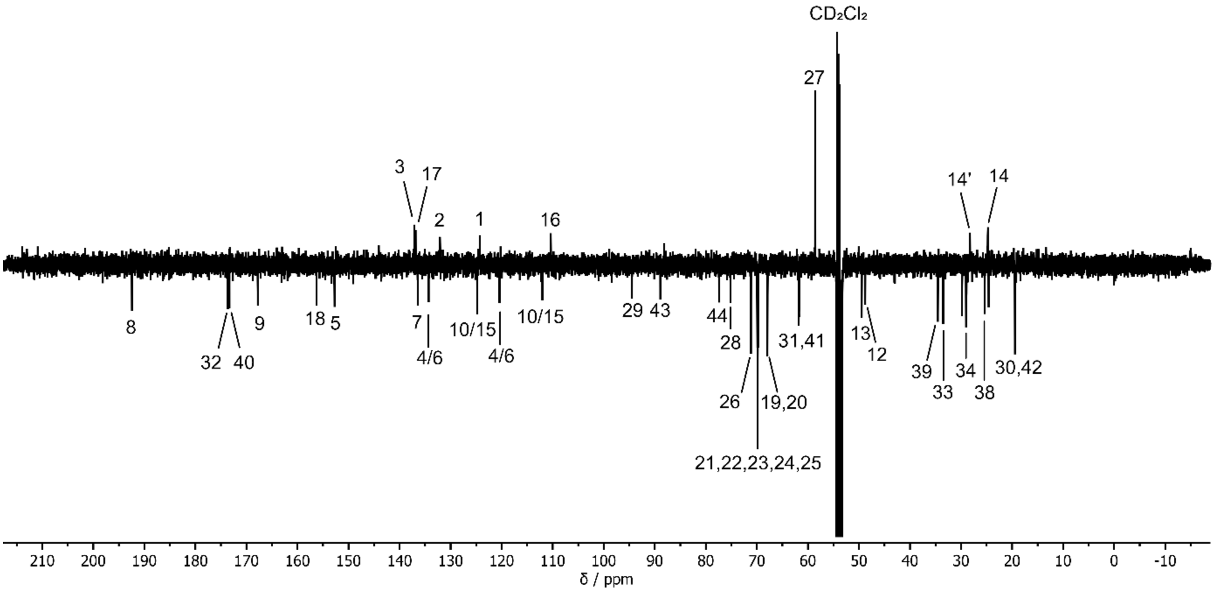

Figure 15 DEPTq NMR (400 MHz, CD_2Cl_2, −80 °C) spectrum of a solution enriched in **D-1** and assignment of the signals to the molecular structure.

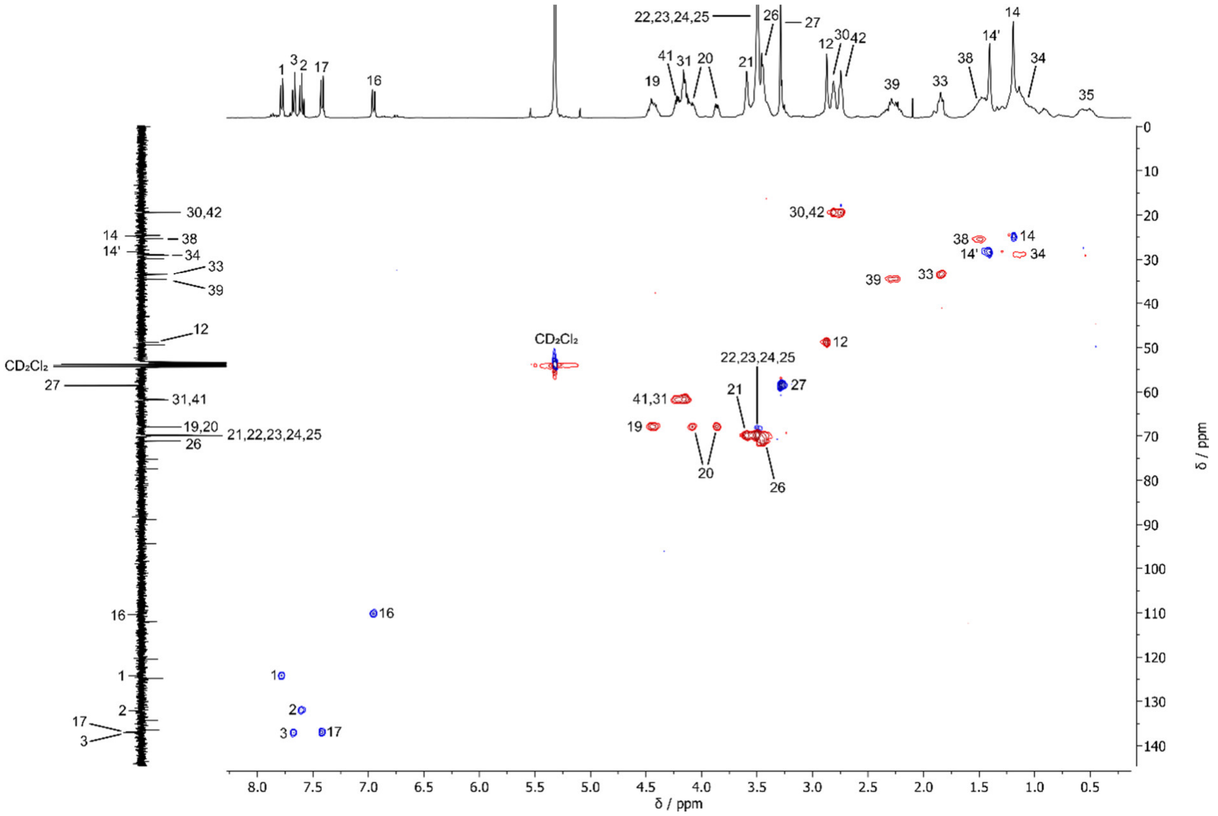

Figure 16 HSQC NMR (400 MHz, DCM-d_2, −80 °C) spectrum of a solution enriched in **D-1** and assignment of the signals to the molecular structure.

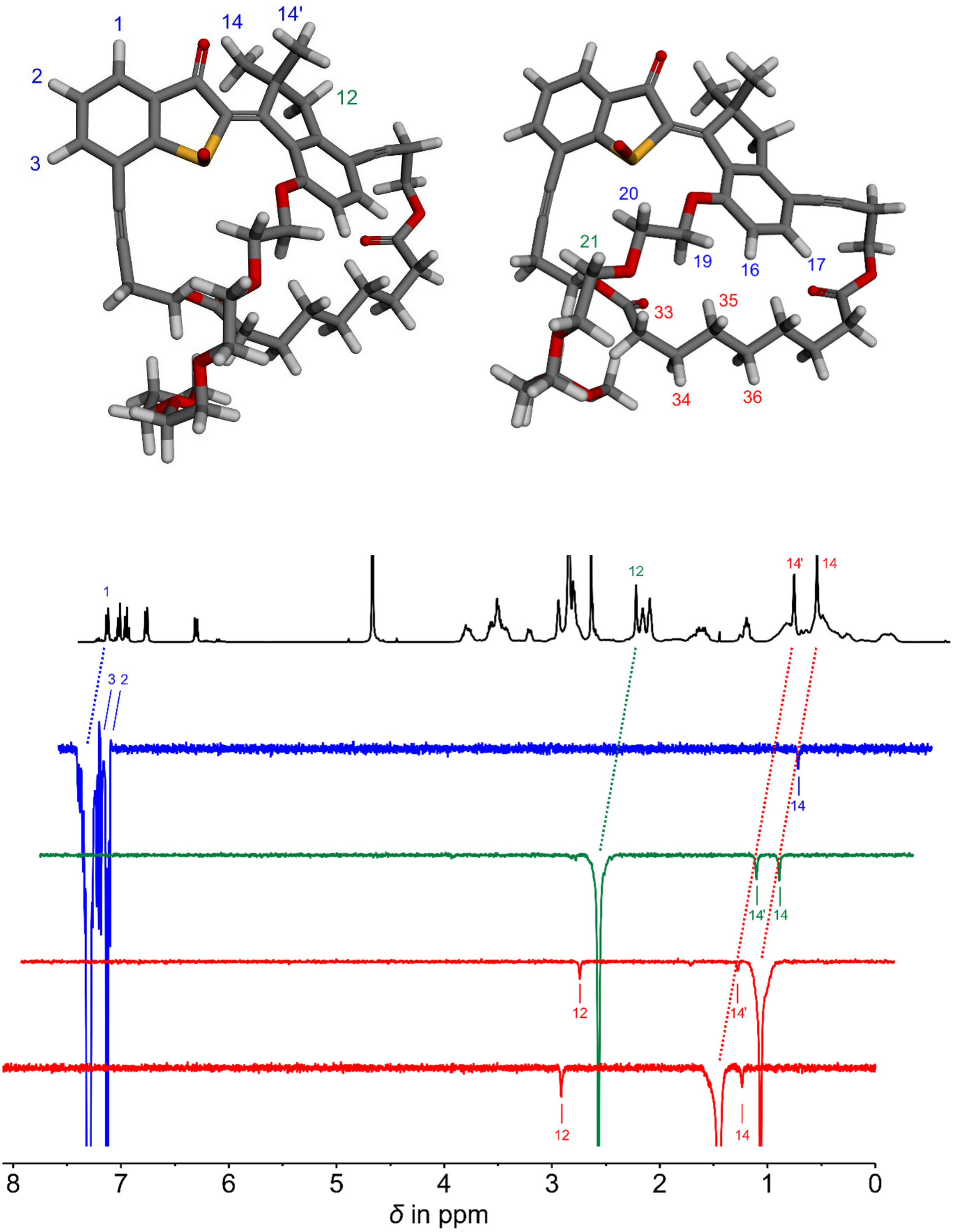

Figure 17 Global minimum structure of **D-1** as calculated on the B3LYP-D3BJ/6-311G(d,p) IEFPCM (CH_2Cl_2) level of theory depicted in two different viewing angles with indicative protons labelled (top). ^{1}H (black) and 1D NOE (coloured) NMR (400 MHz, CD_2Cl_2, −80 °C) spectra of a solution enriched in racemic **D-1** (bottom). Dotted lines indicate, which proton signal was irradiated in the NOE experiment. The (*Z*)-configuration is evidenced by the through-space coupling between proton 1 of the thioindigo fragment and methyl group 14 as predicted by theory.

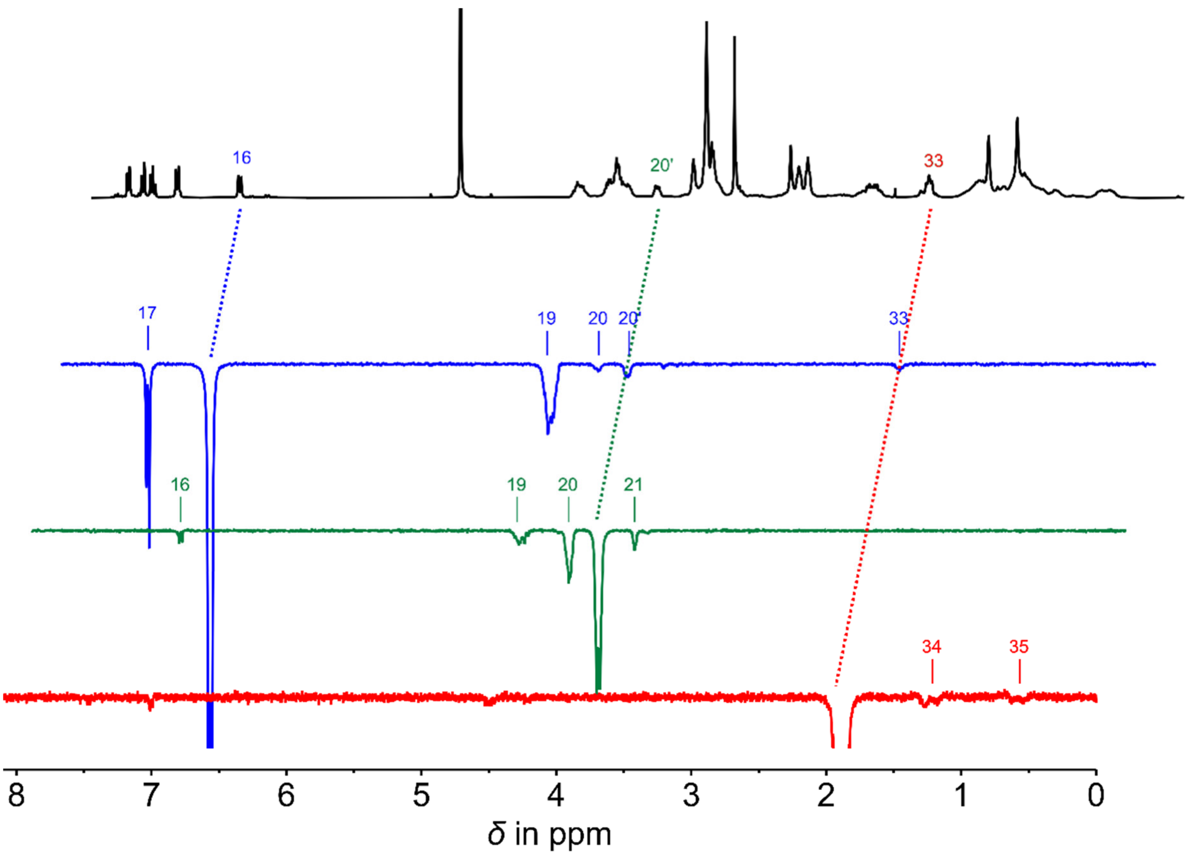

Figure 18 ^{1}H (black) and 1D NOE (coloured) NMR (400 MHz, CD_2Cl_2, −80 °C) spectra of a solution enriched in racemic **D-1**. Dotted lines indicate, which proton signal was irradiated in the NOE experiment. The (*Z*)-configuration is evidenced by the through-space coupling between proton 16 of the indanone fragment and aliphatic ring protons 33. For assignments of indicated signals to the theoretically obtained geometry see **Figure 17** (top).

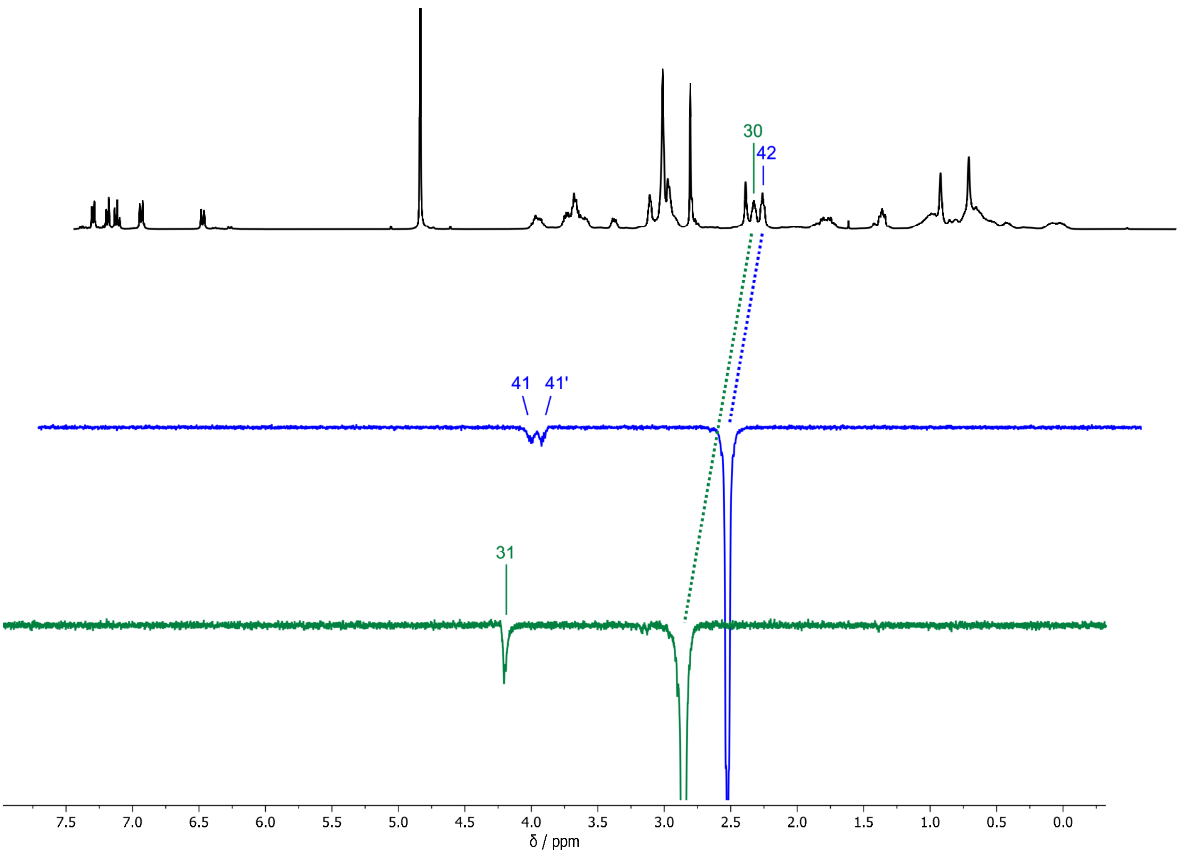

Figure 19 ^{1}H (black) and 1D NOE (coloured) NMR (400 MHz, CD_2Cl_2, −80 °C) spectra of a solution enriched in racemic **D-1**. Dotted lines indicate, which proton signal was irradiated in the NOE experiment. The 1D coupling allows for the resolution of overlapping proton signals 31 and 41.

Isomer **C-2**

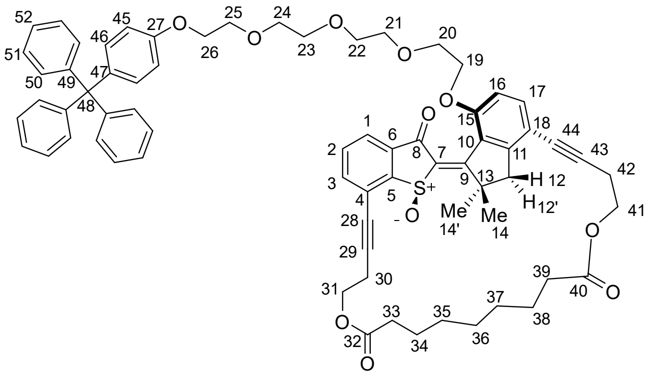

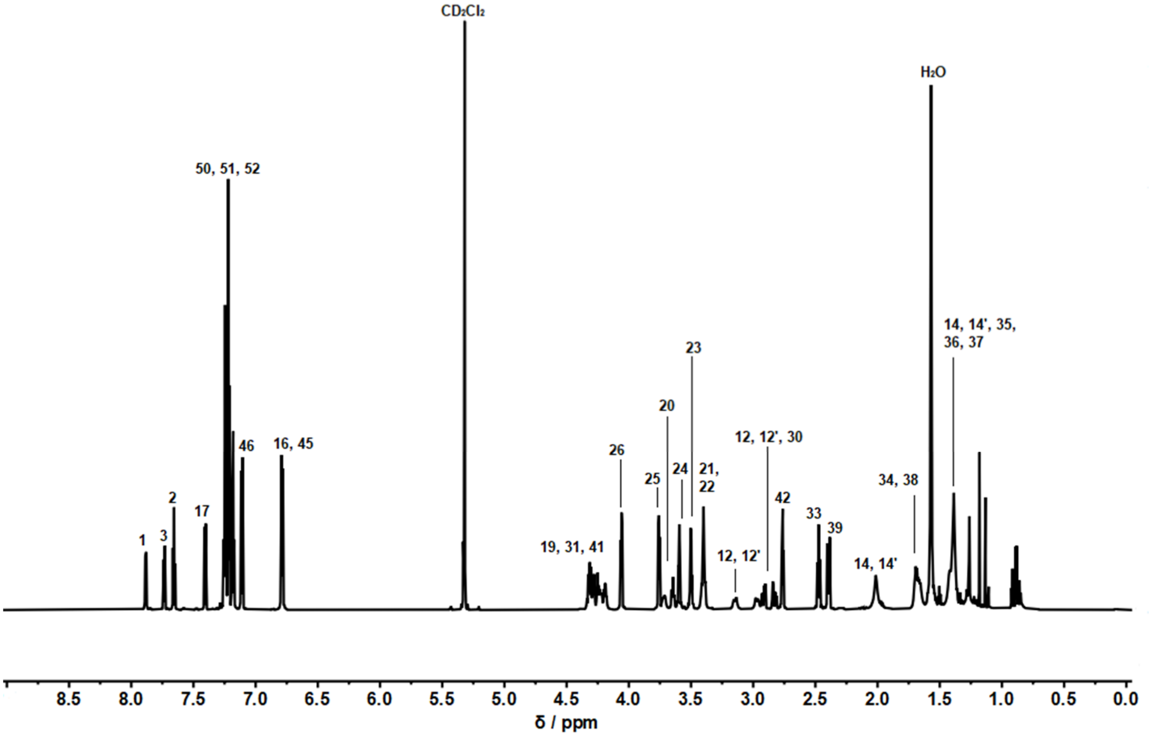

Figure 20 ^{1}H NMR (800 MHz, CD_2Cl_2, 25 °C) spectrum of racemic **C-2** and assignments of the signals to the molecular structure.

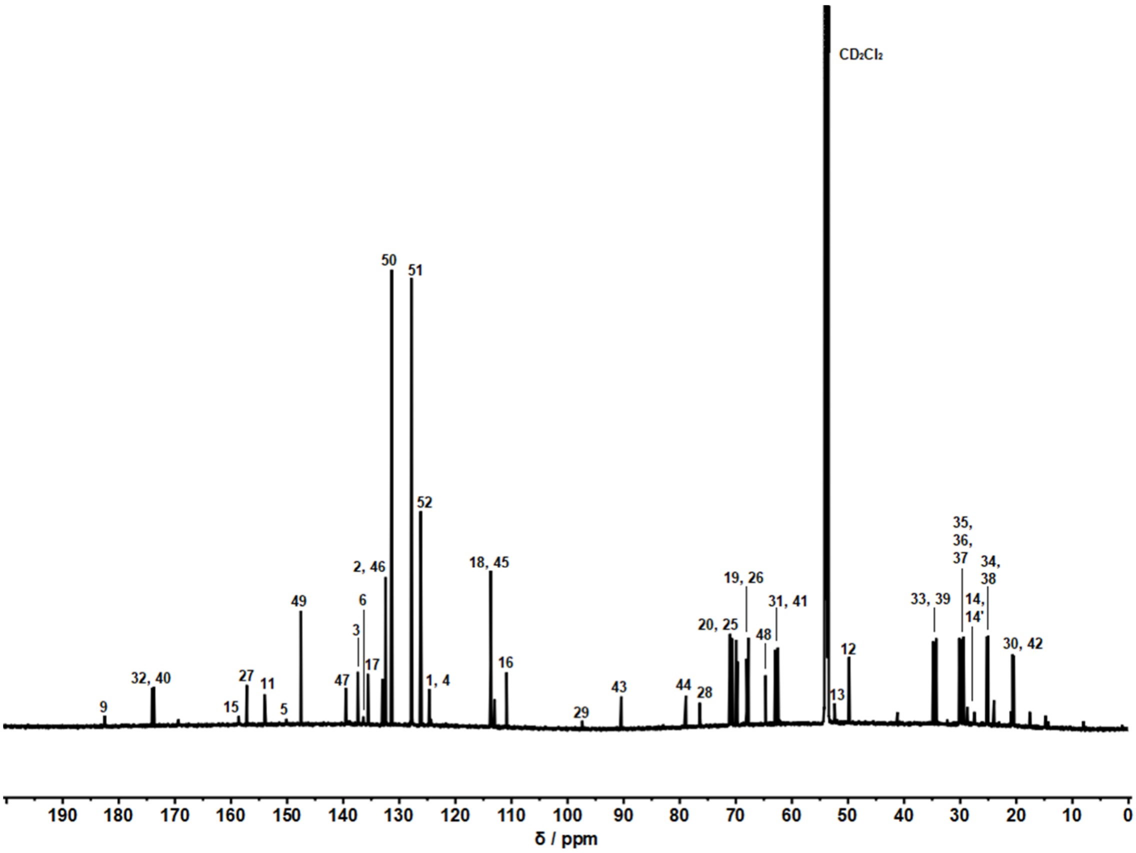

Figure 21 ^{13}C NMR (201 MHz, CD_2Cl_2, 25 °C) spectrum of racemic **C-2** and assignments of signals to the molecular structure.

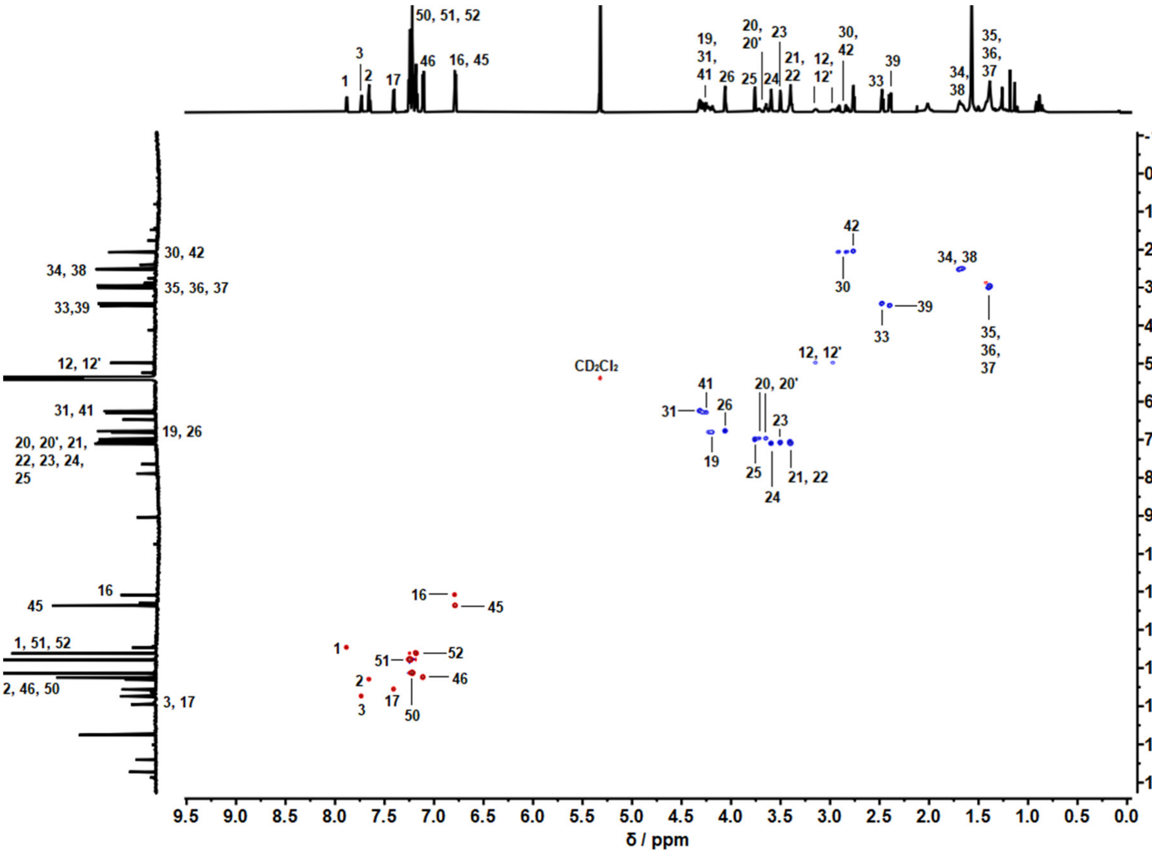

Figure 22 HSQC NMR (CD_2Cl_2, 800 MHz, 25 °C) spectrum of racemic **C-2** and assignments of the signals to the molecular structure.

ISOMER **A-2**

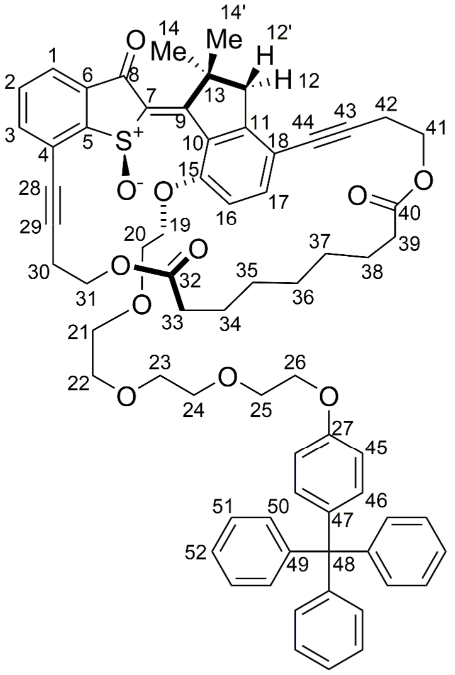

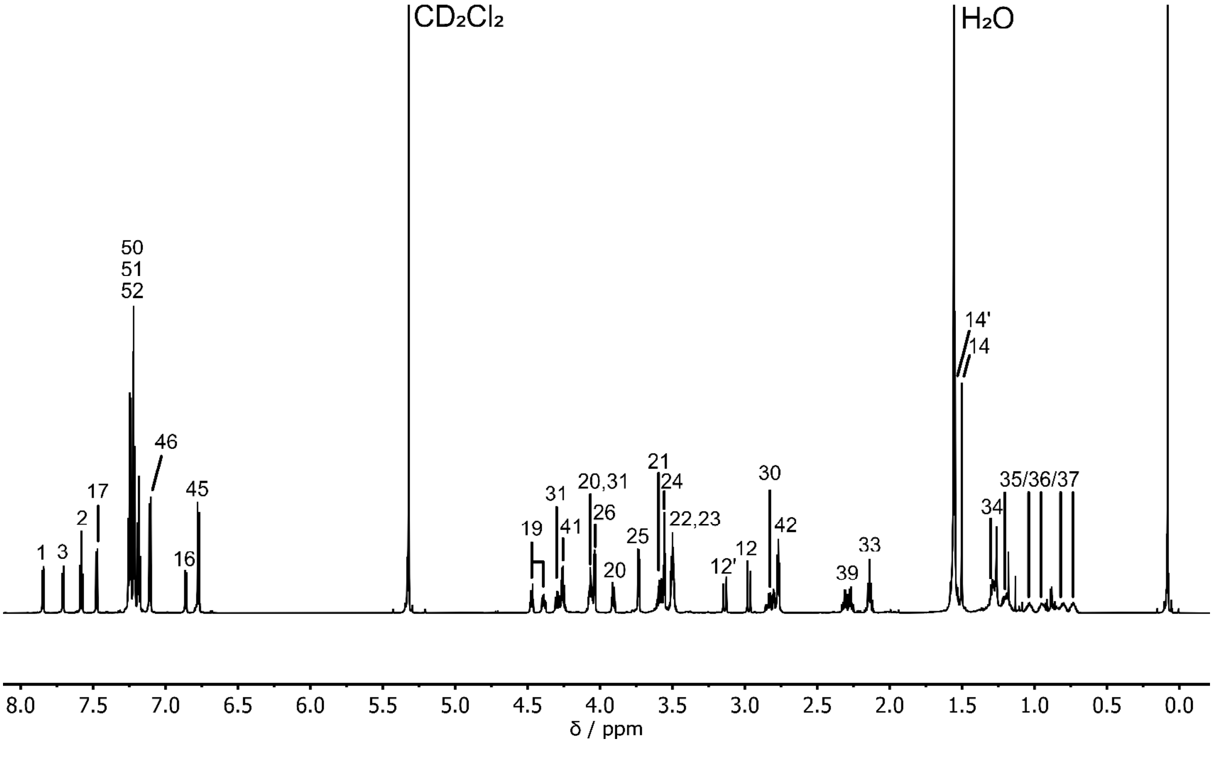

Figure 23 ^{1}H NMR Spectrum (800 MHz, CD_2Cl_2, 25 °C) of racemic **A-2** with assignments of the signals to the molecular structure.

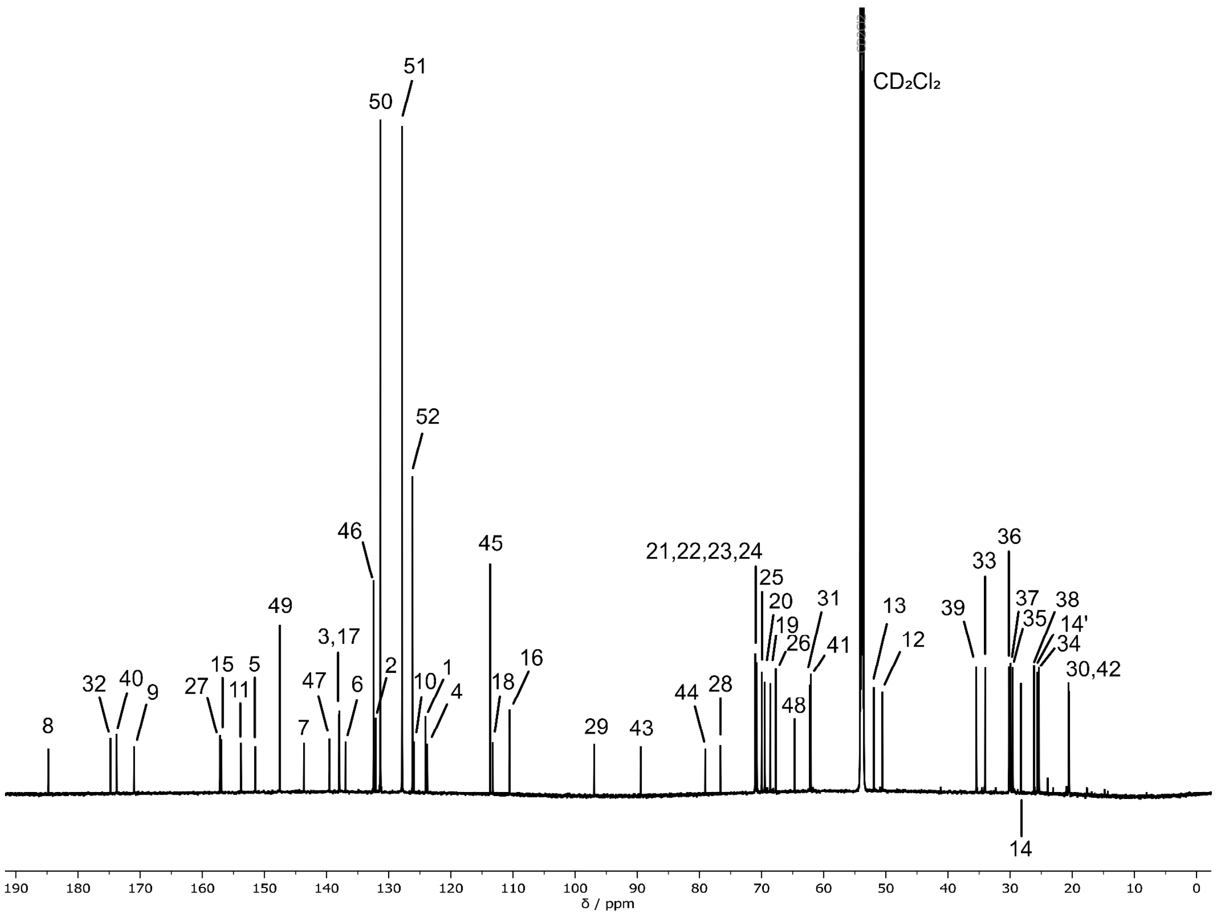

Figure 24 ^{13}C NMR spectrum (201 MHz, CD_2Cl_2, 25 °C) of racemic **A-2** with assignments of the signals to the molecular structure.

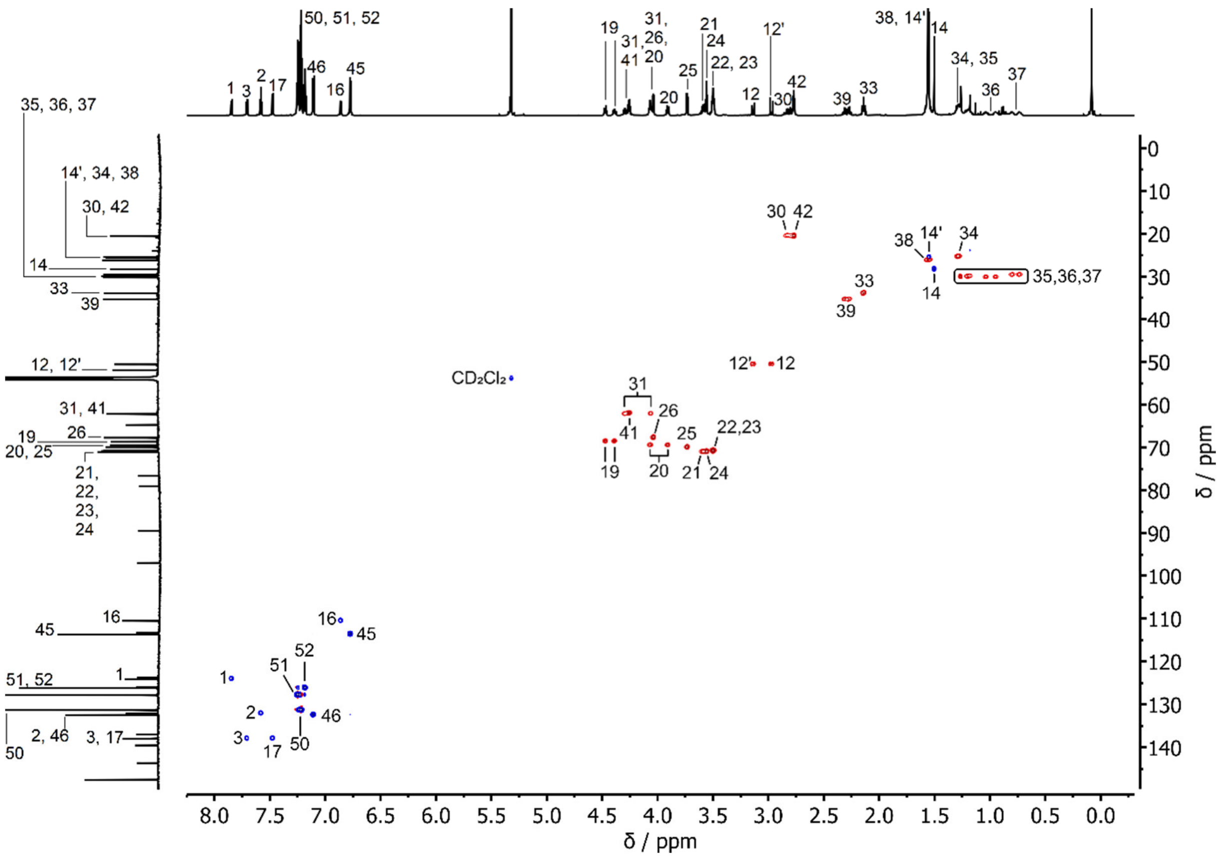

Figure 25 HSQC NMR (800 MHz, CD_2Cl_2, 25 °C) spectrum of racemic **A-2** and assignments of the signals to the molecular structure.

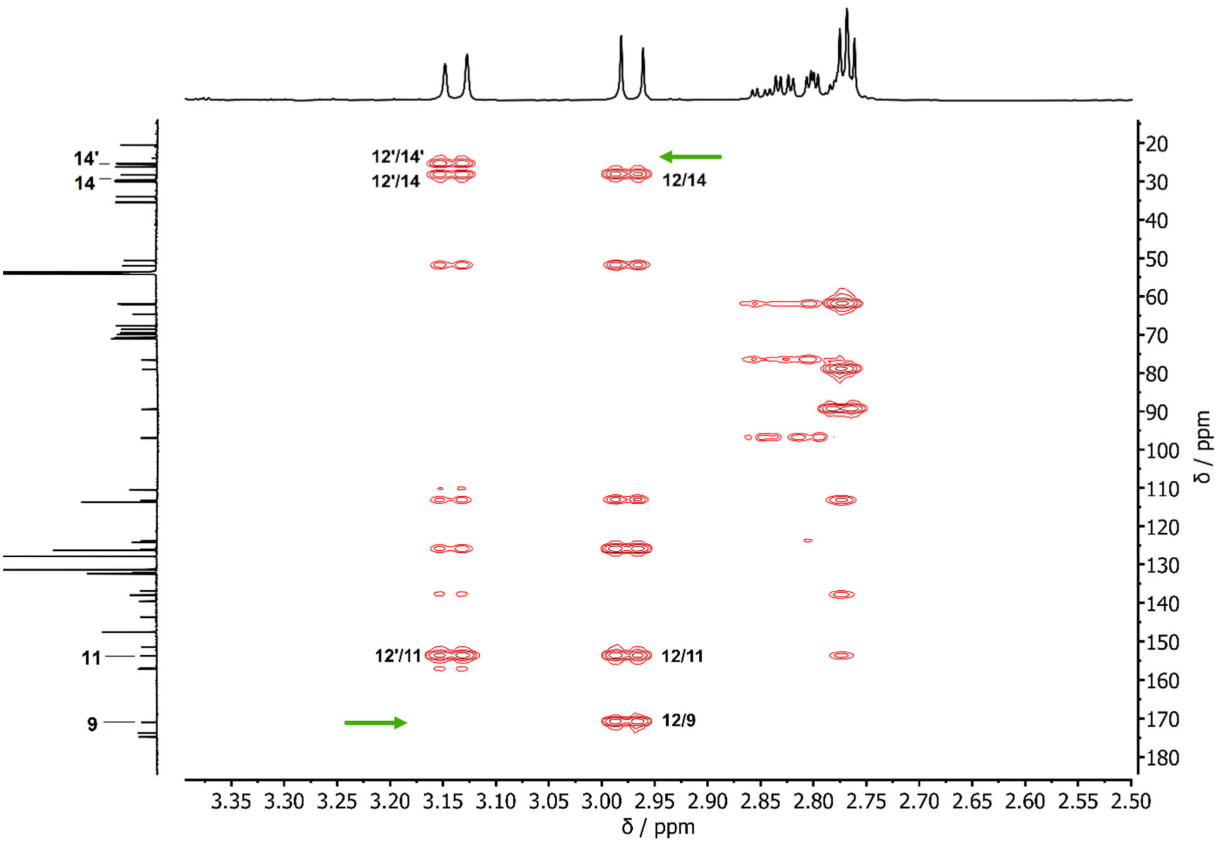

Figure 26 Section of the HMBC (800 MHz, CD_2Cl_2, 25 °C) spectrum of racemic **A-2**. The correlations between proton 12 and carbon atom 14', as well as proton 12' and carbon atom 9, disappear due to the torsional angle being close to 90°.

Isomer **D-2**

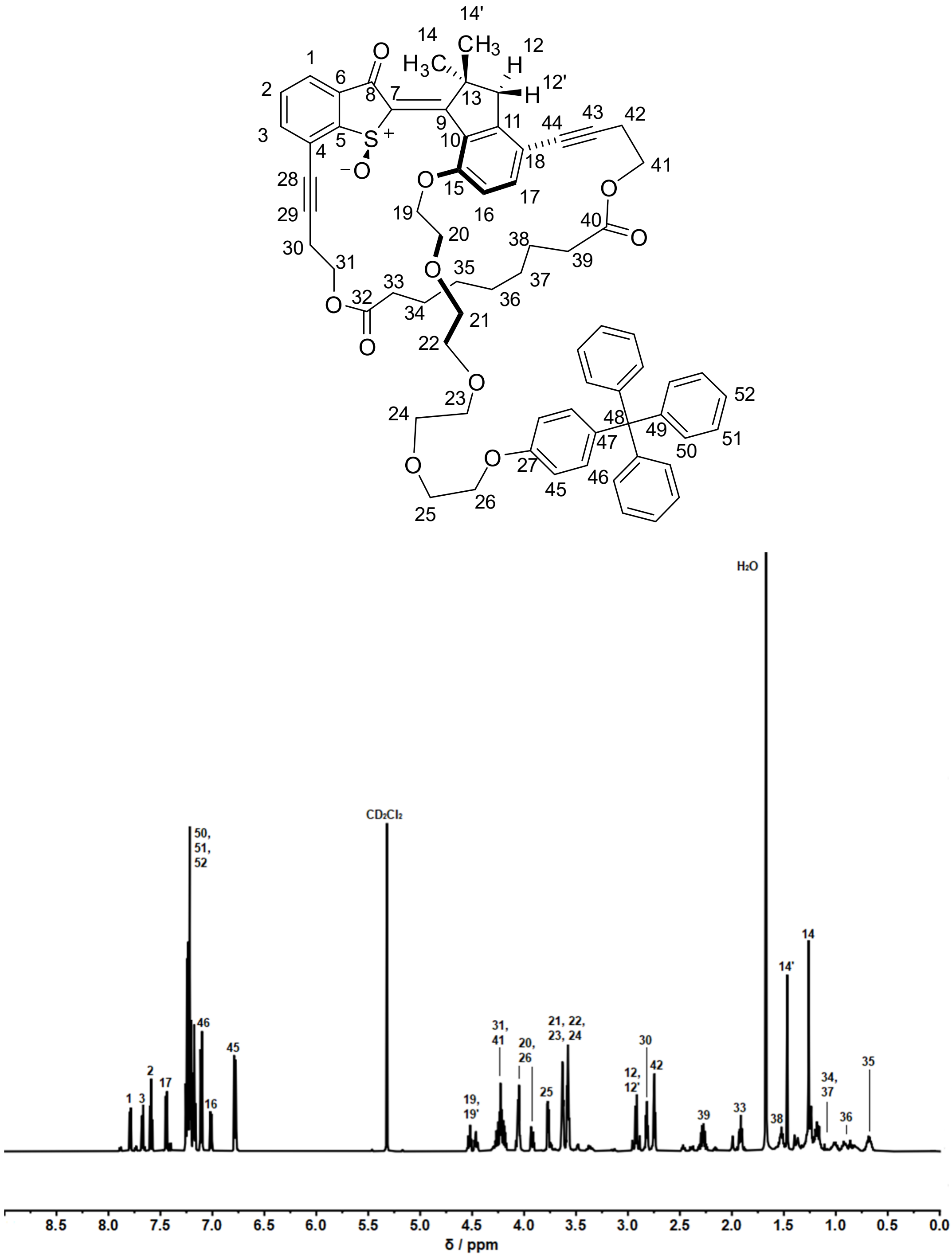

Figure 27 ^{1}H NMR (601 MHz, CD_2Cl_2, 0 °C) spectrum of a mixture enriched in racemic **D-2** and assignments of the signals to the molecular structure. **D-2** was accumulated by external irradiation of a solution of racemic **C-2** in CD_2Cl_2 with 450 nm light at 23 °C.

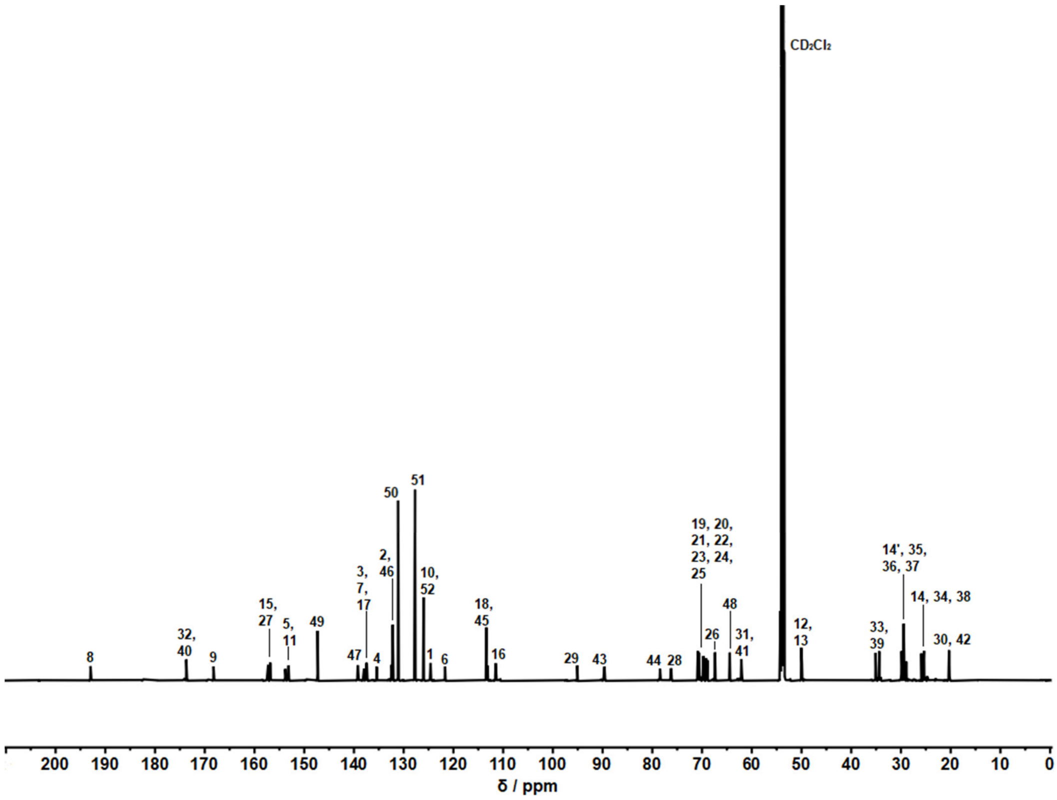

Figure 28 ^{13}C NMR (151 MHz, CD_2Cl_2, 0 °C) spectrum of a solution enriched in racemic **D-2** and assignments of the signals to the molecular structure.

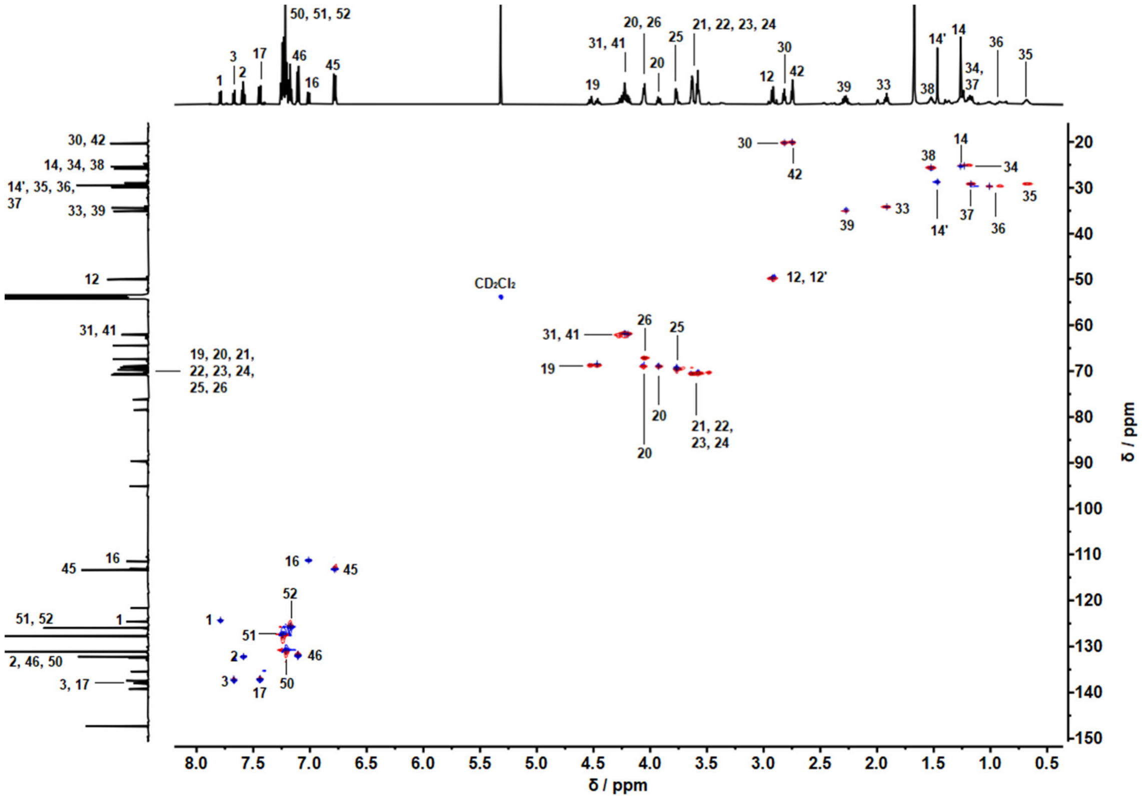

Figure 29 HSQC NMR (601 MHz, CD_2Cl_2, 0 °C) spectrum of a solution enriched in racemic **D-2** and assignments of the signals to the molecular structure.

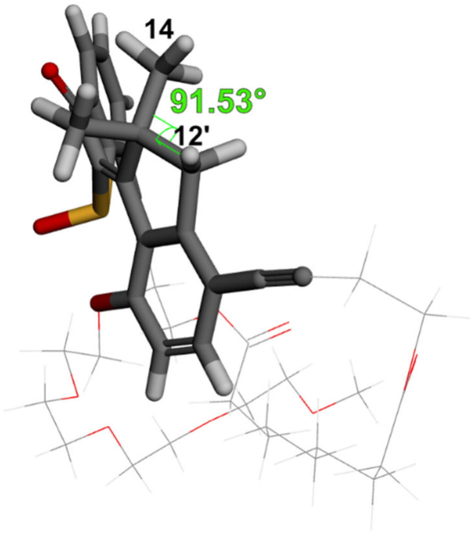

Figure 30 Minimum geometry obtained for **D-1** at the B3LYP-D3BJ/6-311G(d,p) IEFPCM (CH_2Cl_2) level of theory. The HTI core structure is highlighted for clarity. The torsional angle between proton 12’ and carbon atom 14 is close to 90°, which is in agreement with the observed HMBC pattern.

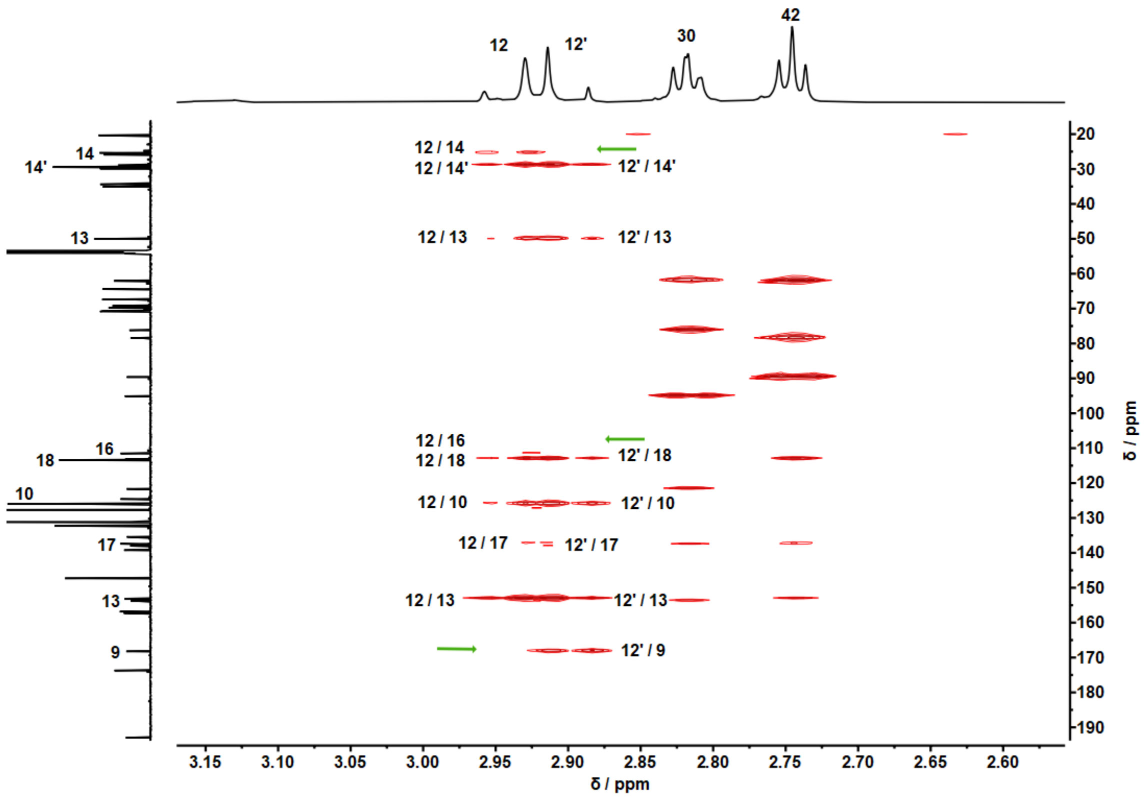

Figure 31 HMBC NMR (601 MHz, CD_2Cl_2, 0 °C) of a solution enriched in **D-2**. No correlation between proton 12’ and carbon atom 14 is observed due to the torsional angle being close to 90°.

Comparison of ^{1}H-NMR spectra of stoppered system 1 and unstoppered system 2

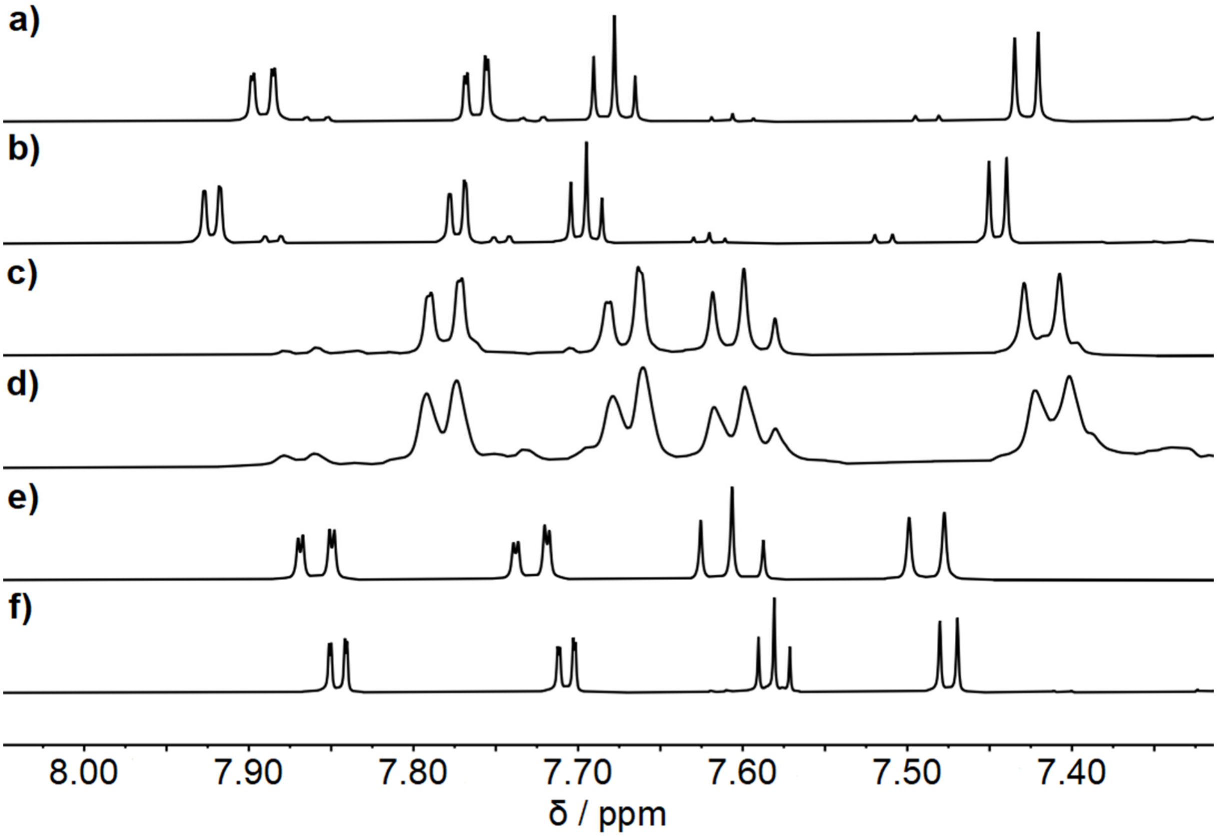

Figure 32 Comparison of the aromatic region of ^{1}H NMR (CD_2Cl_2) spectra. **a) C-1** at 25 °C. **b) C-2** at 25 °C. **c) D-1** at –80 °C. **d) D-2** at –80 °C. **e) A-1** at 25 °C. **f) A-2** at 25 °C.

Elevated Temperature Behavior of Macrocyclic Motors 1 and 2

Thermal double bond isomerization experiments were performed in amberized NMR tubes. Solutions were kept at 80 °C in an oil bath. After the indicated time intervals, solutions were quickly cooled to 0 °C in an ice bath in order to interrupt double bond isomerization and ^{1}H NMR spectra were then recorded at ambient temperature.

The first-order rate constant for the thermal **A-1** to **C-1** and **C-1** to **A-1** isomerization can be obtained from the decay kinetics of **C-1** observed during heating at 80 °C in $(CDCl_2)_2$. The thermal isomerization is a dynamic equilibrium between both isomerization processes and has to be described as in Equation S1:

$$\ln\left(\frac{[\text{C-1}]_0-[\text{C-1}]_{eq}}{[\text{C-1}]_t-[\text{C-1}]_{eq}}\right) = (k_{A\rightarrow C} + k_{C\rightarrow A})t = mt \qquad \text{Equation S1}$$

with

[**C-1**]$_0$ = initial concentration of **C-1**

[**C-1**]$_{eq}$ = concentration of the decaying **C-1** isomer at equilibrium

[**C-1**]$_t$ = concentration of the decaying isomer at a particular point in time

$k_{A\rightarrow C}$ = rate constant of **A** to **C** isomerization

$k_{C\rightarrow A}$ = rate constant of **C** to **A** isomerization

m = slope

t = elapsed time

The slope is received from the ln-plot of the **C** to **A** isomerization according to Equation S1.

The law of mass action must be taken into account and the resulting Equation S2 is as follows:

$$\frac{[\text{A-1}]_{eq}}{[\text{C-1}]_{eq}} = \frac{k_{C\rightarrow A}}{k_{A\rightarrow C}} \qquad \text{Equation S2}$$

with

[**A-1**]$_{eq}$ = concentration of the **A-1** isomer at equilibrium

[**C-1**]$_{eq}$ = concentration of the **C-1** isomer at equilibrium

The rate constant for both isomerization directions can be obtained from the slope (Equations S3 and S4):

$$k_{A\rightarrow C} = \frac{m}{1+\frac{[\text{A-1}]_{eq}}{[\text{C-1}]_{eq}}}$$ Equation S3

$$k_{C\rightarrow A} = \frac{m}{1+\frac{[\text{C-1}]_{eq}}{[\text{A-1}]_{eq}}}$$ Equation S4

Using the *Eyring* Equation S5 the *Gibbs* energy of activation Δ$G^{‡}$ for the thermal isomerization can be calculated for each rate constant:

$$k = \frac{k_B T}{h}\, e^{-\frac{\Delta G^{‡}}{RT}}$$ Equation S5

with

k_B = Boltzmann constant (1.381 × 10^{-23} J K^{-1}

T = temperature in K

h = Planck constant (6.626 × 10^{-34} J s)

R = ideal gas constant (8.314 J $mol^{-1}K^{-1}$)

k = rate constant for both isomerization directions ($k_{A\rightarrow C}$ + $k_{C\rightarrow A}$)

The rearranged Equation S5 and the respective rate constants $k_{A\rightarrow C}$ and $k_{C\rightarrow A}$ can be applied to calculate the *Gibbs* energies of activation Δ$G^{‡}$ for the thermal double bond isomerization processes.

$$\Delta G^{‡} = -RT\,\ln(\frac{kh}{k_B T})$$ Equation S6

The free enthalpy difference between the interconverting isomers ΔG can be calculated from the isomeric ratio at equilibrium according to Equation S7.

$$\Delta G = -RT\ln K = -\,RT\ln\frac{[\text{C-1}]_{eq}}{[\text{A-1}]_{eq}}$$ Equation S7

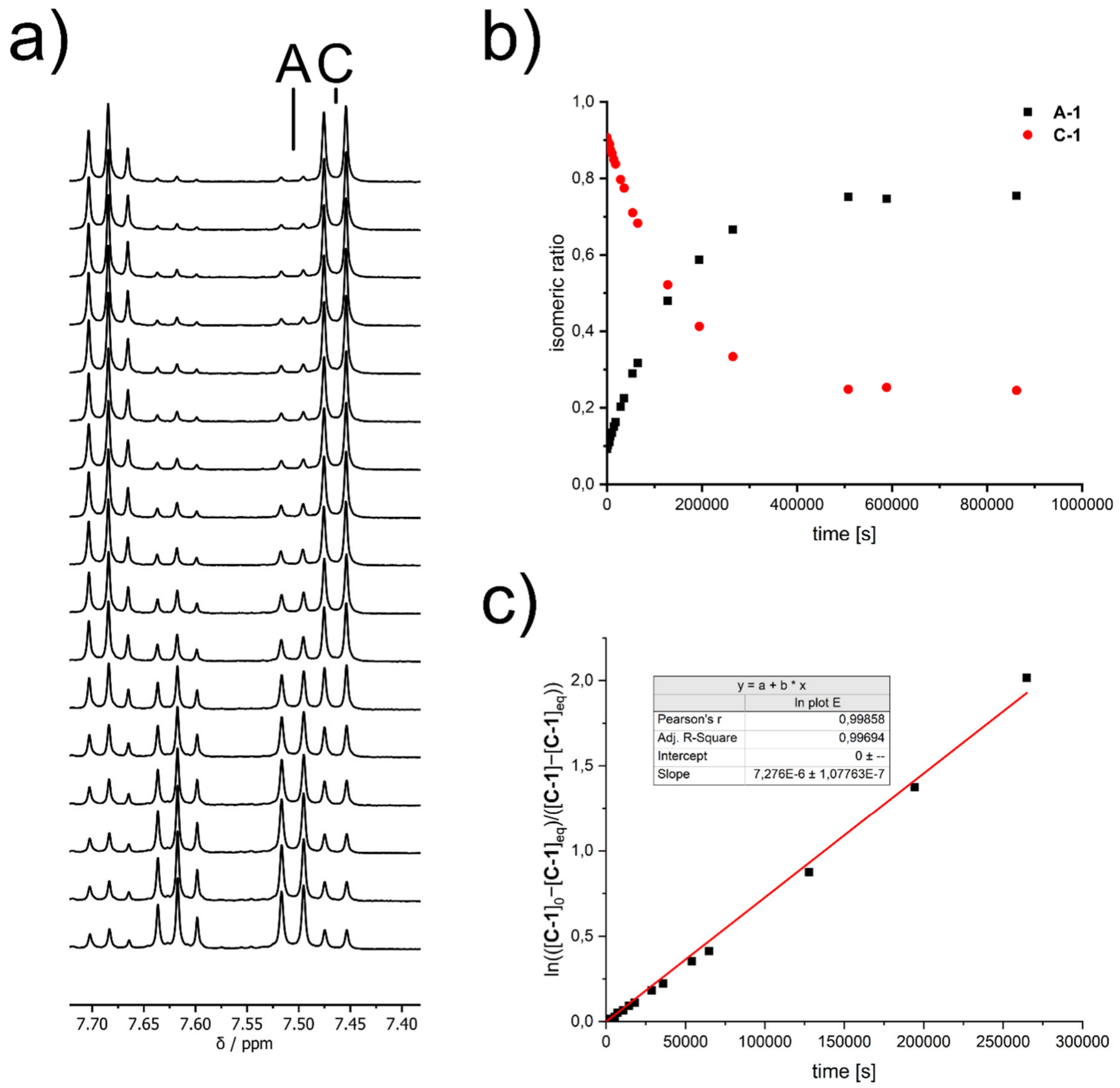

Figure 33 Kinetic analysis of thermal **C-1** to **A-1** double bond isomerization at 80 °C in $(CDCl_2)_2$. **a)** Excerpt of the aromatic region of ^{1}H NMR (400 MHz, 25 °C, $(CDCl_2)_2$) spectra recorded in irregular intervals during double bond isomerization of **C-1**. Proton signals that were used for integration are indicated. **b)** Isomeric ratios of **A-1** and **C-1** changing over time. After heating the mixture at 80 °C for extended time, a constant equilibrium ratio of 75% **A-1** and 25% **C-1** is reached. **c)** First order kinetic analysis taking into account the dynamic equilibrium gives a linear relation. The slope *m* of the linear fit is 7.3×10^{-6} s^{-1}, which can be used to calculate the rate constants for both isomerization directions.

The slope *m* of the linear fit for the thermal **C-1** to **A-1** conversion is 7.3×10^{-6} s^{-1}, which contains the rate constants for both forward and backward isomerization. The resulting *Gibbs* energies of activation $\Delta G^{\ddagger}$ are 30.0 kcal mol^{-1} for **A-1** to **C-1** isomerization, and 29.2 kcal mol^{-1} for **C-1** to **A-1** isomerization at 80 °C.

In thermal equilibrium the relative isomer abundances are 75% **A-1** and 25% **C-1**, which translates into a free enthalpy difference ΔG = 0.8 kcal mol^{-1} at 80 °C, with **A-1** being the thermodynamically more stable isomer.

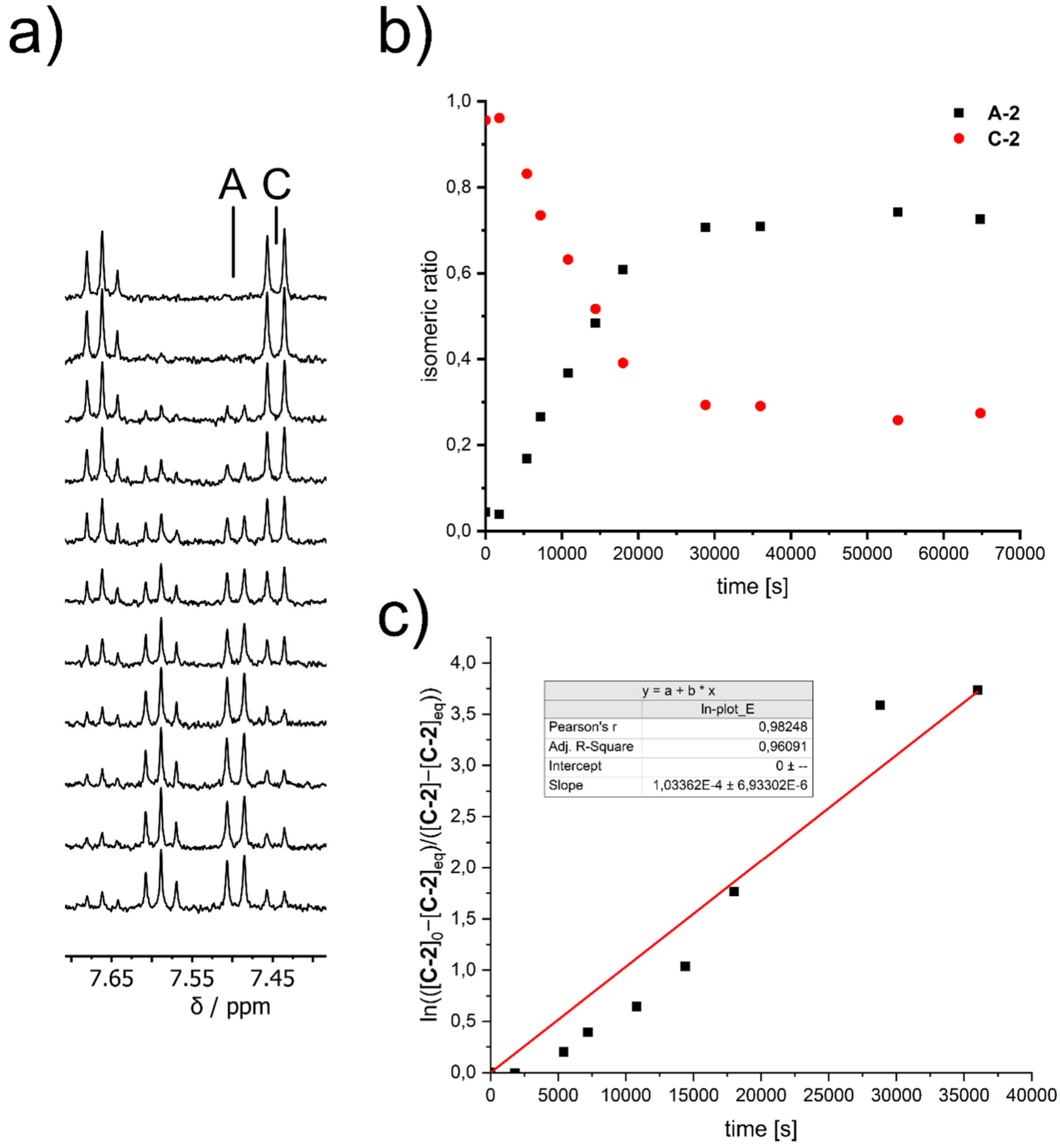

Figure 34 Kinetic analysis of thermal **C-2** to **A-2** double bond isomerization at 80 °C in $(CDCl_2)_2$. **a)** Excerpt of the aromatic region of ^{1}H NMR (400 MHz, 25 °C, $(CDCl_2)_2$) spectra recorded in irregular intervals during double bond isomerization of **C-2**. Proton signals that were used for integration are indicated. **b)** Isomeric ratios of **A-2** and **C-2** changing over time. After heating the mixture at 80 °C for extended time, a constant equilibrium ratio of 73% **A-2** and 27% **C-2** is reached. **c)** First order kinetic analysis taking into account the dynamic equilibrium gives a linear relation. The slope *m* of the linear fit is 1.0×10^{-4} s^{-1}. The corresponding *Gibbs* energies of activation $\Delta G^{\ddagger}$ are 28.1 kcal mol^{-1} for **A-2** to **C-2** and 27.4 kcal mol^{-1} for **C-2** to **A-2** thermal double bond isomerization at 80 °C.

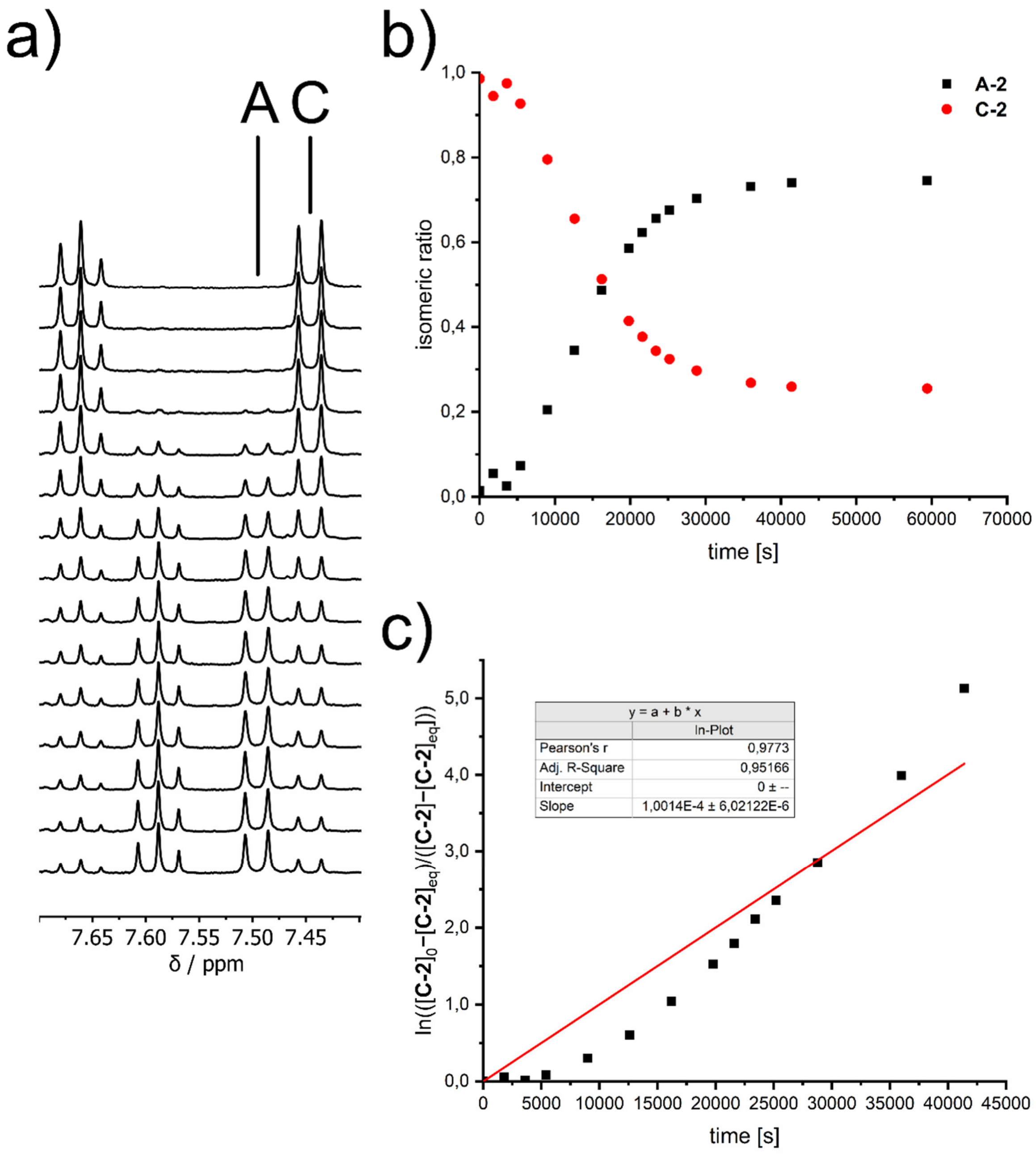

Figure 35 Repeat experiment of thermal **C-2** to **A-2** double bond isomerization at 80 °C in $(CDCl_2)_2$ and kinetic analysis. a) Excerpt of the aromatic region of ^{1}H NMR (400 MHz, 23 °C, $(CDCl_2)_2$) spectra recorded in irregular intervals during double bond isomerization of **C-2**. Proton signals that were used for integration are indicated. b) Isomeric ratios of **A-2** and **C-2** changing over time. After heating the mixture at 80 °C for extended time, a constant equilibrium ratio of 75% **A-2** and 25% **C-2** is reached. c) First order kinetic analysis taking into account the dynamic equilibrium gives a linear relation. The slope m of the linear fit is 1.0×10^{-4} s^{-1}. The corresponding *Gibbs* energies of activation $\Delta G^{\ddagger}$ are 28.2 kcal mol^{-1} for **A-2** to **C-2** and 27.4 kcal mol^{-1} for **C-2** to **A-2** thermal double bond isomerization at 80 °C.

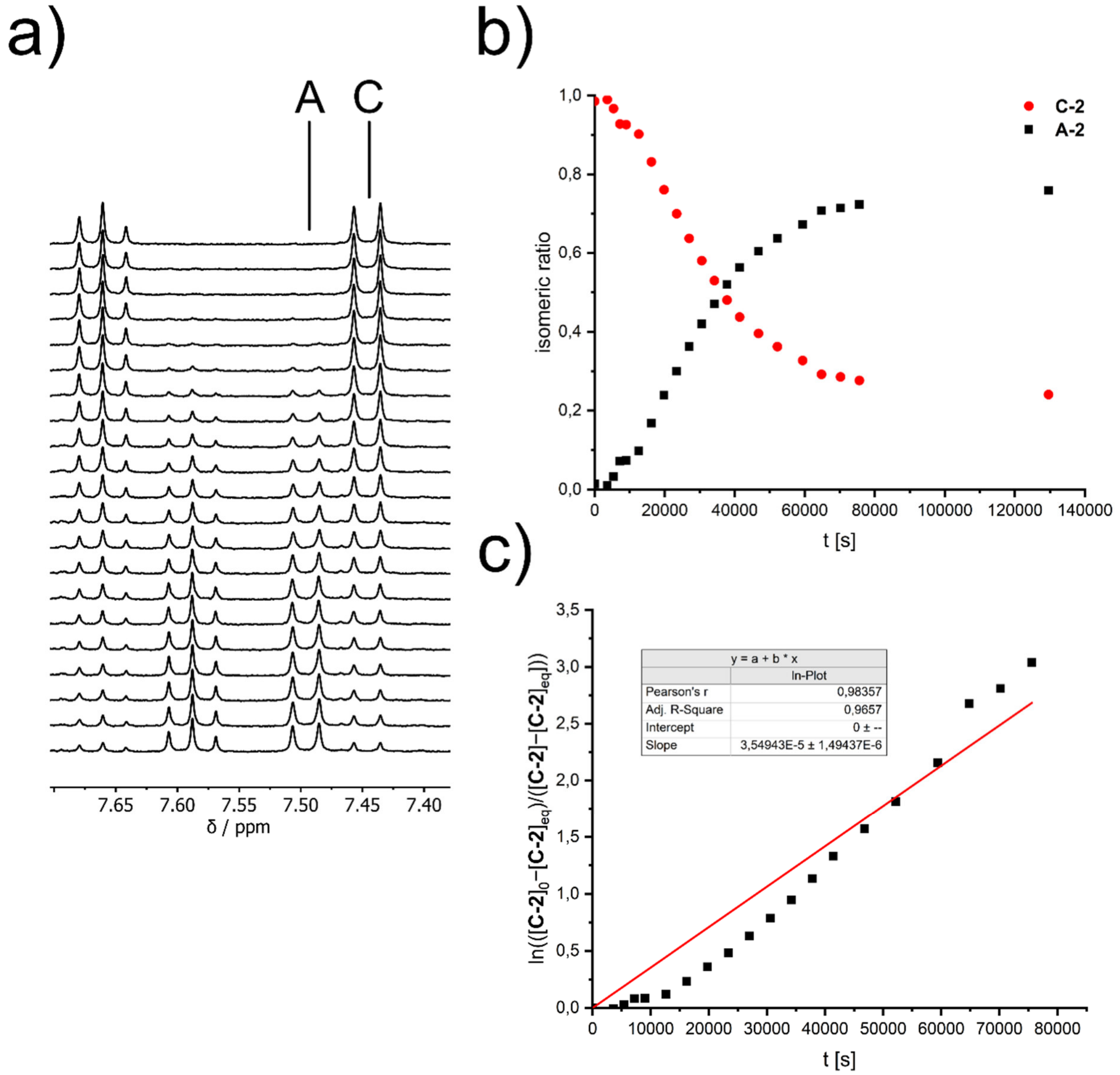

Figure 36 Repeat experiment of thermal **C-2** to **A-2** double bond isomerization at 80 °C in $(CDCl_2)_2$ and kinetic analysis. a) Excerpt of the aromatic region of ^{1}H NMR (400 MHz, 23 °C, $(CDCl_2)_2$) spectra recorded in irregular intervals during double bond isomerization of **C-2**. Proton signals that were used for integration are indicated. b) Isomeric ratios of **A-2** and **C-2** changing over time. After heating the mixture at 80 °C for extended time, a constant equilibrium ratio of 74% **A-2** and 26% **C-2** is reached. c) First order kinetic analysis taking into account the dynamic equilibrium gives a linear relation. The slope *m* of the linear fit is 3.5 × 10^{-5} s^{-1}. The corresponding *Gibbs* energies of activation $\Delta G^{\ddagger}$ are 28.9 kcal mol^{-1} for **A-2** to **C-2** and 28.2 kcal mol^{-1} for **C-2** to **A-2** thermal double bond isomerization at 80 °C.

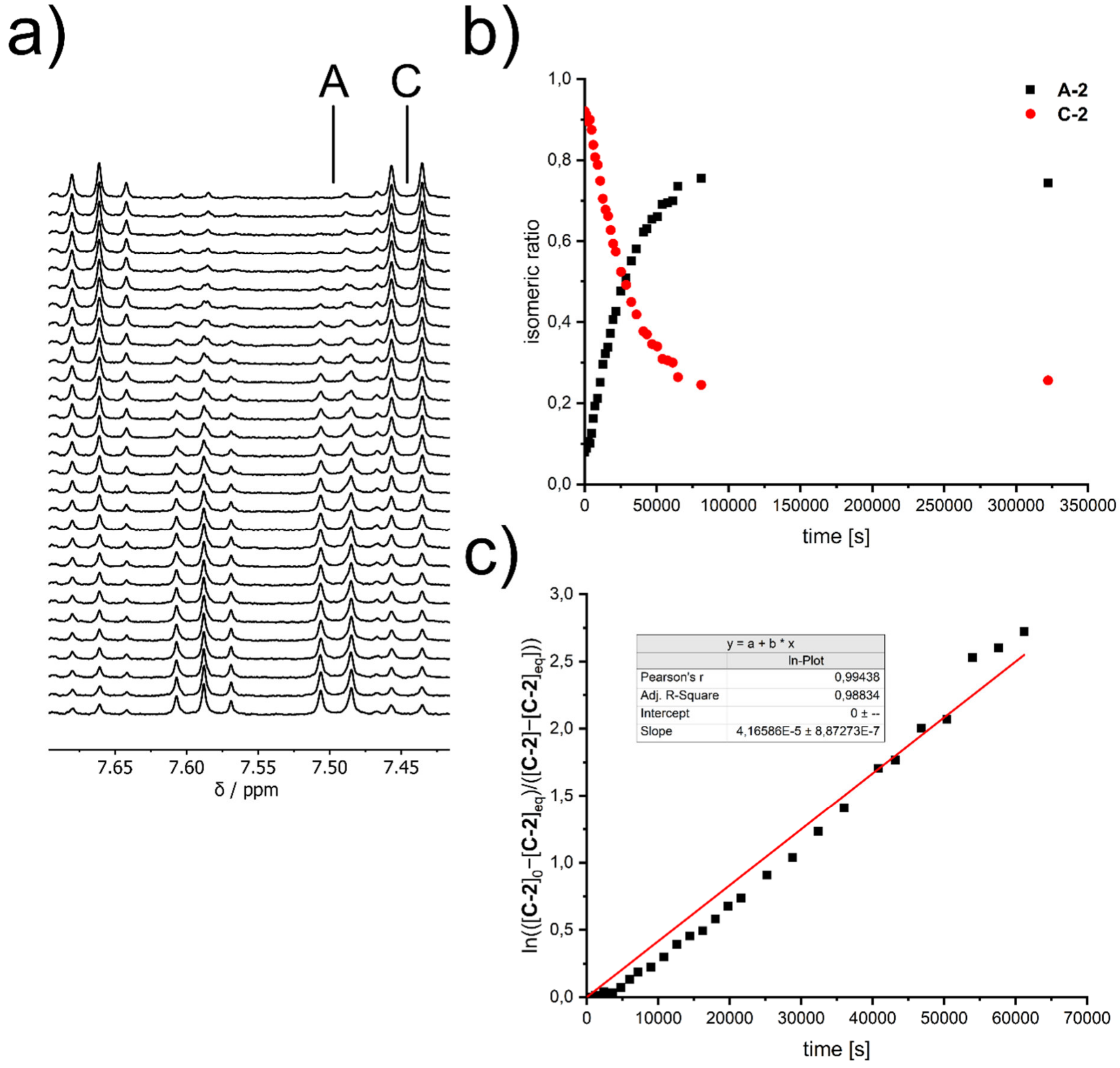

Figure 37 Repeat experiment of thermal **C-2** to **A-2** double bond isomerization at 80 °C in $(CDCl_2)_2$ and kinetic analysis. a) Excerpt of the aromatic region of ^{1}H NMR (400 MHz, 23 °C, $(CDCl_2)_2$) spectra recorded in irregular intervals during double bond isomerization of **C-2**. Proton signals that were used for integration are indicated. b) Isomeric ratios of **A-2** and **C-2** changing over time. After heating the mixture at 80 °C for extended time, a constant equilibrium ratio of 74% **A-2** and 26% **C-2** is reached. c) First order kinetic analysis taking into account the dynamic equilibrium gives a linear relation. The slope *m* of the linear fit is 4.2×10^{-5} s^{-1}. The corresponding *Gibbs* energies of activation $\Delta G^{‡}$ are 28.8 kcal mol^{-1} for **A-2** to **C-2** and 28.0 kcal mol^{-1} for **C-2** to **A-2** thermal double bond isomerization at 80 °C.

Table 1 Summary of results obtained from **C-2** to **A-2** isomerization experiments in $(CDCl_2)_2$ at 80 °C.

Entry	Slope *m*	Equilibrium ratio	ΔG	$\Delta G^{‡}$ A→C	$\Delta G^{‡}$ C→A
	s^{-1}	**A-2**:**C-2**	kcal mol^{-1}	kcal mol^{-1}	kcal mol^{-1}
1	1.0×10^{-4}	73:27	0.7	28.1	27.4
2	1.0×10^{-4}	75:25	0.8	28.2	27.4
3	3.5×10^{-5}	74:26	0.8	28.9	28.2
4	4.2×10^{-5}	74:26	0.8	28.8	28.0
average	6.9×10^{-5}	74:26	(0.8±0.1)	(28.4±0.4)	(27.7±0.4)

The average slope *m* of the linear fit for the thermal **C-2** to **A-2** isomerization at 80 °C is 6.9×10^{-5} s^{-1}, which contains the rate constants for both forward and backward isomerization. The resulting *Gibbs* energies of activation $\Delta G^{\ddagger}$ are 28.4 kcal mol^{-1} for **A-2** to **C-2** isomerization, and 27.7 kcal mol^{-1} for **C-2** to **A-2** isomerization at 80 °C.

It should be noted that in the last two repeat experiments the thermal **C-2** to **A-2** isomerization is significantly slower. At present we can only speculate but the reason for this lag seems to be related to the residual water content in solution. We observed slowed down isomerization in cases, where residual water content was high due to prolonged storage of samples in the freezer. However, when water content decreased over the course of the heating experiment, the isomerization was accelerated.

In the thermal equilibrium the relative isomer abundances are 74% **A-2** and 26% **C-2**, which translates into a free enthalpy difference ΔG = 0.8 kcal mol^{-1} at 80 °C, with **A-2** being the thermodynamically more stable isomer.

Motor Function Elucidation of HTI 1

Comparison Between Ambient and Low Temperature ^{1}H NMR Spectra

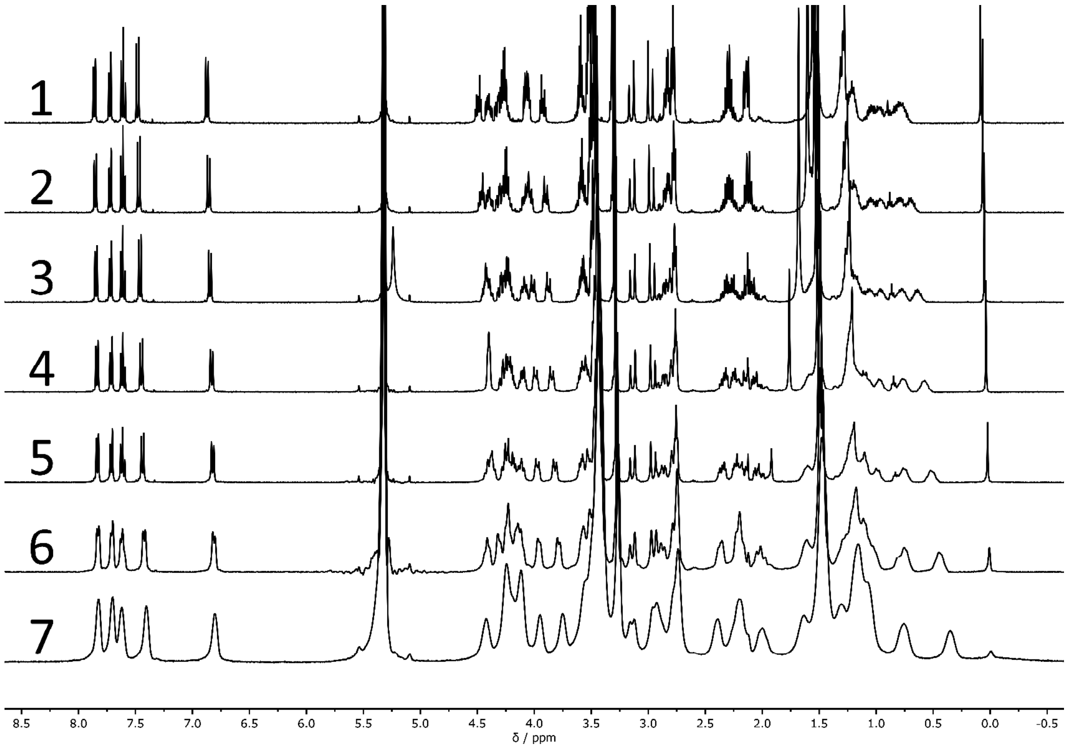

Figure 38 Variable temperature ^{1}H NMR (400 MHz, CD_2Cl_2/CS_2 4/1) spectra of a solution of racemic **A-1** acquired at 1) 25 °C 2) 0 °C 3) −20 °C 4) −40 °C 5) −60 °C 6) −80 °C 7) −105 °C.

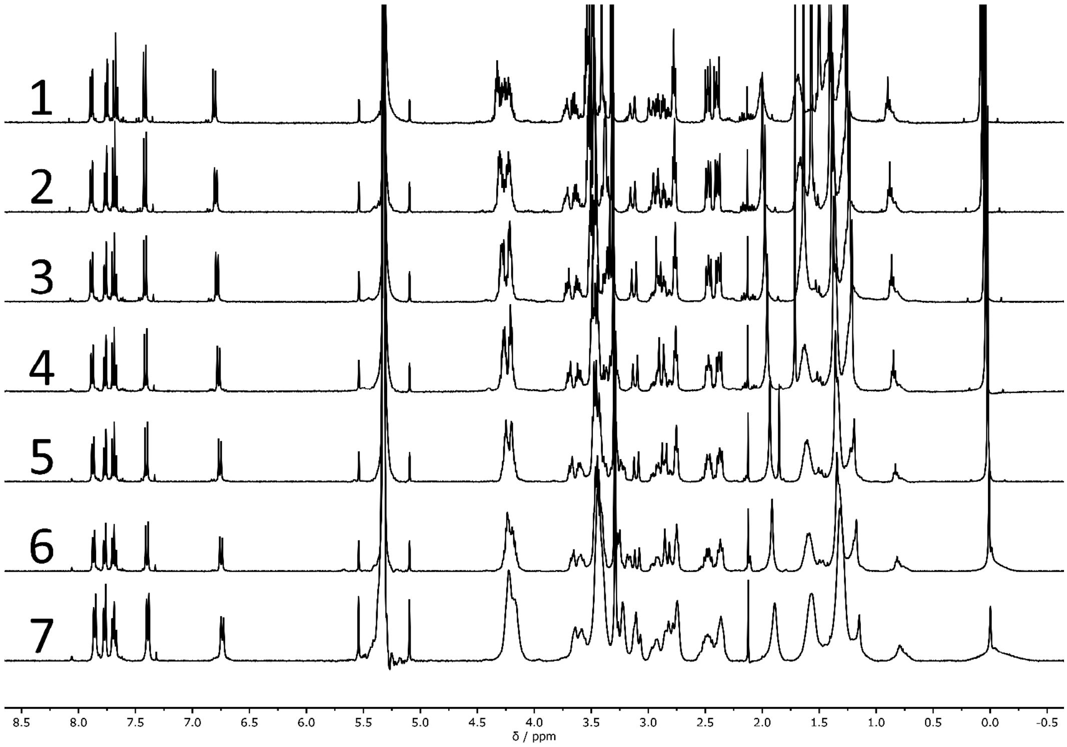

Figure 39 Variable temperature ^{1}H NMR (400 MHz, CD_2Cl_2/CS_2 4/1) spectra of a solution of racemic **C-1** acquired at 1) 25 °C 2) 0 °C 3) −20 °C 4) −40 °C 5) −60 °C 6) −80 °C 7) −105 °C.

Irradiation of Isomer A-1

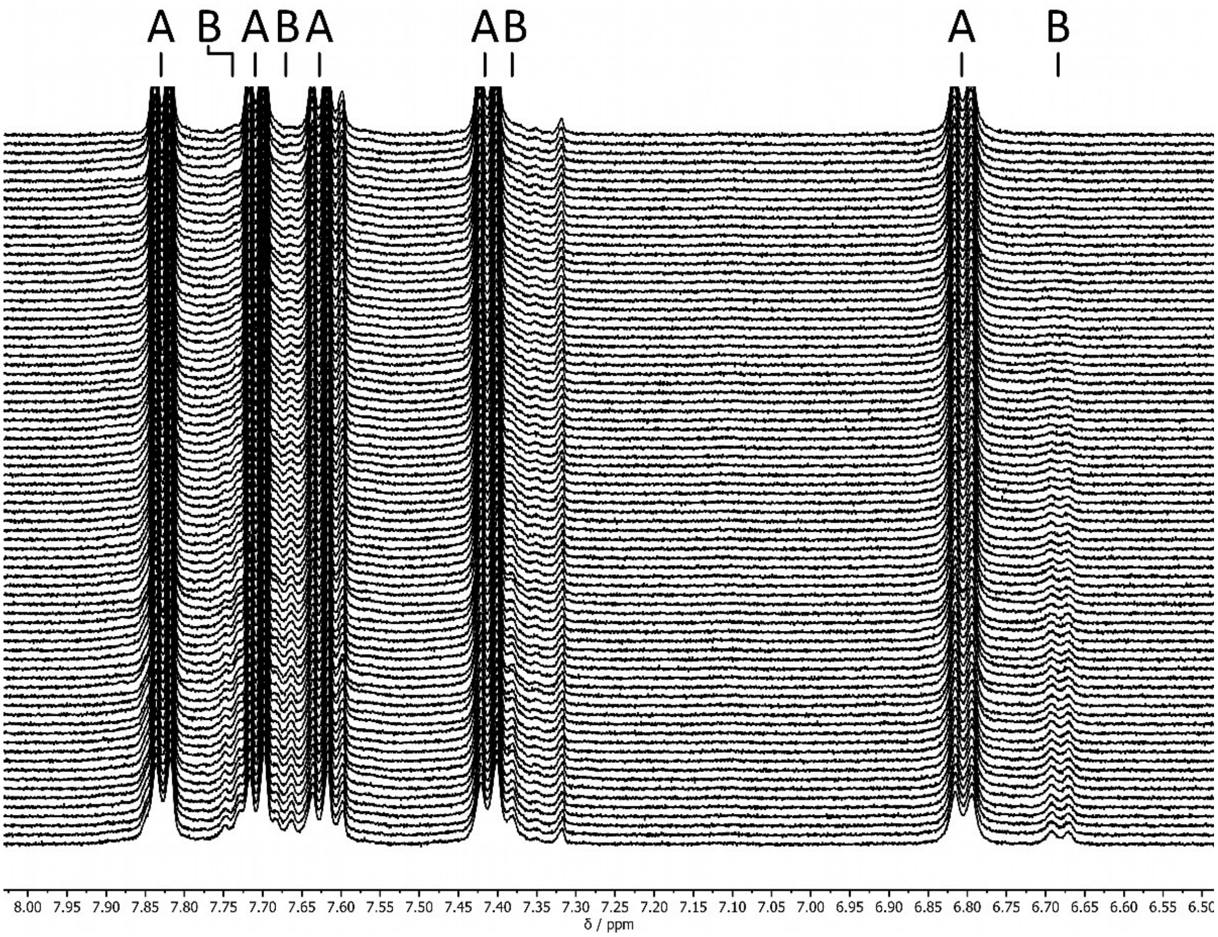

Figure 40 ^{1}H NMR (400 MHz, CD_2Cl_2/CS_2 4/1, −105 °C) spectra acquired during high power *in situ* LED irradiation (405 nm) starting from racemic **A-1** (top spectrum). Spectra were recorded in 25 s intervals. A new set of signals assigned to the **B-1** isomer emerges with increasing irradiation time.

Irradiation of Isomer C-1

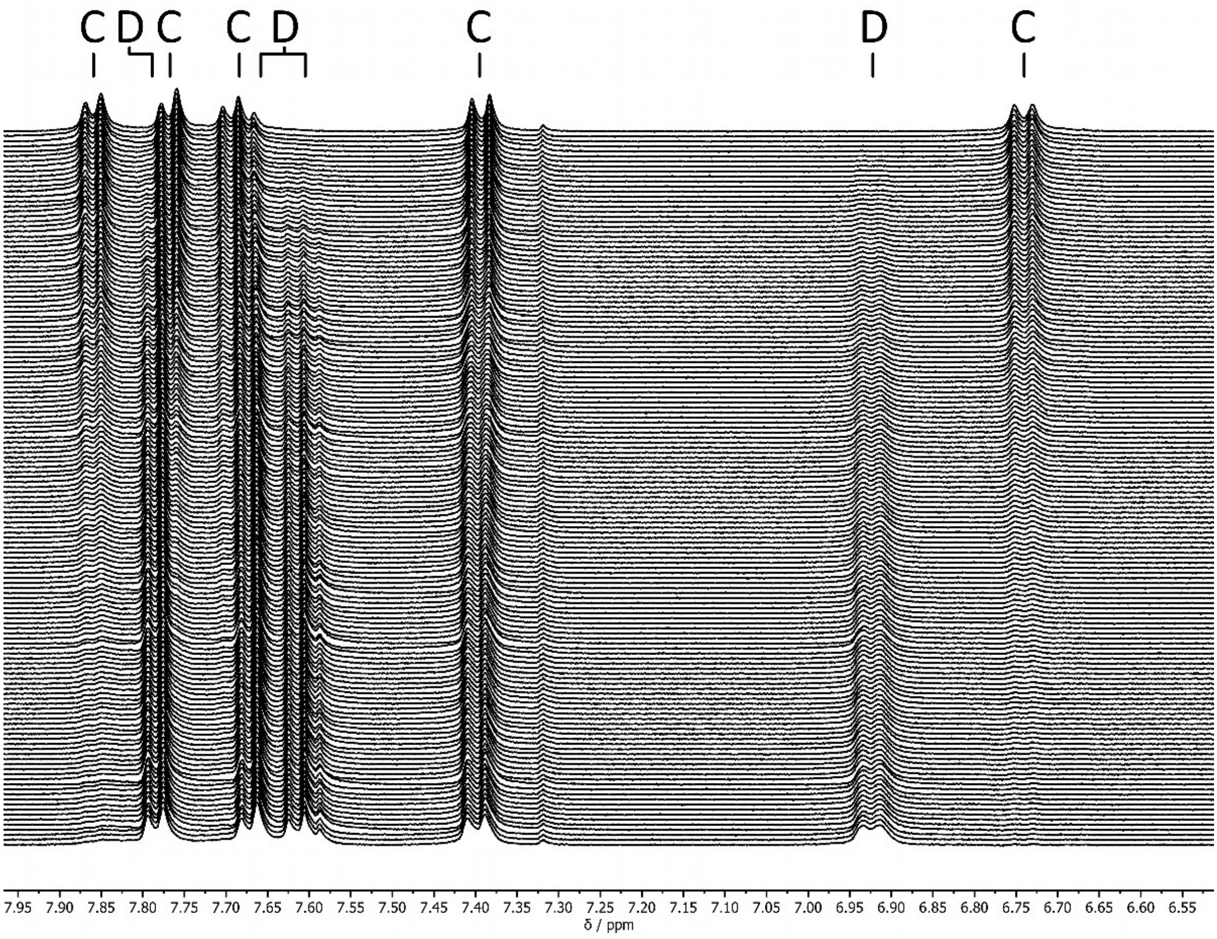

Figure 41 ^{1}H NMR (400 MHz, CD_2Cl_2/CS_2 4/1, −105 °C) spectra acquired during high power *in situ* LED irradiation (450 nm) starting from racemic **C-1** (top spectrum). Spectra were recorded in 35 s intervals. A new set of signals assigned to the **D-1** isomer emerges with increasing irradiation time.

Thermal Conversion of B-1 at Low Temperatures

For the kinetic analysis of the thermal helix inversion of metastable isomer **B-1** to **C-1**, a solution of racemic **A-1** in CD_2Cl_2/CS_2 (4/1, v/v) was irradiated *in situ* with 405 nm light at – 105 °C in order to accumulate isomer **B-1**. Then, the temperature was increased to −80 °C and ^{1}H NMR spectra (400 MHz) were recorded in 2:29 min intervals. Integration of indicative proton signals gives the isomeric ratio for each time point. According to a first order process without entering an equilibrium for the isomerization of **B-1** to **C-1**, the kinetics can be described as follows:

$$\textbf{B-1} \xrightarrow{k} \textbf{C-1} \qquad \text{Equation S8}$$

with *k* being the rate constant. The rate law is

$$\frac{d[\textbf{B-1}]}{dt} = -k[\textbf{B-1}] \qquad \text{Equation S9}$$

Integration of Equation S9 gives

$$\ln[\mathbf{B\text{-}1}] = -kt + \ln[\mathbf{B\text{-}1}]_0 \qquad \text{Equation S9}$$

and can be written as

$$\ln\left(\frac{[\mathbf{B\text{-}1}]_0}{[\mathbf{B\text{-}1}]}\right) = kt \qquad \text{Equation S10}$$

The rate constant *k* of the reaction is obtained by plotting ln([**B-1**]$_0$/[**B-1**]) versus the time *t*, with [**B-1**]$_0$ being the initial concentration of **B-1** and [**B-1**] being the concentration of **B-1** at time *t*. The rate constant *k* is equivalent to the slope of the graph.

The rearranged *Eyring* Equation S6 and the rate constant *k* can be applied to calculate the *Gibbs* energy of activation $\Delta G^{\ddagger}$ for the thermal isomerization.

The half-life $\tau_{1/2}$ of a first order reaction can be expressed as

$$\tau_{1/2} = \frac{\ln 2}{k} \qquad \text{Equation S11}$$

The first order rate constant for the thermal decay of **B-1** is $k_{B\rightarrow C}$ = 4.2 × 10^{-4} s^{-1} at −80 °C. Using Equations S6 and S11, this gives a *Gibbs* energy of activation $\Delta G^{\ddagger}$ = 14.1 kcal mol^{-1} and a half-life of 28 min at −80 °C, respectively.

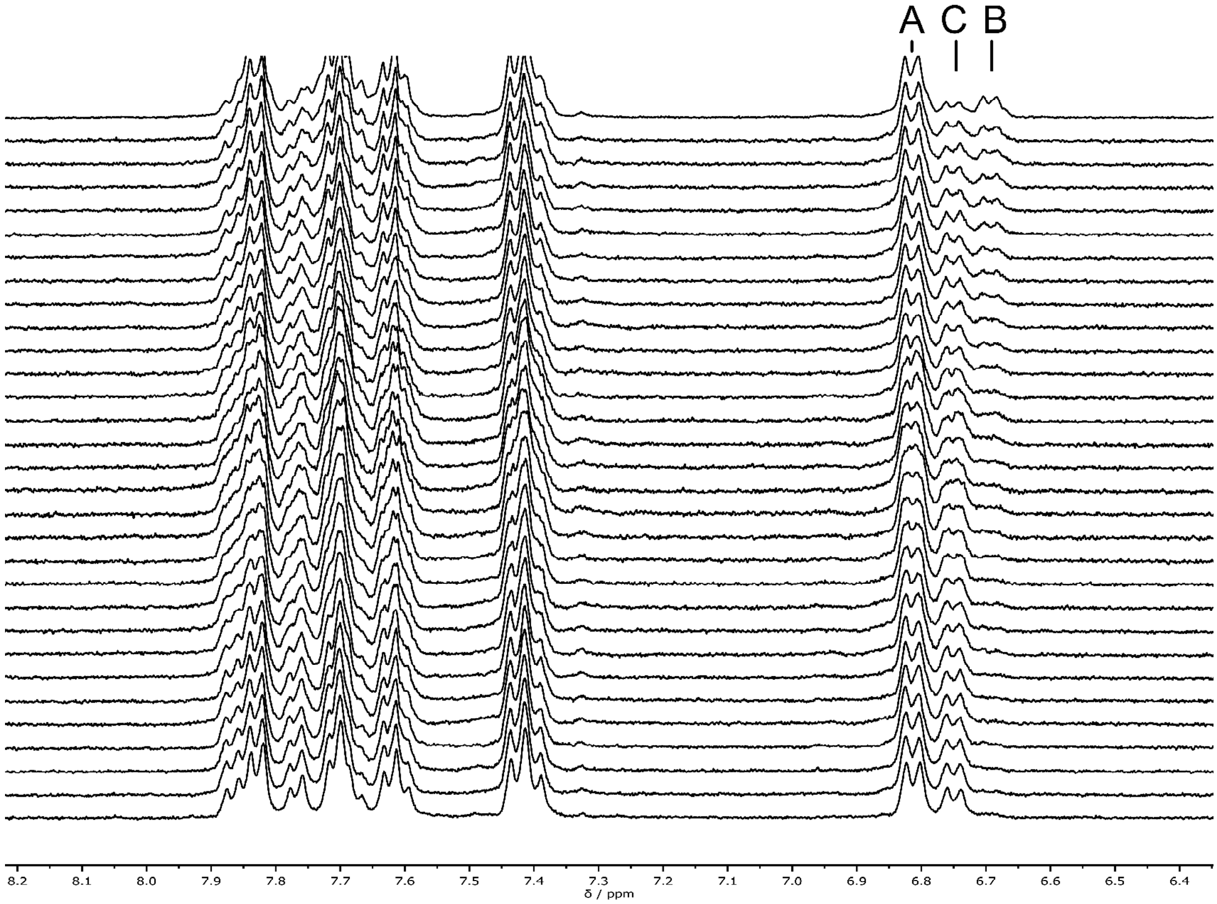

Figure 42 Aromatic region of ^{1}H NMR (400 MHz, CD_2Cl_2/CS_2 4/1, −80 °C) spectra recorded during thermal decay of **B-1** in the dark. Starting from racemic **A-1**, **B-1** was accumulated by *in situ* irradiation with 405 nm at −105 °C. The thermal decay of the metastable **B-1** isomer was monitored at −80 °C in the dark (starting from the top

spectrum). The time difference between the top spectrum and the second spectrum is 7:46 min. The following spectra were recorded in 2:29 min intervals.

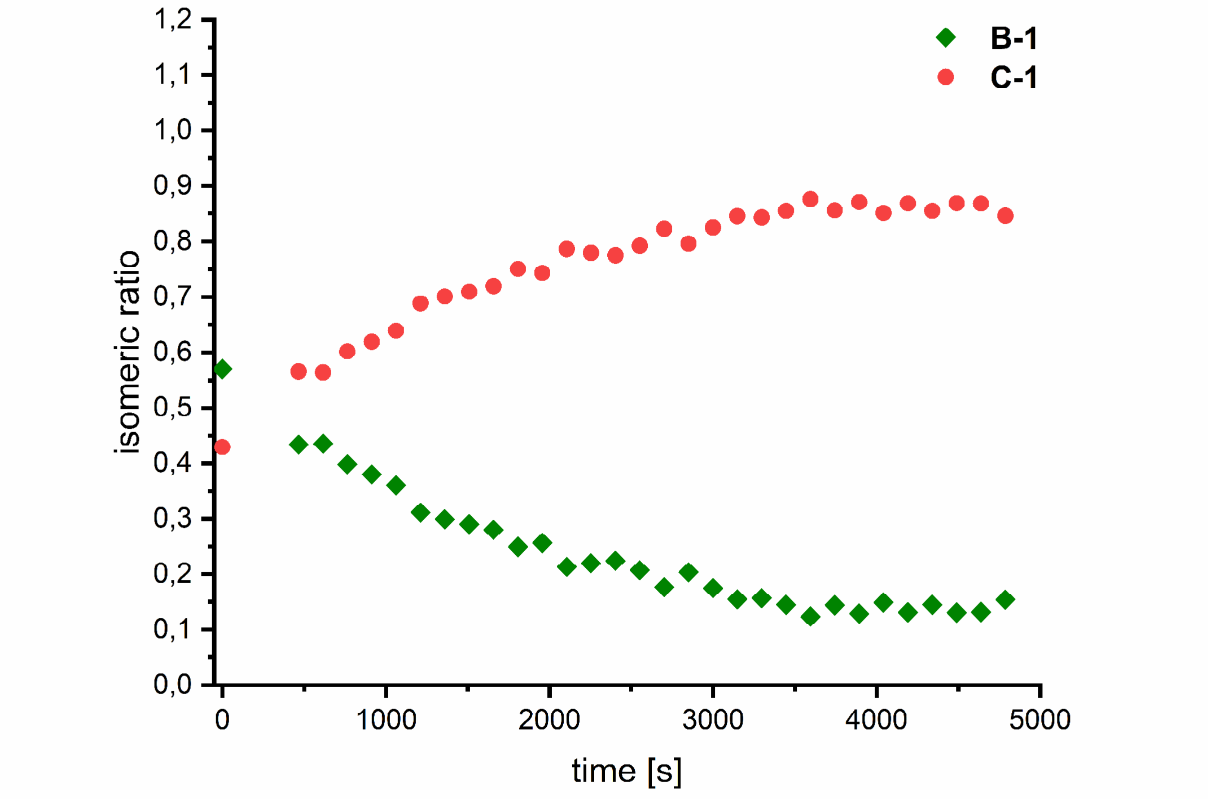

Figure 43 Thermal decay of **B-1** in the dark at −80°C over time. Isomeric ratios were obtained by integration of indicative ^{1}H NMR signals.

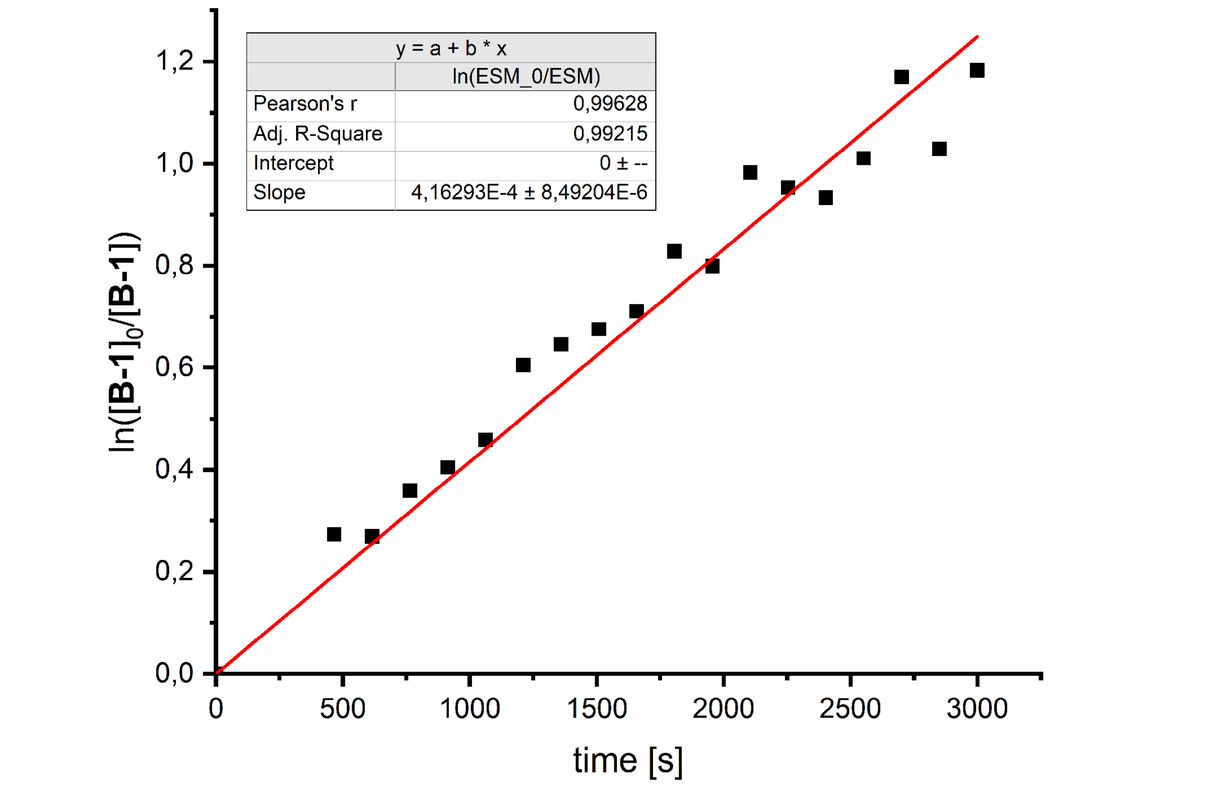

Figure 44 ln plot of thermal **B-1** decay at −80°C in the dark. The slope is 4.2 × 10^{-4} s^{-1}, which corresponds to a *Gibbs* energy of activation $\Delta G^{\ddagger}$ = 14.1 kcal mol^{-1} for the **B-1**- to **C-1** helix inversion at −80°C.

Thermal Conversion of D-1 at Low Temperatures

A solution of racemic **C-1** in CD_2Cl_2 was irradiated with 450 nm at −80°C to accumulate **D-1**. Subsequently, the solution enriched in **D-1** was warmed to −50 °C. Then, ^1H NMR spectra (400 MHz) were recorded in 123 s intervals in the dark to follow the thermal decay of **D-1**. Integration of indicative proton signals gives the isomeric ratio over time. According to a first order process without entering an equilibrium for the isomerization of **D-1** to **A-1**, the kinetics can be analyzed in an analogous way as described above for the thermal **B-1** to **C-1** isomerization. The first order rate constant for the thermal decay of **D-1** is obtained from the linear plot and is $k_{D\rightarrow A}$ = 2.6 × 10^{-4} s^{-1}. Using Equations S6 and S11, this gives a *Gibbs* energy of activation $\Delta G^{\ddagger}$ = 16.6 kcal mol^{-1} and a thermal half-life of 44 min for the **D-1**- to **A-1** helix inversion at −50 °C, respectively.

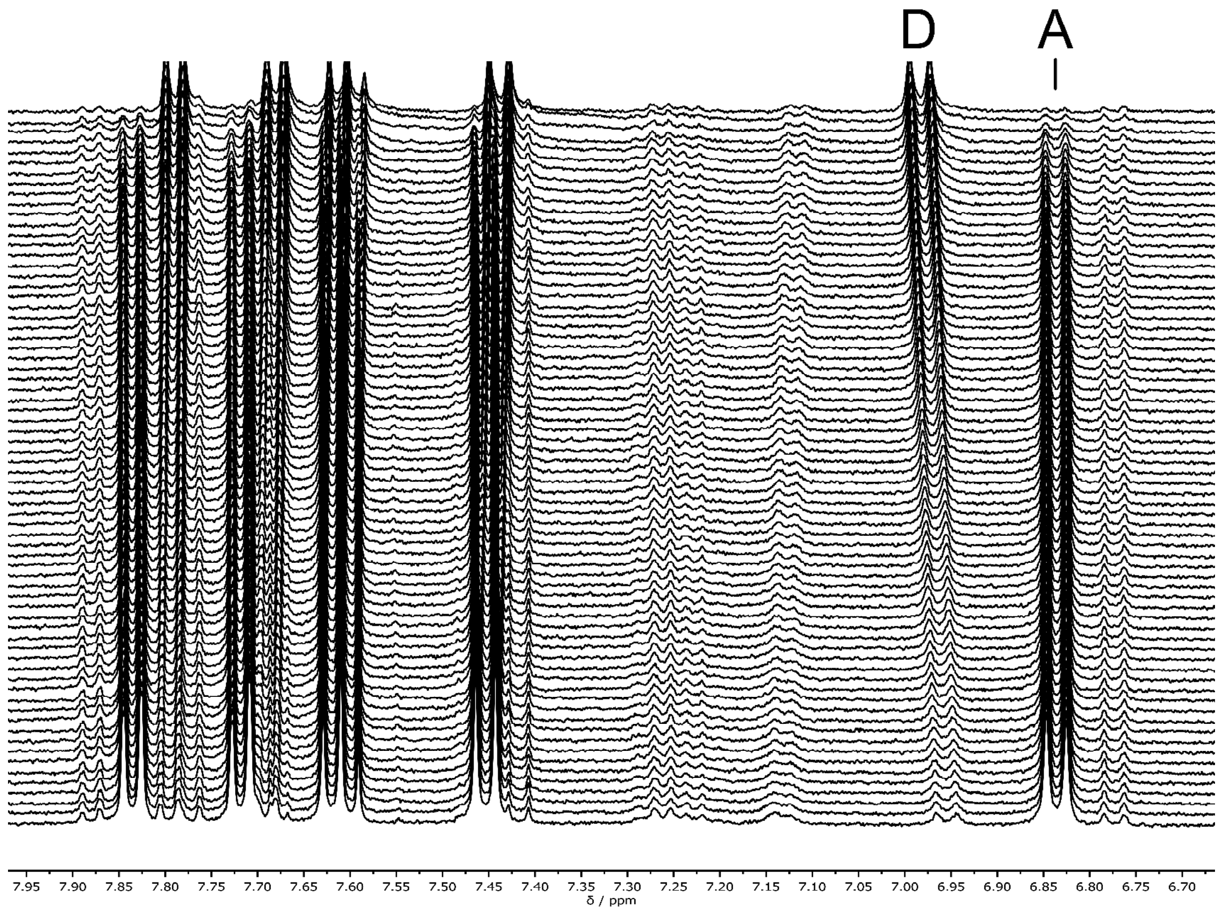

Figure 45 ^{1}H NMR spectra (400 MHz, CD_2Cl_2, −50°C) recorded during thermal isomerization of **D-1** to **A-1** at −50°C in the dark (top to bottom). The spectra were recorded in intervals of 123 s.

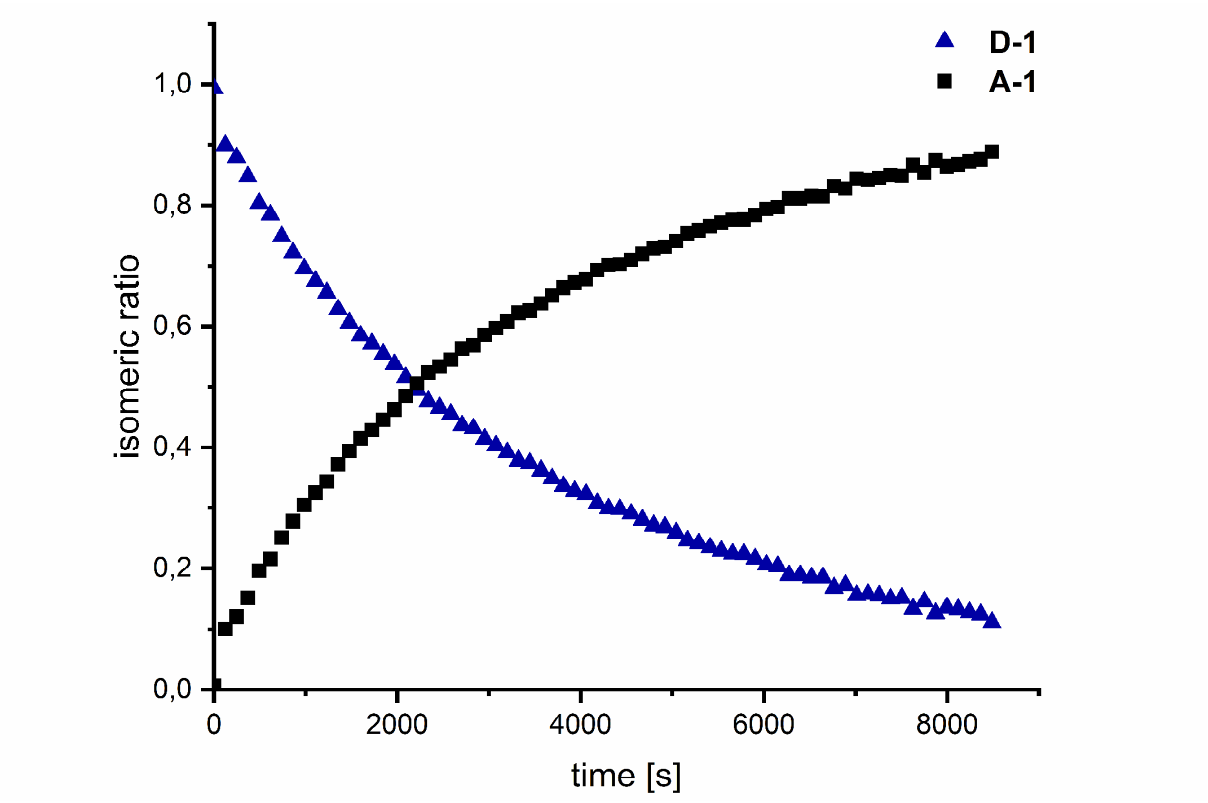

Figure 46 Thermal decay of **D-1** at −50°C in the dark. Isomeric ratios were determined by integration of indicative ^{1}H NMR signals.

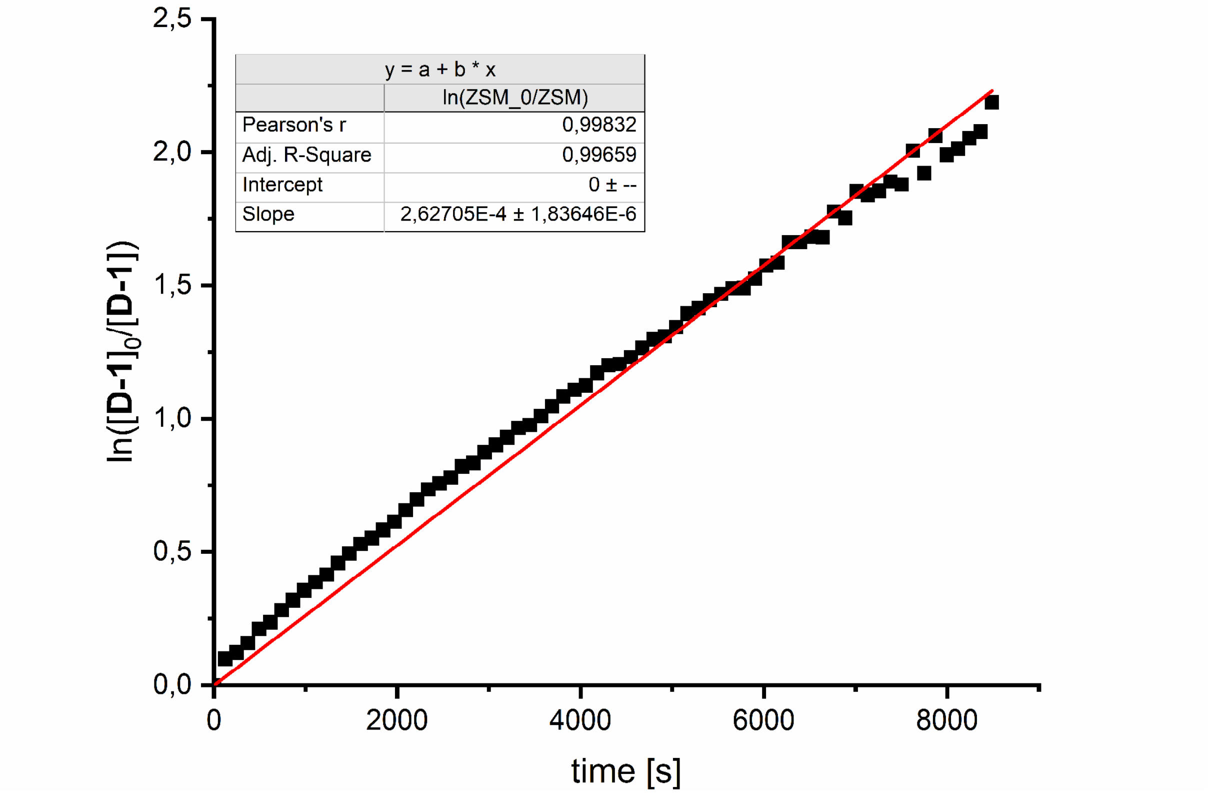

Figure 47 ln plot of thermal **D-1** decay at −50°C in the dark. The slope is 2.6 × 10^{-4} s^{-1}, which corresponds to a *Gibbs* energy of activation $\Delta G^{\ddagger}$ = 16.6 kcal mol^{-1} for the **D-1**- to **A-1** thermal helix inversion.

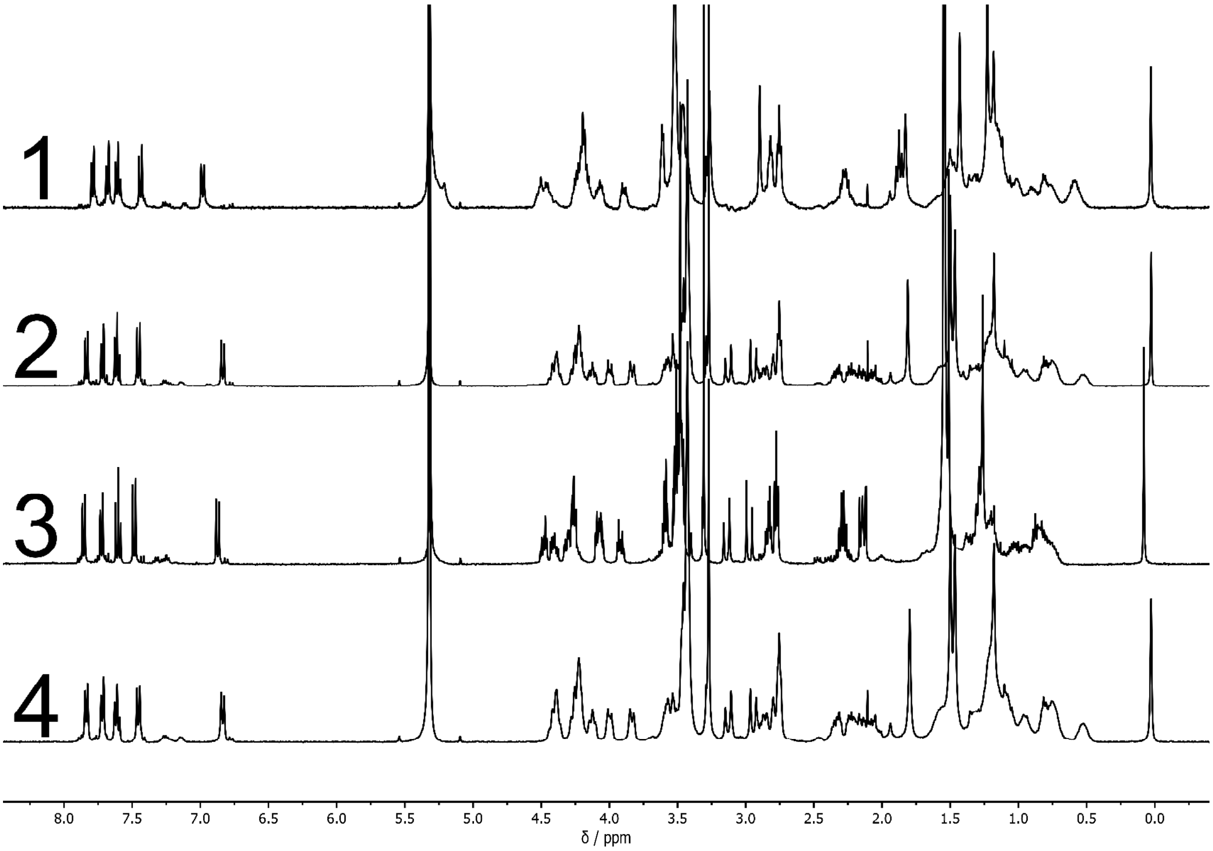

Figure 48 Thermal isomerization of **D-1** at low temperature (^{1}H NMR, 400 MHz, CD_2Cl_2). 1) Solution enriched in **D-1**, prior to thermal decay at −50°C in the dark. 2) Isomer **D-1** is fully converted to **A-1** after 215 min at −50 °C. 3) The same solution after warming to 25 °C. 4) After recooling of the solution to −50 °C. No shift of signals is noticeable between 2) and 4), confirming that **D-1** is thermally converted to **A-1** without any observable intermediate at the lower temperature.

Full Cycle of Motor Operation

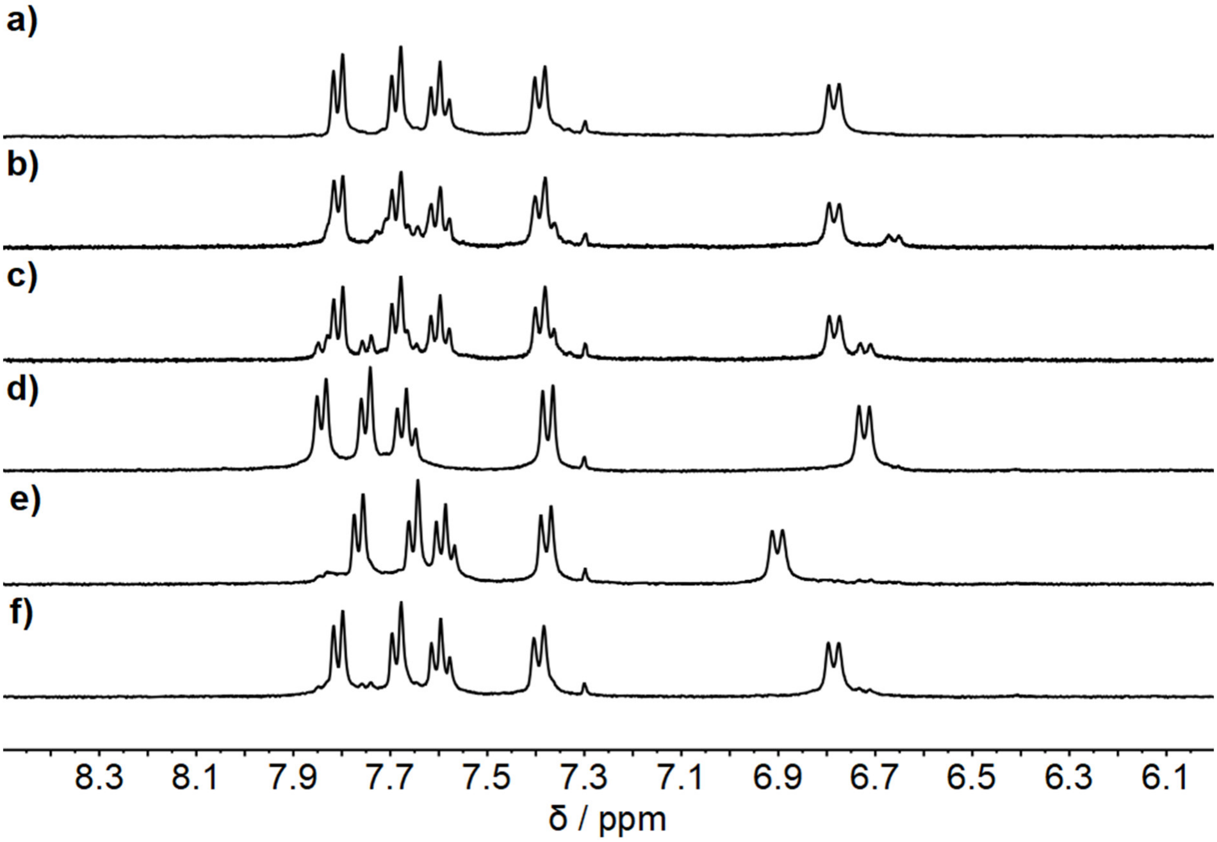

Figure 49 ^{1}H NMR spectra (400 MHz, −105 °C, CD_2Cl_2/CS_2 4/1) recorded during low-temperature irradiation experiments of motor **1**; only the aromatic region is shown for clarity. **a)** Racemic **A-1**. **b)** Photostationary state (pss) reached after *in situ* irradiation of **A-1** with 405 nm light. A new set of signals attributed to **B-1** appeared. **c)** After thermal annealing to 23 °C in the dark, **B-1** disappeared and the proportional appearance of **C-1** was observed. **d)** Racemic **C-1**. **e)** pss reached after subsequent *in situ* irradiation of **C-1** with 450 nm light. A new set of signals appeared, which is attributed to **D-1**. **f)** After thermal annealing at 23 °C in the dark **D-1** fully converted to **A-1**.

Switching Behaviour of HTI 2

Photoswitching

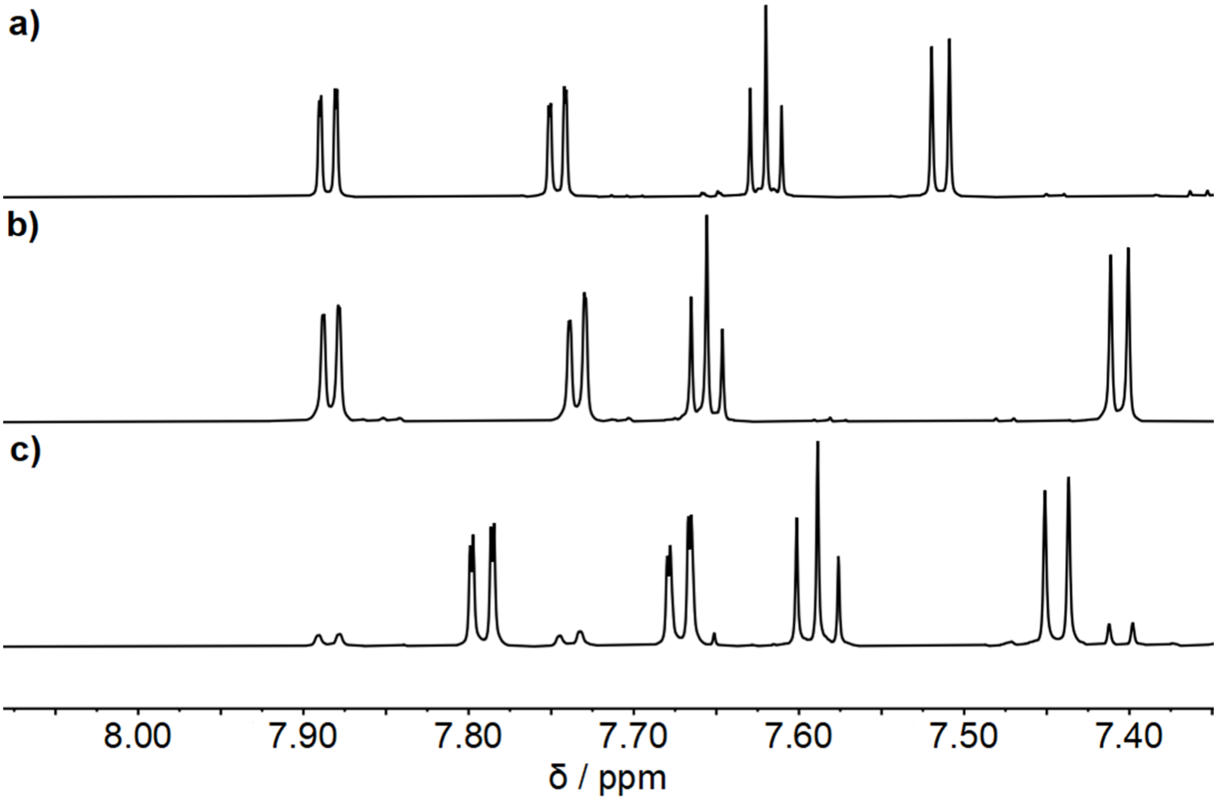

Figure 50 ^{1}H-NMR (400 MHz, CD_2Cl_2, 25 °C) spectra; only the aromatic region is shown for clarity. a) Racemic **A-2**. b) Racemic **C-2**. c) pss reached after external irradiation of **C-2** with 450 nm light. A new set of signals appeared, which could be assigned to **D-2.**

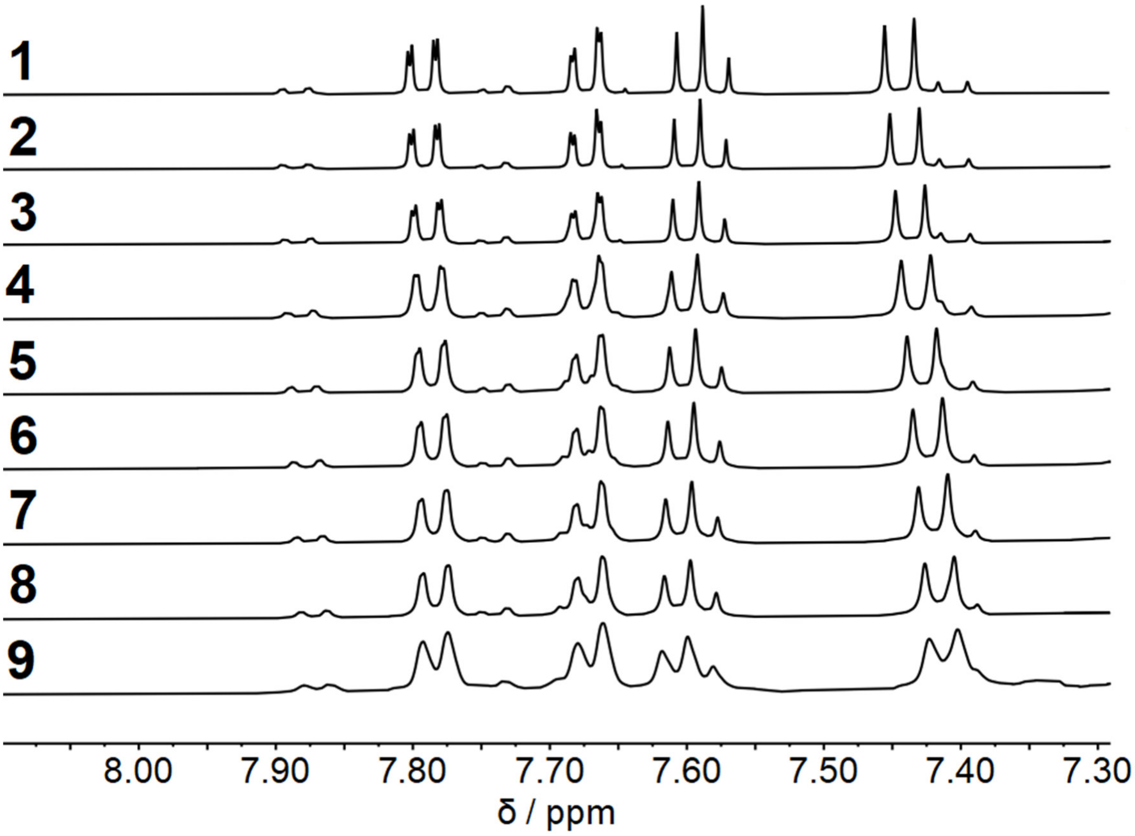

Figure 7 Variable temperature ^{1}H NMR (400 MHz, CD_2Cl_2) spectra of a solution enriched in racemic **D-2** acquired at 1) 0 °C 2) −10 °C 3) −20 °C 4) −30 °C 5) −40 °C 6) −50 °C 7) −60 °C 8) −70 °C 9) −80 °C. Only the aromatic region is shown for clarity.

Thermal Conversion of D-2 at Elevated Temperature

A solution of **C-2** in CD_2Cl_2 was irradiated with 490 nm light until the solution was enriched in **D-2**. Then the solvent was removed *in vacuo* and replaced with $(CDCl_2)_2$. The solution was kept in an amberized NMR tube placed in an oil bath at 65 °C. After the indicated time intervals, the tube was put in an ice bath in order to halt the isomerization reaction and a ^{1}H NMR spectrum was recorded at 25 °C. Isomeric ratios were determined by the integration of indicative proton signals.

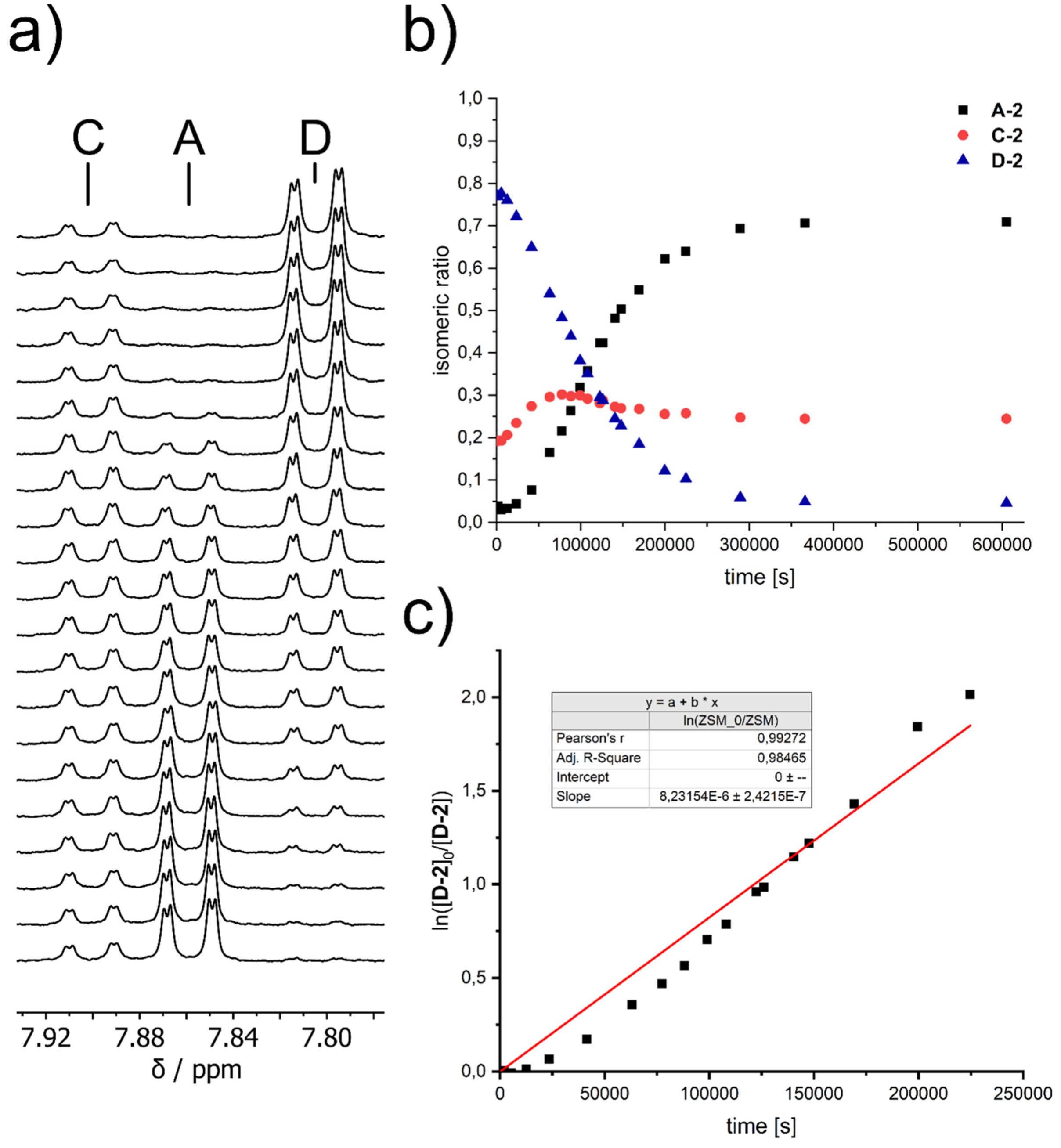

Figure 51 Kinetic analysis of thermal **D-2** isomerization at 65 °C in $(CDCl_2)_2$ in the dark. a) Indicative ^{1}H NMR (400 MHz, $(CDCl_2)_2$, 25 °C) signals that were used for determination of isomeric ratios. b) Changing isomeric ratios over time obtained by integration of indicative ^{1}H NMR signals. c) First order kinetic analysis of the thermal decay of isomer **D-2**. The slope of the linear fit is 8.2 × 10^{-6} s^{-1}.

The observed thermal isomerization behavior of **D-2** is in line with a first order decay of **D-2** to **C-2**, coupled to a follow-up equilibrium between **C-2** and **A-2**.

$$\mathbf{D\text{-}2} \longrightarrow \mathbf{C\text{-}2} \rightleftharpoons \mathbf{A\text{-}2}$$

The rate constant k for the thermal decay of **D-2** is obtained as the slope of a linear fit as described in Equation S10 (replacing **B-1** by **D-2** in that equation). The rate constant is $k_{D \rightarrow C}$ = 8.2 × 10^{-6} s^{-1}, which corresponds to a *Gibbs* energy of activation $\Delta G^{\ddagger}$ = 27.7 kcal mol^{-1} for the thermal **D-2** to **C-2** double bond isomerization at 65 °C.

The isomerization kinetics of **D-2** at 65 °C were additionally simulated using the parameter estimation task in COPASI 4.34[5] with the experimental data as input. The 'Evolutionary Programming' method integrated in COPASI was used, with the number of generations set to 2000 and a population size of 200. Three species (**A-2**, **C-2**, **D-2**) were defined and the initial concentrations were set as 0 (**A-2**), 0.2 (**C-2**) and 0.8 (**D-2**) mol L^{-1} respectively. In a first attempt, all species were considered to be in dynamic equilibrium with each other. Reactions that did not contribute significantly ($k < 1.0 \times 10^{-11}$ s^{-1} for the first iteration, $k < 1.0 \times 10^{-6}$ s^{-1} for the second iteration) were removed and the model updated accordingly. After a total of three iterations, the following model was obtained:

$$\mathrm{D\text{-}2} \rightarrow \mathrm{C\text{-}2} \text{ (mass action irreversible)}$$

$$\mathrm{C\text{-}2} \rightleftharpoons \mathrm{A\text{-}2} \text{ (mass action reversible)}$$

The corresponding rate constants are

$$k_{\mathrm{D\text{-}2 \rightarrow C\text{-}2}} = 7.8 \times 10^{-6}\ \mathrm{s}^{-1}$$

$$k_{\mathrm{C\text{-}2 \rightarrow A\text{-}2}} = 1.6 \times 10^{-5}\ \mathrm{s}^{-1}$$

$$k_{\mathrm{A\text{-}2 \rightarrow C\text{-}2}} = 4.8 \times 10^{-6}\ \mathrm{s}^{-1}$$

The *Gibbs* energies of activation at 65 °C can be obtained from the rate constants as described earlier.

D-2 to **C-2**: $\Delta G^{\ddagger}$ = 27.7 kcal mol^{-1}

C-2 to **A-2**: $\Delta G^{\ddagger}$ = 27.3 kcal mol^{-1}

A-2 to **C-2**: $\Delta G^{\ddagger}$ = 28.1 kcal mol^{-1}

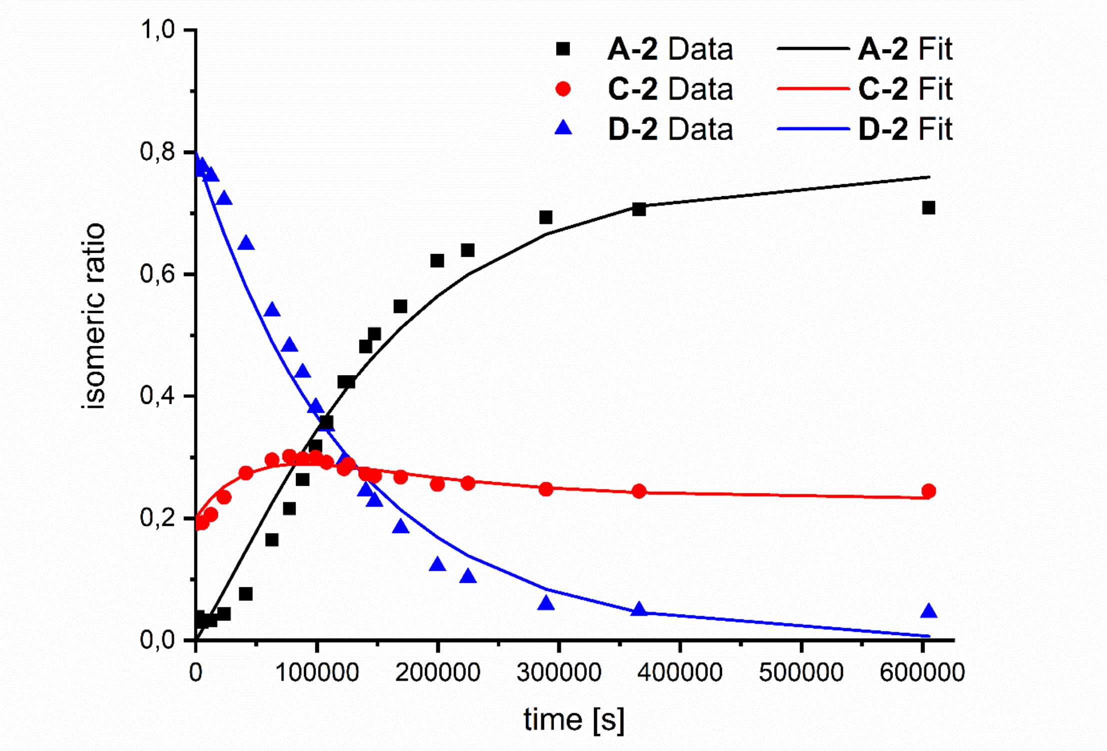

Figure 52 Kinetic analysis of thermal **D-2** isomerization at 65 °C in $(CDCl_2)_2$ in the dark. A plot of the isomeric ratios over time is shown. Experimental data (obtained from ^{1}H NMR integration) are plotted as symbols, the simulated fits obtained from COPASI are plotted as lines. The model is based on an irreversible conversion of **D-2** to **C-2** and an equilibrium between **C-2** and **A-2**.

Separation of Enantiomers

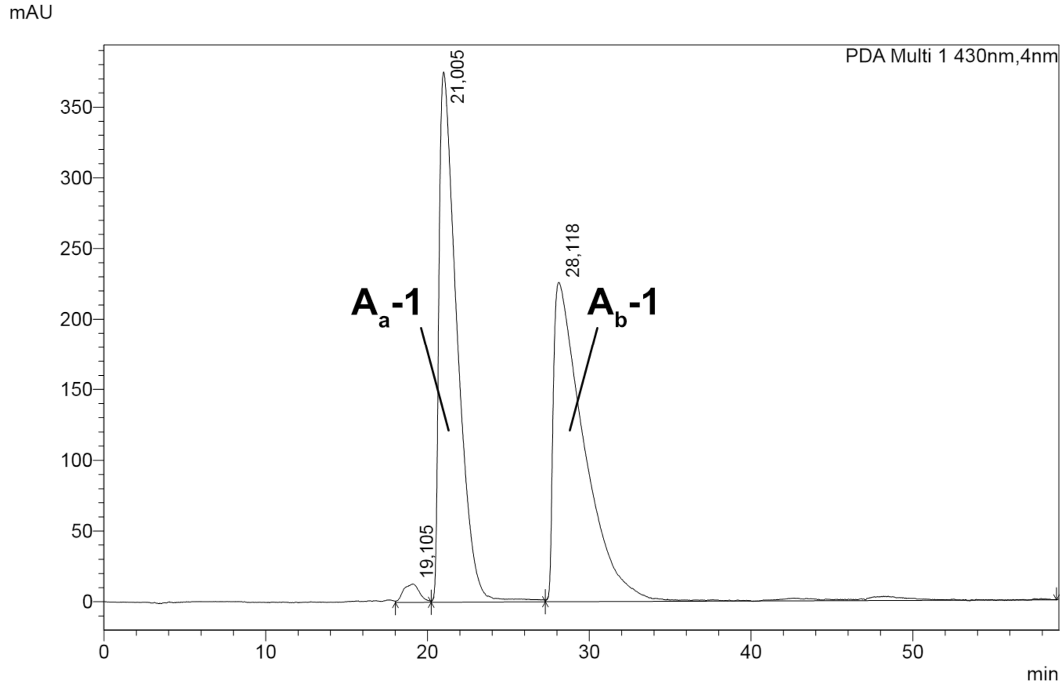

Figure 53 Chromatogram of the chiral resolution of **A-1** by chiral HPLC on a Daicel Chiralpak ID semi-preparative column eluting with *i*-PrOH/*n*-hexane 60/40 at 30 °C and a flow rate of 4.5 mL/min. Retention times are approx. 21 min for **A$_a$-1** and 28 min for **A$_b$-1**. The chromatogram was recorded at 430 nm.

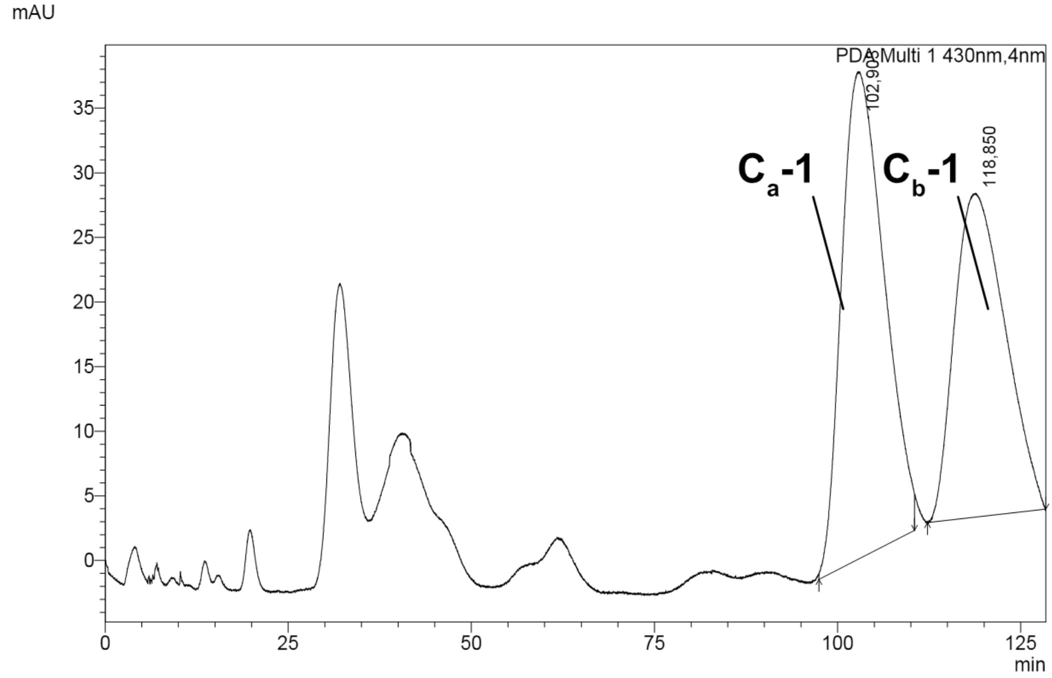

Figure 54 Chromatogram of the chiral resolution of **C-1** by chiral HPLC on a Daicel Chiralpak IC semi-preparative column eluting with *i*-PrOH (100%) at 30 °C and a flow rate of 2.8 mL/min. Retention times are approx. 103 min for **C$_a$-1** and 118 min for **C$_b$-1**. The chromatogram was recorded at 430 nm.

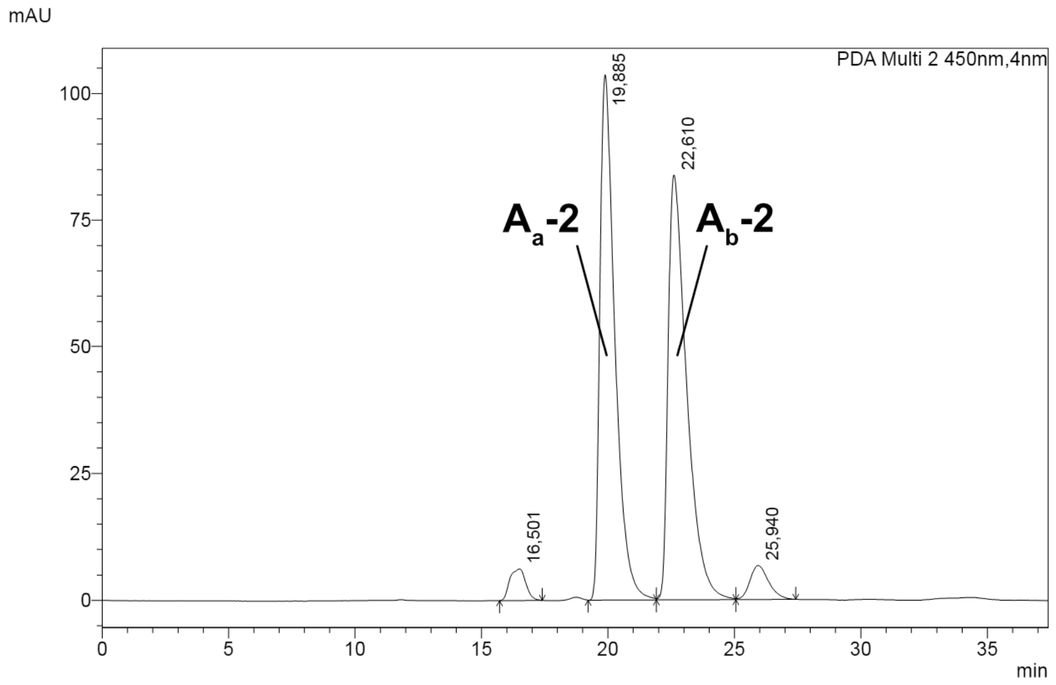

Figure 55 Chromatogram of the chiral resolution of **A-2** by chiral HPLC on a Daicel Chiralpak IC semip-reparative column eluting with EtOAc/*n*-heptane 25/75 at 30 °C and a flow rate of 2 mL/min. Retention times are approx. 20 min for **A$_a$-2** and 23 min for **A$_b$-2**. The chromatogram was recorded at 450 nm.

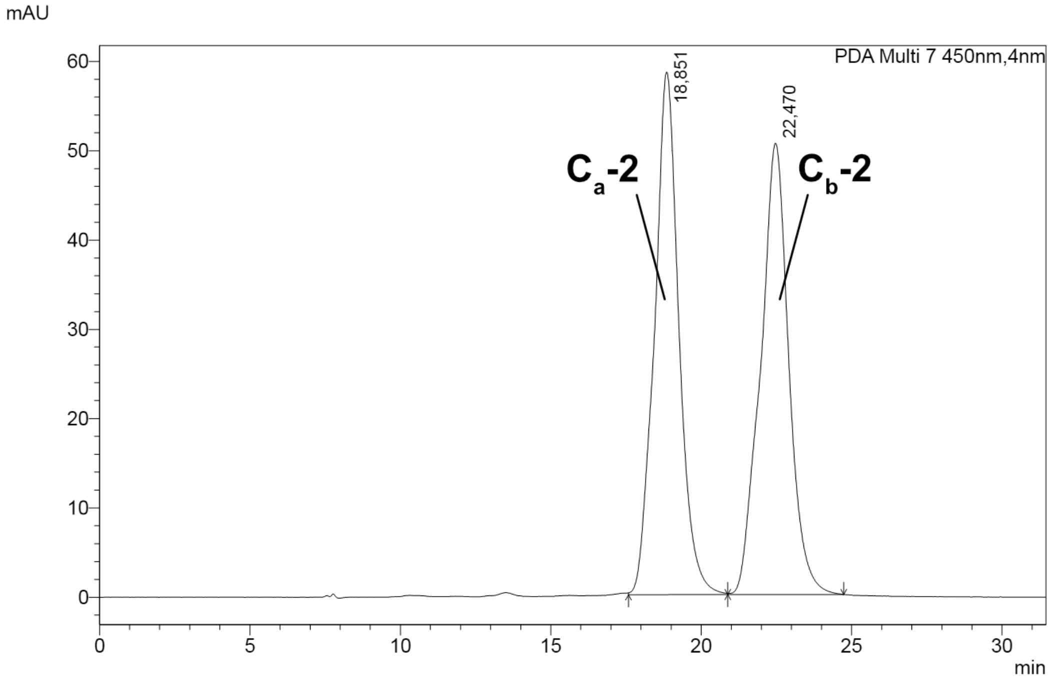

Figure 56 Chromatogram of the chiral resolution of **C-2** by chiral HPLC on a Daicel Chiralpak IC semi-preparative column eluting with EtOAc/*n*-heptane 40/60 at 30 °C and a flow rate of 2 mL/min. Retention times are approx. 19 min for **C$_a$-2** and 22 min for **C$_b$-2**. The chromatogram was recorded at 450 nm.

Photophysical Properties

UV-Vis Absorption Spectra and Photoisomerization

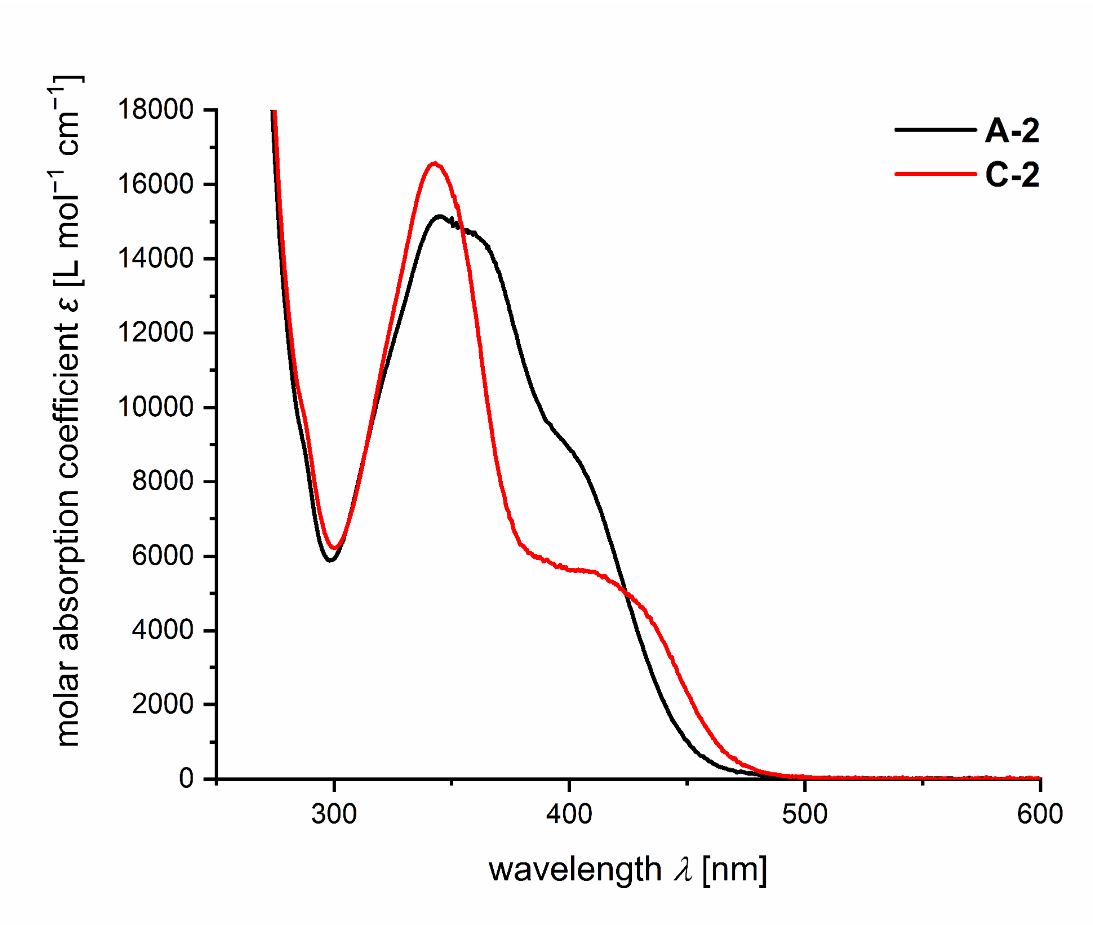

Figure 57 Molar absorption coefficients of racemic **A-2** (black spectrum) and racemic **C-2** (red spectrum) in CH_2Cl_2.

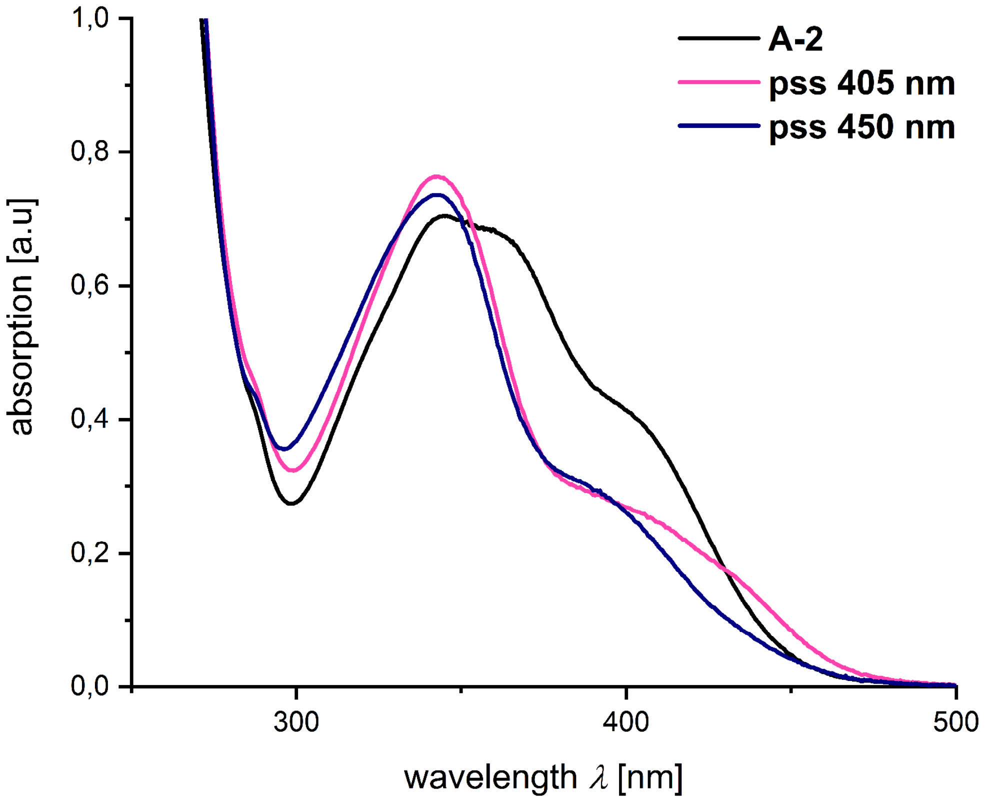

Figure 58 Absorption spectra recorded during photoisomerization of **A-2** in CH_2Cl_2 at 23 °C. A sample of racemic **A-2** (black spectrum) was irradiated with 405 nm light (pink spectrum), followed by irradiation with 450 nm light (blue spectrum). Irradiation of **A-2** with 405 nm light leads to formation of **C-2** (via transient **B-2**), which is subsequently photoisomerized to **D-2**. At 450 nm irradiation strong accumulation of isomer **D-2** takes place (blue spectrum).

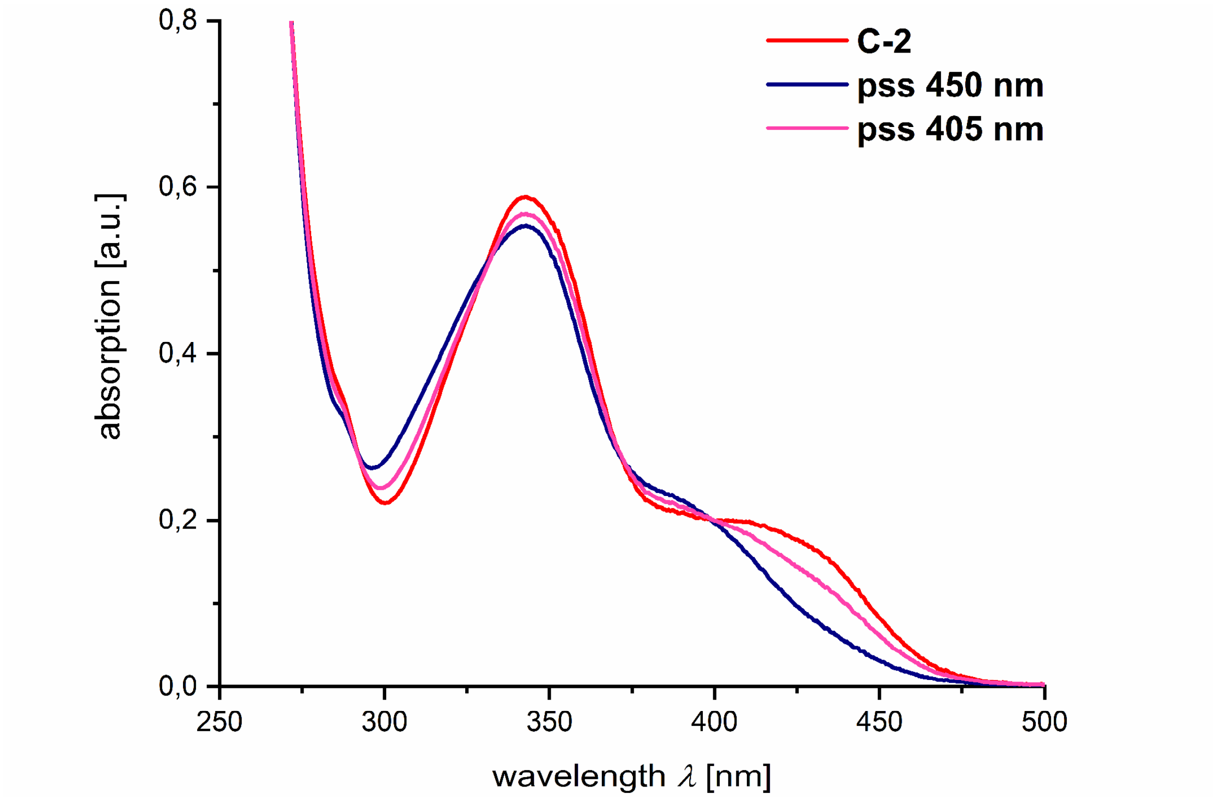

Figure 59 Absorption spectra recorded during photoisomerization of **C-2** in CH_2Cl_2 at 23 °C. A sample of racemic **C-2** (red spectrum) was irradiated with 450 nm light (blue spectrum), followed by irradiation with 405 nm light (pink spectrum). Irradiation of **C-2** with 450 nm light leads to strong accumulation of **D-2** (blue spectrum), which photoisomerizes back to **C-2** at shorter irradiation wavelengths (405 nm in this case, pink spectrum).

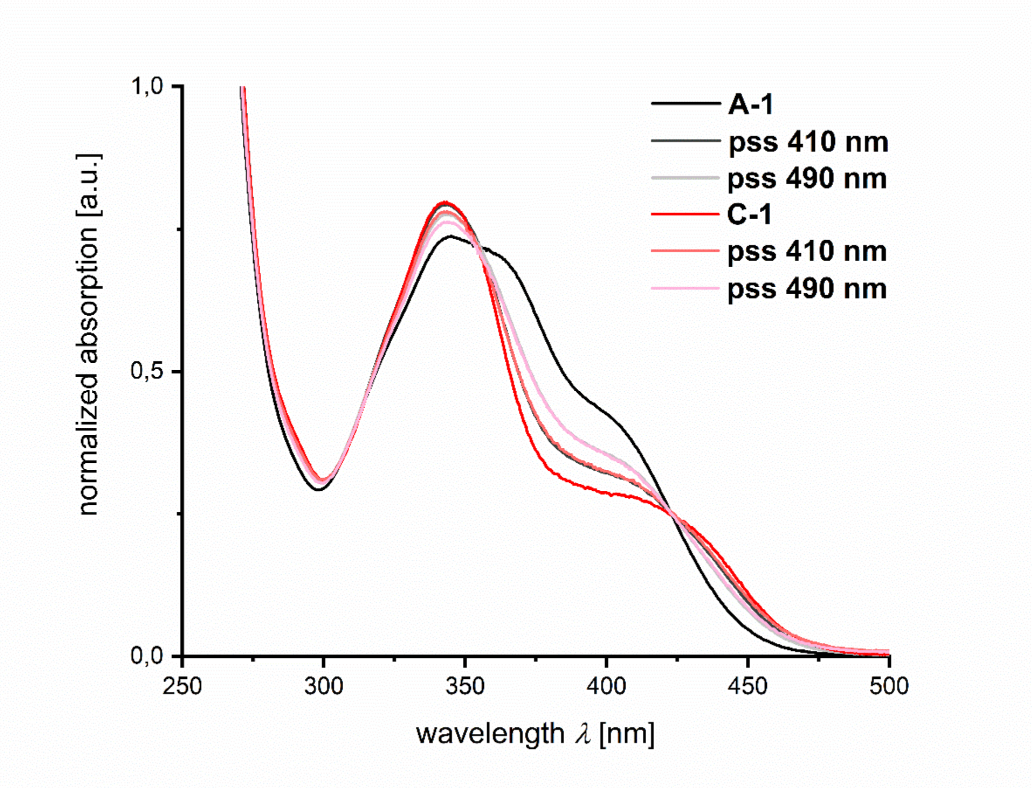

Figure 60 Absorption spectra of racemic **A-1** (black spectrum) and racemic **C-1** (red spectrum), as well as spectra recorded after reaching the pss at 410 nm or 490 nm irradiation. All spectra were recorded in CH_2Cl_2 and are normalized with respect to the isosbestic point at 423 nm. Starting irradiations from either isomer **A-1** or **C-1**, the same pss spectra are obtained.

ECD Spectra

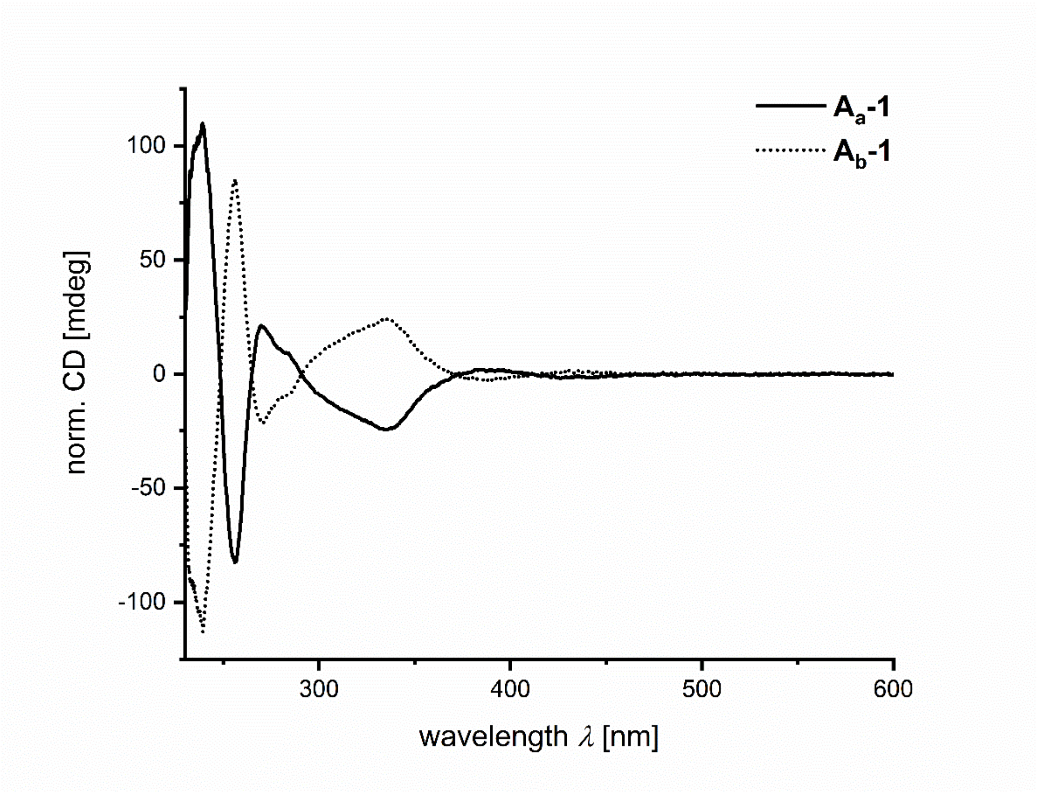

Figure 61 ECD spectra of chirally resolved **A-1** in CH_2Cl_2 solution at 23 °C. Subscript **a** and **b** denote the respective HPLC fraction (see **Figure 53**).

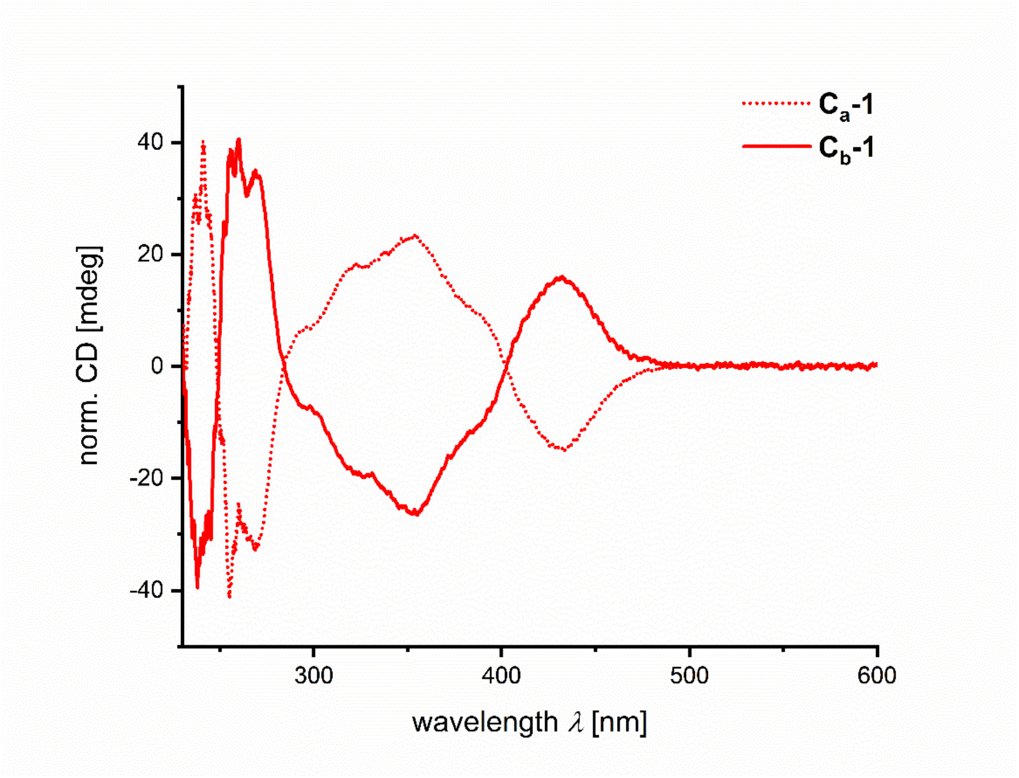

Figure 62 ECD spectra of chirally resolved **C-1** in CH_2Cl_2 solution at 23 °C. Subscript **a** and **b** denote the respective HPLC fraction (see **Figure 54**).

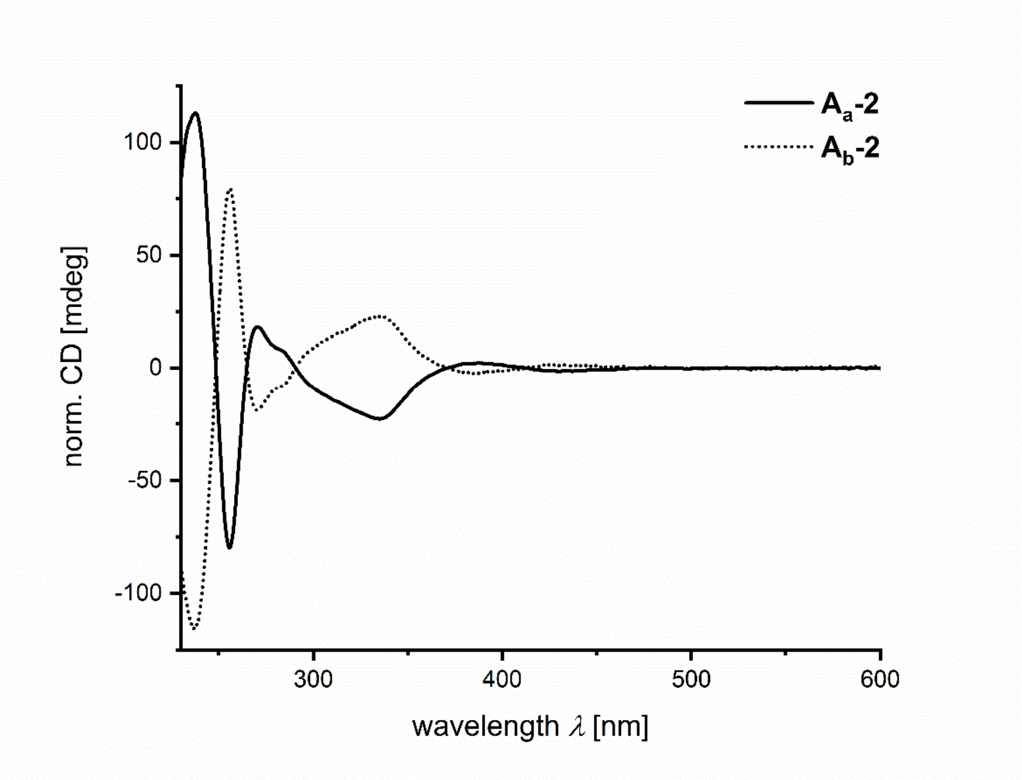

Figure 63 ECD spectra of chirally resolved **A-2** in CH_2Cl_2 at 25 °C. Subscript **a** and **b** denote the HPLC fraction (see **Figure 55**).

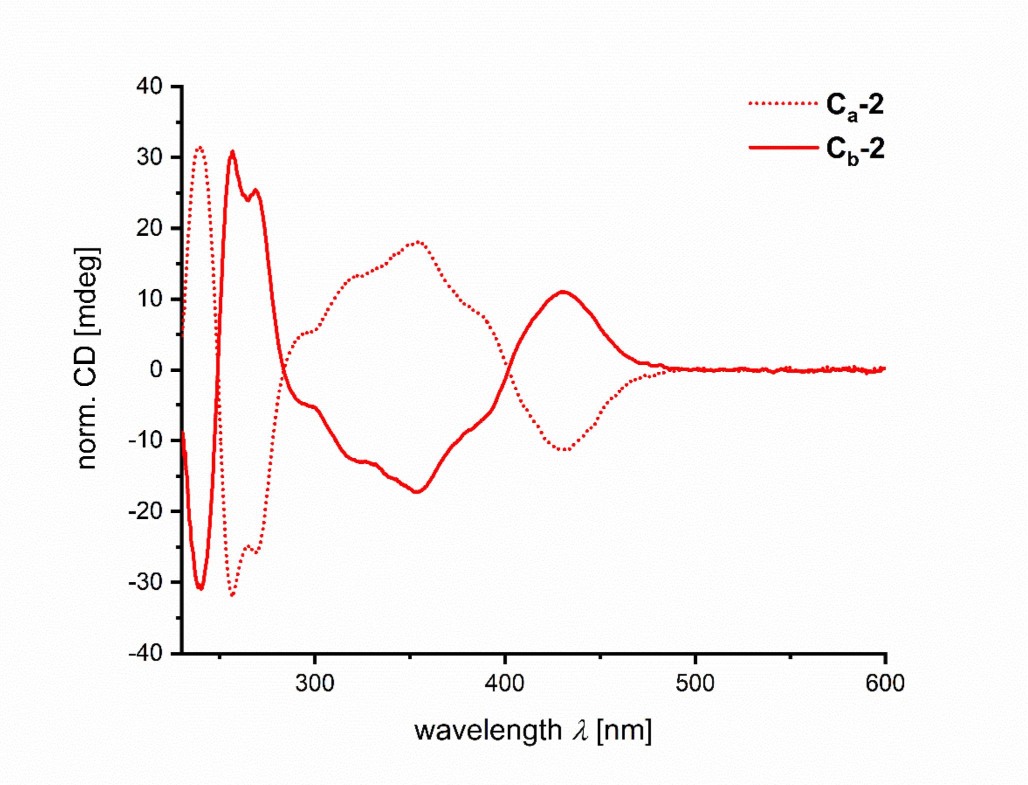

Figure 64 ECD spectra of chirally resolved **C-2** in CH_2Cl_2 solution at 25 °C. Subscript **a** and **b** denote the respective HPLC fraction (see **Figure 56**).

Experimentally determined *g*-Factors

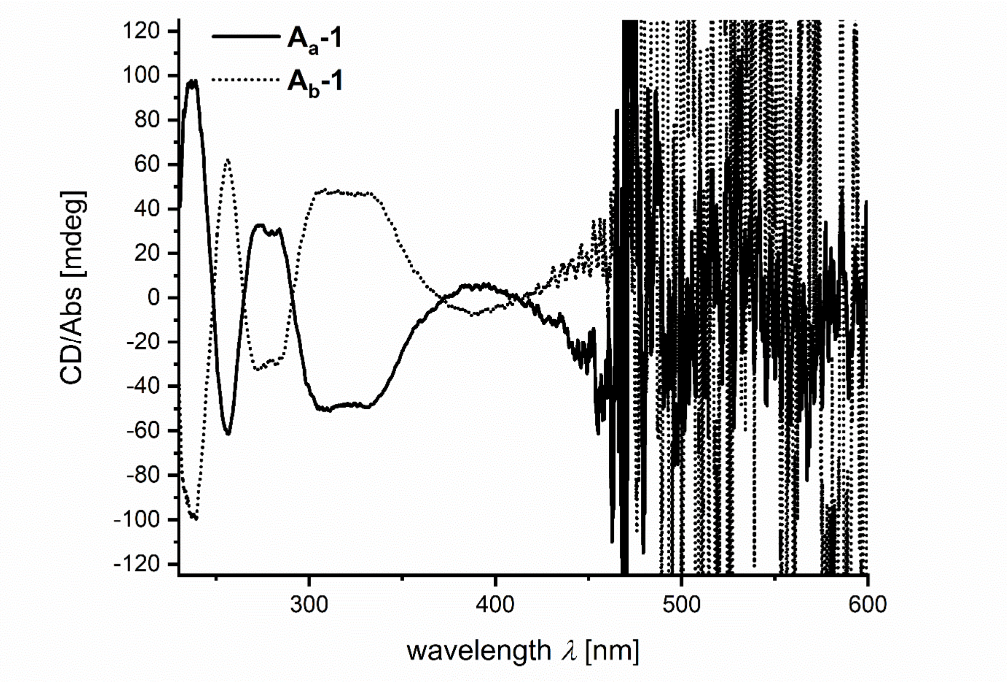

Figure 65 *g*-Factors for enantiomers of **A-1** in CH_2Cl_2 solution at 23 °C obtained by division of the CD in mdeg by the absorption of the same sample. Subscript **a** and **b** denote the respective HPLC fraction (see **Figure 53**).

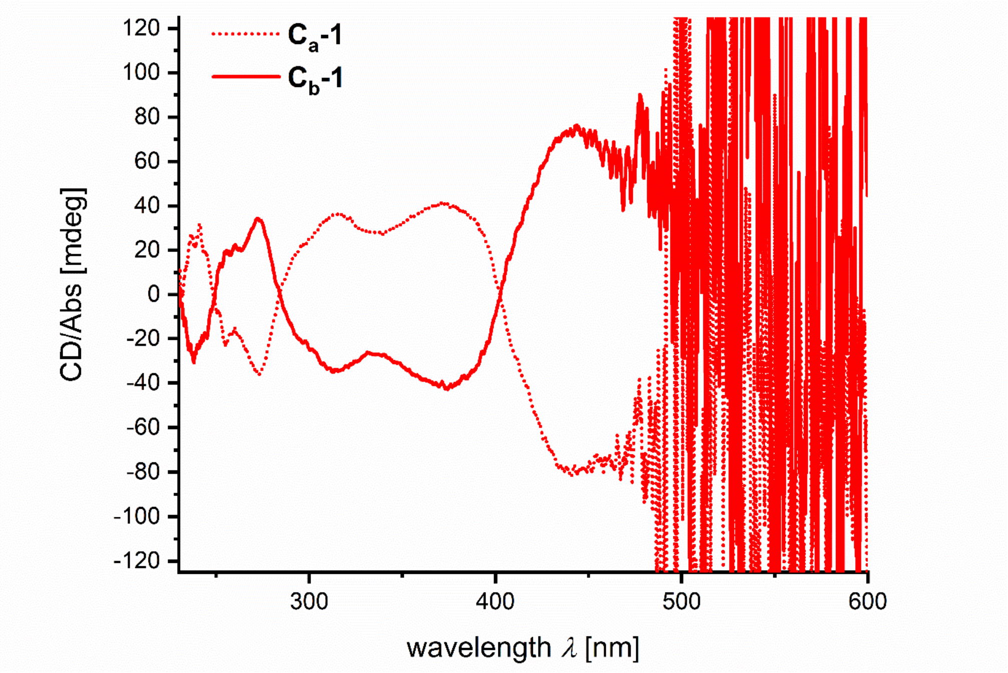

Figure 66 *g*-Factors for enantiomers of **C-1** in CH_2Cl_2 solution at 23 °C obtained by division of the CD in mdeg by the absorption of the same sample. Subscript **a** and **b** denote the respective HPLC fraction (see **Figure 54**).

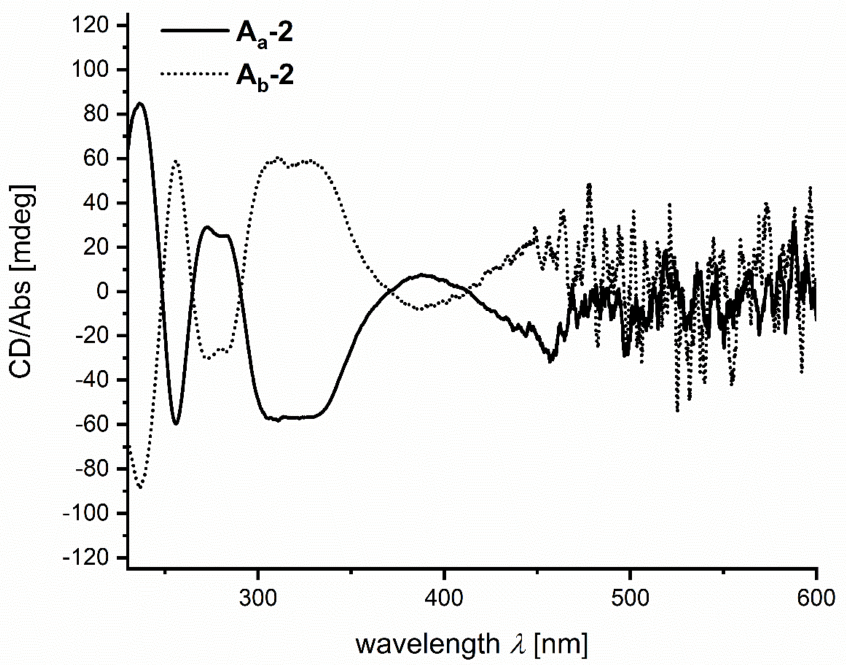

Figure 67 *g*-Factors for enantiomers of **A-2** in CH_2Cl_2 solution at 25 °C obtained by division of the CD in mdeg by the absorption of the same sample. Subscript **a** and **b** denote the HPLC fraction (see **Figure 55**).

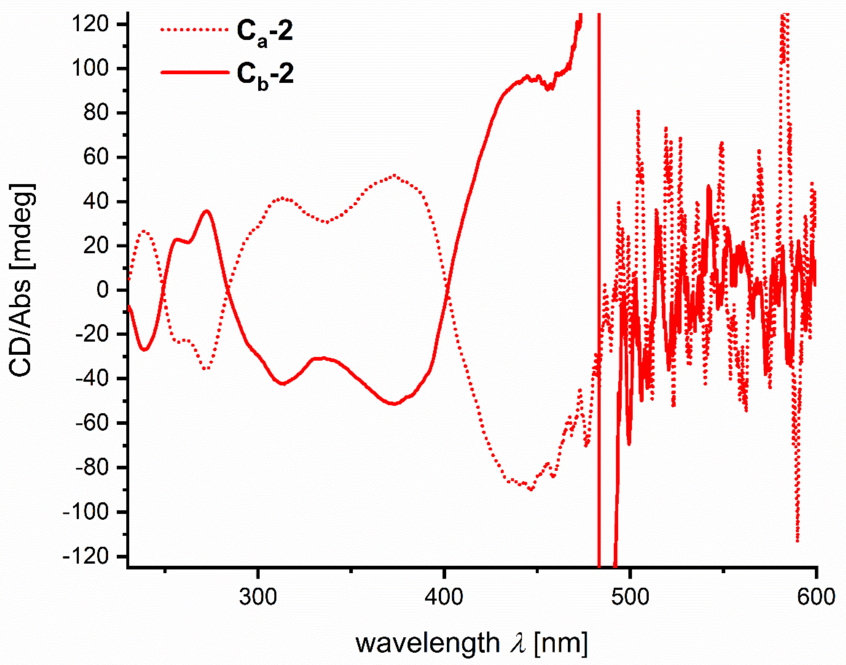

Figure 68 *g*-Factors for enantiomers of **C-2** in CH_2Cl_2 solution at 25 °C obtained by division of the CD in mdeg by the absorption of the same sample. Subscript **a** and **b** denote the respective HPLC fraction (see **Figure 56**).

ECD Photoisomerization Experiments

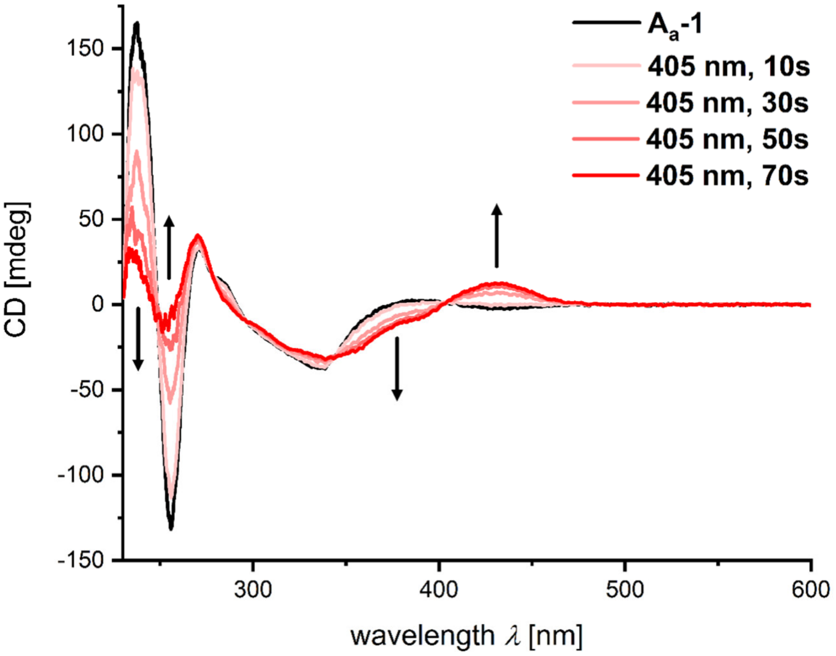

Figure 69 ECD spectra recorded during photoisomerization of enantiomerically pure **A$_a$-1** in CH_2Cl_2 solution at 25 °C (**a** denotes the HPLC fraction). Upon irradiation with 405 nm light at 25 °C, a mixture enriched in **C$_b$-1** was obtained. Therefore, **A$_a$-1** and **C$_b$-1** can be assigned to the same sulfoxide stereo configuration.

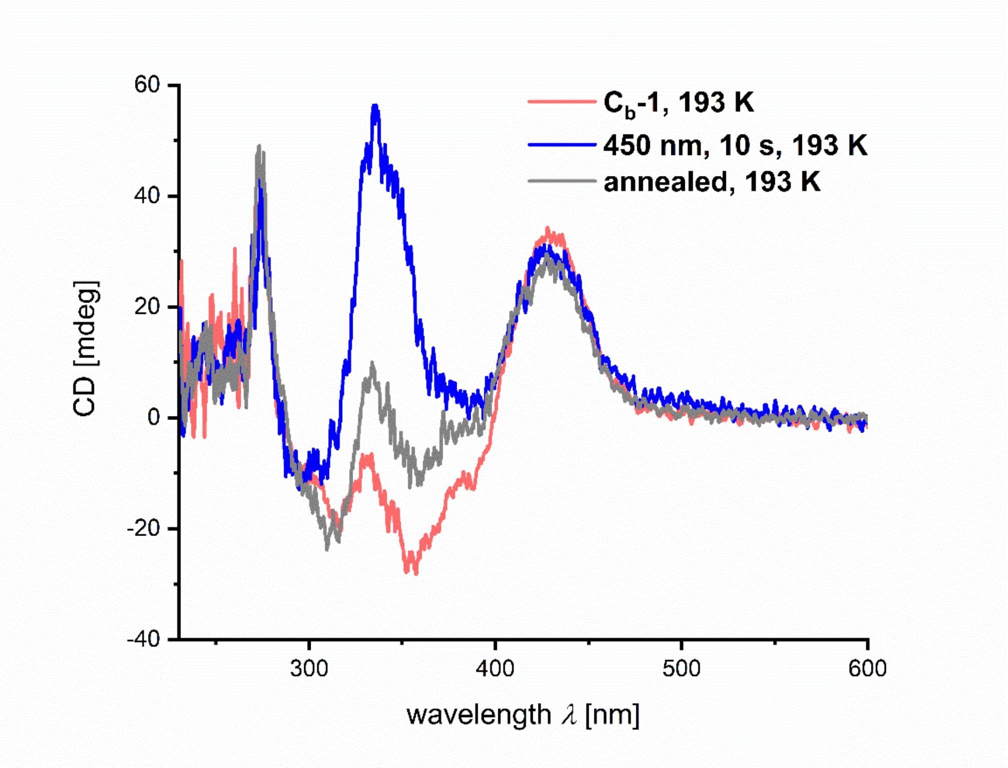

Figure 70 Low temperature ECD spectra recorded during photoisomerization of **C$_b$-1** in CH_2Cl_2 solution at −80 °C. A solution of enantiomerically pure **C$_b$-1** in CH_2Cl_2 (red spectrum) was irradiated with 450 nm light at −80 °C for 10 s (blue spectrum). The sample was annealed at 25 °C and another ECD spectrum was recorded at −80 °C (grey

spectrum). Upon irradiation, there is a strong change in ECD signal around 340 nm which could thus be ascribed to the metastable **D-1** isomer with inverted helicity as compared to **C_b-1**.

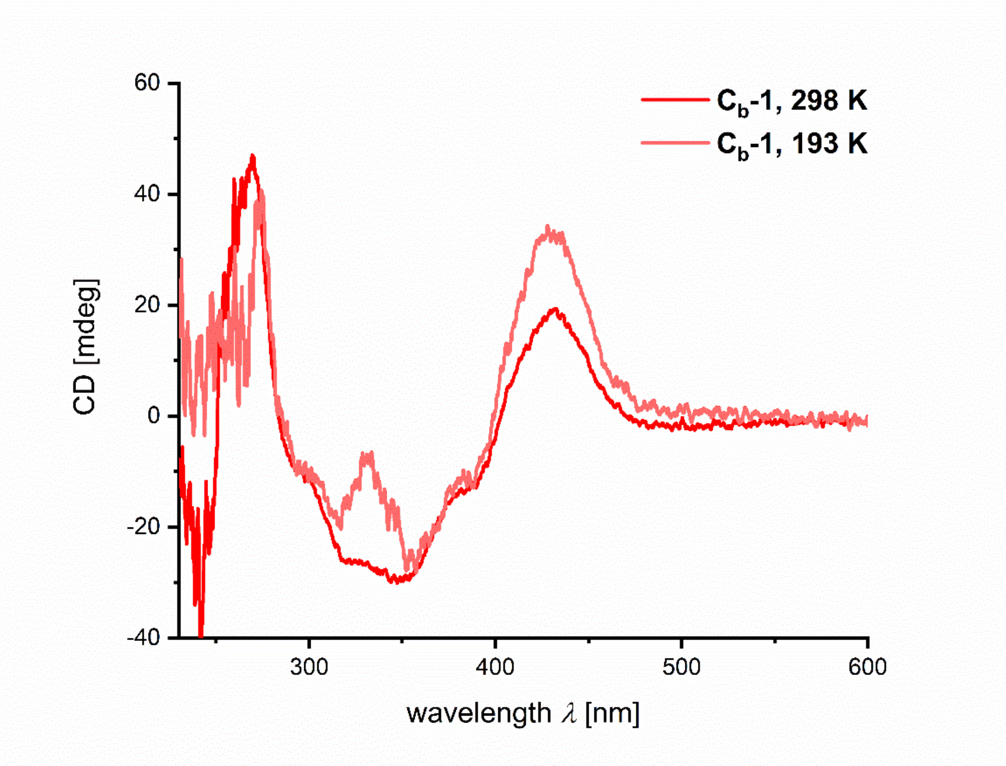

Figure 71 Comparison of ECD spectra of enantiomerically pure **C_b-1** in CH_2Cl_2 solution at 25 °C and −80 °C.

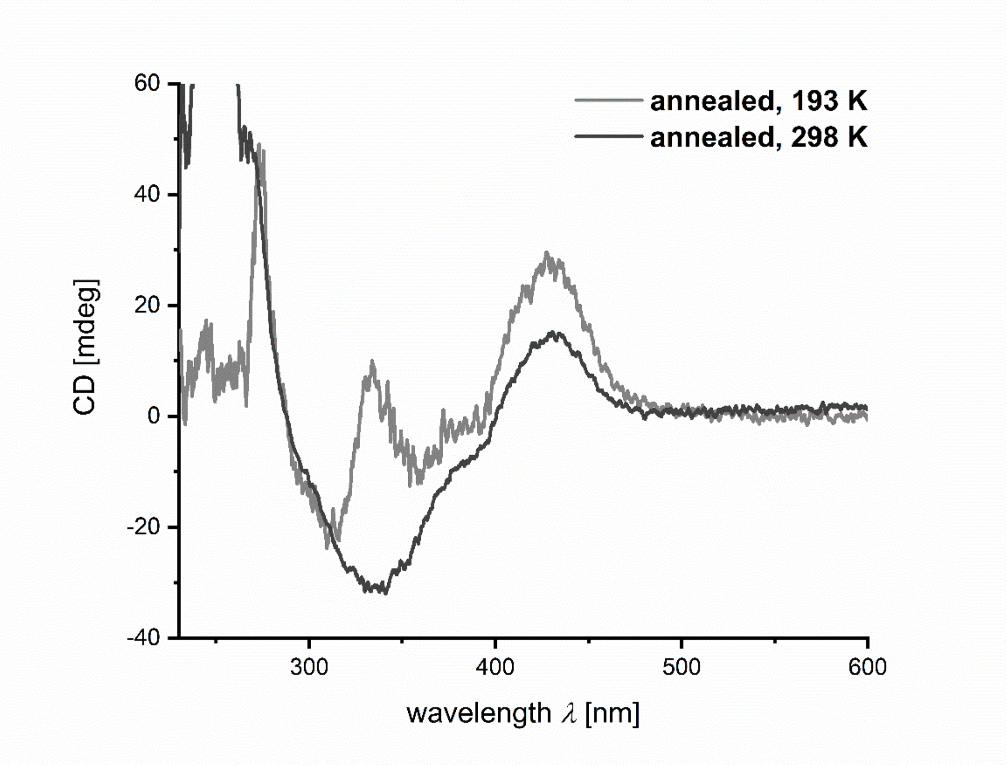

Figure 72 Comparison of ECD spectra obtained after irradiation of **C_b-1** in CH_2Cl_2 solution with 450 nm light at −80 °C followed by thermal annealing at 25 °C. Spectra were measured at −80 °C and 25 °C, respectively.

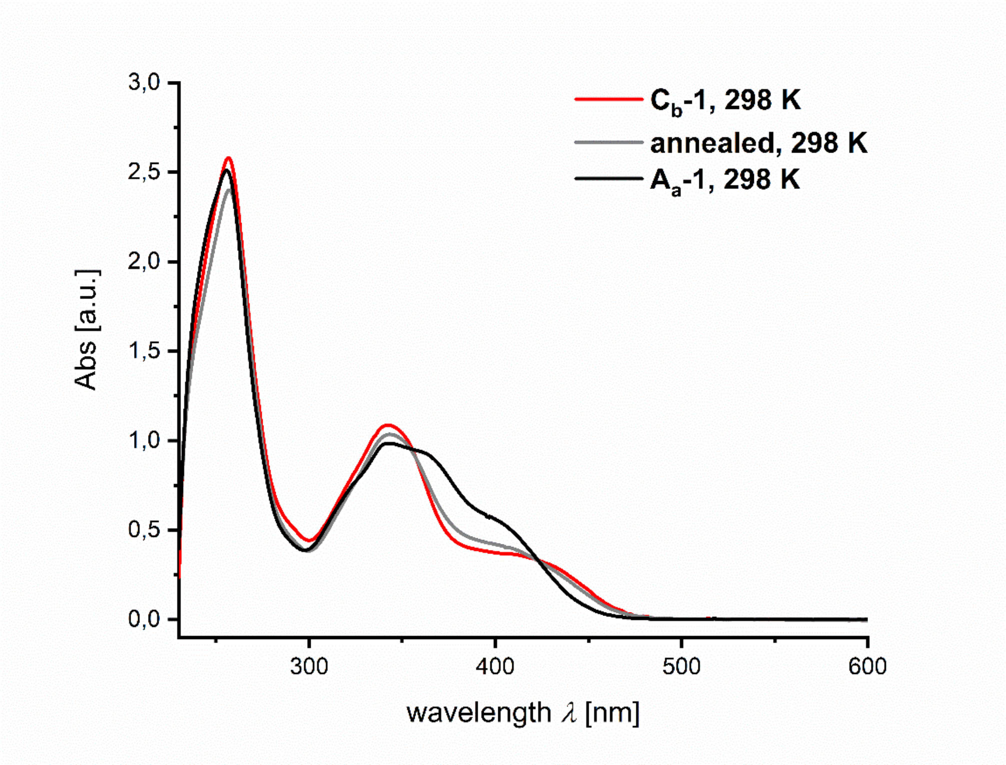

Figure 73 UV/Vis absorption spectra (recorded at 25 °C) corresponding to the low temperature ECD experiment described in **Figures 66-69**. The absorption spectrum of enantiomerically pure **A$_a$-1** was recorded separately and scaled to the isosbestic point at 423 nm. The absorption changes indicate a net conversion of **C$_b$-1** to **A$_a$-1** via transient D-1.

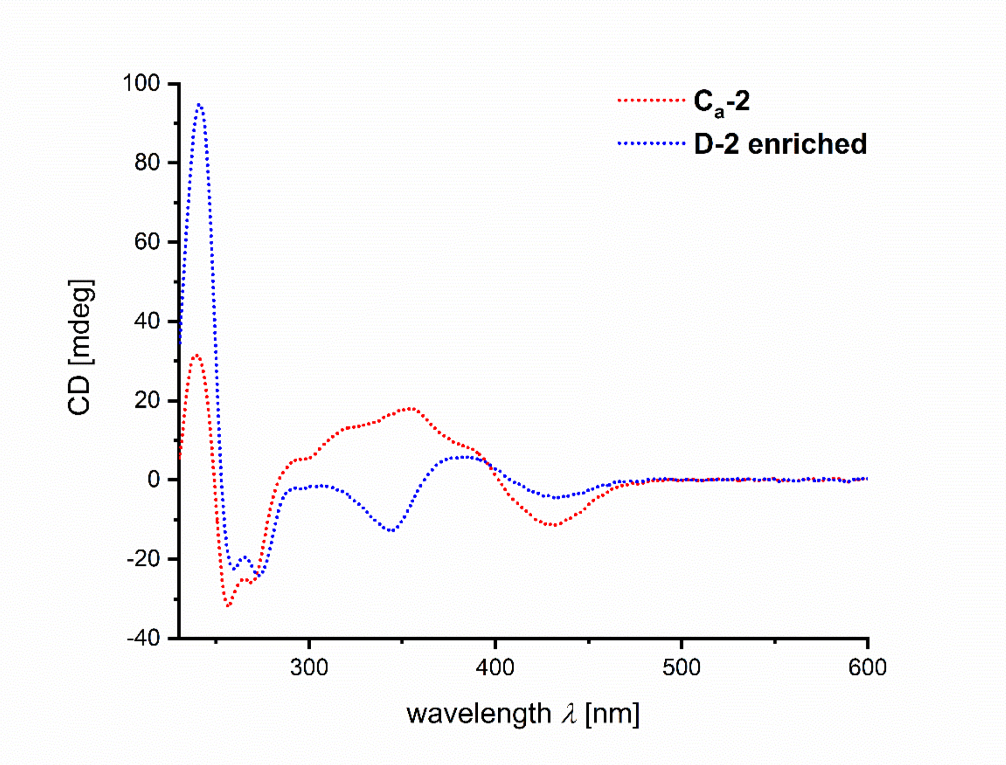

Figure 74 ECD spectra (recorded at 25 °C) of a solution of enantiomerically pure **C$_b$-2** in CH_2Cl_2 before (red spectrum) and after (blue spectrum) irradiation with 450 nm light at 25 °C. A significant change in ECD signal is

observed around 340 nm, which could thus be attributed to the formation of the respective **D-2** isomer with inverted helicity as compared to **C$_b$-2**.

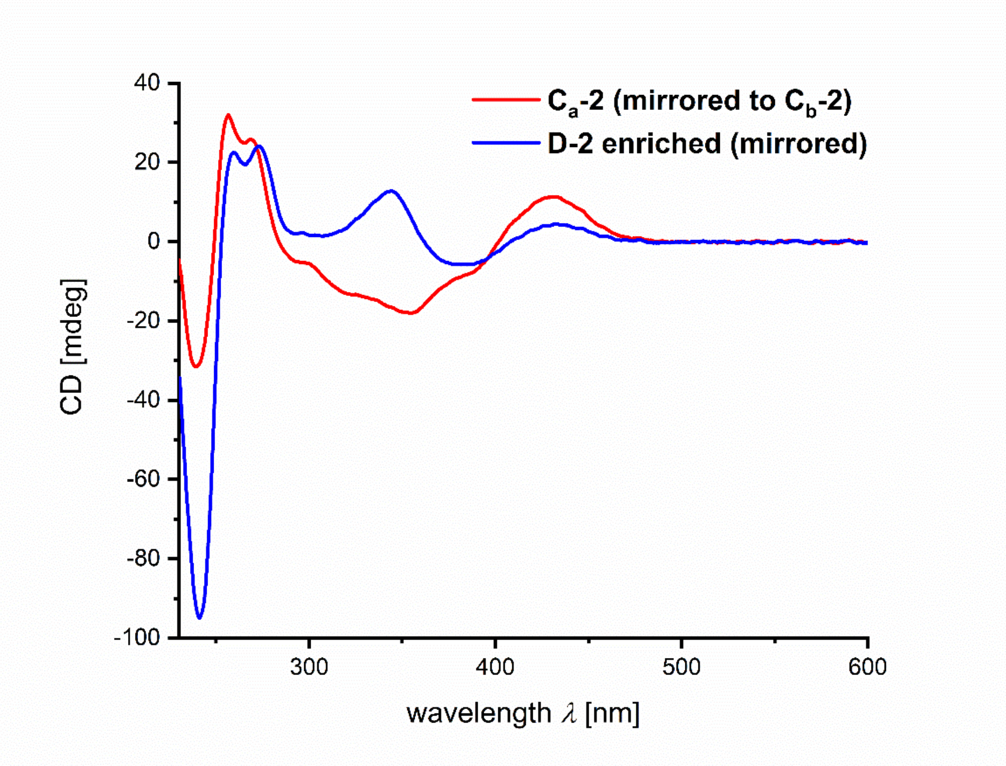

Figure 75 The experimentally obtained ECD spectra shown in **Figure 74** were mirrored by multiplication with −1 for direct comparison with other experiments as well as the theoretical description.

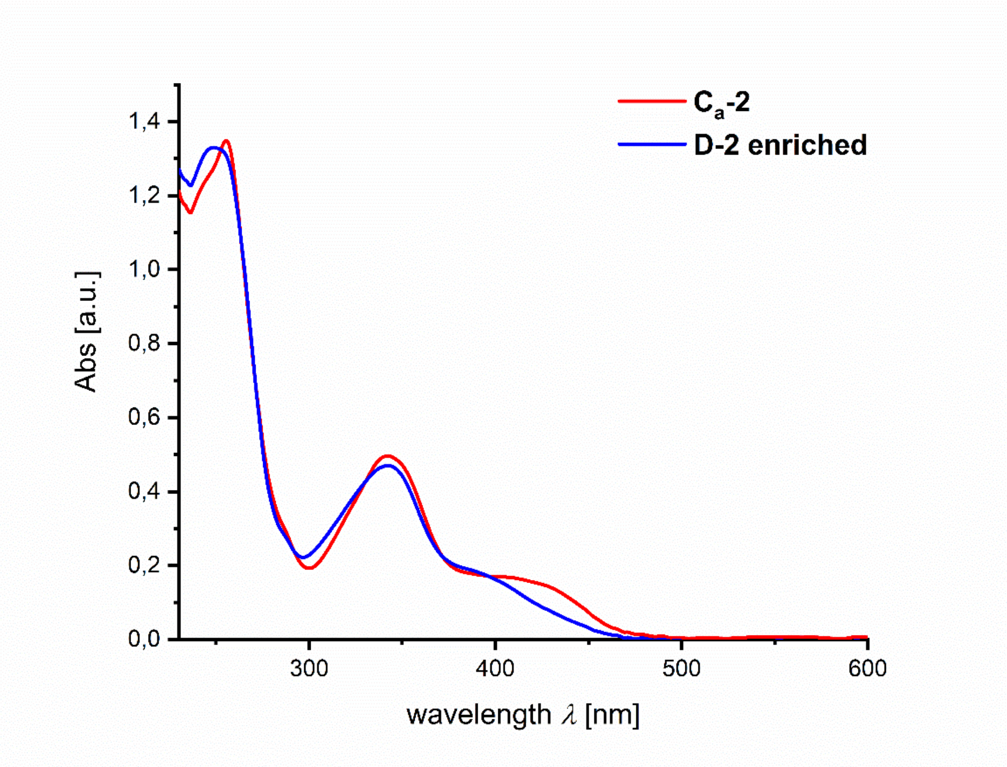

Figure 76 UV/Vis absorption spectra recorded at 25 °C corresponding to the photoisomerization experiment described in **Figure 74**.

Theoretical Description of Macrocyclic Motor 1

A conformational search was conducted for all four isomers of macrocyclic motor **1** with (*S*)-configured sulfoxide stereo center using the MacroModel software. The mixed torsional/low mode sampling was performed with an MMFF forcefield, $CHCl_3$ as the solvent, 1000 steps (100 per bond) and an energy window of 40 kJ mol^{-1}. The initial configurations of motor isomers **A-1**, **B-1**, **C-1**, **D-1** were generated from a crystal structure of **C-1** by manual rotation around the central double bond in the Schrödinger Maestro software. The crystal structure does not qualify of publication because of its low quality and therefore is not included here. For metastable isomers (*S*)-**B-1** and (*S*)-**D-1** constraints were added in order to freeze the helical chirality. For each isomer, the 25 structures of lowest energy were kept as starting points for DFT calculations. The following calculations were all performed using the *Gaussian16* software package[6]. Geometries were optimized at the B3LYP-D3BJ/6-31G(d) IEFPCM (CH_2Cl_2) level of theory followed by optimization with the 'tight' keyword at the B3LYP-D3BJ/6-311G(d,p) IEFPCM (CH_2Cl_2) level of theory. (In a small number of cases the calculations failed to converge and the structures were not considered in the following). Frequency analysis at the same level of theory confirmed the structures to be local minima, as no imaginary modes were obtained. For all calculations using Gaussian, the integration grid was set to ultrafine.

Ground State Geometries

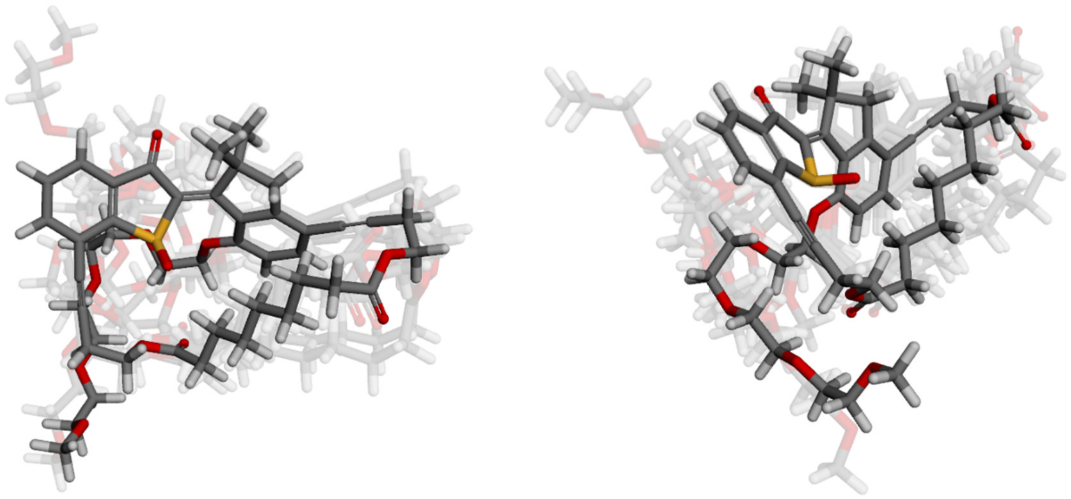

Figure 77 Overlay of significant minimum structures obtained for (*S*)-**A-1** at the B3LYP-D3BJ/6-311G(d,p) IEFPCM (CH_2Cl_2) level of theory. The energy window is 0-2.63 kcal mol^{-1}. The most stable structure is shown with full opacity. Two different viewing angles are shown. Note that there is no "pre-threading" observed in any of these structures, i.e. the TEG chain always resides outside of the macrocycle.

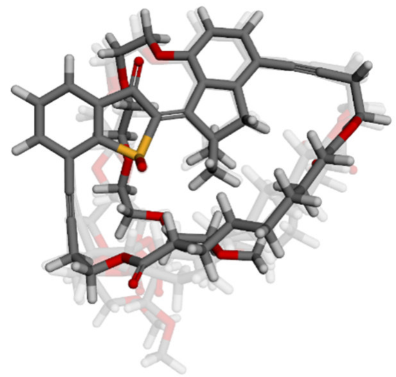

Figure 78 Overlay of significant minimum structures obtained for (*S*)-**B-1** at the B3LYP-D3BJ/6-311G(d,p) IEFPCM (CH_2Cl_2) level of theory. The energy window is 0-3.24 kcal mol^{-1}. The most stable structure is sown with full opacity. Note that there is no "pre-threading" observed, i.e. the TEG chain always resides outside of the macrocyle.

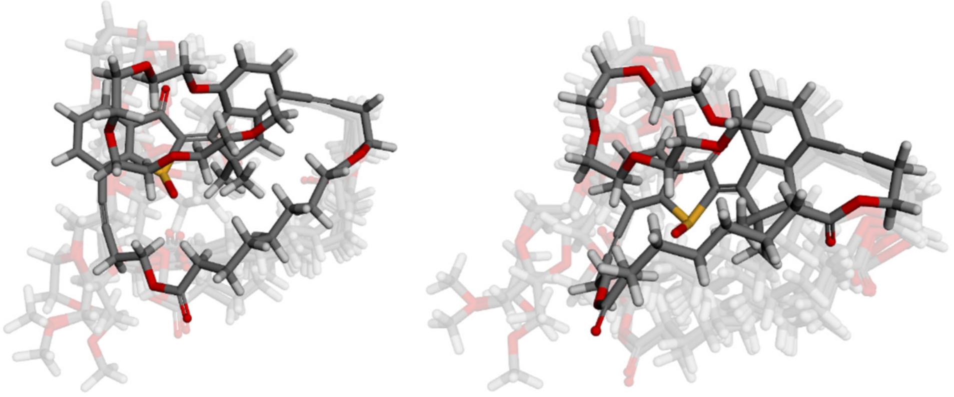

Figure 79 Overlay of significant minimum structures obtained for (*S*)-**C-1** at the B3LYP-D3BJ/6-311G(d,p) IEFPCM (CH_2Cl_2) level of theory. The energy window is 0-3.26 kcal mol^{-1}. The most stable structure is shown with full opacity. Two different viewing angles are shown. Note that there is no "pre-threading" observed, i.e. the TEG chain always resides outside of the macrocycle.

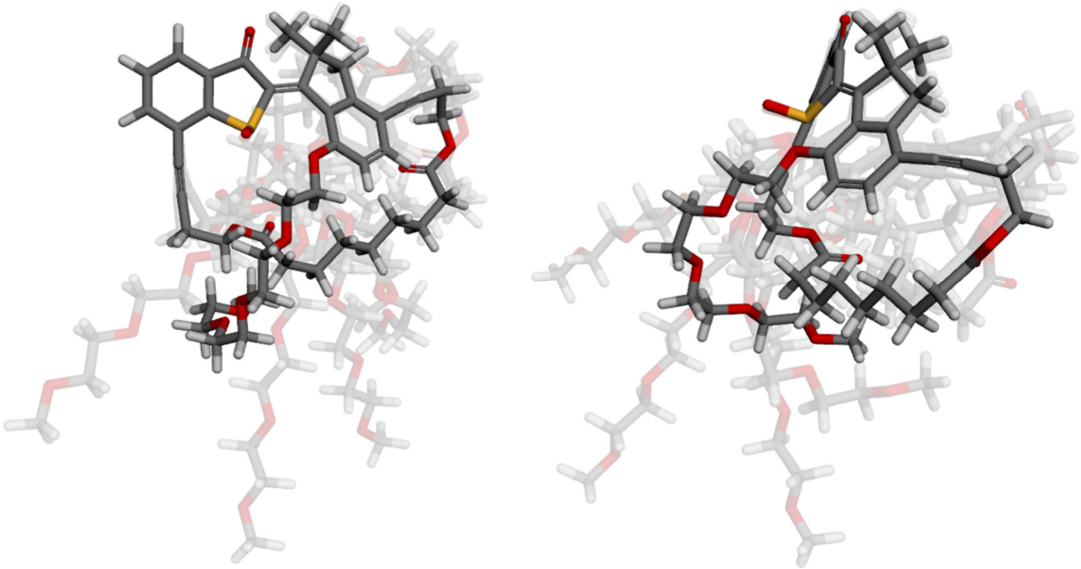

Figure 80 Overlay of significant minimum structures obtained for (*S*)-**D-1** at the B3LYP-D3BJ/6-311G(d,p) IEFPCM (CH_2Cl_2) level of theory. The energy window is 0-3.18 kcal mol^{-1}. The most stable structure is shown with full opacity. Two different viewing angles are shown. Note that there is no "pre-threading" observed in any of these structures, i.e. the TEG chain always resides outside of the macrocycle.

Theoretically Obtained ECD Spectra

ECD spectra were calculated for the lowest-energy ensembles for each respective isomer (see above) using TD-B3LYP-D3BJ/6-311+G(d,p) IEFPCM (CH_2Cl_2). 30 states were calculated for each conformer. An ultrafine integration grid was used. The spectra were additionally Boltzmann-averaged according to the *Gibbs* free energy difference obtained from frequency analysis. Simulation of the theoretically obtained ECD spectra was done using *SpecDis* (V1.71)[7].

Comparison of calculated and experimental ECD spectra

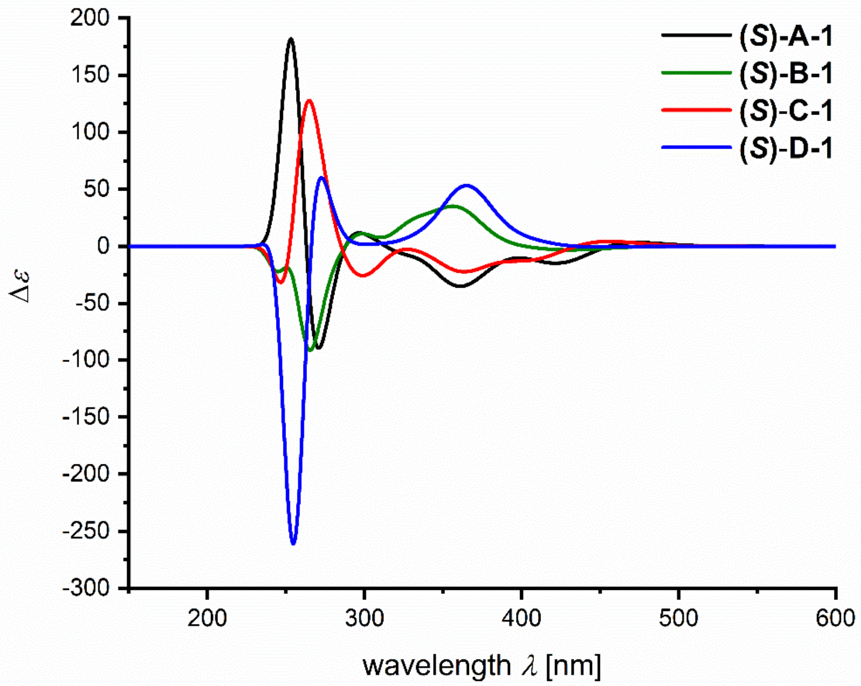

Figure 81 Boltzmann-weighted ECD spectra for (*S*)-configured motor **1** obtained on the TD-B3LYP-D3BJ/6-311+G(d,p) IEFPCM (CH_2Cl_2) level of theory. Spectra were simulated in SpecDis with a sigma value of 0.2 eV and no UV-shift.

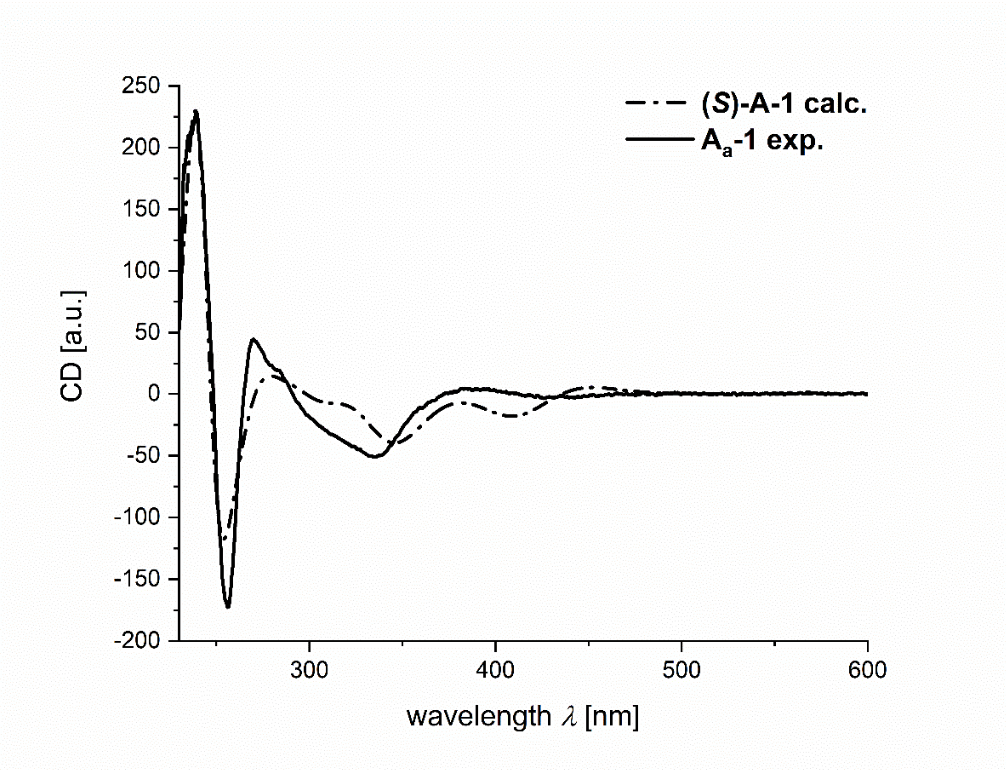

Figure 82 Comparison of experimental (solid line) and theoretically (dotted line) ECD spectra of (*S*)-**A-1**. The subscript **a** denotes the respective HPLC fraction (see **Figure 53**). The theoretical spectrum was blue-shifted by 15 nm and a sigma value of 0.17 eV was used.

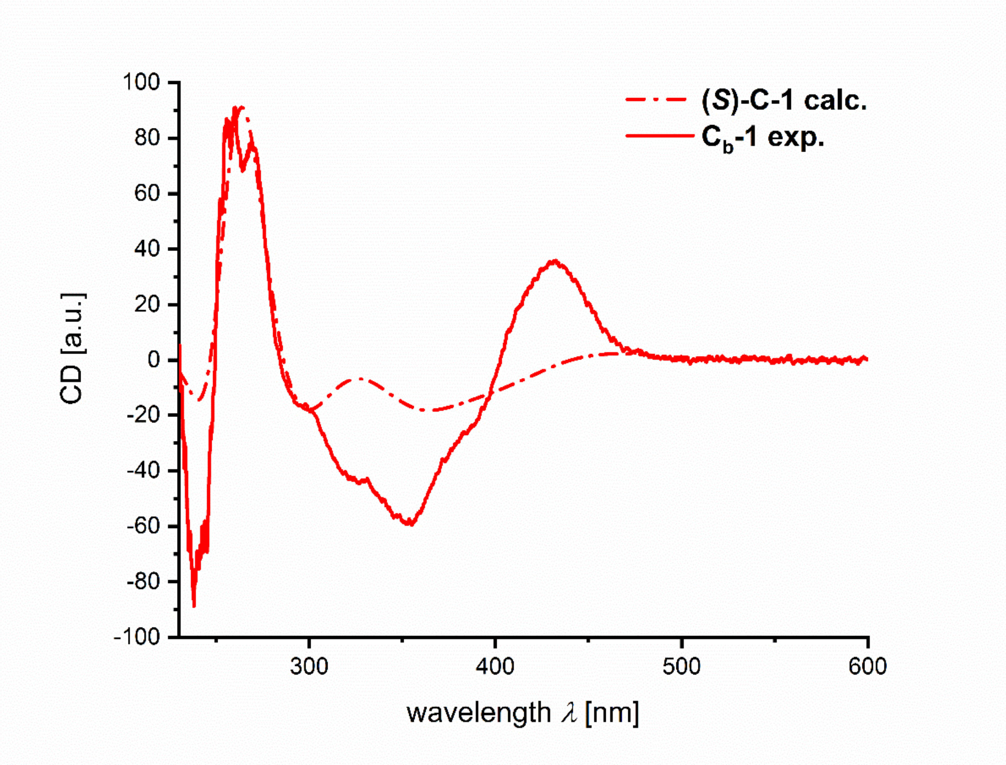

Figure 83 Comparison of experimental (solid line) and theoretically obtained (dotted line) ECD spectra of (*S*)-**C-1**. The subscript **b** denotes the respective HPLC fraction (see **Figure 54**). The theoretical spectrum was blue-shifted by 2 nm and a sigma value of 0.27 eV was used.

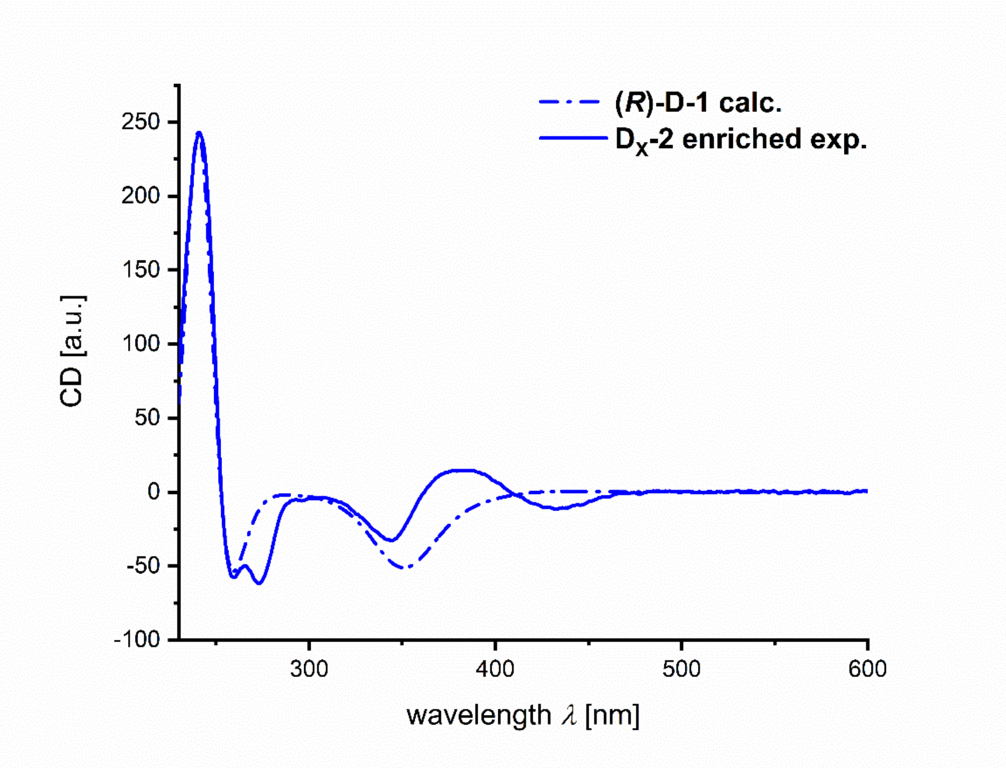

Figure 84 Comparison of theoretically obtained (dotted line) ECD spectrum of (*R*)-**D-1** with the experimentally obtained spectrum of a (*R*)-configured mixture enriched in **D-2** (blue line). The theoretical spectrum was generated from the enantiomeric spectrum of (*S*)-configured **D-1** by multiplication with −1 and was blue-shifted by 14 nm. A sigma value of 0.21 eV was used. For the experimental spectrum, a solution of enantiopure **C$_a$-2** in CH_2Cl_2 was irradiated with 450 nm light for 30 s.

Pre-threaded Structure A_i-1

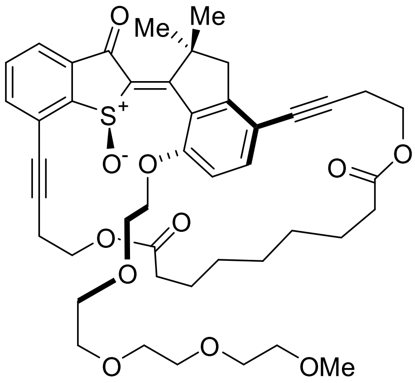

Figure 85 Threaded structure **A_i-1** ((*Z,S,P*)-configuration). Unlike in structure **A_{ii}-1**, the TEG-chain is in a "threaded" state.

In order to gain insight into possible preformed “threaded” states, structure **A_i-1** was optimized at the B3LYP-D3BJ/6-311G(d,p) IEFPCM (CH_2Cl_2) level of theory. The threaded starting structure **A_i-1** was created by manual modification of a **D-1** structure, followed by a minimization with the force field integrated in Maestro. It was then employed as a starting structure in conformational searches using Macro Model. However, even with constraints on the TEG chain, only conformers of **A_{ii}-1** were found as a result of that search. From this observation, and the obtained energy difference of 16.5 kcal mol^{-1} between the optimized **A_i-1** geometry and the lowest **A_{ii}-1** conformer, we conclude that structures of “pre-threaded” type are energetically highly unfavorable. This is also in agreement with the experimental observation of an unusually high *Gibbs* energy of activation for the thermal **D-1** to **A-1** isomerization (16.4 kcal mol^{-1} vs 5.8 kcal mol^{-1} for the analogous process for the first generation HTI motor[8]), as the isomerization process is expected to proceed with simultaneous threading of the TEG chain through the macrocycle.

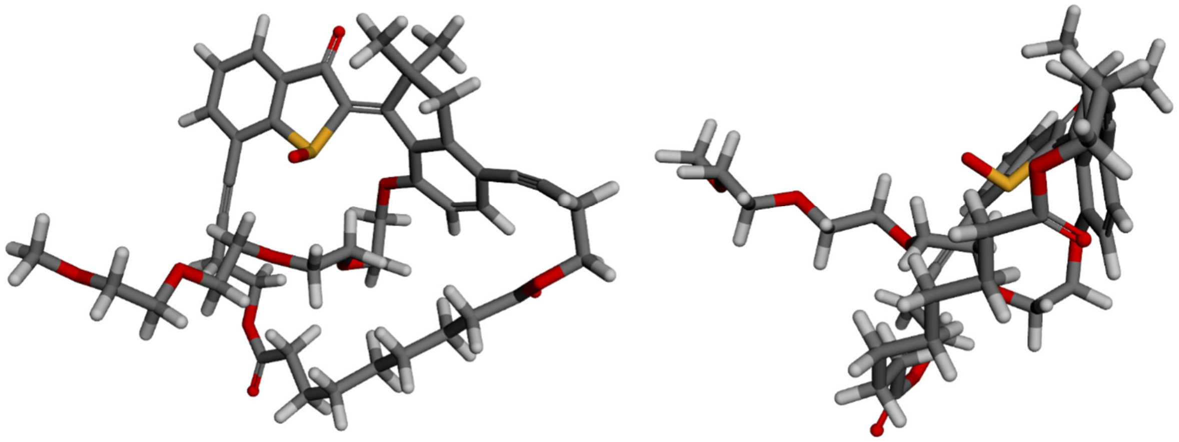

Figure 86 Ground state geometry obtained for **A_i-1** at the B3LYP-D3BJ/6-311G(d,p) IEFPCM (CH_2Cl_2) level of theory. Front and side view are shown to illustrate threading of the TEG chain.

XYZ-Data

xyz-Files are available online as a .zip-archive. The numbering and relative energies of conformers are given in Table **2**.

Table **2** Conformer numbering and respective energies obtained at the B3LYP-D3BJ/6-311G(d,p) IEFPCM(CH_2Cl_2) level of theory.

Isomer	Conformer	Energy (Hartree)	Rel. Energy (Hartree)	Rel. Energy (kcal mol^{-1})
**A-1**	1	-2972.922507	0.002466	+1.55
	2	-2972.924973	0	0
	3	-2972.922966	0.002007	+1.26
	4	-2972.921515	0.003458	+2.17
	5	-2972.920779	0.004194	+2.63
	6	-2972.921723	0.00325	+2.04
	7	-2972.920778	0.004195	+2.63
	8	-2972.924462	0.000511	+0.32
	threaded	-2972.898754	0.026219	+16.45
**B-1**	1	-2972.923927	0	0
	2	-2972.920398	0.003529	+2.21
	3	-2972.918766	0.005161	+3.24
	4	-2972.922089	0.001838	+1.15
**C-1**	1	-2972.919637	0.000218	+0.14
	2	-2972.91795	0.001905	+1.2
	3	-2972.916543	0.003312	+2.08
	4	-2972.915907	0.003948	+2.48
	5	-2972.919432	0.000423	+0.27
	6	-2972.917951	0.001904	+1.19
	7	-2972.918253	0.001602	+1.01
	8	-2972.919855	0	0
	9	-2972.914663	0.005192	+3.26
	10	-2972.914794	0.005061	+3.18
	11	-2972.915191	0.004664	+2.93
**D-1**	1	-2972.905719	0.00507	+3.18
	2	-2972.90814	0.002649	+1.66
	3	-2972.910789	0	0
	4	-2972.908917	0.001872	+1.17
	5	-2972.908782	0.002007	+1.26

Crystal Structure Data of *(E)*-5

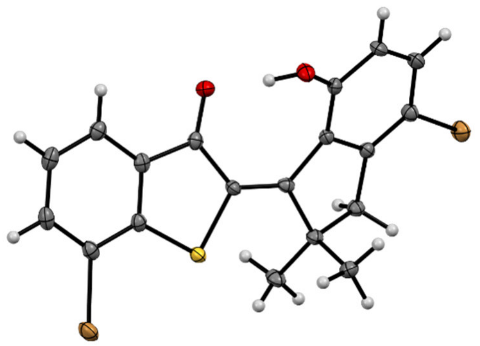

Compound	**(*E*)-5 (xv390)**<br>CCDC 2123711
net formula	$C_{19}H_{14}Br_2O_2S$
M_r/g mol^{-1}	466.18
crystal size/mm	0.100 × 0.070 × 0.020
T/K	106.(2)
radiation	MoKα
diffractometer	'Bruker D8 Venture TXS'
crystal system	triclinic
space group	'P -1'
a/Å	8.2363(5)
b/Å	8.3643(5)
c/Å	12.1540(8)
α/°	93.051(2)
β/°	93.362(2)
γ/°	95.150(2)
V/Å^3	831.04(9)
Z	2
calc. density/g cm^{-3}	1.863
μ/mm^{-1}	5.012
absorption correction	Multi-Scan
transmission factor range	0.74–0.91
refls. measured	14942
R_{int}	0.0425
mean σ(*I*)/*I*	0.0411
θ range	2.888–28.279
observed refls.	3535
x, y (weighting scheme)	0.0148, 0.6424
hydrogen refinement	H(C) constr, H(O) refall
refls in refinement	4086
parameters	223
restraints	0
$R(F_{obs})$	0.0263
$R_w(F^2)$	0.0632
S	1.033
shift/error$_{max}$	0.001
max electron density/e Å^{-3}	0.431
min electron density/e Å^{-3}	−0.325

NMR Spectra of Synthetic Intermediates

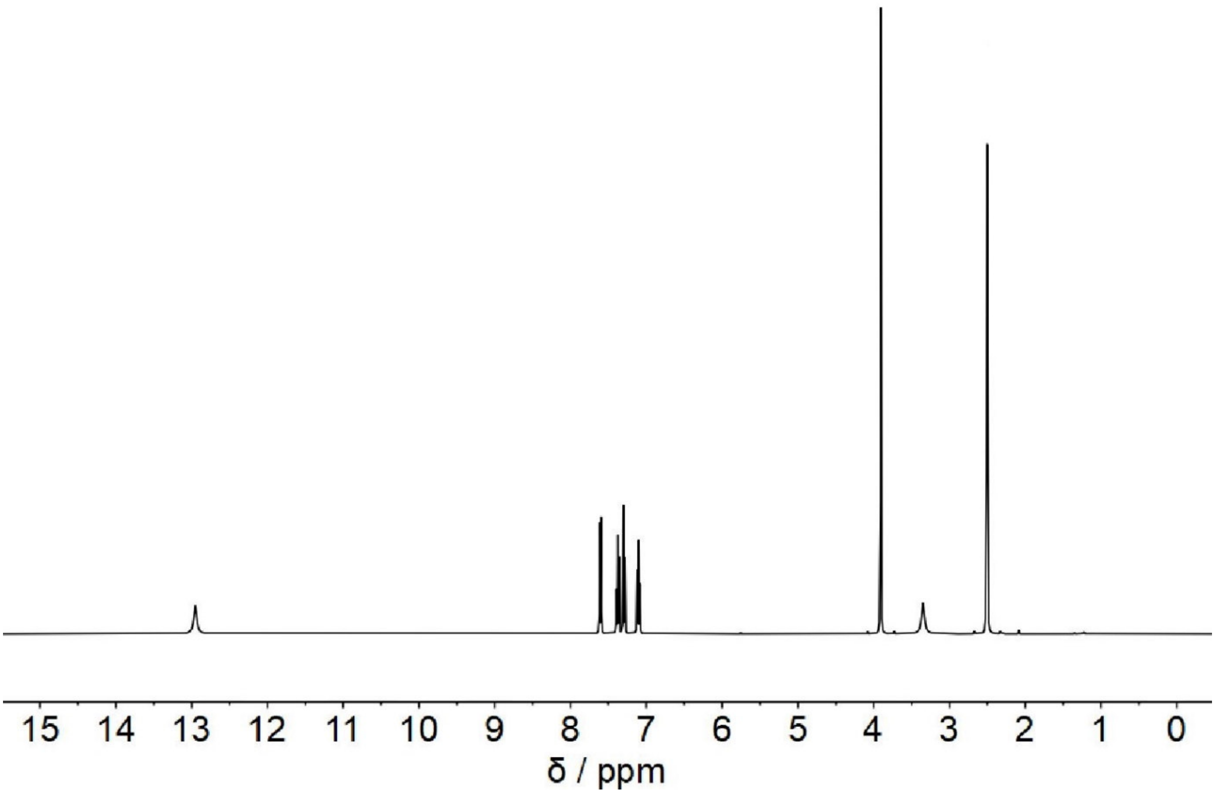

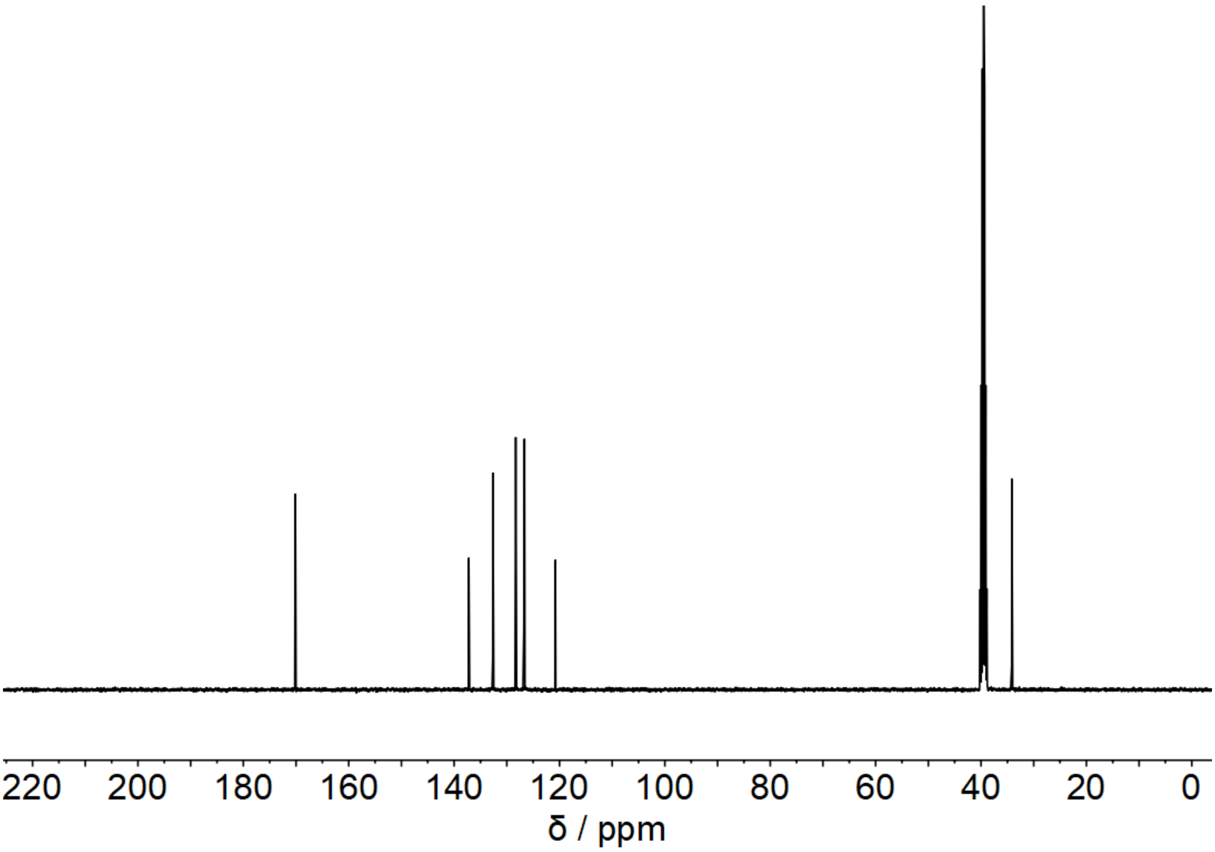

Figure 87 NMR spectra of compound **S1** (400 MHz, $(CD_3)_2SO$): ^{1}H NMR spectrum (top) and ^{13}C NMR spectrum (bottom).

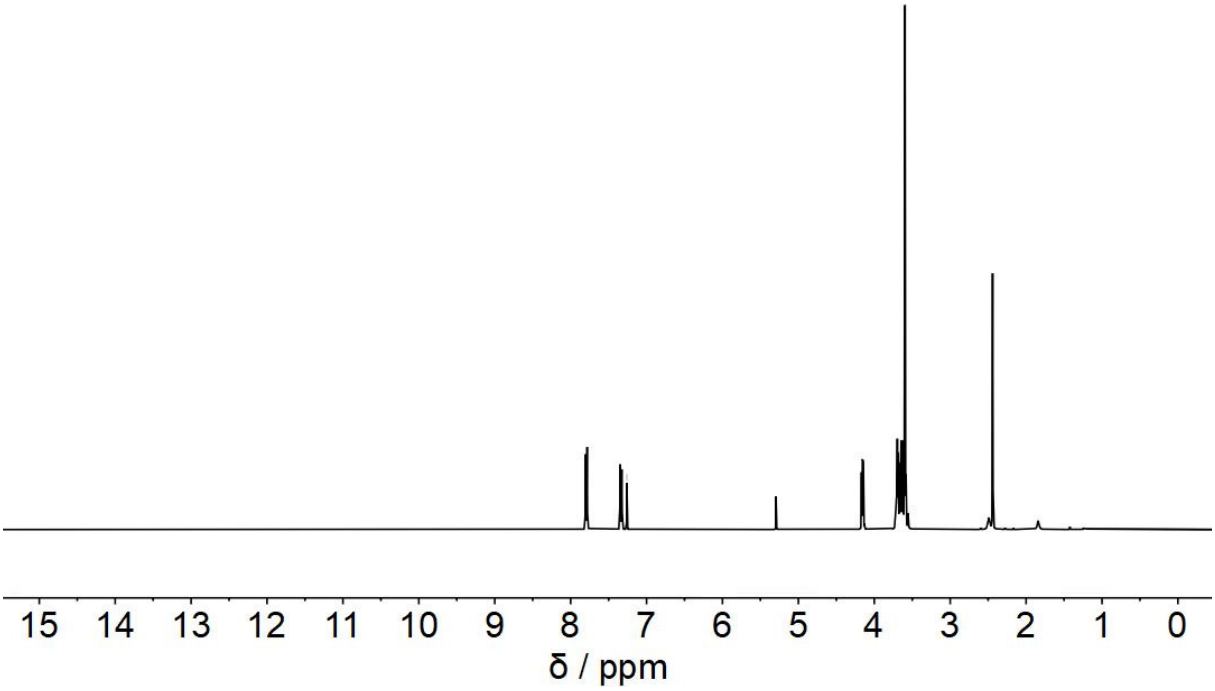

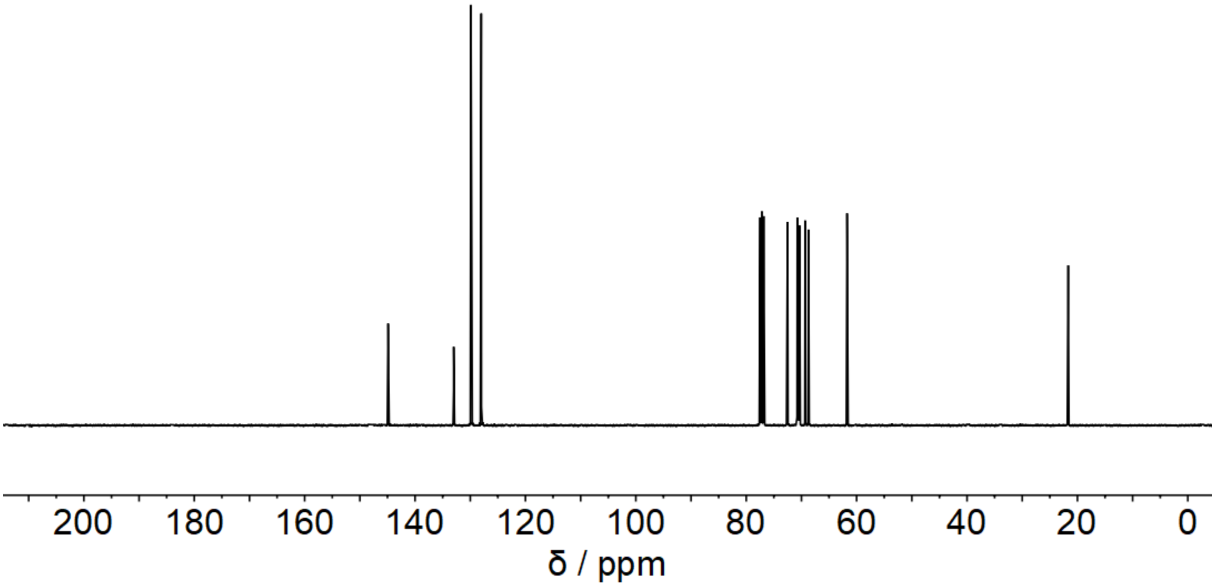

Figure 88 NMR spectra of compound **S2** (400 MHz, $CDCl_3$): 1H NMR spectrum (top) and 13C NMR spectrum (bottom).

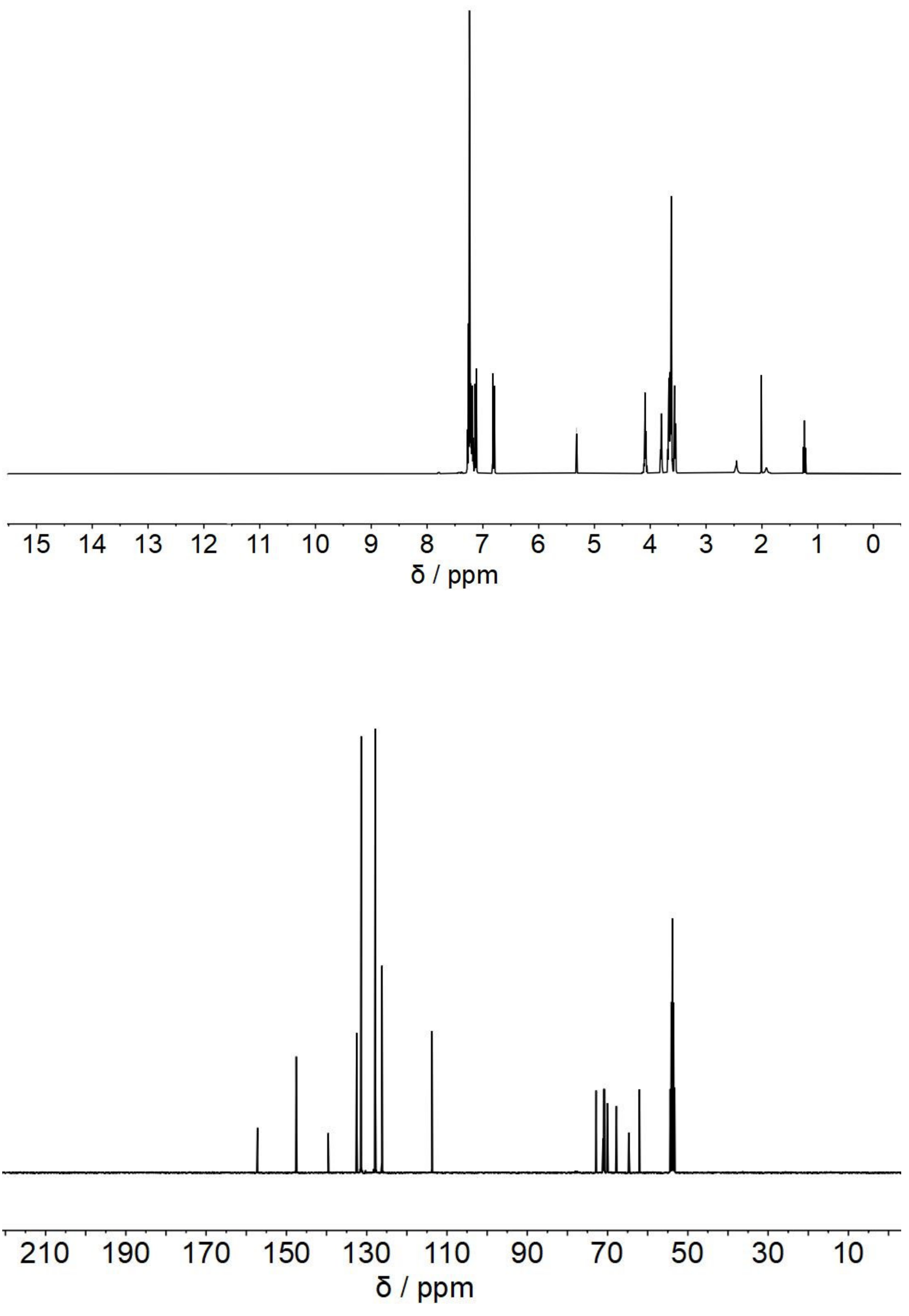

Figure 89 NMR spectra of compound **S3** (400 MHz, CD_2Cl_2): ^{1}H NMR spectrum (top) and ^{13}C NMR spectrum (bottom).

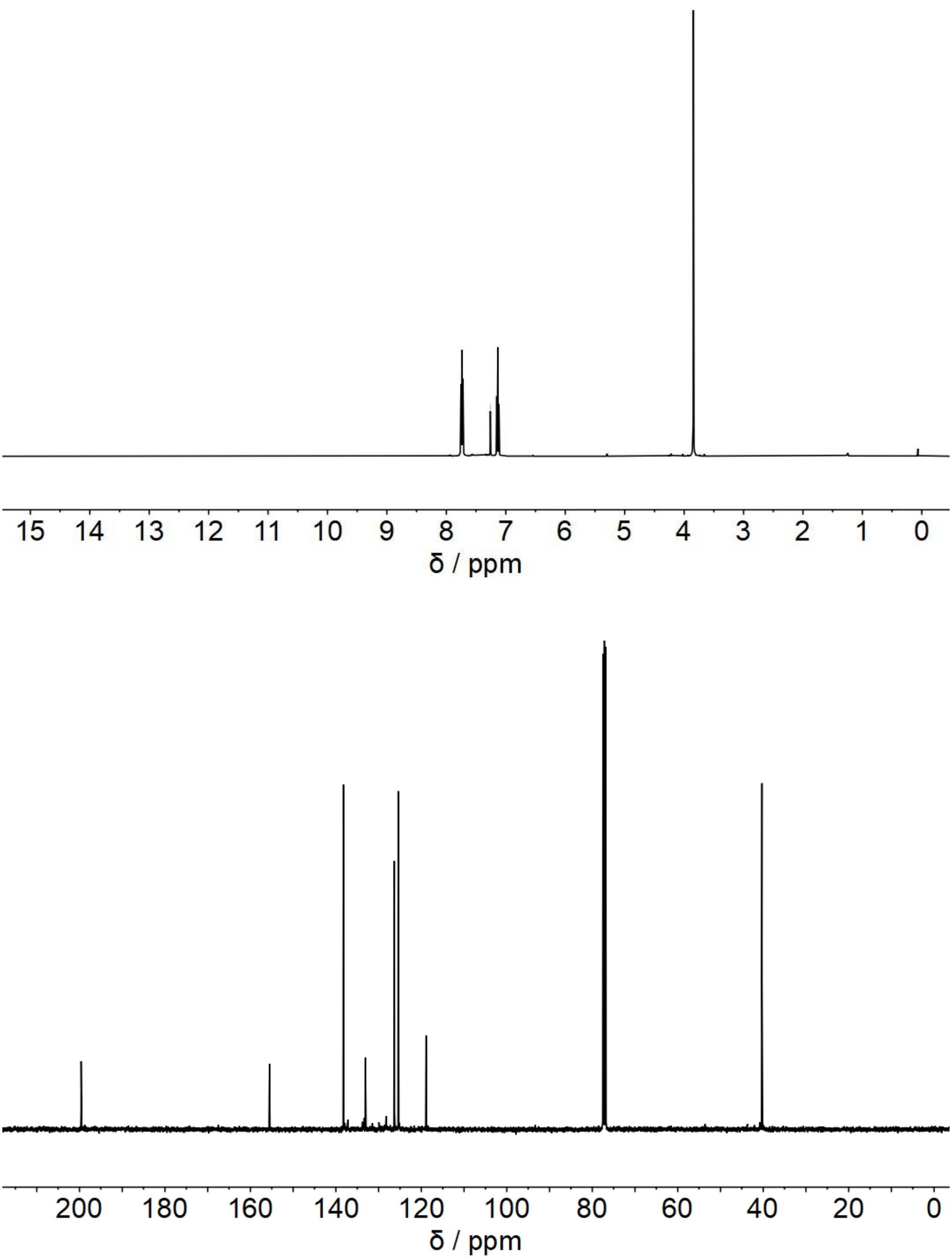

Figure 90 NMR spectra of compound **3** (400 MHz, $CDCl_3$): ^1H NMR spectrum (top) and ^{13}C NMR spectrum (bottom).

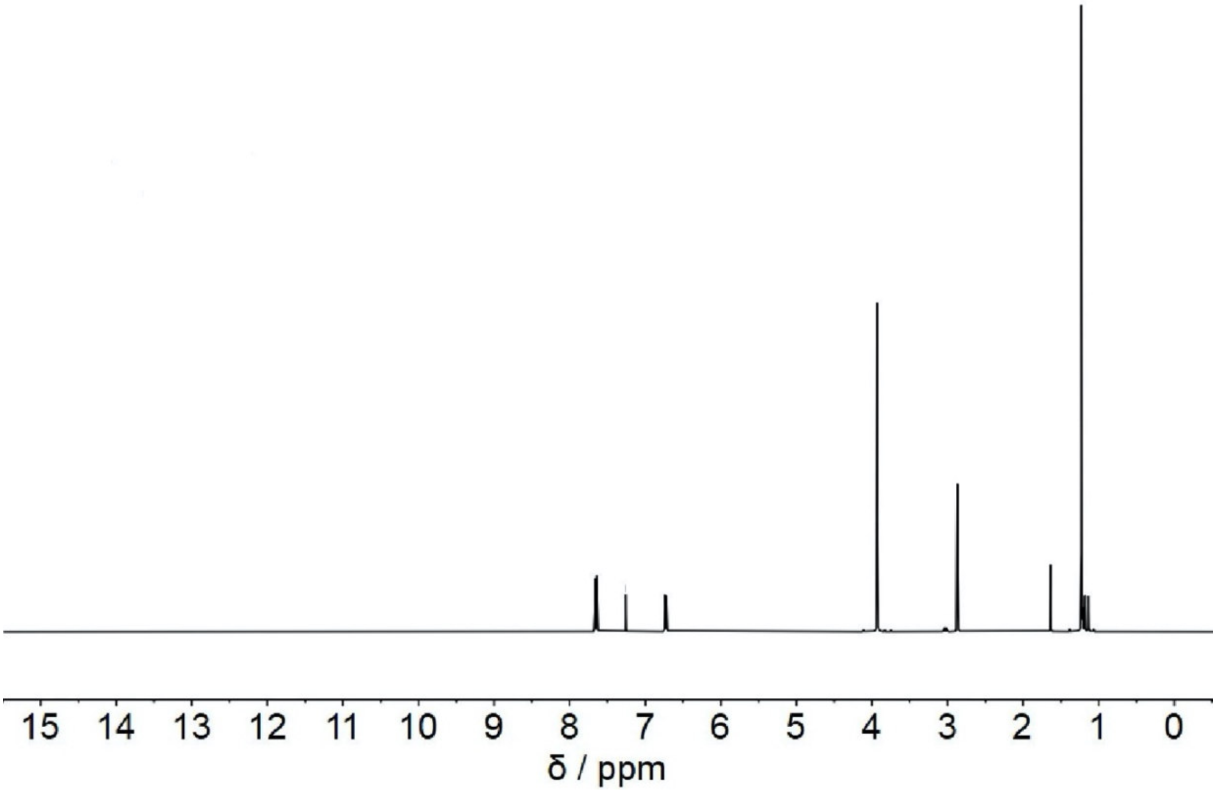

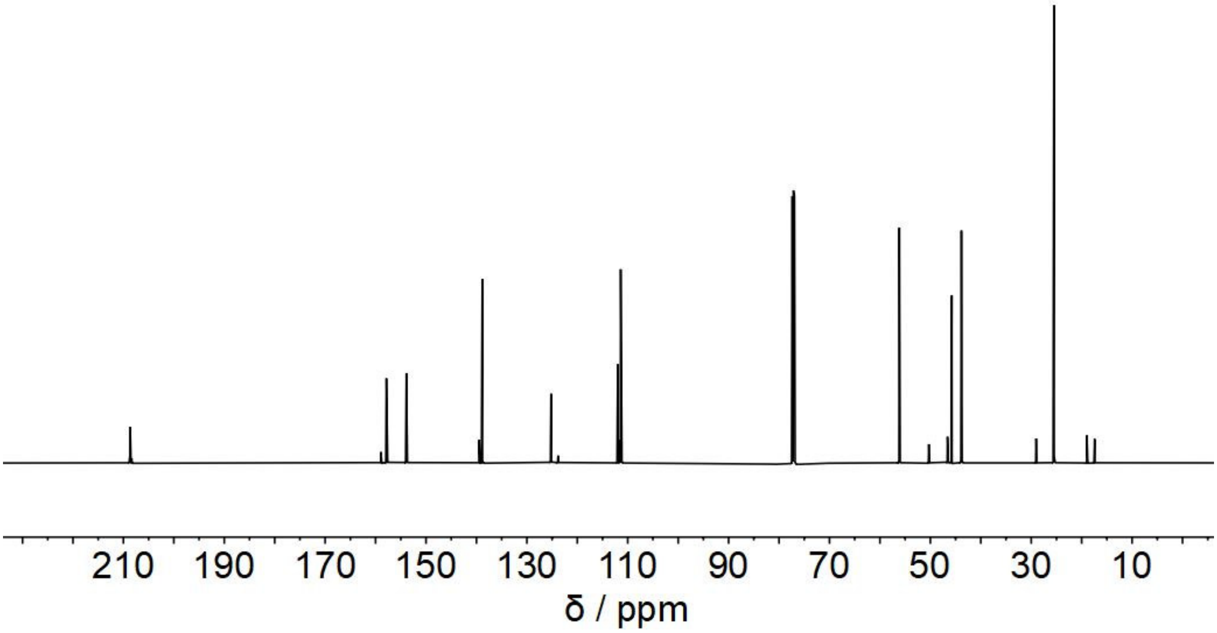

Figure 91 NMR spectra of compound **4** (599 MHz, $CDCl_3$): ^{1}H NMR spectrum (top) and ^{13}C NMR spectrum (bottom).

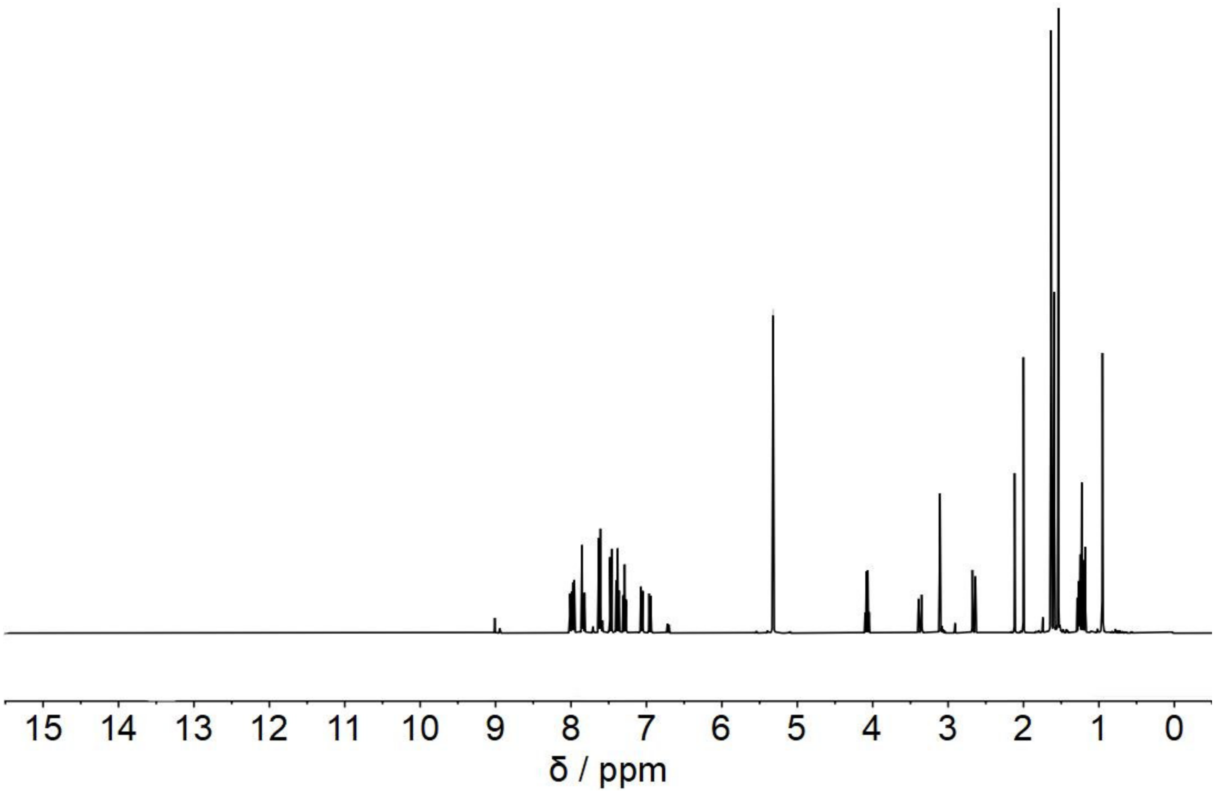

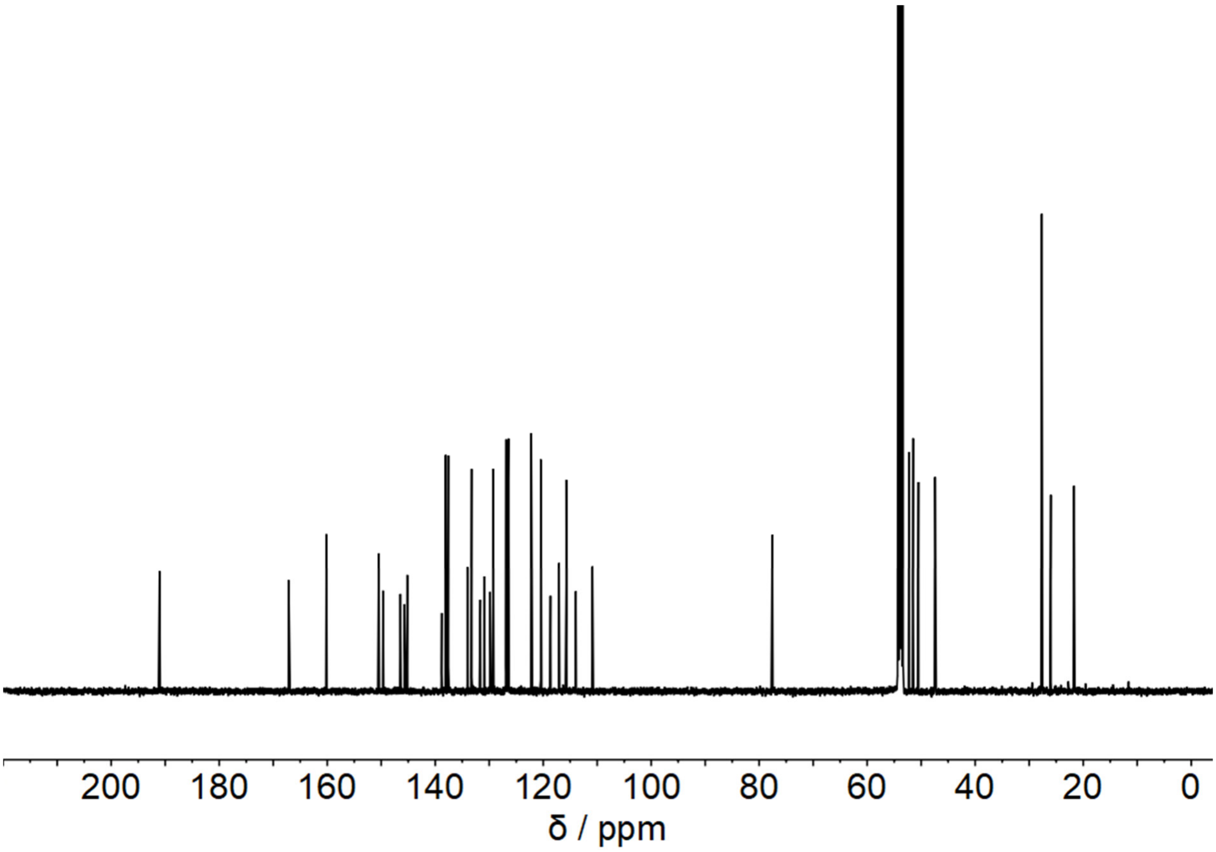

Figure 92 NMR spectra of compound **5** (mixture of (*E*)- and (*Z*)-isomers, 500 MHz, CD_2Cl_2): ^{1}H NMR spectrum (top) and ^{13}C NMR spectrum (bottom).

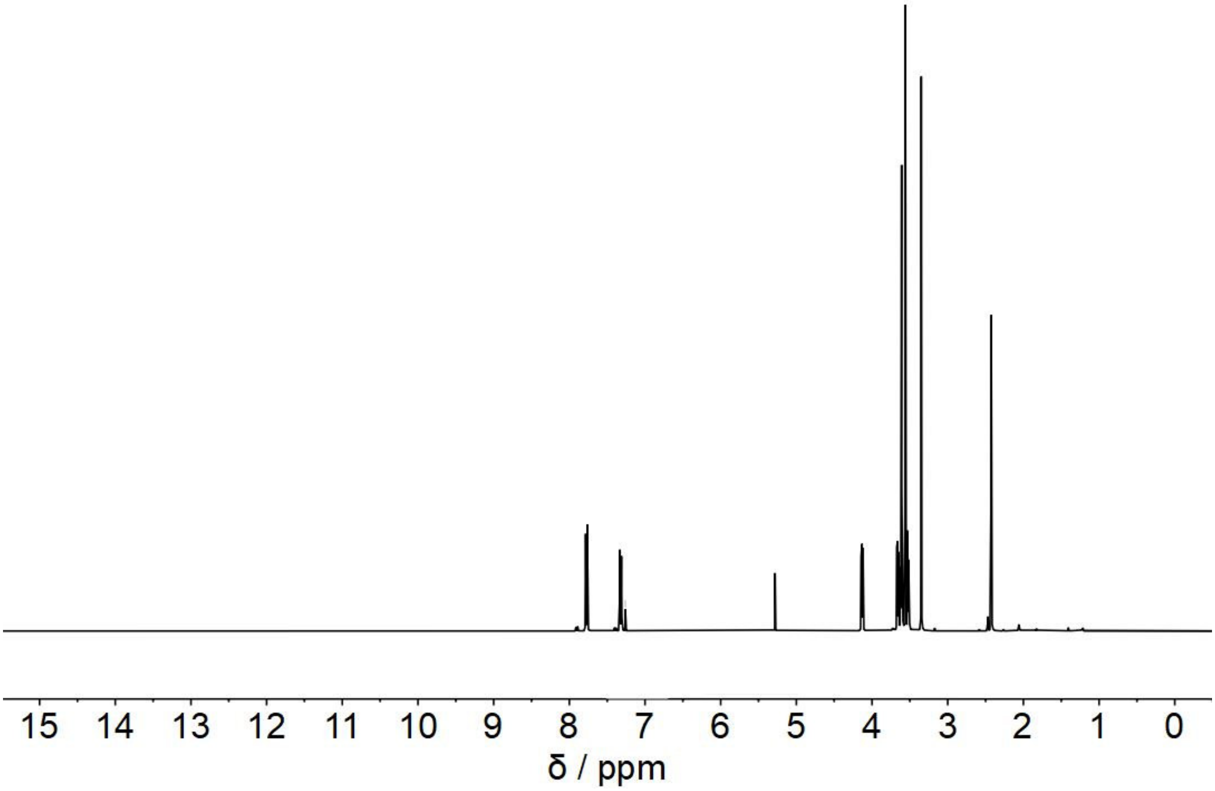

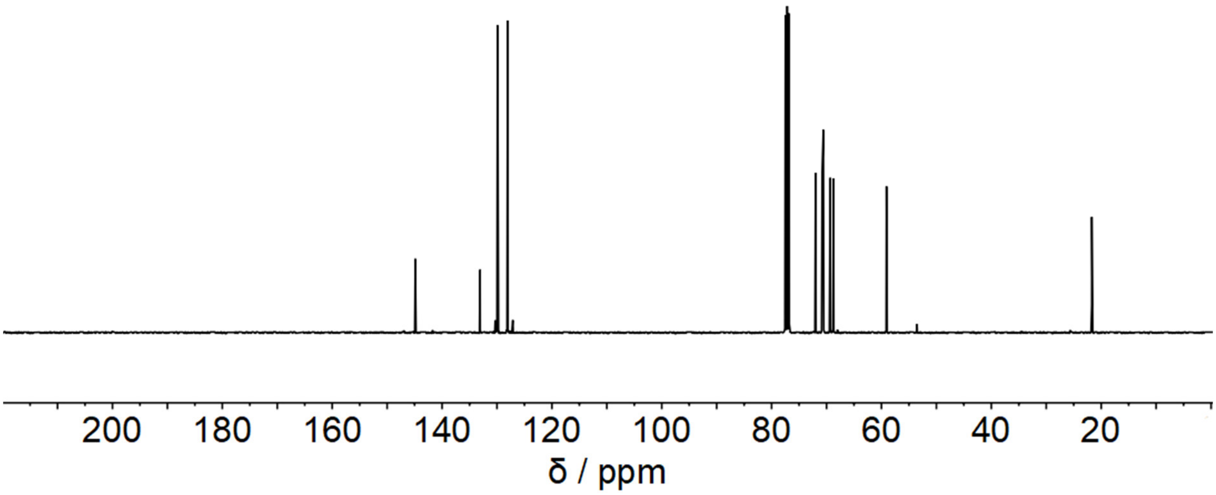

Figure 93 NMR spectra of compound **6a** (400 MHz, $CDCl_3$): ^1H NMR spectrum (top) and ^{13}C NMR spectrum (bottom).

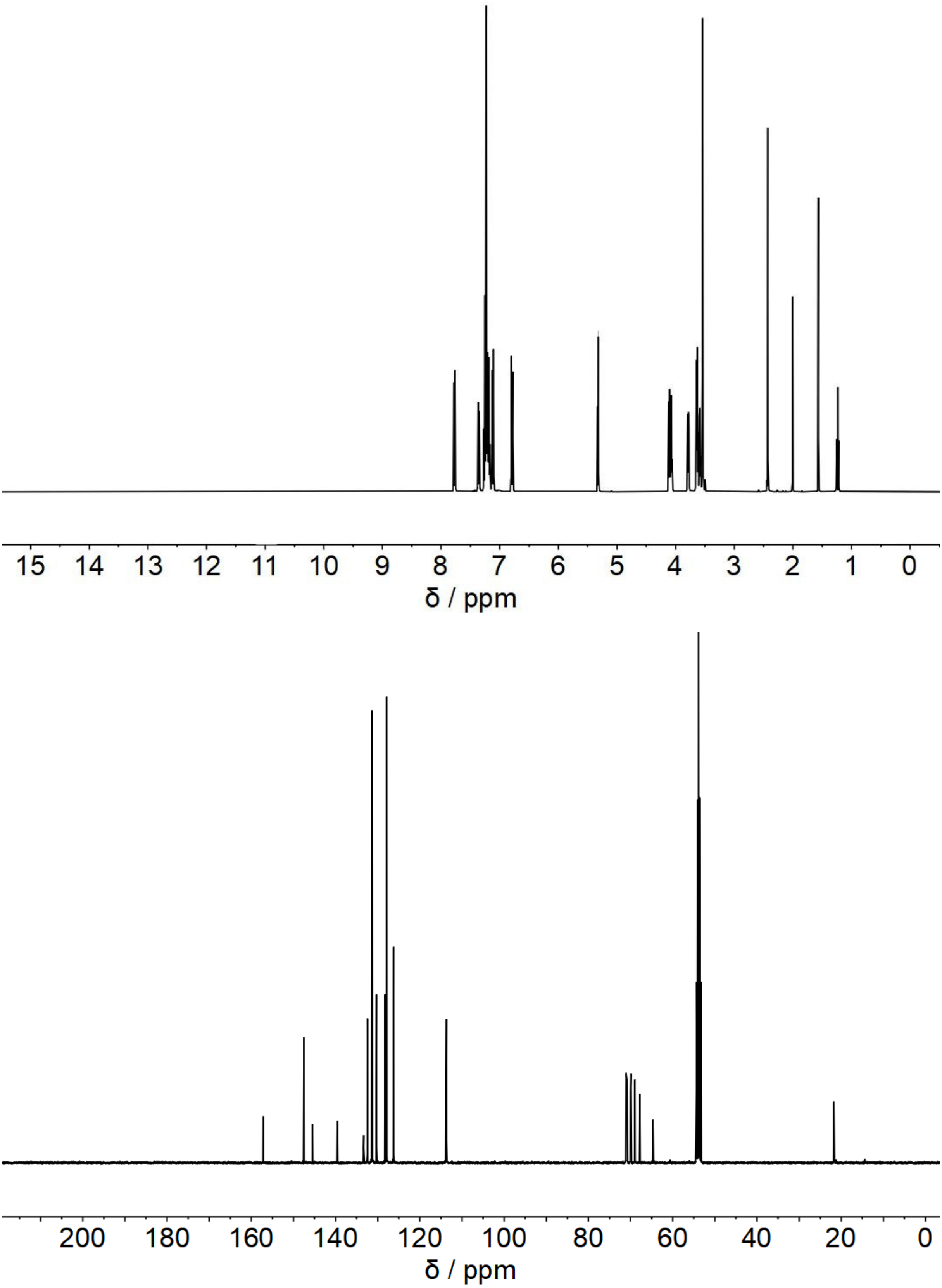

Figure 94 NMR spectra of compound **6b** (400 MHz, CD_2Cl_2): ^{1}H NMR spectrum (top) and ^{13}C NMR spectrum (bottom).

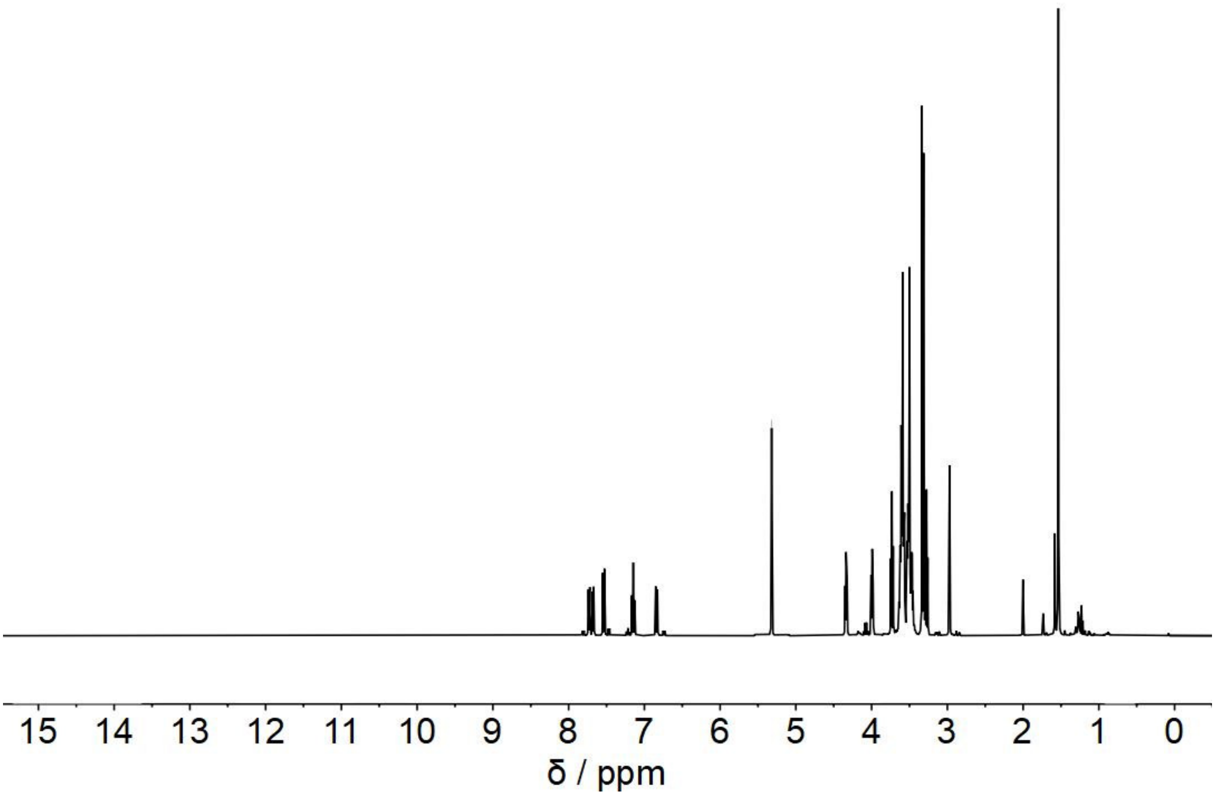

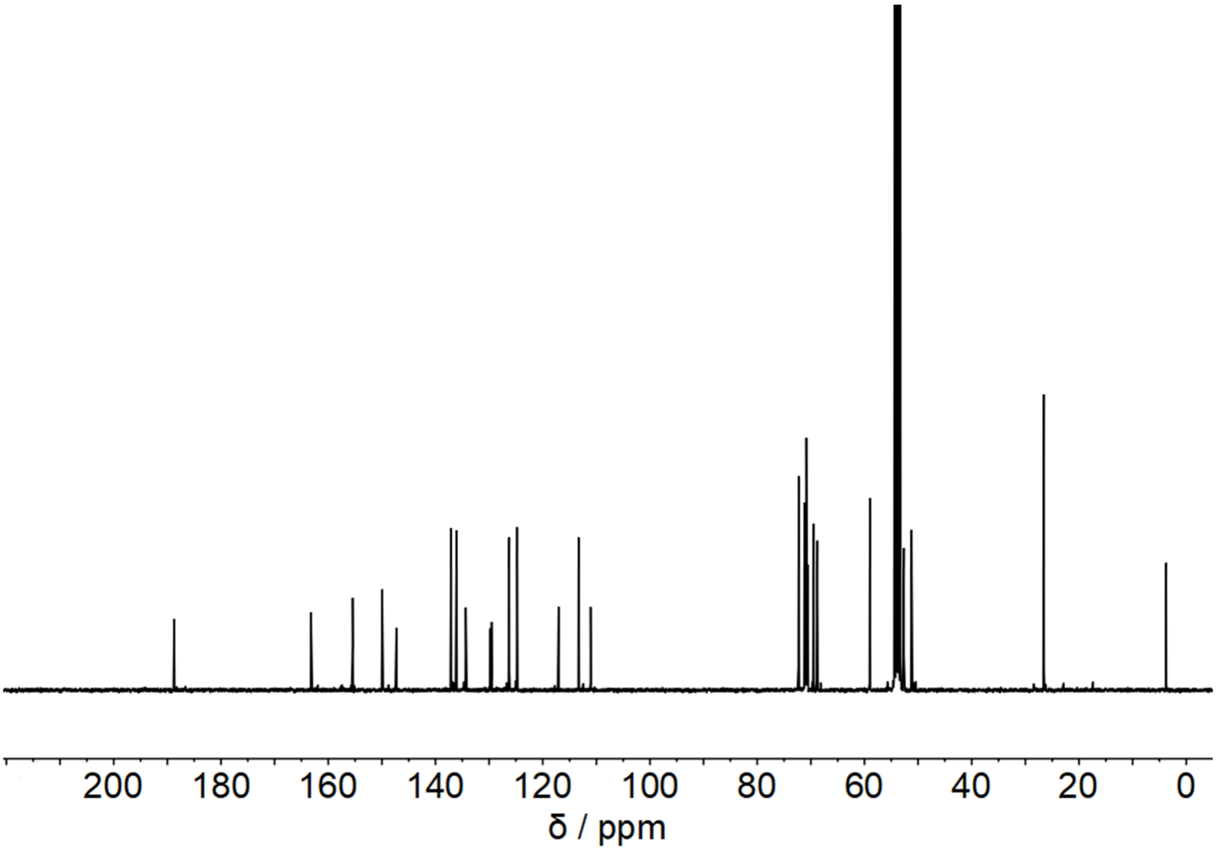

Figure 95 NMR spectra of compound **(*Z*)-7a** (400 MHz, CD_2Cl_2): ^{1}H NMR spectrum (top) and ^{13}C NMR spectrum (bottom).

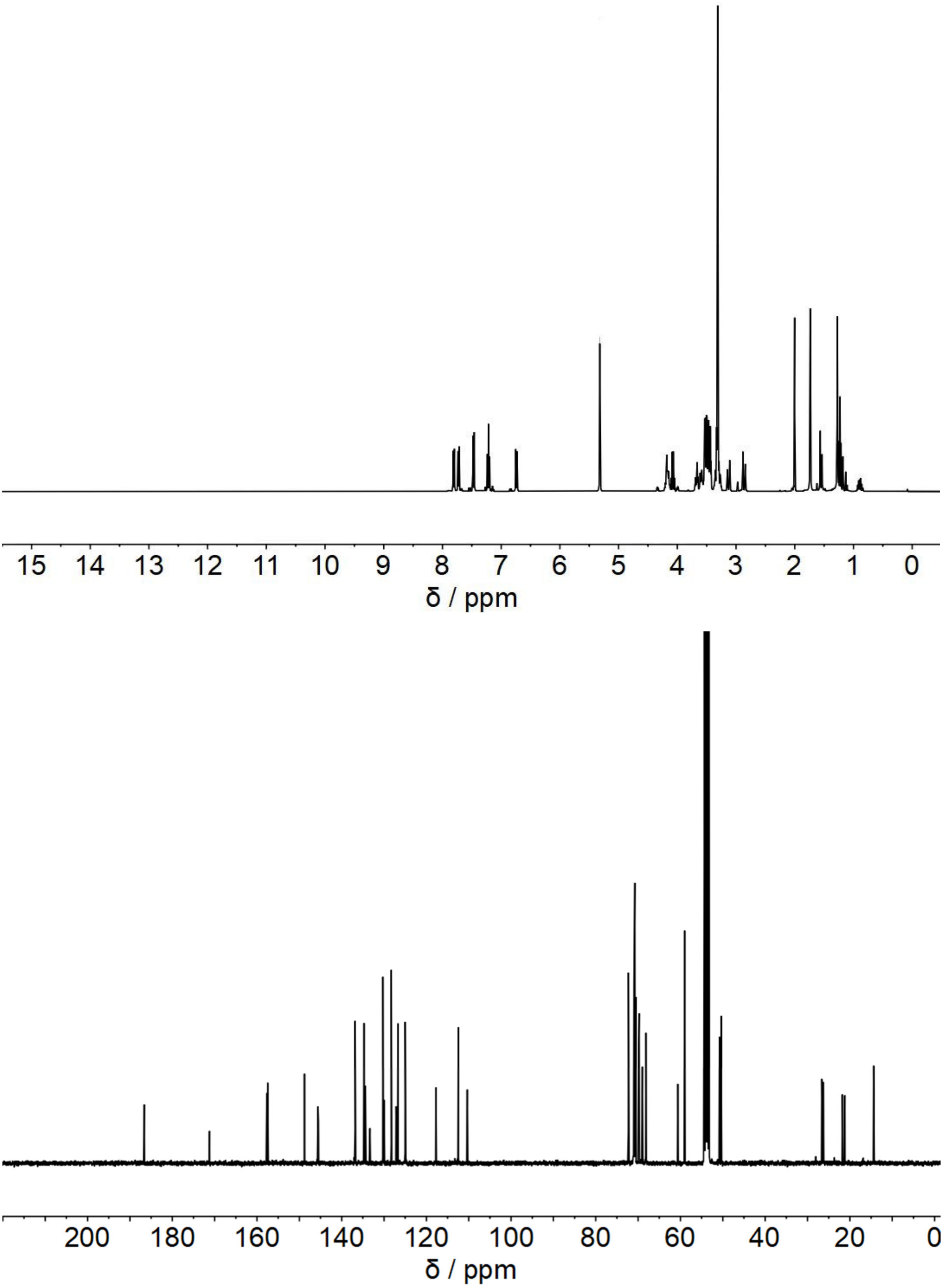

Figure 96 NMR spectra of compound **(*E*)-7a** (400 MHz, CD_2Cl_2): ^{1}H NMR spectrum (top) and ^{13}C NMR spectrum (bottom).

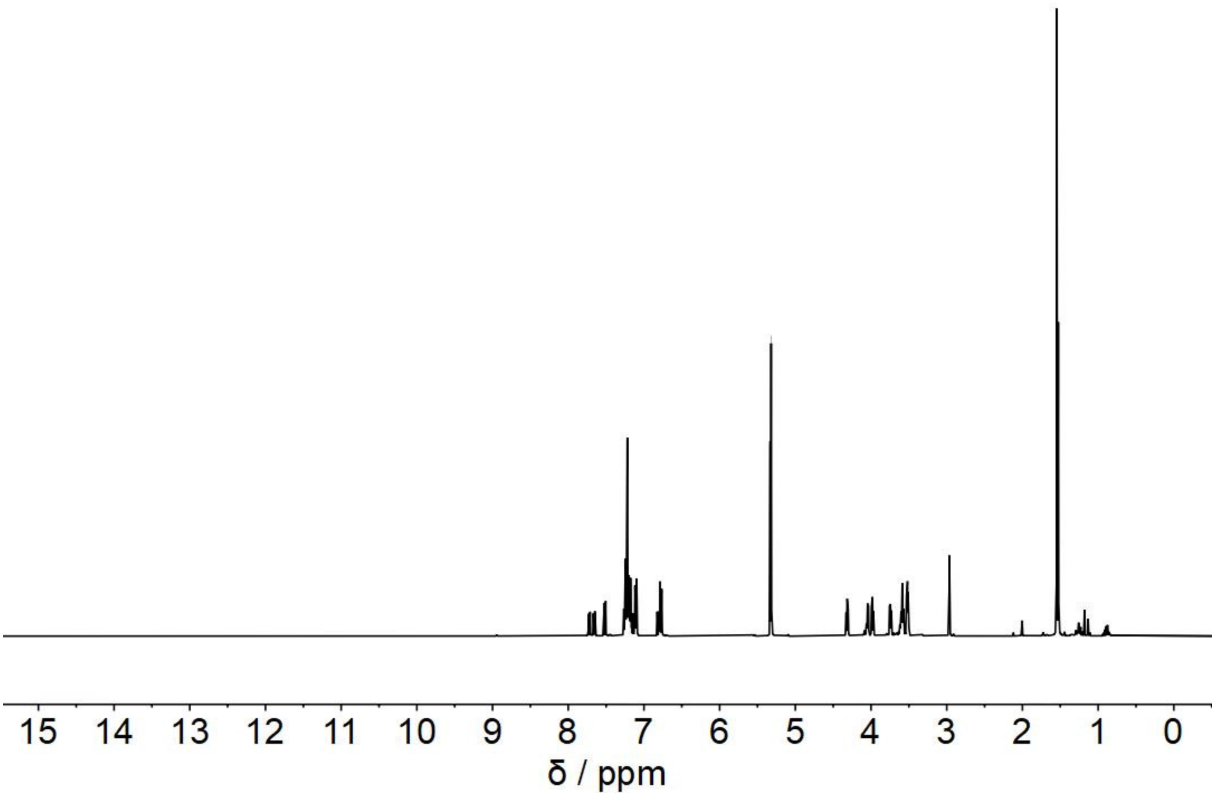

Figure 97 ^{1}H NMR (400 MHz, CD_2Cl_2) spectrum of compound ***(Z)*-7b**.

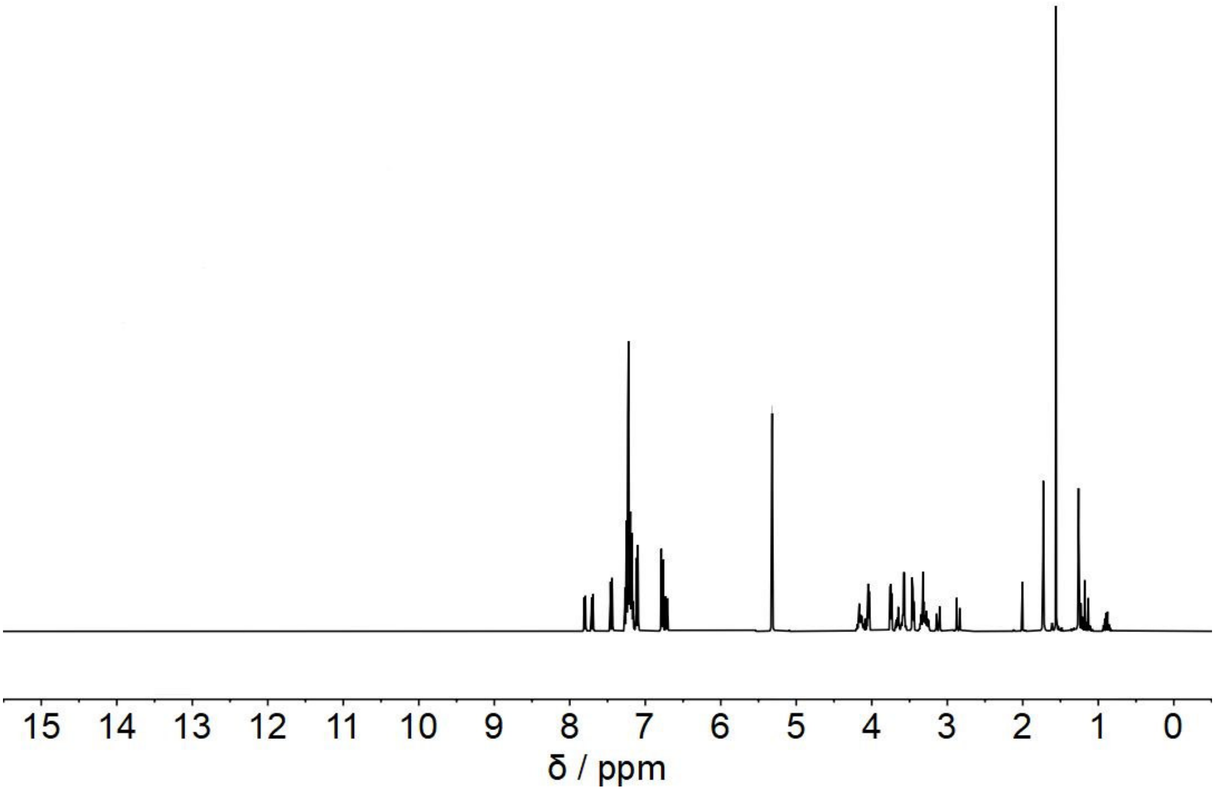

Figure 98 ^{1}H NMR (400 MHz, CD_2Cl_2) spectrum of compound ***(E)*-7b**.

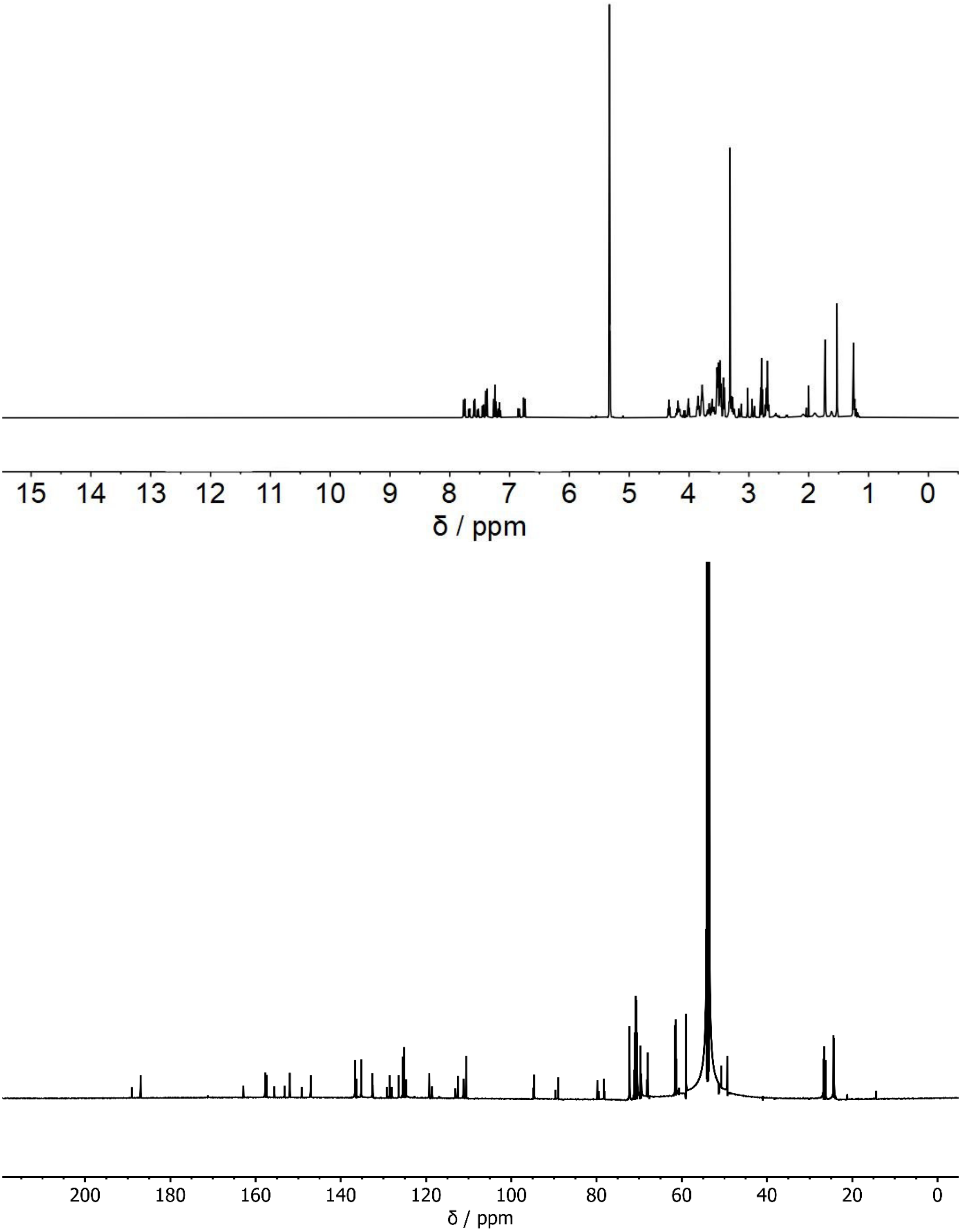

Figure 99 NMR spectra of compound **8a** (mixture of (*E*)- and (*Z*)-isomers, 800 MHz, CD_2Cl_2): ^{1}H NMR spectrum (top) and ^{13}C NMR spectrum (bottom).

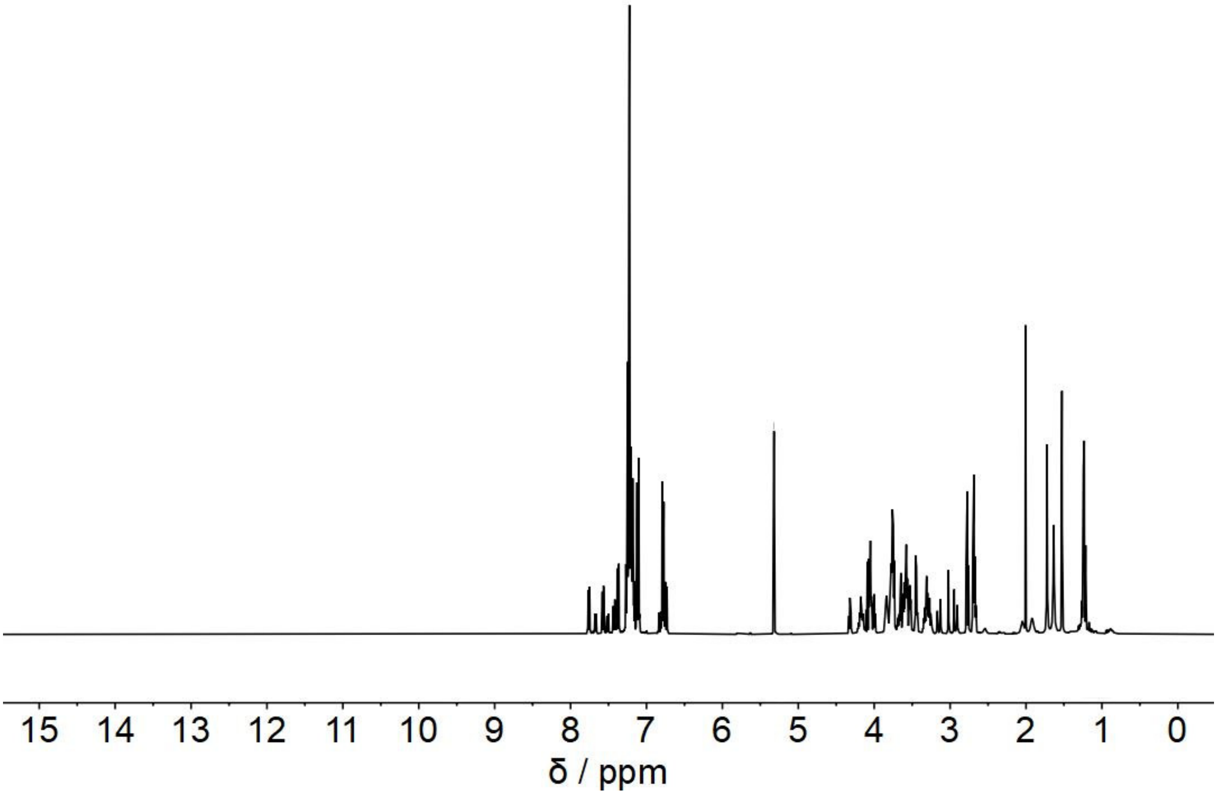

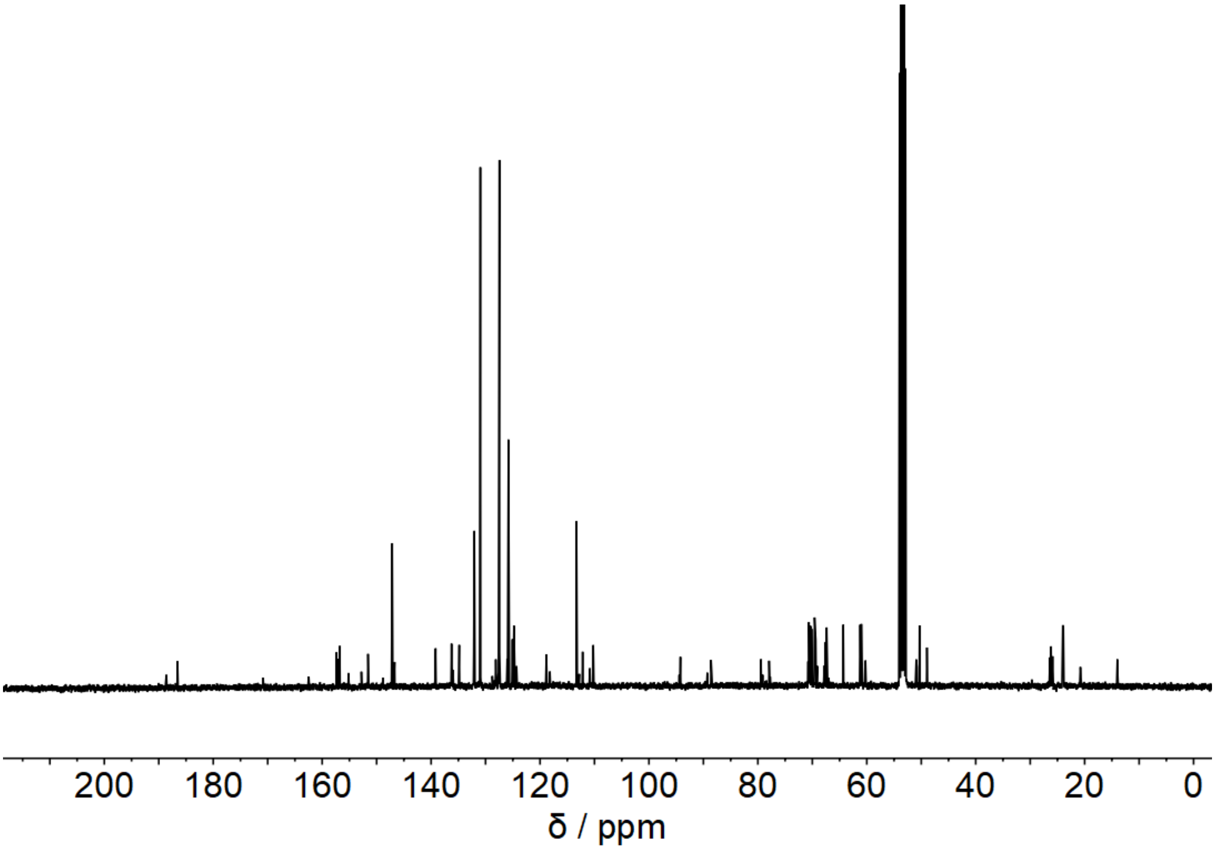

Figure 100 NMR spectra of compound **8b** (mixture of (*E*)- and (*Z*)-isomers, 400 MHz, CD2Cl2): ^{1}H NMR spectrum (top) and ^{13}C NMR spectrum (bottom).

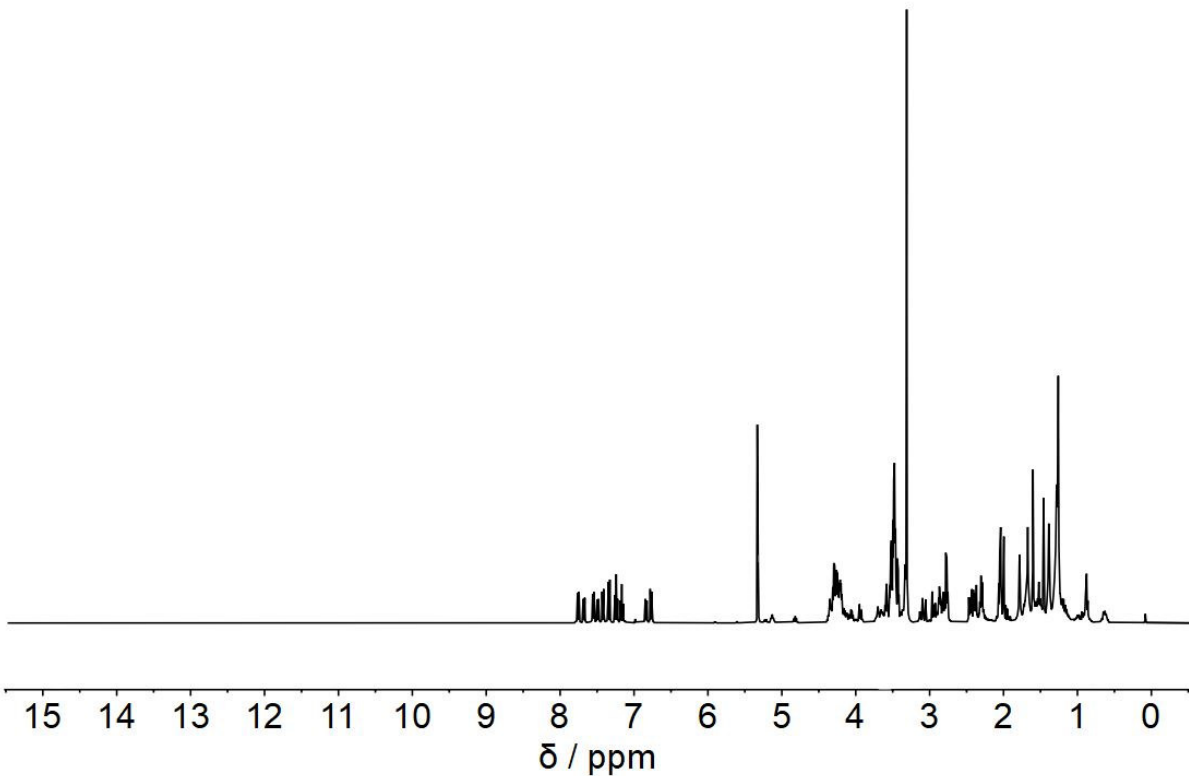

Figure 101 ^{1}H NMR spectrum (mixture of (*E*)- and (*Z*)-isomers, 400 MHz, CD_2Cl_2) of compound **9a**.

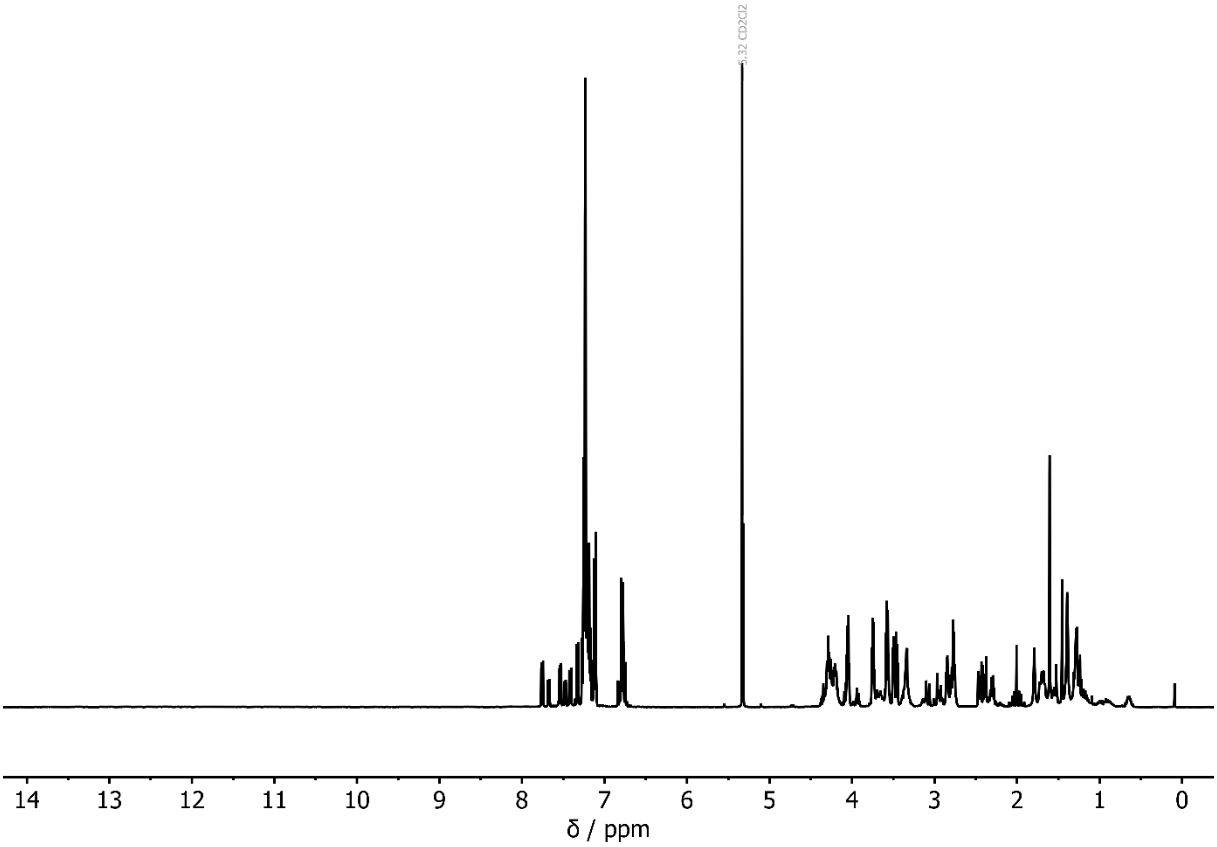

Figure 102 ^{1}H NMR spectrum (mixture of (*E*)- and (*Z*)-isomers, 400 MHz, CD_2Cl_2) of compound **9b**.

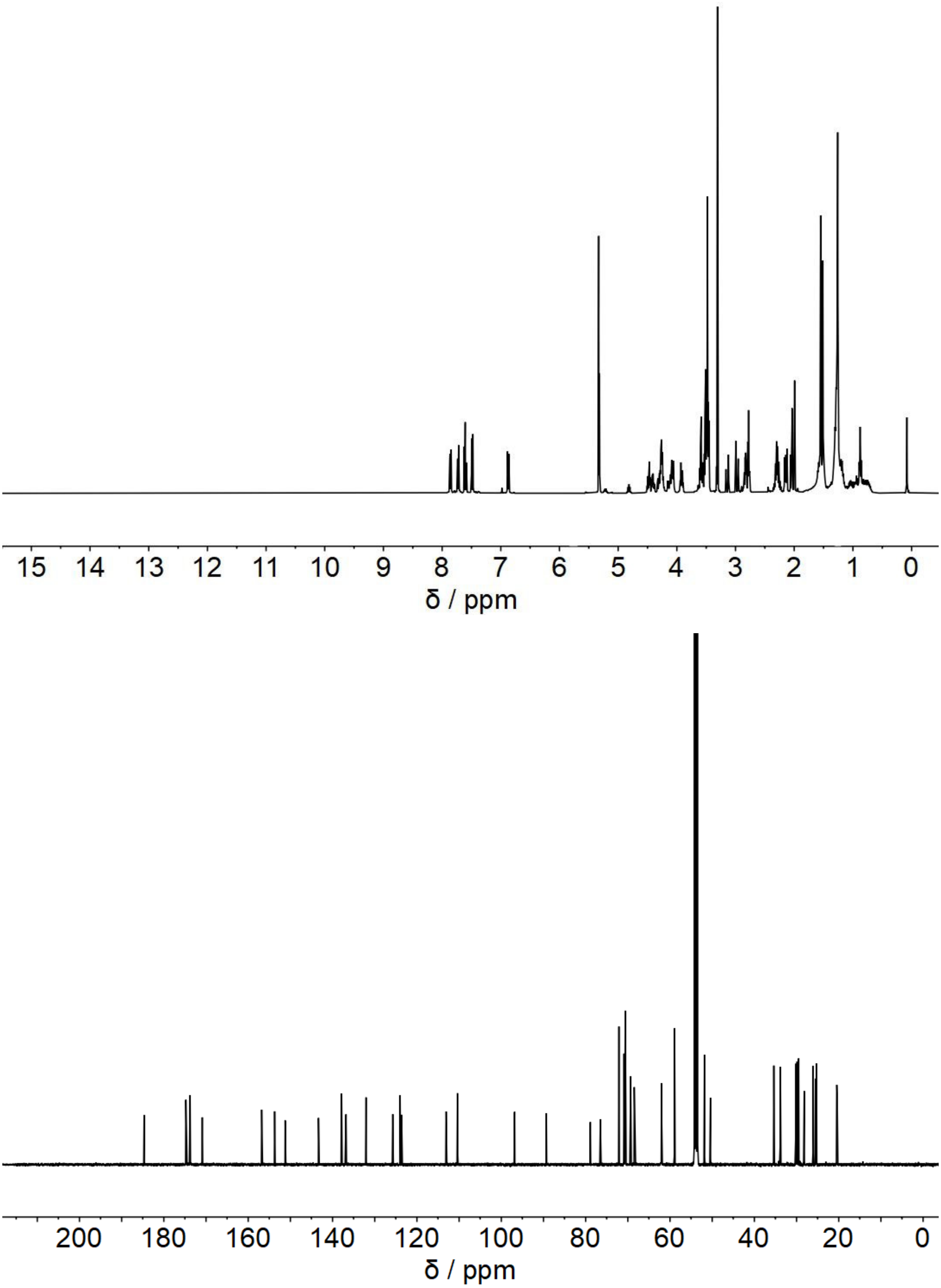

Figure 103 NMR spectra of compound **A-1** (600 MHz, CD_2Cl_2): ^1H NMR spectrum (top) and ^{13}C NMR spectrum (bottom).

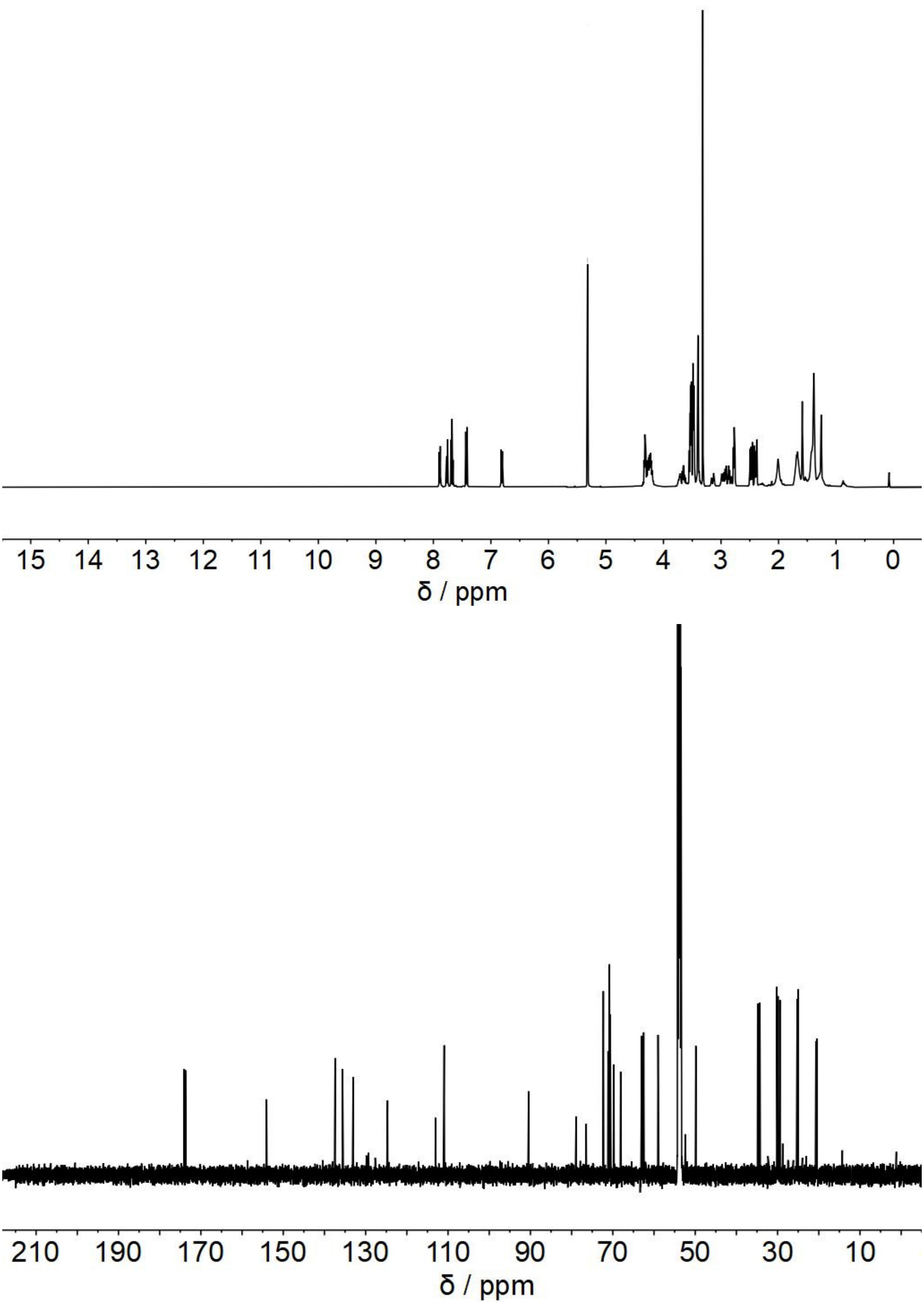

Figure 104 NMR spectra of compound **C-1** (600 MHz, CD_2Cl_2): ^{1}H NMR spectrum (top) and ^{13}C NMR spectrum (bottom).

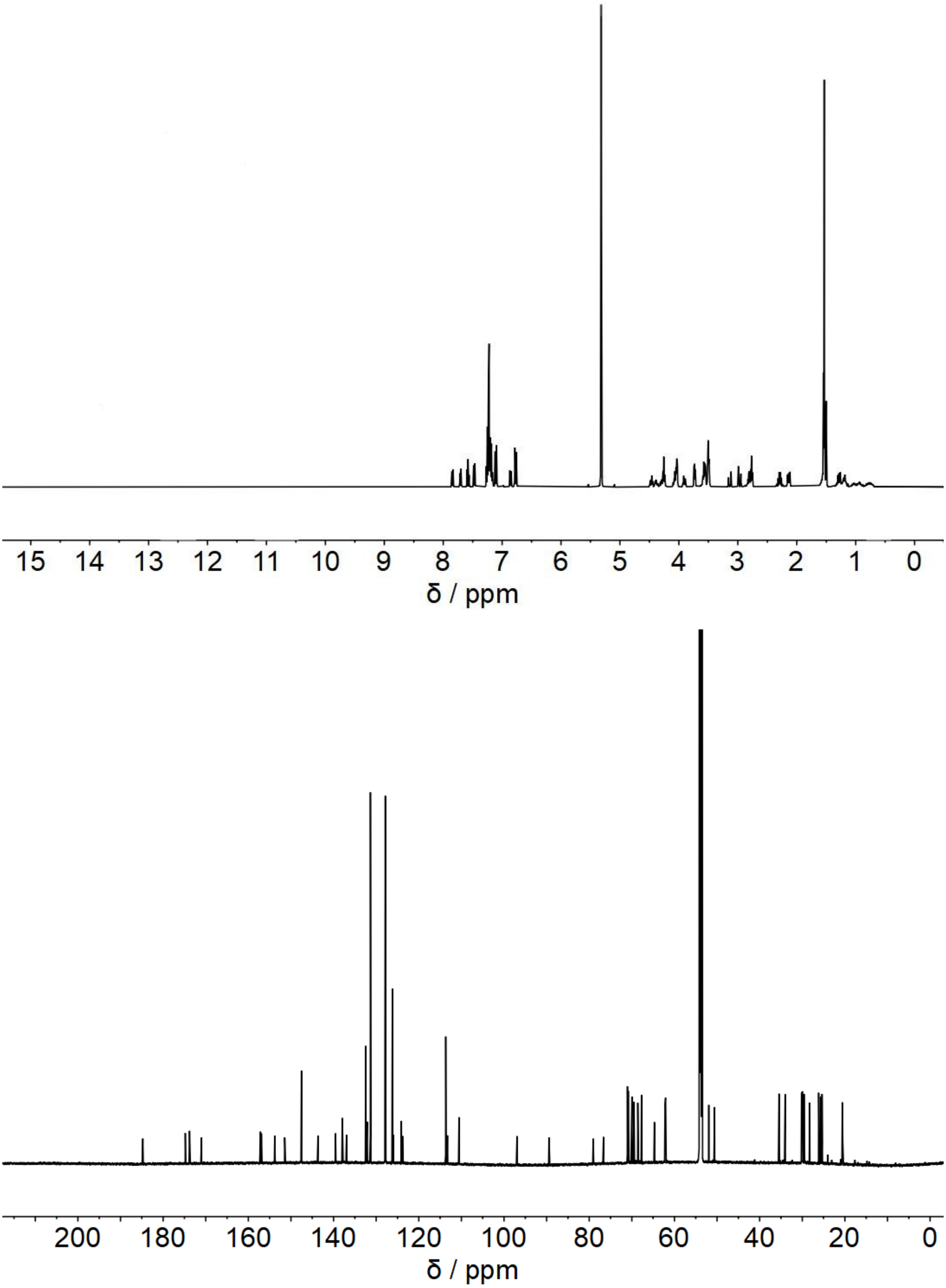

Figure 105 NMR spectra of compound **A-2** (800 MHz, CD_2Cl_2): ^1H NMR spectrum (top) and ^{13}C NMR spectrum (bottom).

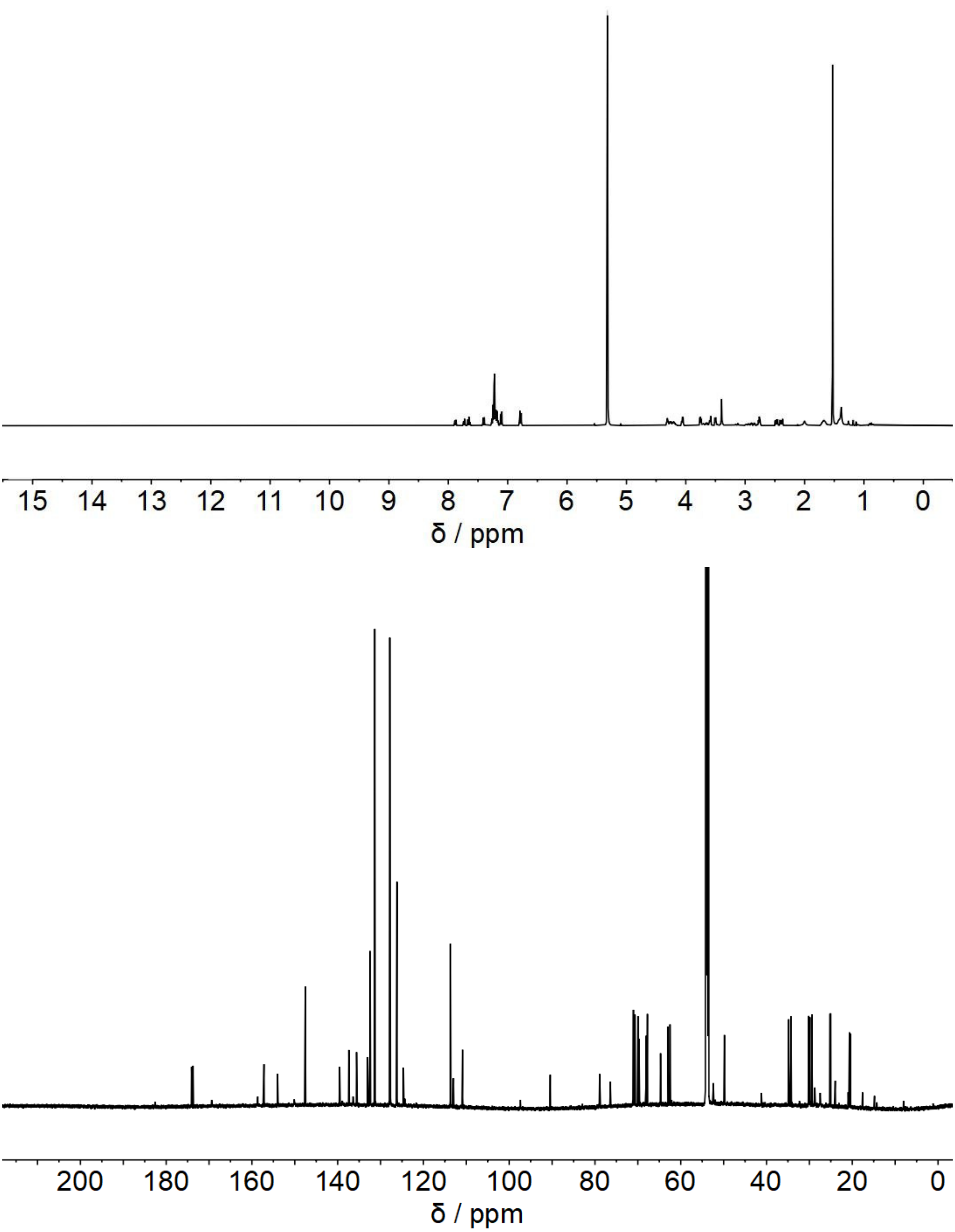

Figure 106 NMR spectra of compound **C-2** (800 MHz, CD_2Cl_2): ^1H NMR spectrum (top) and ^{13}C NMR spectrum (bottom).

References

[1] S. Wiedbrauk, T. Bartelmann, S. Thumser, P. Mayer, H. Dube, *Nat. Commun.* **2018**, *9*, 1456.

[2] P. Tegeder, M. Freitag, K. M. Chepiga, S. Muratsugu, N. Möller, S. Lamping, M. Tada, F. Glorius, B. J. Ravoo, *Chem. Eur. J.* **2018**, *24*, 18682-18688.

[3] V. Balzani, J. Becher, A. Credi, M. B. Nielsen, F. M. Raymo, J. F. Stoddart, A. M. Talarico, M. Venturi, *J. Org. Chem.* **2000**, *65*, 1947-1956.

[4] M. D. Tzirakis, M. N. Alberti, H. Weissman, B. Rybtchinski, F. Diederich, *Chem. Eur. J.* **2014**, *20*, 16070-16073.

[5] S. Hoops, S. Sahle, R. Gauges, C. Lee, J. Pahle, N. Simus, M. Singhal, L. Xu, P. Mendes, U. Kummer, *Bioinform.* **2006**, *22*, 3067-3074.

[6] Gaussian 16, Revision B.01, M. J. Frisch, G. W. Trucks, H. B. Schlegel, G. E. Scuseria, M. A. Robb, J. R. Cheeseman, G. Scalmani, V. Barone, G. A. Petersson, H. Nakatsuji, X. Li, M. Caricato, A. V. Marenich, J. Bloino, B. G. Janesko, R. Gomperts, B. Mennucci, H. P. Hratchian, J. V. Ortiz, A. F. Izmaylov, J. L. Sonnenberg, D. Williams-Young, F. Ding, F. Lipparini, F. Egidi, J. Goings, B. Peng, A. Petrone, T. Henderson, D. Ranasinghe, V. G. Zakrzewski, J. Gao, N. Rega, G. Zheng, W. Liang, M. Hada, M. Ehara, K. Toyota, R. Fukuda, J. Hasegawa, M. Ishida, T. Nakajima, Y. Honda, O. Kitao, H. Nakai, T. Vreven, K. Throssell, J. A. Montgomery, Jr., J. E. Peralta, F. Ogliaro, M. J. Bearpark, J. J. Heyd, E. N. Brothers, K. N. Kudin, V. N. Staroverov, T. A. Keith, R. Kobayashi, J. Normand, K. Raghavachari, A. P. Rendell, J. C. Burant, S. S. Iyengar, J. Tomasi, M. Cossi, J. M. Millam, M. Klene, C. Adamo, R. Cammi, J. W. Ochterski, R. L. Martin, K. Morokuma, O. Farkas, J. B. Foresman, and D. J. Fox, Gaussian, Inc., Wallingford CT, **2016**.

[7] T. Bruhn, A. Schaumlöffel, Y. Hemberger, G. Pescitelli, *SpecDis*, Version 1.71, Berlin, Germany, **2017**.

[8] R. Wilcken, M. Schildhauer, F. Rott, L. A. Huber, M. Guentner, S. Thumser, K. Hoffmann, S. Oesterling, R. de Vivie-Riedle, E. Riedle, H. Dube, *J. Am. Chem. Soc.* **2018**, *140*, 5311-5318.
